# Helicate-to-tetrahedron transformation of chiral lanthanide supramolecular complexes induced by ionic radii effect and linker length

**DOI:** 10.1038/s42004-021-00553-8

**Published:** 2021-08-05

**Authors:** King-Him Yim, Chi-Tung Yeung, Michael R. Probert, Wesley Ting Kwok Chan, Lewis E. Mackenzie, Robert Pal, Wing-Tak Wong, Ga-Lai Law

**Affiliations:** 1 State Key Laboratory of Chemical Biology and Drug Discovery, Department of Applied Biology and Chemical Technology, The Hong Kong Polytechnic University, Kowloon, Hong Kong; 2 Chemistry, School of Natural and Environmental Sciences, Newcastle University, Newcastle Upon Tyne, UK; 3 Department of Chemistry, Durham University, Durham, UK; 4 The Hong Kong Polytechnic University Shenzhen Research Institute, Shenzhen, PR China

**Keywords:** Coordination chemistry, Self-assembly

## Abstract

Controlled formation of desired lanthanide supramolecular complexes is challenging because of the difficulties in predicting coordination geometry, as well as a labile coordination number. Herein, we explore the effect of ionic radii and linker length on supramolecular species formation. A helicate-to-tetrahedron transformation occurred between [Ln_2_**L1**_3_] and [Ln_4_**L1**_6_] (Ln = La, Sm, Eu, Gd, Tb and Lu). For six lanthanide ions, the unfavored tetrahedron [La_4_**L1**_6_] can only be observed in a concentrated mixture with the helicate [La_2_**L1**_3_] where no pure [La_4_**L1**_6_] species was isolated via crystallization. For Sm, Eu, Gd, Tb, the [Ln_4_**L1**_6_] supramolecular tetrahedron can be isolated via crystallization from diisopropyl ether. A similar result was also observed for Lu, but the tetrahedral structure was found to be relatively stable and transformed back to [Lu_2_**L1**_3_] much slower upon dissolution.  No tetrahedron formation was observed with **L3** giving rise to only [Ln_2_**L3**_3_] species, in which **L3** contains a longer and more flexible linker compared with that of **L1**. Results show that the supramolecular transformation in these systems is governed by both the ionic radii as well as the ligand design. Special focus is on both [Eu_2_**L1**_3_] and [Eu_4_**L1**_6_] which form chiral entities and exhibit interesting circular polarized luminescence.

## Abstract (web summary)

Lanthanide-based supramolecular complexes have labile and hard-topredict chemical structures. Here the authors show controlled formation of either helicates or tetrahedron shaped cages based on the interplay between the metal ionic radius and the ligand composition.

## Introduction

Self-assembly of various supramolecular architectures has received phenomenal attention over recent years. This is primarily due to the diversity in the range of applications for the resultant materials such as catalysts^1,2^, luminescent probes^3,4^, and magnetic materials^5^. This interest has been renewed by the work on molecular machines by the Nobel laureates Sauvage, Stoddart, and Feringa. Hence literature has shown an emerging number of self-assembled supramolecular edifices such as helicates^6–8^, cages^9–12^, and metal organic frameworks which have been built based on transition metals^13^. Although numerous examples of supramolecular structures have been reported, the precise control and formation of the target supramolecular architectures still remains a challenge especially with the *f*-elements. As supramolecular architectures are self-assembled by spontaneous assembly of noncovalent interactions^14^, typically, some unwanted thermodynamically stable clusters are formed. Therefore, ligand design is crucial to avoid or reduce the formation of undesired structural topologies.

In order to achieve the specific structural and photophysical properties for desired supramolecules, Raymond and co-workers have proposed a design principle based on the symmetry relationship between the ligand and metal ion^15^. For example, the use of *C*_2_-symmetrical bis-bidentate ligands is ideal for preparing M_2_L_3_ helicates. When three *C*_2_-symmetrical ligands coordinate with two metal ions, two metals ions will share the same C_3_ axis and three *C*_2_ axis will be perpendicular to the *C*_3_ axis^16^. Therefore, two chelating groups of the *C*_2_-symmetrical bis-bidentate ligand must be parallel and the offset of the two chelating group in the ligand should be small so that the *C*_3_ and *C*_2_ axis can approach 90° to form a helicate^17,18^.

A similar approach can also be applied for the design of M_4_L_6_ structures. For M_4_L_6_ tetrahedral cages, four metal ions act as the vertices and six *C*_2_-symmetric bis-bidentate ligands act as the edges. The *C*_2_ and *C*_3_ axis is rigidly fixed by the ligand design, and the ideal approach angle between the *C*_3_ axis and the extended *C*_2_ ligand plane has been calculated to be 35.3°^18^. Since entropy favors the lower stoichiometry M_2_L_3_ complex^15^, the metal binding units of the ligand should be offset from one another so that the helicate formation is disfavored^17^.

Although supramolecular structures can be prepared by the above approach, lanthanide-based supramolecular architectures are still less commonly reported compared with transition metals based supramolecules^19–22^. By contrast to transition metals, which have a strong directional coordination nature, lanthanide ions also exhibit labile coordination numbers and poor stereochemical preferences which make it more challenging to control their assemblies^20,23^. However, the unique chemical and physical properties of lanthanide ions such as optical and magnetic features which have shown promising application in luminescent sensing and MRI contrasting agent have made it a worthy research field to explore^24–27^.

**Fig. 1:**
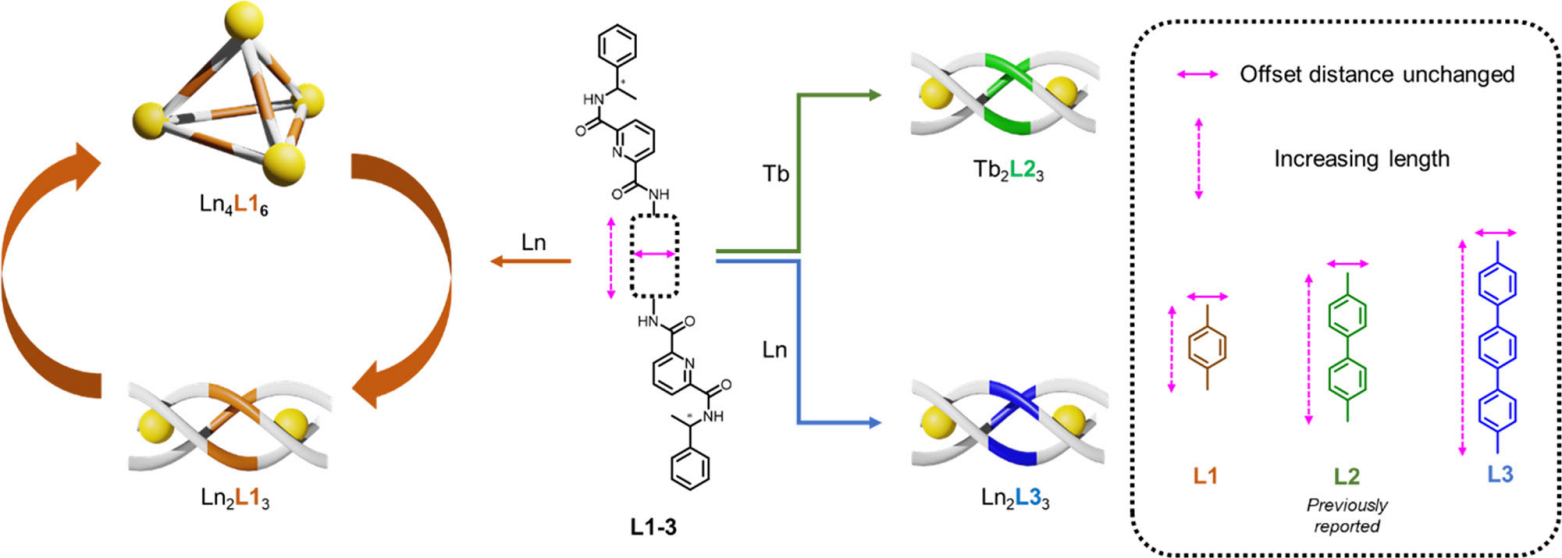
Supramolecular formation of [Ln_2_**L1**_3_], [Ln_4_**L1**_6_]. [Tb_2_**L2**_3_], and [Ln_2_**L3**_3_]. The length of the spacer will greatly affect the supramolecular formation. With the use of a shorter ligand **L1**, helicate-to-tetrahedron transformation was observed. For longer **L2** and **L3**, only stable helicates were formed and no helicate-to-tetrahedron transformation was observed.

Although preparing lanthanide supramolecular complexes is more challenging compared with those of the transition metals, most of the reported M_4_L_6_ cases obey the design principle that has been proposed by Raymond’s group^28–30^. Most of the reported M_4_L_6_ lanthanide tetrahedral cages were successfully prepared because of the offset of two chelating groups created by a rigid spacer. Sun and co-workers also pointed out the importance of the offset distance for preparation of cages and they showed the evolution from cage to cube by increasing the offset distance^30^. (Fig. 1).

In this study, two *C*_2_-symmetric bis-bidentate ligands were designed based on the symmetry principles discussed and they were expected to form stable helicate structures. We found that the length of the spacer will greatly affect the supramolecular formation even if the offset distance remained unchanged. Moreover, the slight change in ionic radii also govern the supramolecular formation as well as the stability of the complexes.

## Results and discussion

### Ligand design and synthesis

Previously we have reported that **L2** can form enantiomeric pure lanthanide bimetallic triple helicates by point chirality effect^31^. Based on this finding, we varied the length of the linker and observed the changes in supramolecular formation. **L1** and **L3** were designed based on two pyridine-2,6-dicarboxamide (pcam) moieties, which tends to form stable nine-coordinated lanthanide complexes to avoid the lability of Ln^III^ ions^32^. Two chiral metal-chelating pcam moieties are connected with a rod-like monophenyl linker to form **L1**, resulting in a shorter moiety than **L2**. The longer ligand was prepared by coupling two chiral pcam moieties with a triphenyl linker to form **L3**. The reported X-ray crystal structure of [Eu_2_**L2**_3_] revealed that two europium ions shared the same *C*_3_ axis which obey the symmetry design principle^31^. Therefore, we expected that **L1** and **L3** could also form a stable helicate since the offset distance of two chelating group did not change. Two pairs of the new ligands **L1**^**R/S**^ and **L3** ^**R/S**^ were prepared in two steps using generic HATU peptide coupling reactions. **L1** and **L3** were fully characterized by NMR and ESI-HRMS.

### ^1^H NMR titration and UV–Vis titration

The metal to ligand ratio and the solution-state supramolecular formation was first investigated by ^1^H NMR and UV-CD titration. A hypothesis of a metal to ligand ratio of 2:3 was investigatied by monitoring the change of proton NMR signal. The ^1^H NMR signal at 1.7 ppm, which corresponds to the methyl group of the ligand, decreased gradually upon addition of Eu(OTf)_3_ to a solvent mixture containing **L1** and a new siganl at 2.1 ppm in the aliphatic region increased progressively. At 0.67 eq., all the ligand signal disappeared appearing to confirm the hypothesis. The proton NMR titration was completed in low ligand concentration (1.09 × 10^−3^ M) and only a single species was observed, which also confirmed that pure [Eu_2_**L1**_3_] can be prepared under low ligand concentration. UV–Vis titration was performed in a solvent mixture of CHCl_3_/MeOH/MeCN (12:1:12, v/v/v) and the result showed a progressive decrease of the ligand absorption (281, 315, 353 nm) upon addition of Eu(OTf)_3_ to the ligand solution (Supplementary Fig. 1). Simultaneously, a smooth evolution of absorption was observed at 320 nm corresponding to the complex. An end point ~0.67 was observed at four wavelengths in plots showing the changes of molar absorptivity as a function of total equivalents of Eu(OTf)_3_.

**Fig. 2:**
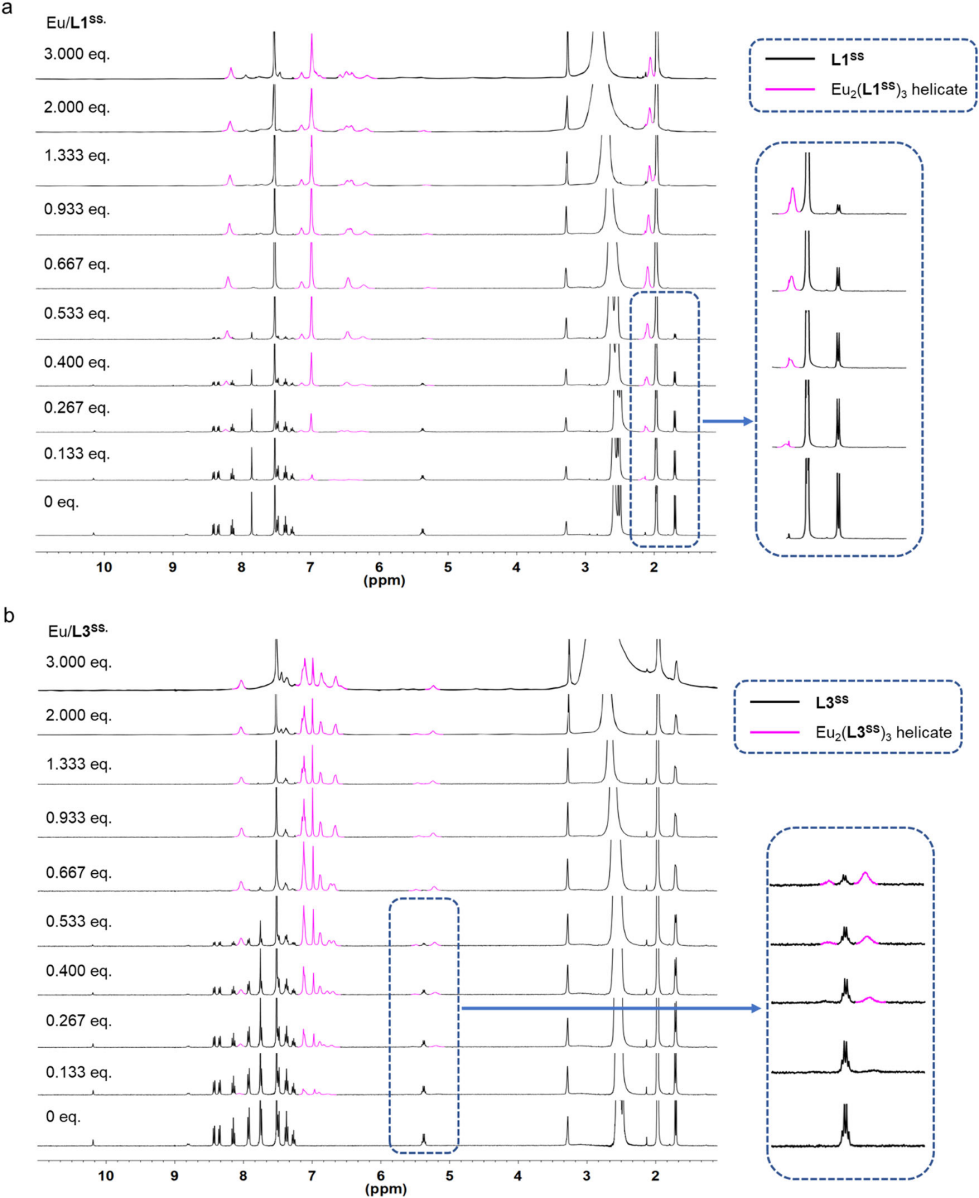
^1^H NMR titrations of **L1** and **L3** with Eu(OTf)_3_. **a** Variation in ^1^H NMR spectra of **L1**^**SS**^ (1.09 × 10^−3^ M in 50:5:45, v/v/v of CDCl_3_/CD_3_OD/CD_3_CN) with Eu(OTf)_3_ (0.108 M in CD_3_OD) at 298 K. When Eu/**L1**^**SS**^ = 0.667 eq., all the ligand signals disappeared which confirmed the metal to ligand ratio is 2–3. **b** Variation in ^1^H NMR spectra of **L3**^**SS**^ (8.80 × 10^−4^ M in 50:5:45, v/v/v of CDCl_3_/CD_3_OD/CD_3_CN) with Eu(OTf)_3_ (0.090 M in CD_3_OD) at 298 K (bottom). When Eu/**L3**^**SS**^ = 0.667 eq., all the ligand signals disappeared which confirmed the metal to ligand ratio is 2–3.

For ligand **L3**, the expected metal to ligand ratio 2:3 was investigated by proton NMR signal (Fig. 2b). The ^1^H NMR signal at 5.3 ppm corresponding to the chiral proton of the ligand decreased gradually upon addition of Eu(OTf)_3_. A new chiral proton signal at 5.5 ppm increased progressively and all the ligand signal disappeared at 0.67 eq., again suggesting confirmation of the hypothesis. UV–Vis titration was performed and the result also showed an end point at ~0.67 (Supplementary Fig. 2).

### Synthesis of [Ln_2_**L1**_3_]

Following the previously described protocol, the corresponding Eu metal complex was synthesized by treating 8 × 10^−3^ M ligand with 0.67 equivalents of Eu(OTf)_3_ in a solution of CDCl_3_/MeOD/CD_3_CN (12/1/12, v/v/v). The resulting complex [Eu_2_**L1**_3_] was first characterized by ^1^H NMR spectroscopy. Comparison of the proton NMR of the free ligand, most signals arising from the europium complexes are shifted, that confirmed the formation of paramagnetic europium complex. However, the ^1^H NMR resonances resolved into two sets of signals in a ratio of ~96:4. The minor species was disappeared after dissolution in CD_3_CN for 3 days. Similar result was also obtained without the use of MeOH (Supplementary Fig. 3). When counteranion was changed to perchlorate and chloride, formation of Eu_2_**L1**_3_ was observed. However, the helicate was not stable for further investigation when either perchlorate or chloride was used as counteranion (Supplementary Figs. 4–6). The supramolecular formation is highly sensitive to the ligand concentration. In a lower ligand concentration (1 × 10^−3^ M), only single major species was observed (Supplementary Fig. 7). The concentration effect was also evidenced by another proton NMR titration in which the minor species can be observed when 2.15 × 10^−3^ M **L1** was titrating with Eu(OTf)_3_ (Supplementary Fig. 8). Attempt to obtain pure major species by increasing the ligand concentration was not successful due to the poor ligand solubility. Elevating the reaction temperature will not promote the formation of minor species. The concentration effect was further investigated by dissolving helicate into CD_3_CN and monitored by proton NMR. Increasing the helicate concentration will lead to an increase in formation of minor species. However, the limit of major species to minor species ratio was found to be 2.9:1 when ligand concentration is 88.9 mM. Further increase in ligand concentration will not change the major species to minor species ratio significantly (Supplementary Fig. 9). The major species was then characterized by ESI-HMRS. The ESI-HRMS revealed one set of independent peak series, corresponding to the dinuclear species [Eu_2_**L1**_3_]. [Eu_2_**L1**_3_] showed three different clusters of peaks which correspond to three different species with +2, +3, and +4 charged states. Each cluster was found to correspond to the species with progressive loss of triflate anion and proton cation. For example, peaks with *m/z* equal to 1369.1962, 1294.2137, and 1219.2385 can be assigned to the species of [{[Eu_2_**L1**^**SS**^_3_](OTf)_m_}-H_n_]^2+^ with *m* = 4, *n* = 0; *m* = 3, *n* = 1; *m* = 2, *n* = 2. The assignments were also confirmed by comparing the corresponding isotopic pattern of the simulated and experimental results.

When Eu was replaced by Sm using the same protocol, a similar result was observed. The crude ^1^H NMR revealed the presence of a major species and a minor species in a ratio of 94:6 (Supplementary Fig. 10). The minor species slowly transformed into major species after 3 days and the resulting complex was analyzed by ESI-HRMS. The ESI-HRMS revealed one set of independent peak series, corresponding to the dinuclear species [Sm_2_**L1**_3_]. Eu was further replaced with Gd and Tb. The desired [Gd_2_**L1**_3_] and [Tb_2_**L1**_3_] were prepared by adding 1 × 10^−3^ M ligand into the correspond Gd(OTf)_3_ and Tb(OTf)_3_. Due to the strong paramagnetic nature, characterization by NMR is not possible. The ESI-HRMS showed a dominant species corresponding to [Gd_2_**L1**_3_] and [Tb_2_**L1**_3_] with different clusters corresponding to the progressive loss of triflate anion and proton.

For La and Lu, the corresponding bimetallic helicates were synthesized by adding 2 eq. of lanthanide ions to 3 eq. of **L1**. Similar with that of [Eu_2_**L1**_3_] and [Sm_2_**L1**_3_], the crude NMR of La complexes showed two sets of signal corresponding to a major species and a minor species. Interestingly, unlike Eu (Supplementary Fig. 11), the La minor species transformed to major species immediately after dilution (Supplementary Fig. 12). For Lu, insignificant amount of minor species was observed for treatment of 0.008 M **L1** with Lu(OTf)_3_ and pure [Lu_2_**L1**_3_] can be also prepared by decreasing ligand concentration to 0.001 M (Supplementary Fig. 13). The formation of desired [La_2_**L1**_3_] and [Lu_2_**L1**_3_] was also confirmed by ESI-HRMS and no other supramolecular species can be observed in ESI-HRMS

### Synthesis of [Tb_2_**L2**_3_]

**Fig. 3:**
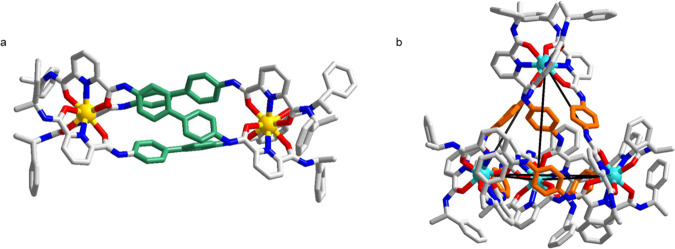
X-ray crystal structure of lanthanide supramolecular complexes. **a** X ray crystal structure of [Tb_2_**L2**^**SS**^_3_] showing a helicate structure. **b** X ray crystal structure of [Eu_4_**L1**^**SS**^_6_] showing a tetrahedron structure.

The complexation of **L2** was reported by our group previously^31^. In this study, the new complex [Tb_2_**L2**_3_] was synthesized by adding 2 eq. of Tb(OTf)_3_ to 3 eq. of **L2**. The ESI-HRMS revealed one set of independent peak series, corresponding to the dinuclear species [Tb_2_**L2**_3_] (Supplementary Fig. 14). [Tb_2_**L2**_3_] exhibits two different clusters of peaks which correspond to two different species with +3 and +4 charged states. Each cluster was found to correspond to the species with progressive loss of triflate anion and proton cation. For example, peaks with *m/z* equal to 943.5166, 893.5298, 843.5465, and 793.5624 can be assigned to the species of [{[Tb_2_**L2**^**SS**^_3_](OTf)_m_}-H_n_]^2+^ with *m* = 3, *n* = 0; *m* = 2, *n* = 1; *m* = 1, *n* = 2; *m* = 0, *n* = 3. The assignments were also confirmed by comparing the corresponding isotopic pattern of the simulated and experimental results. The supramolecular structure was further confirmed by X-ray crystallography (Fig. 3a).The single crystal structure of [Tb_2_**L2**^**SS**^_3_] is analogous to the previously reported Eu(III) complex^31^, in which three ligands **L2** envelope two terbium centers in a helical manner. Each terbium center carries a tricapped trigonal prismatic geometry (Supplementary Table 4), coordinating with one nitrogen and two oxygen atoms from each ligand. Tb-N distances range from 2.500(5) to 2.566(5) Å (average = 2.529 Å), comparable to known Tb-N average distance of 2.540 Å in the literature^33^. Likewise, the Tb-O distances in [Tb_2_(**L2**^**SS**^)_3_] [2.377(4)–2.432 (4) Å, average 2.405 Å] are also typical of those reported in literature (average: 2.401 Å).

### Synthesis of [Ln_2_**L3**_3_]

Similar to **L1** and **L2**, the corresponding [Eu_2_**L3**_3_] can be obtained by addition of two equivalents Eu(OTf)_3_ into the solution containing three equivalent of **L3**. The turbid solution turned into a clear yellowish solution rapidly indicating complexation. Similar with that of [Eu_2_**L1**_3_], NMR characterization shows that most signals arising from the europium complexes are shifted due to the coordination with the paramagnetic Eu^III^ ions which confirmed the formation of the europium complex. The ^1^H NMR displayed only single set of signals and the ESI-HRMS revealed one set of independent peak series, corresponding to the dinuclear species [Eu_2_**L3**_3_]. [Eu_2_**L3**_3_] exhibits three different clusters of peaks which correspond to three different species with +3 and +4 charged states. Each cluster was found to correspond to the species with progressive loss of triflate anion and proton cation. For example, peaks with *m/z* equal to 1015.2181, 965.2259, 915.2409, and 865.2620 can be assigned to the species of [{[Eu_2_**L3**^**SS**^_3_](OTf)_m_}-H_n_]^3+^ with *m* = 3, *n* = 0; *m* = 2, *n* = 1; *m* = 1, *n* = 2; *m* = 0, *n* = 3. The assignments were also confirmed by comparing the corresponding isotopic pattern of the simulated and experimental results. Similar results were also observed for the preparation of other lanthanide bimetallic helicates including La, Sm, Gd, Tb, and Lu. All the [Ln_2_**L3**_3_] were fully characterized by NMR and ESI-HRMS.

### Helicate-to-tetrahedron transformation

Attempt to prepare crystal of [Eu_2_**L1**^**SS**^_3_] by slow evaporation was not successful. However, a single crystal was obtained by slow diffusion of diisopropyl ether into an acetonitrile solution of the complex. Different solvents was also examined including methyl t-butyl ether, diethyl ether, ethyl acetate, dichloromethane and acetone. However, helicate-to-tetrahedron transformation was only observed when ether (methyl t- butyl ether, diethyl ether, diisopropyl ether) was used. Single X-ray diffraction revealed a [Eu_4_**L1**^**SS**^_6_] tetrahedral structure, which showed a helicate-to-tetrahedron transformation via ether crystallization (Fig. 3b). In the crystal structure of [Eu_4_**L1**^**SS**^_6_], each europium can be described to be tricapped trigonal prism (Supplementary Table 5). Four europium ions are all arranged in the same Δ-configurations, which shows the successful chiral induction from the chiral moiety to the metal center. Four europium ions occupy the vertices and six C_2_-symmetrical ligands act as the edges to form a tetrahedron topology.

**Fig. 4:**
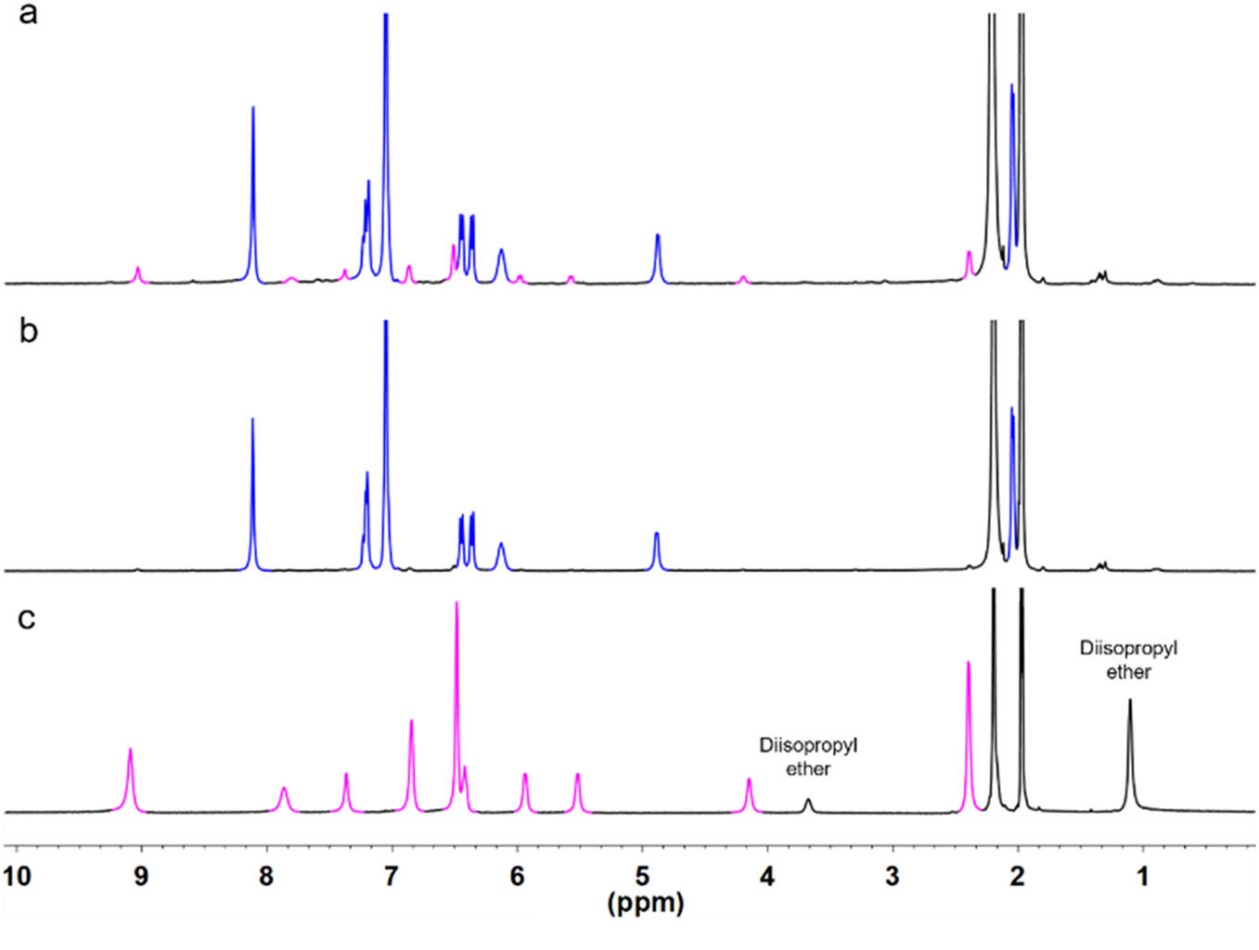
^1^H NMR of [Eu_2_**L1**_3_]/[Eu_4_**L1**_6_]. **a** ^1^H NMR of crude mixture containing both of [Eu_2_**L1**_3_] (blue) and [Eu_4_**L1**_6_] (magenta) in a ratio of 96:4. **b** ^1^H NMR showing pure [Eu_2_**L1**_3_] by dissolving crude mixture after 3 days. **c** ^1^H NMR of pure [Eu_4_**L1**_6_] that was done simultaneously after dissolution of [Eu_4_**L1**_6_].

**Fig. 5:**
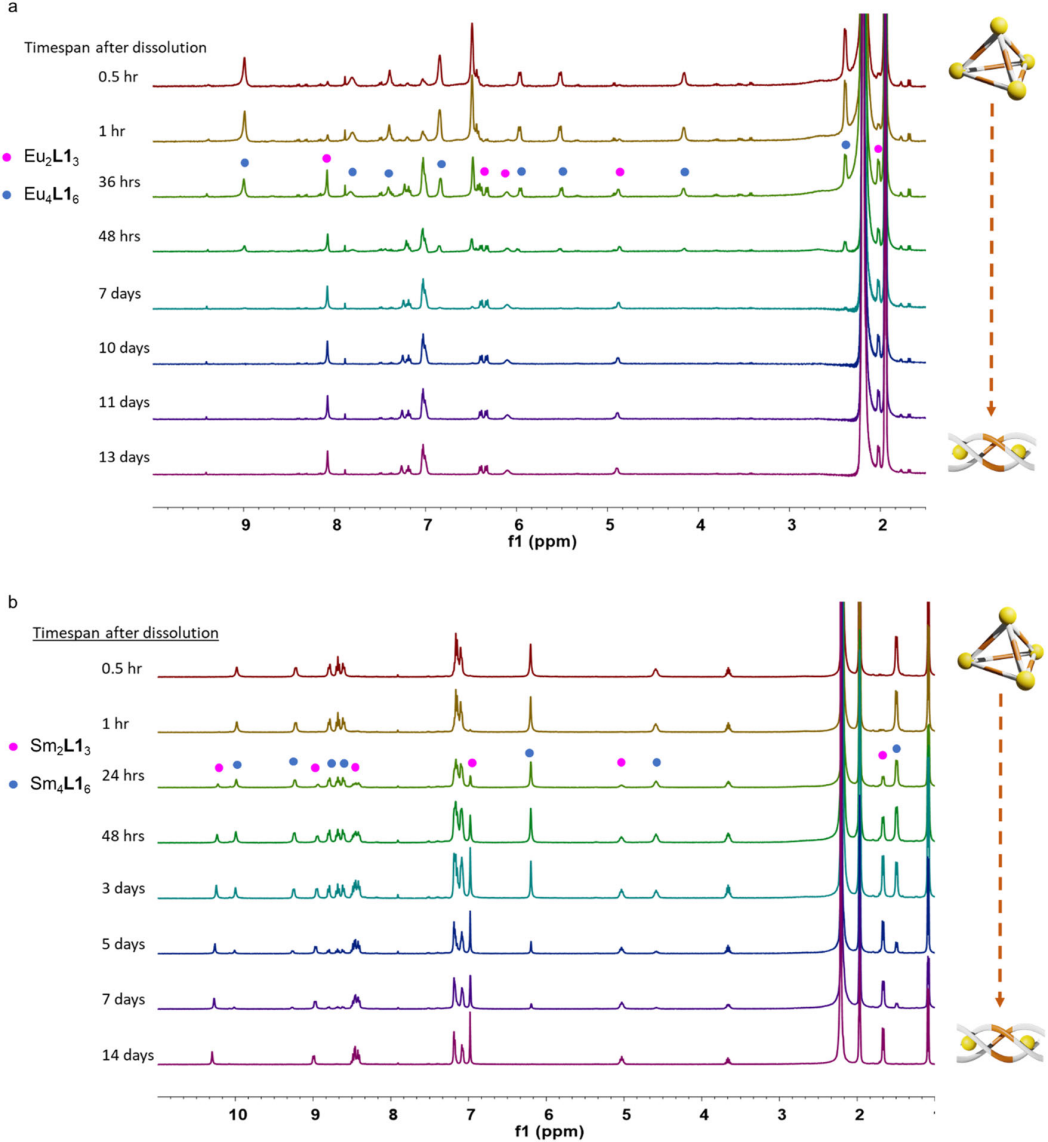
^1^H NMR spectrum of the tetrahedron-to-helicate transformation. **a** Tetrahedron-to-helicate transformation from [Eu_4_**L1**_6_] to [Eu_2_**L1**_3_] (4.53 × 10^−4^ M in CD_3_CN). [Eu_4_**L1**_6_] slowly rearranged back to [Eu_2_**L1**_3_] after dilution in CD_3_CN for 11 days. **b** Tetrahedron-to-helicate transformation from [Sm_4_**L1**_6_] to [Sm_2_**L1**_3_] (4.12 × 10^−4^ M in CD_3_CN) (bottom) upon dissolution in CD_3_CN. [Sm_4_**L1**_6_] slowly rearranged back to [Sm_2_**L1**_3_] after dilution in CD_3_CN for 14 days.

The crystal was then washed with diisopropyl ether and dried under vacuum for further characterization. ^1^H NMR showed a very different resonance compared with that of the mixture of both helicate and tetrahedron. Only a single set of signals was appeared in ^1^H NMR, suggesting only [Eu_4_(**L1**^**SS**^)_6_] presented in the solution (Fig. 4). From the ^1^H NMR result, the integrations are equivalent to the ligand in *C*_2_-symmetric nature, indicating the similar lanthanide geometry with helicate. Comparing with that of helicate, the peaks in tetrahedron NMR are relatively broad. (Supplementary Fig. 15). Since the pseudocontact shifts depends on the distance between the paramagnetic metal ion and proton, it is believed that the proton in tetrahedron locate closer to the europium center. Moreover, the proton in tetrahedron also experienced a stronger paramagnetic shift compared with that of helicate. ESI-HRMS were then performed to characterize the tetrahedron by dissolving the crystal. ESI-HRMS further confirmed the chemical formulas of the isolated tetrahedron. The ESI-HRMS revealed one set of independent peak series, corresponding to the tetranuclear species with the progressive loss of anions and protons. For example, peaks with *m/z* equal to 1065.3668, 1035.3778, 1005.3876 and 975.3884 can be assigned to the species of [{[Eu_4_**L1**^**SS**^_6_](OTf)_m_}-H_n_]^5+^ with *m* = 7, *n* = 0; *m* = 6, *n* = 1; *m* = 5, *n* = 2; *m* = 4, *n* = 3. It was found that the [Eu_4_(**L1**^**SS**^)_6_] is unstable since it rearranged back to [Eu_2_(**L1**^**SS**^)_3_] after dilution in MeCN for 11 days (Fig. 5a).

With this interesting finding, we further investigated whether the helicate-to-tetrahedron transformation will be affected by ionic radii. By replacing the metal source of Eu to Sm, with similar ionic radii, helicate-to-tetrahedron transformation was also observed via ether crystallization. Upon diffusing ether into a solution containing [Sm_2_**L1**_3_], single crystal of [Sm_4_**L1**_6_] was obtained. [Sm_4_**L1**_6_] was then characterized by NMR and ESI-HRMS to confirm the chemical formula. The ^1^H NMR studies also showed a very different signal compared with that of [Sm_2_**L1**_3_]. Similar with the result of [Eu_4_**L1**_6_], [Sm_4_**L1**_6_] is unstable and will slowly transform back to [Sm_2_**L1**_3_] upon dissolution in MeCN (Fig. 5b) Similar helicate-to-tetrahedron transformation can be observed when Eu is replaced by Gd and Tb. Single crystal can be obtained by slow diffusion of ether. However, characterization by NMR is not possible due to the strong paramagnetic nature of Gd and Tb.

Thus, the helicate-to-tetrahedron transformation of Gd and Tb can be only confirmed by ESI-HRMS. ESI-HRMS showed a single set of signal corresponding to the tetrahedron [Gd_4_**L1**_6_] and [Tb_4_**L1**_6_] respectively, confirming the successful helicate to tetrahedron transformation. Although the slow transformation process from tetrahedron to helicate cannot be monitored by ^1^H NMR, the stability of the tetrahedron can be confirmed by dissolving the tetrahedron in MeCN and wait for 14 days before performing ESI-HRMS. The result also showed that [Gd_4_**L1**_6_] and [Tb_4_**L1**_6_] are unstable at low concentration and will rearrange back to [Gd_2_**L1**_3_] and [Tb_2_**L1**_3_] respectively (Supplementary Figs. 16 and 17).

Similar helicate-to-tetrahedron transformation can be observed when Eu is replaced by Lu. Upon diffusing ether into a solution containing [Lu_2_**L1**_3_], single crystal of [Lu_4_**L1**_6_] was obtained and characterized by NMR and ESI-HRMS to confirm the formation of tetranuclear species. The ^1^H NMR also showed a very different signal compared with that of [Lu_2_**L1**_3_]. Interestingly, different from that of [Eu_4_**L1**_6_] and [Sm_4_**L1**_6_], [Lu_4_**L1**_6_] was found to be relatively stable and only insignificant amount of [Lu_2_**L1**_3_] was found upon dissolution in MeCN after 10 days. (Supplementary Figs. 18 and 19).

When Eu was replaced by La, no crystal can be formed when ether is slowly diffused into a solution containing [La_2_**L1**_3_]. The sample was dried and characterized by ^1^HNMR, which showed that the complex remains in stable [La_2_**L1**_3_] helicate form and no other supramolecular complexes can be observed (Supplementary Fig. 20).

Result showed that the length of linker has a great influence on the helicate-to-tetrahedron transformation. In this study, the metal-chelating planes of three ligands are parallel to each other sharing the same *C*_3_ axis. It was expected to form stable M_2_**L**_3_ helicate. Theoretically, longer and more flexible linker may increase the chance of forming unwanted cluster^34^. Therefore, the monophenyl linker in **L1** is relatively shorter compared with that of **L2** and **L3** which is expected to favor the formation of stable helicate without other supramolecular product. However, helicate-to-tetrahedron transformation was observed for **L1** and pure tetrahedron can be isolated via ether crystallization. Even if the offset of two chelating units are the same for the three ligands, no helicate-to-tetrahedron transformation can be observed for **L2** and **L3**. Therefore, results indicates that the distance between two chelating group also governs the supramolecular formation.

**Fig. 6:**
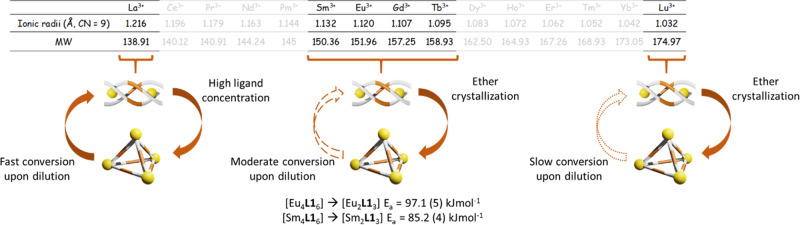
Summary of helicate-to-tetrahedron transformation by different lanthanide ions. Ionic radii showed a great influence on the interconvertion between helicate and tetrahedron. Largest La^3+^ ion prefers to form stable helicate while smallest Lu^3+^ ions can form both stable helicate and relatively stable tetrahedron assemblies. Lanthanide ions that lies between La and Lu were found to form stable helicates and upon dilution tetrahedron-to-helicate transformation was observed. Both [Eu_4_**L1**_6_] and [Sm_4_**L1**_6_] exhibited similar activation energy under the tetrahedron-to-helicate transformation process.

The supramolecular stability of this system also varies across the lanthanide series (Fig. 6). Generally, lanthanide ions are considered as having similar size and thus having similar physical and chemical properties. The effect of ionic radii on self-assembly process was well documented^35,36^. In this study, we found that the result is consistent with the work that reported by Hamacek’s group^37^. The largest La ion highly prefers to form stable helicate and the labile tetrahedron can be only observed under high concentration of ligand. The use of the smallest Lu ions can form both stable helicate and relatively stable tetrahedron at low concentration. The lanthanide ions that lies between La and Lu were found to form stable helicate at low concentration and stable tetrahedron at high concentration. Kinetics studies revealed that both [Eu_4_**L1**_6_] and [Sm_4_**L1**_6_] exhibited similar activation energy (97.1 (5) and 85.2 (4) kJ mol^−1^ repectively) under the tetrahedron-to-helicate transformation process, probably due to the similar size of ionic radii. The positive value of ΔH_298_ also confirmed that such transformation process is endothermic (Supplementary Figs. 21, 22 and Supplementary Tables 1, 2).

Regarding the formation process of the tetrahedron, we hypothesized that the M_2_L_3_ helicate is a crucial intermediate in forming the M_4_L_6_ tetrahedron. Here we propose that the ligands and metal ions will first self-assembled to form the entropically favored helicate, which when subjected to a highly concentrated environment will undergo rearrangement to form the tetrahedral species such as in the crystallization process. The helicate here is expected to act as an important intermediate in the formation of the tetrahedron which forms in a stepwise approach. This hypothesis was further supported by trying to form the Lu tetrahedron in a one pot reaction. Despite testing different reaction conditions, the direct formation of Lu tetrahedron was not possible. All the tetrahedron seems to only form upon crystallization, from a solution of helicate intermediates. It was noted that the rate of tetrahedron formation was dependent on the size of the lanthanide ion.

### Photophysical measurements

Photophysical measurement was performed for the complexes formed with **L1** and **L3**. For **L1**, all the solution measurements of pure [Eu_4_**L1**_6_] tetrahedron were performed within 1 h after dissolution of sample in order to avoid the effect of tetrahedron-to-helicate transformation. The UV–Vis absorption measurement showed that the helicates exhibit different photophysical properties compared with that of tetrahedron. The λ_max_ of [Eu_2_**L1**_3_] was blueshifted for 19 nm compared with that of [Eu_4_**L1**_6_] (Supplementary Fig. 23).

The enantiomeric purities of Eu complexes were confirmed by solution circular dichroism (CD) measurement. Similar with that of UV–Vis absorption, [Ln_2_**L1**_3_] and [Ln_4_**L1**_6_] (Ln = La, Eu, and Sm) also showed differences in the CD absorption. For [Ln_2_**L1**_3_], strong cotton effects were observed at 212, 240, 259, 273, 312, and 381 nm while for [Ln_4_**L1**_6_], strong cotton effects were observed at 212, 240, 259, 273, 312, and 362 nm (Supplementary Figs. 24–34). A mirror image of CD spectra was observed for *R*- and *S*-isomer. For [Ln_2_**L3**_3_] (Ln = La and Eu), strong Cotton effects were observed at 209, 240, 263, 290, 334, and 381 nm and mirror image was also observed for the opposite isomer (Supplementary Figs. 35–40).

The luminescent properties of the complexes were examined. For [Eu_2_**L1**_3_] and [Eu_4_**L1**_6_], successful sensitization of Eu^III 5^D_0_ excited state via antenna effect was observed. Upon excitation of [Eu_2_**L1**_3_] at 311 nm, characteristics narrow Eu red emission lines were observed, occurring at 595, 616, 688, and 697 nm, which corresponds to the transition from first excited state ^5^D_0_ to ^7^F_*J*_ ground state (*J* = 1, 2, and 4). Similar result was observed upon excitation of [Eu_4_**L1**_6_] at 330 nm. The relative quantum yield of [Eu_2_**L1**^**RR**^_3_] was determined to be 4.41% which is higher than that of [Eu_4_**L1**^**RR**^_6_] (0.83%).

**Fig. 7:**
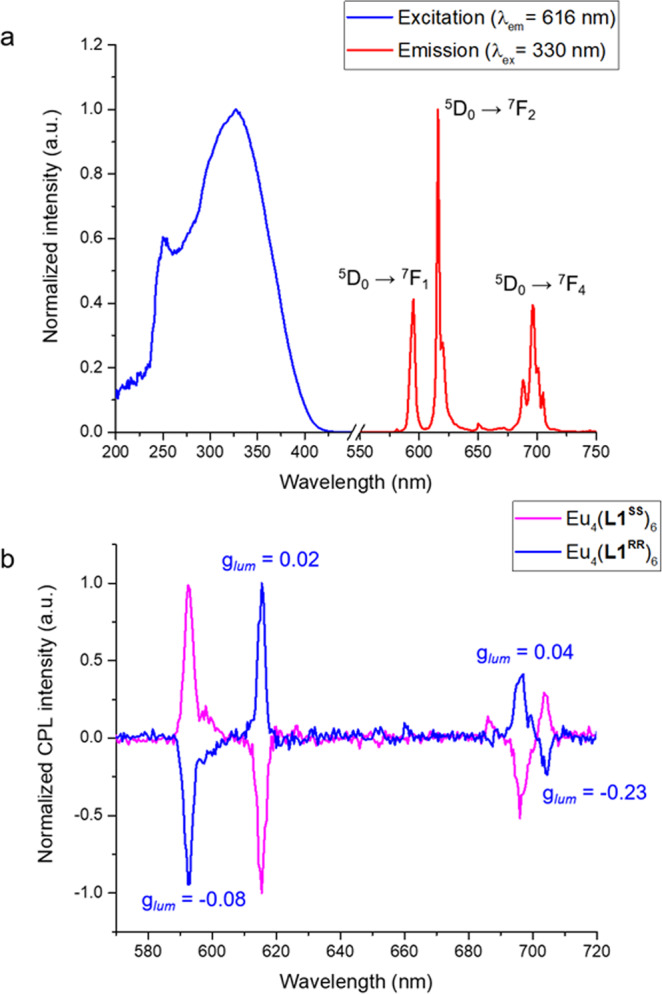
Photophysical data of Eu_4_**L1**^**RR/SS**^_6_. **a** Normalized excitation and emission spectra. Characteristics Eu emission lines were observed which corresponds to the transition from ^5^D_0_ to ^7^F_*J*_ (*J* = 1, 2, and 4). **b** Normalized CPL spectra of Eu_4_**L1**^**RR/SS**^_6_ in MeCN.

The enantiomeric nature of both helicate and tetrahedron complexes were also examined and confirmed by the corresponding circularly polarized luminescence (CPL) spectra. CPL was quantified by the luminescence dysmmetry factor, *g*_lum_, calculated by 2(I_L_ − I_R_)/(I_L_ + I_R_), where I_L_ and I_R_ are the intensity of emitted left and right circularly polarized light respectively. For both [Eu_2_**L1**^**SS**^_3_] and [Eu_4_**L1**^**SS**^_6_], fairly strong CPL was observed for the ^5^D_0_ −> ^7^F_1_ transition, with *g*_lum_ = +0.10 for both complexes. Similarly, for both complexes, CPL emitted from the ^5^D_0_ −> ^7^F_2_ transition was somewhat weaker, *g*_lum_ = −0.02. As expected, the enantiomers of these complexes had mirror CPL spectra (full *g*_lum_ values are provided in Supplementary Table 5). The CD and CPL result were consistent with the diastereoselective and diastereoselective breaking supramolecular formation behavior that reported by Wong and Law et al.^31^. Results showed that the Eu complexes, especially [Eu_4_**L1**_6_], exhibits impressive luminescence and CPL properties (Fig. 7).

## Conclusion

In conclusion, we have demonstrated the effect of ionic radii and length of linker on the lanthanide self-assembly supramolecular formation. Apart from the offset distance between two chelating group in *C*_2_ symmetrical ligand, we found that lanthanide supramolecular formation is also sensitive to the distance between two metal-chelating group. The formation of tetrahedron can also be favored by varying the length of the central bridging linker. In our study we also show that the larger lanthanide ion tends to favor the formation of dimetallic helicates whereas with a decrease in size, the preferential control is lost and the central lanthanides shows a formation of another species which is the tetrahedron. On the other hand, the smallest lanthanide, Lu formed the most stable tetrahedron which was tolerant to dilution with no obvious signs of re-transformation back to the helicate intermediate. This work is important in elucidating the parameters in order to manipulate the design properties to create self assembling supramolecular lanthanide edifices.

## Methods

### General

Unless otherwise stated, all chemicals and solvents were obtained commercially and without further purification before used. All moisture-sensitive compounds were manipulated using standard Schlenk line techniques. All moisture-sensitive reactions were conducted under a nitrogen atmosphere in glasswares that were oven-dried at 140 °C overnight prior to use. Anhydrous dimethylformamide (DMF) and N,N-Diisopropylethylamine (DIPEA) were purchased from Acros. Other solvents were used as received. Grace silica gel 60 (40–63 mesh) was used for column chromatography.1D NMR spectra were recorded on a Bruker Ultrashield Advance Pro 400 MHz instrument and Jeol ECZ500R 500 MHz NMR spectrometer and the chemical shifts were determined with tetramethylsilane (TMS) or solvents in parts per million (ppm). Elemental analyses were performed on an Elementar Vario EL cube elemental analyzer. HRMS were performed on a Waters Synapt G2-Si Quadrupole MS.

### Syntheses

All the new compounds were fully characterized and spectra are given in Supplementary Methods. For NMR spectra see Supplementary Figs. 41–80. For ESI-HRMS spectra see Supplementary Figs. 81–97.

### Photophysical measurement

Single-photon luminescence spectra were recorded using an Edinburgh Instrument FLSP920 spectrophotometer that was equipped with Xe900 continuous xenon lamp, μF920 microsecond flashlamp and a single-photon counting Photomultiplier Tube. The excitation and emission spectra recorded on the FLSP920 were corrected with the correction file from the F900 software. UV–Visible absorption spectra were recorded with an Agilent Technologies Cary 8454 spectrophotometer. CD spectra were recorded with a Jasco J-801 spectropolarimeter with a 1 cm cell at 25 °C. and presented as Δɛ in M^−1^ cm^−1^. CPL spectra were recorded using a custom-build spectrometer consisting of a laser-driven light source (Energetiq EQ–99 LDLS, spectral range 170–2100 nm) coupled to an Acton SP2150 monochromator (600 g/nm, 300 nm Blaze) enabling excitation with a 6 nm FWHM band-pass. Emission was collected at 90° to excitation. The emission pathway consisted of a photo-elastic modulator (PEM) (Hinds Instruments PEM-90) coupled to a lock-in amplifier (Hinds Instruments SignaLoc 2100). The emitted light was then focused onto an Acton SP2150 monochromator (1200 g/nm, 500 nm Blaze) equipped with a high sensitivity cooled Photo Multiplier Tube (H10723– S5 20 Extended red–multialkali PMT based photosensor (5 V)). Spectra were recorded using a 5 spectral average sequence in the range of 570–720 nm with 0.5 nm spectral intervals and 500 μs integration time. Analysis of CPL spectra was conducted in Microsoft Excel, with any instrumental baselines zeroed. Samples were prepared in CH_3_CN to a concentration equivalent to 0.3 absorbance (at 312 nm for [Eu_2_(**L1**^**SS/RR**^)_3_] and 330 nm for [Eu_4_(**L1**^**SS/RR**^)_6_]) as measured by a UV/VIS absorbance spectrometer (UNICAM UV2). Samples were contained in a quartz cuvette with a 10 × 10 mm pathlength (111-10-40, Hellma Analytics) for measurement. For CPL measurement, samples of [Eu_2_(**L1**^**SS/RR**^)_3_] were excited at 312 nm and samples of [Eu_4_(**L1**^**SS/RR**^)_6_] were excited at 331 nm.

### Single crystal X-ray diffractions

Tb_2_(**L2**^**SS**^)_3_ was collected on a Bruker D8-Venture Diffractometer System with a micro-focus Mo-Kα radiation. Of a total of 210003 reflections collected, 31,661 were unique (*R*_int_ = 0.0893). Multi-scan absorption correction was applied by SADABS program^38^, and the SAINT program utilized for the integration of the diffraction profile^39^. The structure was solved by direct method and was refined by a full-matrix least-squares treatment on *F*^*2*^ using the SHELXLE programme system^40^. The final *R*-values were: *R*1 = 0.0524 [I>2 s(I)] and *wR*2 = 0.1159 (all data).

Crystals of the complex Eu_4_**L1**_6_ showed rapid degradation when exposed to atmospheric conditions, this was observed optically and in the diffraction pattern of the sample. Rapid transfer to the laboratories standard inert mounting oil Fomblin YR1800, resulted in a minor increase in the survival time of the sample, but significant damage was observed even over short timeframes. Furthermore, rapid transfer of the block like crystals to a cold stream of nitrogen gas at 150 K caused crystal cracking and a further decrease in time to loss of crystallinity. These results are entirely consistent with rapid volatile solvent loss in structures that contain large open pores and accessible volumes. Crystal mounting in Lindemann capillary tubes was attempted and showed that if solvent loss could be prevented diffraction data could be collected, this was demonstrated through diffraction patterns collected from small fragments of crystals that survived mounting in capillaries at elevated temperatures from the laboratory standard of 150 K, with an optimal data collection temperature appearing to be 220 K. Crystal selection is inherently more difficult when mounting crystals in this way, particularly when the samples are so sensitive to atmospheric changes. Therefore, other mounting oils were investigated with increasing viscosity. Finally, neat Paratone oil was found to allow sufficient working time to select crystals and provide, at least initially well resolved diffraction patterns. The prolonged exposure of the final sample also showed gradual loss of diffracting power indicating the reduction in the long-range order in the structure. Data were collected on a Bruker D8 Venture single crystal diffractometer with a Bruker Photon II detector and an Incoatec IµS 3 Cu micro-focus X-ray source. These data were then processed within the APEX3 software suite and solved and refined using the OLEX2 interface to the SHELX suite of programs. The data processing was truncated to only include the frames from the unit cell data collection along with the first 3 experimental ‘runs’. Data collected after this showed a significant reduction in diffraction intensity at higher angles and would reduce the overall data quality when scaled together, the result of this is a reduction in data completeness. Integrated data were treated for absorption and decay effects within the SADABS program using the linear decay algorithm.

Initial structure solution using the program SHELXT was facile, with early identification of the metal centers and the majority of the linker moieties. Progression of the refinement beyond the location of the full ‘molecule’ and several counter ions proved increasingly limited by the lack of interpretable electron density formed through difference Fourier synthesis. The final structure reported has the inclusion of all triflate ions that could be located via the identification of suitable peaks in the electron density representing the sulfur atoms. The majority of these ions required the manual construction of the moieties and tight restraints to be applied for the earlier stages of refinement, even in the final rounds these ions are not well defined. The remaining triflate ion and solvent molecule contributions were then removed from the diffraction data using the PLATON SQUEEZE algorithm. Improved refinement statistics can be achieved through the removal of all counter ions and solvent from the structure through this method and many combinations of this approach. All refinement results showed conclusive formation of the tetrahedron structure of Eu_4_**L1**_6_. Diffraction data are supplied for both the non SQUEEZed and final reflection files, with the presented structure refined as described.

### Kinetic study

The activation parameters for tetrahedron-to-helicate transformation can be derived from the rate constants at different temperature. Based on the Arrhenius Equation, a plot of ln*k* versus 1/T affords the activation energy E_a_ and frequency factor A.

$$lnk = lnA - \frac{{E_a}}{{RT}}$$

where *k* is the rate constant, A is the frequency factor, E_a_ is the activation energy (Jmol^−1^), R is the gas constant (8.314 JK^−1^mol^−1^), T is the temperature (K).

Enthalpy ΔH can be calculated after obtaining the activation energy by the following equation.

$$E_a = {\Delta}H + RT$$

where E_a_ is the activation energy (Jmol^−1^), R is the gas constant (8.314 JK^−1^ mol^−1^), T is the temperature (K). Kinetic studies were performed by dissolving the Eu/Sm tetrahedron in d-MeCN. The stock tetrahedron solution was then separated evenly and transferred to five NMR tubes. Water bath with four different temperatures (308, 318, 328, 338 K) were prepared. The temperature was maintained by thermostats and monitored by thermometers. NMR tubes were then put into the water bath and NMR was performed to observe the tetrahedron-to-helicate ratio. The concentration of the mixture of tetrahedron and helicate was calculated by the following equations depending on the resolution of NMR spectrum:

2 [Ln_2_**L1**_3_] / 4[Ln_4_**L1**_6_] = integration of linker proton in helicate/integration of linker proton in tetrahedron OR 6 [Ln_2_**L1**_3_]/4[Ln_4_**L1**_6_] = integration of [NH+phenyl proton] in helicate/integration of linker proton in tetrahedron WITH 3 [Ln_2_**L1**_3_] + 6 [Ln_4_**L1**_6_] = [**L1**].

## Supplementary information

Supplementary information

Description of Additional Supplementary Files

Supplementary Data 1

Supplementary Data 2

## Data Availability

The X-ray crystallographic coordinates for structures reported in this article have been deposited at the Cambridge Crystallographic Data Centre (CCDC), under deposition number CCDC No. 1009616 for Tb_2_(**L2**^**SS**^)_3_ (Supplementary Data 1) and CCDC No. 2014348 for Eu_4_(**L1**^**SS**^)_6_ (Supplementary Data 2). These data can be obtained free of charge from The Cambridge Crystallographic Data Centre via www.ccdc.cam.ac.uk/data_request/cif. All other data supporting the finding in this study are available within the article and its Supplementary Information, as well as from the authors upon reasonable request.
