# Supplementary material for: Helicate-to-tetrahedron transformation of chiral lanthanide supramolecular complexes induced by ionic radii effect and linker length
Source: Commun Chem. 2021 Aug 5;4:116. doi: 10.1038/s42004-021-00553-8 (PMC9814731; doi:10.1038/s42004-021-00553-8)
Supplement: Supplementary file 1 — Supplementary information [file 42004_2021_553_MOESM1_ESM.pdf]

Helicate-to-tetrahedron transformation of chiral lanthanide supramolecular complexes induced by ionic radii effect and linker length

King-Him Yim, Chi-Tung Yeung, Michael R. Probert, Wesley Ting Kwok Chan, Lewis E. Macknezie, Robert Pal, Wing-Tak Wong,* and Ga-Lai Law*

Table of Contents

Section	Page number
Experimental procedures	2-10
Data for result and discussion	11-34
NMR characterization	35-49
ESI-HRMS characterization	50-66
Photophysical studies	68-77
X-ray crystallography data	77-83
References	83

Experimental Procedures

(S)-6-((1-phenylethyl)carbamoyl)picolinic acid 1^S and (R)- 6-((1-phenylethyl)carbamoyl)picolinic acid 1^R

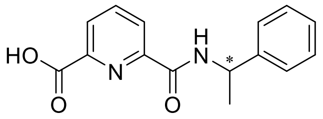

This synthesis is reported by our group previously.[1] To a stirred solution of 2,6-pyridinedicarboxylic acid (5 g, 29.90 mmol, 2.50 equiv.) in anhydrous DMF (70 ml) at room temperature, HATU (4.55 g, 11.90 mmol, 1 equiv.) was added in portions over 10 min under nitrogen. After allowing it to stir for 20 min, (S)-(-)-1-phenylethylamine (1.53 ml, 11.97 mmol, 1 equiv.) was added dropwise and the reaction mixture was allowed to stir for 20 min. DIPEA (5.50 ml, 31.60 mmol, 2.60 equiv.) was then added to the reaction mixture over 5 min and the resulting solution was stirred at room temperature for 14 h. The reaction mixture was then diluted with H_2O (100 ml), and extracted with DCM (30 ml x 3), dried with $MgSO_4$, filtered, and concentrated in vacuo. Then dissolved it in water and extracted with EA to remove excess DMF. The resulting residue was purified by recrystallization in EtOAc solvent to give a white solid. **1^S**: (1.46g, 5.42 mmol, 45.39% yield), mp 110.2 – 117.3 °C. ^{1}H NMR (400 MHz, $CDCl_3$, 298K, δ): 1.65 (d, 7.0Hz, 3H), 5.37 (p, J=7.1Hz, 1H), 7.20-7.44 (m, 5H), 8.01 (d, J=8.2Hz, 1H), 8.11 (t, J=7.8Hz, 1H), 8.36 (dd, J=7.7, 1.2Hz, 1H), 8.48 (dd, J=7.8, 1.2Hz, 1H).^{13}C NMR (400MHz, $CDCl_3$, 300K, δ): 21.50, 49.14, 126.25, 126.72, 126.95, 127.48, 128.67, 139.50, 142.63, 145.37, 149.56, 162.48, 164.67. HRMS (ESI) calcd. for $C_{15}H_{14}N_2O_3Na$ [**1^S**+Na]$^+$: 293.0897, found 293.0895. The enantiomeric purity was determined with HPLC using AS-H column (Hexane/i-propanol: 80/20; flow rate 1.0 ml/min) and compared with a racemic mixture according to the elution orders with the retention times, t_S = 7.15 mins and t_R= 9.62 mins) to be > 99.9%ee. **1^R** was isolated, following the procedure for **1^S** with the use of (R)-(+)-1-phenylethylamine instead, in 55.10% yield (1.78g, 6.59 mmol): mp 110.3 – 113.9 °C. ^{1}H NMR (400 MHz, $CDCl_3$, 298K): δ 1.60 (d, J=6.9Hz, 3H), 5.34 (p, J=7.1Hz, 1H), 7.18-7.41 (m, 5H), 8.06 (t, J=7.8Hz, 1H), 8.29-8.38 (m, 2H), 8.44 (dd, J=7.8, 1.2Hz, 1H). ^{13}C NMR (100 MHz, $CDCl_3$, 300K, δ): 21.50, 30.94, 49.12, 126.23, 126.69, 126.95, 127.44, 128.63, 139.48, 142.63, 145.41, 149.52, 162.58, 164.80. HRMS (ESI) calcd. for $C_{15}H_{14}N_2O_3Na$ [**1^R**+Na]$^+$: 293.0897, found 293.0896. The enantiomeric purity was determined to be 97.0%ee.

N^2,$N^{2'}$-(1,4-phenylene)bis(N^6-((*S*)-1-phenylethyl)pyridine-2,6-dicarboxamide) ($L1^{SS}$) and N^2,$N^{2'}$-(1,4-phenylene)bis(N^6-((*R*)-1-phenylethyl)pyridine-2,6-dicarboxamide) ($L1^{RR}$)

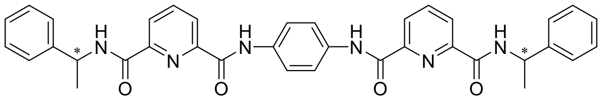

To a stirred solution of **1^R** (0.25 g, 0.92 mmol, 2.2 equiv.) in anhydrous DMF (4 mL) at room temperature, HATU (0.765 g, 2.01 mmol, 4.8 equiv.) was added under nitrogen. After allowing it to stir for 20 min, a 4,4'-diamino-p-terphenyl (0.045 g, 0.417 mmol, 1.0 equiv.) was added and the reaction mixture was allowed to stir for 20 min in dark. DIPEA (0.89 mL, 5.1 mmol, 12.5 equiv.) was then added to the reaction mixture and the resulting solution was stirred at room temperature for 14 h. The reaction mixture was then diluted with H_2O (10 mL) and extracted with DCM (5 × 3 mL). After removing the organic volatile under reduced pressure, the residue was then washed with CH_3CN (10 mL), and fine powder was progressively precipitated out. Then the solid was collected by centrifugation and the desired compound was isolated. **($L1^{RR}$)**: (0.20 g, 0.33 mmol, 70% yield), ^{1}H NMR (400 MHz, $(CD_3)_2SO$, 299 K, δ): 1.67 (d, *J* = 8 Hz, 6H), 5.35–5.45 (m, 2H), 7.24 (t, *J* = 8 Hz, 2H), 7.34 (t, *J* = 8 Hz, 4H), 7.45 (d, *J* = 8 Hz, 4H), 7.70 (s, 4H), 8.07 (t, *J* = 8 Hz, 2H), 8.35 (d, *J* = 8 Hz, 2H), 8.39 (d, *J* = 8 Hz, 2H), 9.27 (d, *J* = 8 Hz, 2H). ^{13}C NMR (100 MHz, $(CD_3)_2SO$, 300 K, δ): 22.17(CH_3), 48.81(CH), 122.36, 125.41, 126.52, 127.28, 128.80, 134.71, 140.20, 144.52, 149.14, 149.42, 162.28, 163.39. HRMS (ESI) calcd. for $C_{72}H_{64}N_{12}O_8Na$ [2**$L1^{RR}$**+Na]$^+$: 1247.4862, found 1247.4850. **($L1^{SS}$)** was synthesized, following the procedure for **($L1^{RR}$)** with the use of **1^S** instead, in 60% yield (0.17 g, 0.28 mmol): ^{1}H NMR (400 MHz, $(CD_3)_2SO$, 299 K, δ): 1.67 (d, *J* = 8 Hz, 6H), 5.35–5.45 (m, 2H), 7.24 (t, *J* = 8 Hz, 2H), 7.34 (t, *J* = 8 Hz, 4H), 7.45 (d, *J* = 8 Hz, 4H), 7.70 (s, 4H), 8.07 (t, *J* = 8 Hz, 2H), 8.35 (d, *J* = 8 Hz, 2H), 8.39 (d, *J* = 8 Hz, 2H), 9.27 (d, *J* = 8 Hz, 2H). ^{13}C NMR (100 MHz, $(CD_3)_2SO$, 300 K, δ): 22.15, 48.78, 122.35, 125.39, 125.58, 126.51, 127.26, 128.78, 134.71, 140.19, 144.51, 149.13, 149.41, 162.28, 163.20. HRMS (ESI) calcd. for $C_{72}H_{64}N_{12}O_8Na$ [2**$L1^{SS}$**+Na]$^+$: 1247.4862, found 1247.4850.

N^2,$N^{2'}$-([1,1':4',1''-terphenyl]-4,4''-diyl)bis(N^6-((R)-1-phenylethyl)pyridine-2,6-dicarboxamide) ($L3^{RR}$) and N^2,$N^{2'}$-([1,1':4',1''-terphenyl]-4,4''-diyl)bis(N^6-((R)-1-phenylethyl)pyridine-2,6-dicarboxamide) ($L3^{RR}$)

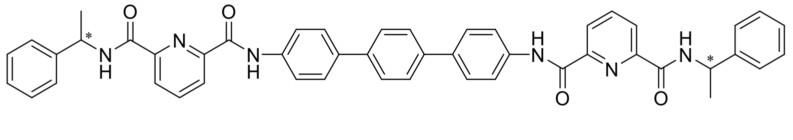

To a stirred solution of **1^R** (0.5 g, 1.85 mmol, 2.2 equiv.) in anhydrous DMF (8 mL) at room temperature, HATU (1.65 g, 4.06 mmol, 4.8 equiv.) was added under nitrogen. After allowing it to stir for 20 min, a 4,4'-diamino-p-terphenyl (1.10 g, 4.21 mmol,

1.0 equiv.) was added and the reaction mixture was allowed to stir for 20 min in dark. DIPEA (1.78 mL, 10.2 mmol, 12.5 equiv.) was then added to the reaction mixture and the resulting solution was stirred at room temperature for 14 h. The reaction mixture was then diluted with H2O (20 mL) and extracted with DCM (5 × 6 mL). After removing all of the organic volatile under reduced pressure, the residue was then washed with CH_3CN three times (20 mL x 3), and fine powder was progressively precipitated out. Then the solid was collected by filtration and the desired compound was isolated. **($L3^{RR}$)**: (0.62 g, 0.81 mmol, 95.28% yield), ^{1}H NMR (400 MHz, $(CD_3)_2SO$, 299 K, δ): 1.69 (d, *J* = 6.96 Hz, 6H), 5.35–5.45 (m, 2H), 7.29 (t, *J* = 2.65 Hz, 2H), 7.39 (t, *J* = 8 Hz, 4H), 7.46 (d, *J* = 8 Hz, 4H), 7.66 (d, *J* = 8 Hz, 8H), 7.78 (d, *J* = 8 Hz, 4H), 8.09 (t, *J* = 8 Hz, 2H), 8.38 (d, *J* = 8 Hz, 2H), 8.44 (d, *J* = 8 Hz, 2H), 8.54 (d, *J* = 8 Hz, 2H), 10.01 (s, 2H). ^{13}C NMR (101 MHz, 300 K, δ): ^{13}C NMR (101 MHz, $(CD_3)_2SO$, 300K, δ): 163.11, 162.22, 149.59, 149.14, 144.60, 140.23, 138.74, 137.85, 135.95, 128.83, 127.29, 126.62, 125.66, 125.40, 122.16, 48.68, 22.28. HRMS (ESI) calcd. for $C_{48}H_{40}N_6O_4Na$ [**$L3^{RR}$**+Na]$^+$: 787.3003, found 787.3038. **($L3^{SS}$)** was synthesized, following the procedure for **($L3^{RR}$)** with the use of **1^S** instead, in 80% yield (0.4 g, 0.52 mmol): ^{1}H NMR (400 MHz, $(CD_3)_2SO$, 299 K, δ): 1.72 (d, *J* = 8 Hz, 6H), 5.40–5.48 (m, 2H), 7.30 (t, *J* = 8 Hz, 2H), 7.41 (t, *J* = 8 Hz, 4H), 7.49 (d, *J* = 8 Hz, 4H), 7.70 (d, *J* = 8 Hz, 8H), 7.83 (d, *J* = 8 Hz, 4H), 8.13 (t, 2H), 8.40 (d, *J* = 8 Hz, 2H), 8.47 (d, *J* = 8 Hz, 2H), 8.77 (d, *J* = 8 Hz, 2H). ^{13}C NMR (100 MHz, $(CD_3)_2SO$, 300 K, δ): 163.11, 162.22, 149.61, 149.15, 144.60, 140.23, 138.75, 137.86, 135.95, 128.83, 127.29, 126.62, 125.65, 125.40, 122.15, 48.67, 22.28. HRMS (ESI) calcd. for $C_{48}H_{40}N_6O_4Na$ [**$L3^{RR}$**+Na]$^+$: 787.3003, found 787.3038.

General synthetic procedures of [Ln_2**L1**$_3$]

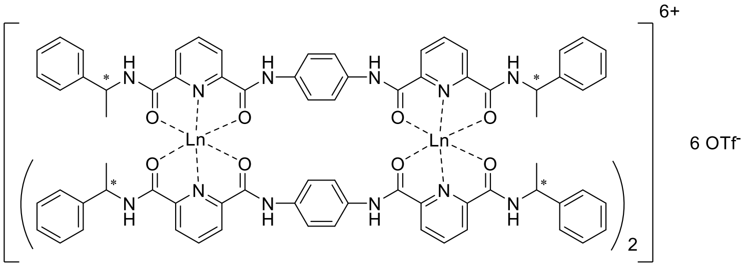

To a white suspension of **L1** (10 mg, 0.016 mmol, 1.5 equiv.) in a mixture of 8.49 ml of DCM/MeOH (12:1, v/v), a solution of $Ln(OTf)_3$ (0.011 mmol, 1 equiv.) (Ln = La, Sm, Eu, Gd, Tb and Lu) in 7.83 ml of CH_3CN was added. The solution was changed to homogeneous colorless solution immediately. The solution was then reacted for 16 hrs at room temperature and pressure. After 16 hrs, the solvent was removed under reduced pressure to give desired product.

[La_2**L1**$^{SS}_3$]: (12.0 mg, 3.99 x 10^{-3} mmol, 73.3% yield), ^1H NMR (400 MHz, CD_3CN, 299 K, δ): δ 10.32 (s, 3 x 2H), 9.00 (d, *J* = 6.9 Hz, 3 x 2H), 8.56 – 8.35 (m, 3 x 6H), 7.24 – 7.01 (m, 3 x 14H), 5.11 (p, *J* = 7.0 Hz, 3 x 2H), 1.71 (d, *J* = 7.1 Hz, 3 x 6H). ^{13}C NMR (100 MHz, CD_3CN, 300 K, δ): 167.57, 166.99, 148.75, 148.51, 143.14, 141.70, 133.52, 128.51, 127.66, 126.60, 126.31, 125.93, 123.56, 52.25, 20.61. HRMS (ESI) calcd. for $C_{112}H_{96}F_{12}La_2N_{18}O_{24}S_4$ [La_2(**L1**^{SS})$_3$ + 4OTf$^-$]$^{2+}$: 1355.6841, found 1355.6792. Elemental analysis calcd. for $C_{114}H_{96}F_{18}La_2N_{18}O_{30}S_6·8H_2O$: C 43.41, H 3.58, N 7.99, found: C 43.35, H 3.60, N 7.92.

[La_2**L1**$^{RR}_3$]: (13.5 mg, 4.48 x 10^{-3} mmol, 82.4% yield), ^1H NMR (400 MHz, CD_3CN, 299 K, δ): 10.34 (s, 3 x 2H), 9.01 (d, *J* = 7.0 Hz, 3 x 2H), 8.61 – 8.39 (m, 3 x 6H), 7.25 – 6.97 (m, 3 x 14H), 5.11 (p, *J* = 7.0 Hz, 3 x 2H), 1.71 (d, *J* = 7.1 Hz, 3 x 6H). ^{13}C NMR (101 MHz, CD_3CN, 300 K, δ): 167.52, 166.92, 148.69, 148.45, 143.05, 141.64, 133.46, 128.44, 127.59, 126.53, 126.25, 125.86, 123.48, 52.21, 20.55. HRMS (ESI) calcd. for $C_{111}H_{95}F_9La_2N_{18}O_{21}S_3$ [La_2(**L1**^{RR})$_3$ + 3OTf$^-$ - H$^+$]$^{2+}$: 1280.7042, found 1280.7103. Elemental analysis calcd. for $C_{114}H_{96}F_{18}La_2N_{18}O_{30}S_6·19H_2O$: C 40.80, H 4.03, N 7.52, found: C 40.90, H 3.57, N 7.30.

[Sm_2**L1**$^{SS}_3$]: (14.5 mg, 4.78 x 10^{-3} mmol, 87.9% yield), ^1H NMR (400 MHz, CD_3CN, 299 K, δ): 10.40 (s, 3 x 2H), 9.11 (d, *J* = 6.8 Hz, 3 x 2H), 8.59 – 8.31 (m, 3 x 6H), 7.18 (dd, *J* = 5.2, 1.9 Hz, 3 x 6H), 7.07 (dd, *J* = 6.8, 2.8 Hz, 3 x 4H), 6.98 (s, 3 x 4H), 5.08 – 4.92 (m, 3 x 2H), 1.66 (d, *J* = 7.0 Hz, 3 x 6H). ^{13}C NMR (125 MHz, CD_3CN, 300 K, δ): 169.49, 168.71, 149.49, 149.22, 143.59, 141.77, 133.34, 128.61, 127.66, 125.87, 125.66, 125.32, 123.32, 52.29, 20.63. HRMS (ESI) calcd. for $C_{111}H_{96}F_9N_{18}O_{21}S_3Sm_2$ [Sm_2(**L1**^{SS})$_3$ + 3OTf$^-$]$^{3+}$: 861.8131, found 861.8192. Elemental analysis calcd. for $C_{114}H_{96}F_{18}Sm_2N_{18}O_{30}S_6·18H_2O$: C 40.78, H 3.96, N 7.51, found: C 40.80, H 3.53, N 7.42.

[Sm_2**L1**$^{RR}_3$]: (12.1 mg, 3.99 x 10^{-3} mmol, 73.3% yield), ^1H NMR (495 MHz, CD_3CN, 299 K, δ): 10.15 (s, 3 x 2H), 8.89 (s, 3 x 2H), 8.45 – 8.32 (m, 3 x 6H), 7.13 (s, 3 x 6H), 7.03 (s, 3 x 4H), 6.92 (s, 3 x 4H), 5.01 – 4.95 (m, 3 x 2H), 1.72 – 1.50 (m, 3 x 6H). ^{13}C NMR (125 MHz, CD_3CN, 300 K, δ): 169.48, 168.71, 149.49, 149.23, 143.59, 141.76, 133.34, 128.61, 127.67, 125.87, 125.66, 125.31, 123.32, 52.28, 20.63. HRMS (ESI) calcd. for $C_{111}H_{96}F_9N_{18}O_{21}S_3Sm_2$ [Sm_2(**L1**^{RR})$_3$ + 3OTf$^-$]$^{3+}$: 861.8131, found 861.8160. Elemental analysis calcd. for $C_{114}H_{96}F_{18}Sm_2N_{18}O_{30}S_6·18H_2O$: C 40.78, H 3.96, N 7.51, found: C 40.80, H 3.36, N 7.35.

[Eu_2**L1**$^{SS}_3$]: (14.1 mg, 4.64 x 10^{-3} mmol, 85.4% yield), ^1H NMR (400 MHz, CD_3CN, 299 K, δ): 8.12 (s, 3 x 4H), 7.40 (s, 3 x 2H), 7.20 (t, *J* = 7.9 Hz, 3 x 2H), 7.05 (d, *J* = 3.9 Hz, 3 x 10H), 6.45 (d, *J* = 8.0 Hz, 3 x 2H), 6.36 (d, *J* = 7.9 Hz, 3 x 2H), 6.14 (s, 3 x 2H), 5.04 (s, 3 x 2H), 2.04 (d, *J* = 6.0 Hz, 3 x 6H). ^{13}C NMR (125 MHz, CD_3CN, 300 K, δ): 160.24, 155.37, 142.84, 142.47, 141.94, 134.83, 128.50, 127.50, 125.65, 125.29, 91.92, 91.80, 51.70, 21.73. HRMS (ESI) calcd. for $C_{112}H_{96}Eu_2F_{12}N_{18}O_{24}S_4$ [Eu_2(**L1**^{SS})$_3$ + 4OTf$^-$]$^{2+}$: 1369.1985, found 1369.1962. Elemental analysis calcd. for $C_{114}H_{96}F_{18}Eu_2N_{18}O_{30}S_6·18H_2O$: C 40.74, H 3.96, N 7.50, found: C 40.83, H 3.56, N 7.44.

[Eu_2**L1**$^{RR}_3$]: (13.6 mg, 4.48 x 10^{-3} mmol, 82.3% yield), ^1H NMR (400 MHz, CD_3CN, 299 K, δ): 8.07 (s, 3 x 4H), 7.13 (d, *J* = 15.9 Hz, 3 x 4H), 6.99 (s, 3 x 10H), 6.37 (s, 3 x 2H), 6.28 (d, *J* = 8.2 Hz, 3 x 2H), 6.09 (s, 3 x 2H), 4.80 (s, 3 x 2H), 1.99 (s, 3 x 6H). ^{13}C NMR (125 MHz, CD_3CN, 300 K, δ): 163.43, 160.28, 155.37, 142.83, 142.46, 142.02, 134.81, 128.50, 127.50, 125.66, 125.29, 91.94, 91.80, 51.70, 21.73. HRMS (ESI) calcd. for $C_{112}H_{96}Eu_2F_{12}N_{18}O_{24}S_4$ [Eu_2(**L1**^{RR})$_3$ + 4OTf$^-$]$^{2+}$: 1369.1985, found 1369.1949. Elemental analysis calcd. for $C_{114}H_{96}F_{18}Eu_2N_{18}O_{30}S_6·4H_2O$: C 44.05, H 3.37, N 8.11, found: C 44.10, H 3.38, N 7.99.

[Gd_2**L1**$^{SS}_3$]: (12.5 mg, 4.10 x 10^{-3} mmol, 75.4% yield), HRMS (ESI) calcd. for $C_{111}H_{96}F_9N_{18}O_{21}S_3Gd_2$ [Gd_2(**L1**^{SS})$_3$ + 3OTf$^-$]$^{3+}$: 866.4836, found 861.4828. Elemental analysis calcd. for $C_{114}H_{96}F_{18}Gd_2N_{18}O_{30}S_6·21H_2O$: C 39.98, H 4.06, N 7.36, found: C 40.01, H 3.54, N 7.30.

[Gd_2**L1**$^{RR}_3$]: (14.3 mg, 4.69 x 10^{-3} mmol, 86.2% yield), HRMS (ESI) calcd. for $C_{111}H_{96}F_9N_{18}O_{21}S_3Gd_2$ [Gd_2(**L1**^{RR})$_3$ + 3OTf$^-$]$^{3+}$: 866.4836, found 861.4865. Elemental analysis calcd. for $C_{114}H_{96}F_{18}Gd_2N_{18}O_{30}S_6·22H_2O$: C 39.77, H 4.10, N 7.32, found: C 39.88, H 3.30, N 7.20.

[Tb_2**L1**$^{SS}{}_3$]: (14.7 g, 4.82 x 10^{-3} mmol, 88.6% yield), HRMS (ESI) calcd. for $C_{109}H_{94}F_3N_{18}O_{15}STb_2$ [Tb_2(**L1**^{SS})$_3$ + OTf^- - $2H^+$]$^{3+}$: 767.5113, found 767.5154. Elemental analysis calcd. for $C_{114}H_{96}F_{18}Tb_2N_{18}O_{30}S_6{\cdot}16H_2O$: C 41.01, H 3.86, N 7.55, found: C 41.07, H 3.49, N 7.48.

[Tb_2**L1**$^{RR}{}_3$]: (13.6 g, 4.46 x 10^{-3} mmol, 81.9% yield), HRMS (ESI) calcd. for $C_{112}H_{96}Eu_2F_{12}N_{18}O_{24}S_4$ [Tb_2(**L1**^{RR})$_3$ + $4OTf^-$]$^{2+}$: 1300.7232, found 1300.7169. Elemental analysis calcd. for $C_{114}H_{96}F_{18}Tb_2N_{18}O_{30}S_6{\cdot}15H_2O$: C 41.24, H 3.82, N 7.59, found: C 41.36, H 3.33, N 7.48.

[Lu_2**L1**$^{SS}{}_3$]: (14.2 g, 4.61 x 10^{-3} mmol, 84.7% yield), ^{1}H NMR (400 MHz, CD_3CN, 299 K, δ): 10.42 (s, 3 x 2H), 9.02 (d, *J* = 6.8 Hz, 3 x 2H), 8.56 – 8.33 (m, 3 x 6H), 7.17 (ddd, *J* = 8.9, 5.5, 3.3 Hz, 3 x 6H), 7.02 (d, *J* = 5.0 Hz, 3 x 8H), 4.97 (p, *J* = 6.9 Hz, 3 x 2H), 1.75 – 1.58 (m, 3 x 6H). ^{13}C NMR (125 MHz, CD_3CN, 300 K, δ): 166.82, 166.38, 147.04, 146.66, 143.08, 141.74, 133.26, 128.66, 127.61, 126.31, 125.79, 125.69, 123.07, 52.29, 20.77. HRMS (ESI) calcd. for $C_{111}H_{96}F_9Lu_2N_{18}O_{21}S_3$ [Lu_2(**L1**^{SS})$_3$ + $3OTf^-$]$^{3+}$: 878.1615, found 878.1616. Elemental analysis calcd. for $C_{114}H_{96}F_{18}Lu_2N_{18}O_{30}S_6{\cdot}7H_2O$: C 42.68, H 3.46, N 7.86, found: C 42.66, H 3.39, N 7.77.

[Lu_2**L1**$^{RR}{}_3$]: (14.4 g, 4.67 x 10^{-3} mmol, 85.9% yield), ^{1}H NMR (400 MHz, CD_3CN, 299 K, δ): 10.31 (s, 3 x 2H), 8.93 (s, 3 x 2H), 8.38 (s, 3 x 6H), 7.13 (s, 3 x 6H), 6.97 (s, 3 x 8H), 4.92 (d, *J* = 7.5 Hz, 3 x 2H), 1.61 (d, *J* = 7.1 Hz, 3 x 6H). ^{13}C NMR (125 MHz, CD_3CN, 300 K, δ): 166.41, 165.98, 146.63, 146.33, 142.69, 141.32, 128.26, 127.21, 125.87, 125.43, 125.29, 122.64, 51.86, 20.36. HRMS (ESI) calcd. for $C_{112}H_{96}F_{12}Lu_2N_{18}O_{24}S_4$ [Lu_2(**L1**^{RR})$_3$ + $4OTf^-$]$^{2+}$: 1391.7185, found 1391.7209. Elemental analysis calcd. for $C_{114}H_{96}F_{18}Lu_2N_{18}O_{30}S_6{\cdot}7H_2O$: C 42.68, H 3.46, N 7.86, found: C 42.74, H 3.54, N 7.84.

General synthetic procedures of [Ln_4**L1**$_6$]

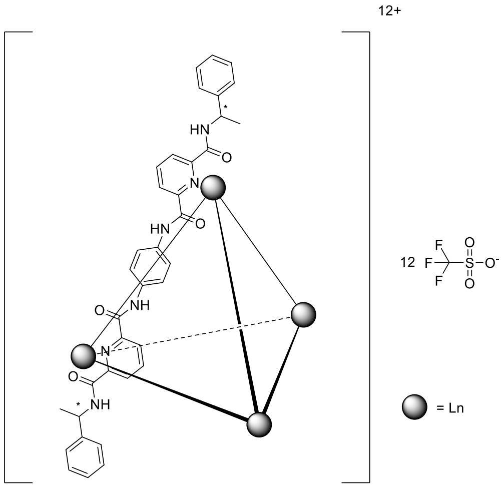

Ln_2**L1**$_3$ (0.66 mmol) were dissolved in CH_3CN (0.6 ml) and ether was allowed to slowly diffuse into the solution. The solution was decanted, and the crystal was washed with ether and dried under vacuum to obtain the homometallic tetrahedron crystal.

[Eu_4**L1**$^{SS}_6$]: (1.80 mg, 2.96 x 10^{-4} mmol, 90.0% yield), ^{1}H NMR (495 MHz, CD_3CN, 299 K, δ): 9.04 (s, 6 x 4H), 7.81 (s, 6 x 2H), 7.31 (s, 6 x 2H), 6.79 (s, 6 x 4H), 6.40 (d, *J* = 31.8 Hz, 6 x 8H), 5.88 (s, 6 x 2H), 5.47 (s, 6 x 2H), 4.09 (s, 6 x 2H), 2.34 (s, 6 x 6H). ^{13}C NMR (125 MHz, CD_3CN, 300 K, δ): 161.73, 156.50, 153.58, 142.91, 136.72, 135.57, 133.80, 127.79, 126.76, 125.52, 124.96, 121.93, 119.36, 92.00, 91.32, 52.29, 22.27. HRMS (ESI) calcd. for $C_{225}H_{192}Eu_4F_{27}N_{36}O_{51}S_9$ [Eu_4(**L1**^{SS})$_6$ + 9 OTf$^-$]$^{3+}$: 1875.2490, found 1875.2516. Elemental analysis calcd. for $C_{228}H_{192}Eu_4F_{36}N_{36}O_{60}S_{12}\cdot 11H_2O\cdot C_6H_{14}O$: C 44.10, H 3.61, N 7.91, found: C 44.12, H 3.68, N 7.83.

[Eu_4**L1**$^{RR}_6$]: (1.68 mg, 2.77 x 10^{-4} mmol, 83.8% yield), ^{1}H NMR (495 MHz, CD_3CN, 299 K, δ) 9.03 (s, 6 x 4H), 7.80 (s, 6 x 2H), 7.33 (s, 6 x 2H), 6.79 (s, 6 x 4H), 6.40 (d, *J* = 30.9 Hz, 6 x 8H), 5.89 (s, 6 x 2H), 5.47 (s, 6 x 2H), 4.11 (s, 6 x 2H), 2.33 (s, 6 x 6H). ^{13}C NMR (125 MHz, CD_3CN, 300 K, δ): 161.81, 156.51, 153.57, 142.91, 136.77, 135.56, 133.85, 128.50, 127.79, 126.76, 125.52, 124.96, 121.96, 119.39, 92.10, 91.38, 52.29, 22.26. HRMS (ESI) calcd. for $C_{225}H_{192}Eu_4F_{27}N_{36}O_{51}S_9$ [Eu_4(**L1**^{RR})$_6$ + 9 OTf$^-$]$^{3+}$: 1875.2490, found 1875.2516. Elemental analysis calcd. for $C_{228}H_{192}Eu_4F_{36}N_{36}O_{60}S_{12}\cdot 14H_2O\cdot 2(C_6H_{14}O)$: C 44.15, H 3.83, N 7.72, found: C 44.13, H 3.78, N 7.72.

[Sm_4**L1**$^{SS}_6$]: (1.74 mg, 2.87 x 10^{-4} mmol, 86.9% yield), ^{1}H NMR (495 MHz, CD_3CN, 299 K, δ) 9.91 (s, 6 x 2H), 9.20 (s, 6 x 2H), 8.76 (d, *J* = 9.6 Hz, 6 x 2H), 8.63 (s, 6 x 2H), 8.54 (d, *J* = 8.6 Hz, 6 x 2H), 7.19 – 6.98 (m, 6 x 10H), 6.14 (s, 6 x 4H), 4.52 (d, *J* = 8.7 Hz, 6 x 2H), 1.43 (s, 6 x 6H). ^{13}C NMR (125 MHz, CD_3CN, 300 K, δ): 170.25, 167.32, 150.56, 148.82, 143.61, 142.40, 132.68, 128.54, 127.64, 126.67, 125.68, 125.39, 122.20, 121.60, 119.64, 52.47, 20.73. HRMS (ESI) calcd. for $C_{222}H_{191}F_{18}N_{36}O_{42}S_6Sm_4$ [Sm_4(**L1**^{SS})$_6$ + 6 OTf$^-$ - H$^+$]$^{5+}$: 1034.1745, found 1034.1748. Elemental analysis calcd. for $C_{228}H_{192}Sm_4F_{36}N_{36}O_{60}S_{12}\cdot 15H_2O\cdot 2(C_6H_{14}O)$: C 44.07, H 3.85, N 7.71, found: C 43.91, H 3.51, N 7.76.

[Sm_4**L1**$^{RR}_6$]: (1.89 g, 3.12 x 10^{-4} mmol, 94.4% yield), ^{1}H NMR (495 MHz, CD_3CN, 299 K, δ) ^{1}H NMR (495 MHz, Acetonitrile-*d*$_3$) δ 9.93 (s, 6 x 2H), 9.22 (s, 6 x 2H), 8.76 (d, *J* = 8.9 Hz, 6 x 2H), 8.74 – 8.59 (m, 6 x 2H), 8.54 (d, *J* = 9.3 Hz, 6 x 2H), 7.07 (dd, *J* = 29.5, 9.9 Hz, 6 x 10H), 6.13 (s, 6 x 4H), 4.52 (s, 6 x 2H), 1.44 (s, 6 x 6H). ^{13}C NMR (125 MHz, CD_3CN, 300 K, δ): 170.26, 167.34, 150.55, 148.83, 143.61, 142.42, 132.68, 128.54, 127.64, 126.67, 125.86, 125.67, 125.41, 122.21, 121.62, 119.64, 52.47, 20.73. HRMS (ESI) calcd. for $C_{225}H_{192}Sm_4F_{27}N_{36}O_{51}S_9$ [Sm_4(**L1**^{RR})$_6$ + 9 OTf$^-$]$^{3+}$: 1873.2460, found 1873.2405. Elemental analysis calcd. for $C_{228}H_{192}Sm_4F_{36}N_{36}O_{60}S_{12}\cdot 12H_2O\cdot C_6H_{14}O$: C 44.02, H 3.63, N 7.90, found: C 44.03, H 3.72, N 7.92.

[Gd_4**L1**$^{SS}_6$]: (1.45 mg, 2.38 x 10^{-4} mmol, 72.1% yield), HRMS (ESI) calcd for $C_{224}H_{192}F_{24}Gd_4N_{36}O_{48}S_8$ [Gd_4(**L1**^{SS})$_6$ + 8 OTf$^-$]$^{4+}$ is 1374.4520, found 1374.4512. Elemental analysis calcd. for $C_{228}H_{192}Gd_4F_{36}N_{36}O_{60}S_{12}\cdot 12H_2O$: C 43.40, H 3.45, N 7.99, found: C 43.31, H 3.38, N 7.78.

[Gd_4**L1**$^{RR}_6$]: (1.23 mg, 2.02 x 10^{-4} mmol, 61.2% yield), HRMS (ESI) calcd for $C_{225}H_{192}F_{27}Gd_4N_{36}O_{51}S_9$ [Gd_4(**L1**^{RR})$_6$ + 9 OTf$^-$]$^{4+}$ is 1882.2535 (100%), found 1882.2499. Elemental analysis calcd. for $C_{228}H_{192}Gd_4F_{36}N_{36}O_{60}S_{12}\cdot 13H_2O$: C 43.28, H 3.47, N 7.97, found: C 43.22, H 3.58, N 7.85.

[Tb_4**L1**$^{SS}_6$]: (1.37 mg, 2.25 x 10^{-4} mmol, 68.1% yield), HRMS (ESI) calcd for $C_{222}H_{191}F_{18}N_{36}O_{42}S_6Tb_4$ [Tb_4(**L1**^{SS})$_6$ + 6 OTf$^-$ - H$^+$]$^{5+}$ is 1040.9802, found 1040.9800. Elemental analysis calcd. for $C_{228}H_{192}Tb_4F_{36}N_{36}O_{60}S_{12}\cdot 12H_2O\cdot C_6H_{14}O$: C 43.79, H 3.61, N 7.86, found: C 43.74, H 3.68, N 7.81.

[Tb_4**L1**$^{RR}{}_6$]: (1.66 mg, 2.72 x 10^{-4} mmol, 82.5% yield), HRMS (ESI) calcd for $C_{222}H_{191}F_{18}N_{36}O_{42}S_6Tb_4$ [Tb_4(**L1**^{SS})$_6$ + 6 OTf^- - H^+]$^{5+}$ is 1040.9802, found 1040.9839. Elemental analysis calcd. for $C_{228}H_{192}Tb_4F_{36}N_{36}O_{60}S_{12}{\cdot}17H_2O{\cdot}C_6H_{14}O$: C 43.18, H 3.72, N 7.75, found: C 43.13, H 3.46, N 7.67.

[Lu_4**L1**$^{SS}{}_6$]: (1.89 mg, 3.07 x 10^{-4} mmol, 92.9% yield), ^1H NMR (400 MHz, CD_3CN, 299 K, δ) 10.20 (s, 6 x 2H), 9.07 (s, 6 x 2H), 8.46 (dd, *J* = 10.6, 5.7 Hz, 6 x 6H), 7.05 (dt, *J* = 21.9, 6.3 Hz, 6 x 10H), 6.71 (d, *J* = 4.9 Hz, 6 x 4H), 5.02 (q, *J* = 6.5 Hz, 6 x 2H), 1.65 (t, *J* = 5.9 Hz, 6 x 6H). HRMS (ESI) calcd. for $C_{225}H_{192}Lu_4F_{27}N_{36}O_{51}S_9$ [Lu_4(**L1**^{SS})$_6$ + 9 OTf^-]$^{3+}$: 1905.6090, found 1905.6060.

[Lu_4**L1**$^{RR}{}_6$]: (1.34 mg, 2.17 x 10^{-4} mmol, 65.9% yield), ^1H NMR (405 MHz, CD_3CN, 299 K, δ) 10.22 (s, 6 x 2H), 9.07 (s, 6 x 2H), 8.47 (d, *J* = 5.5 Hz, 6 x 6H), 7.21 – 6.91 (m, 6 x 10H), 6.72 (s, 6 x 4H), 5.02 (p, *J* = 7.0 Hz, 6 x 2H), 1.66 (d, *J* = 7.1 Hz, 6 x 6H). HRMS (ESI) calcd. for $C_{225}H_{192}Lu_4F_{27}N_{36}O_{51}S_9$ [Lu_4(**L1**^{RR})$_6$ + 9 OTf^-]$^{3+}$: 1905.6090, found 1905.6080.

General synthetic procedures of [Ln_2**L3**$_3$]

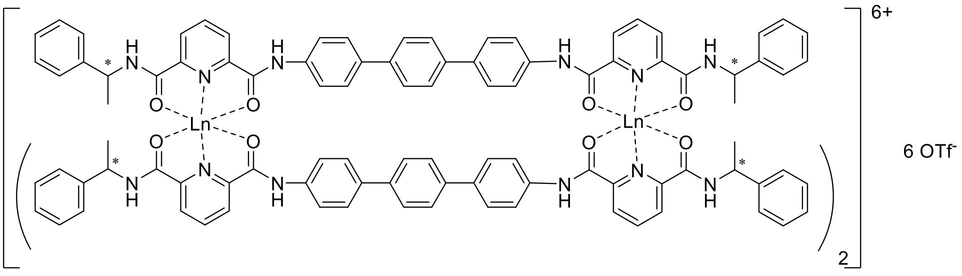

To a white suspension of **L3** (30 mg, 0.039 mmol, 1.5 equiv.) in a mixture of 2.55 ml of DCM/MeOH (12:1, v/v), a solution of $Ln(OTf)_3$ (0.026 mmol, 1 equiv.) (Ln = La, Sm, Eu, Gd, Tb and Lu) in 2.35 ml of CH_3CN was added. The solution was changed to yellow immediately. The solution was then reacted for 16 hrs at room temperature and pressure. After 16 hrs, the solvent was removed under reduced pressure to give desired product.

[La_2**L3**$^{SS}{}_3$]: (39.4 mg, 0.114 mmol, 86.9% yield), ^{1}H NMR (400 MHz, CD_3CN, 299 K, δ): 10.21 (s, 3 x 2H), 9.07 (d, J = 7.1 Hz, 3 x 2H), 8.67 – 8.40 (m, 3 x 6H), 7.88 (d, J = 8.3 Hz, 3 x 4H), 7.31 – 7.20 (m, 3 x 6H), 7.14 (s, 3 x 4H), 7.02 (d, J = 8.3 Hz, 3 x 4H), 6.95 (s, 3 x 4H), 5.14 – 5.05 (m, 3 x 2H), 1.76 (d, J = 7.1 Hz, 3 x 6H). ^{13}C NMR (101 MHz, CD_3CN, 300 K, δ): 168.78, 168.17, 150.80, 150.04, 144.52, 142.98, 139.58, 130.07, 129.10, 128.65, 128.40, 127.82, 127.37, 123.02, 119.33, 118.78, 55.51, 22.11. HRMS (ESI) calcd. for $C_{147}H_{120}F_9La_2N_{18}O_{21}S_3$ [La_2(**L3^{SS}**)$_3$ + 3 OTf$^-$]$^{3+}$: 1006.2012, found 1006.2037. Elemental analysis calcd. for $C_{150}H_{120}F_{18}N_{18}O_{30}S_6La_2 \cdot 7H_2O$: C 50.14, H 3.76, N 7.02, found: C 50.24, H 4.21, N 6.71.

[La_2**L3**$^{RR}{}_3$]: (37.9 mg, 0.109 mmol, 83.7% yield), ^{1}H NMR (400 MHz, CD_3CN, 299 K, δ): 10.21 (s, 3 x 2H), 9.07 (d, J = 6.6 Hz, 3 x 2H), 8.54 (d, J = 21.5 Hz, 3 x 6H), 7.88 (d, J = 8.3 Hz, 3 x 4H), 7.25 (s, 3 x 6H), 7.13 (s, 3 x 4H), 7.02 (d, J = 8.2 Hz, 3 x 4H), 6.96 (s, 3 x 4H), 5.08 (s, 3 x 2H), 1.76 (d, J = 7.1 Hz, 3 x 6H). ^{13}C NMR (125 MHz, CD_3CN, 300 K, δ): 167.40, 166.79, 149.42, 148.66, 143.13, 141.62, 138.50, 138.19, 135.54, 128.70, 127.72, 127.27, 127.02, 126.39, 125.98, 121.64, 52.14, 22.24. HRMS (ESI) calcd. for $C_{147}H_{120}F_9La_2N_{18}O_{21}S_3$ [La_2(**L3^{RR}**)$_3$ + 3 OTf$^-$]$^{3+}$: 1006.2012, found 1006.2037. Elemental analysis calcd. for $C_{150}H_{120}F_{18}N_{18}O_{30}S_6La_2 \cdot 6H_2O$: C 50.40, H 3.72, N 7.05, found: C 50.42, H 3.95, N 6.90.

[Sm_2**L3**$^{SS}{}_3$]: (38.1 mg, 0.0109 mmol, 83.5% yield), ^{1}H NMR (400 MHz, CD_3CN, 299 K, δ): 10.15 (s, 3 x 2H), 9.03 (d, J = 7.0 Hz, 3 x 2H), 8.44 (dq, J = 7.3, 4.3, 3.5 Hz, 3 x 6H), 7.89 (d, J = 8.3 Hz, 3 x 4H), 7.35 – 7.16 (m, 3 x 6H), 7.10 (dd, J = 7.0, 2.5 Hz, 3 x 4H), 6.98 (d, J = 8.2 Hz, 3 x 4H), 6.91 (s, 3 x 4H), 5.19 (q, J = 7.0 Hz, 3 x 2H), 1.77 (d, J = 6.9 Hz, 3 x 6H). ^{13}C NMR (101 MHz, CD_3CN, 300K, δ): 169.41, 168.43, 149.63, 148.99, 143.29, 141.71, 138.44, 137.96, 135.57, 128.69, 127.62, 127.20, 126.80, 125.81, 125.20, 121.36, 52.31, 20.90. HRMS (ESI) calcd. for $C_{147}H_{120}F_9Sm_2N_{18}O_{21}S_3$ [Sm_2(**L3^{SS}**)$_3$ + 3 OTf$^-$]$^{3+}$: 1013.8759, found 1013.8749. Elemental analysis calcd. for $C_{150}H_{120}F_{18}N_{18}O_{30}S_6Sm_2 \cdot 8H_2O$: C 49.58, H 3.77, N 6.94, found: C 49.59, H 4.07, N 6.81.

[Sm_2**L3**$^{RR}{}_3$]: (36.5 mg, 0.0104 mmol, 79.9% yield), ^{1}H NMR (400 MHz, CD_3CN, 299 K, δ): 10.11 (s, 3 x 2H), 9.00 (d, J = 7.0 Hz, 3 x 2H), 8.43 (dq, J = 7.3, 4.3, 3.5 Hz, 3 x 6H), 7.89 (d, J = 8.3 Hz, 3 x 4H), 7.42 – 7.17 (m, 3 x 6H), 7.10 (dd, J = 7.0, 2.5 Hz, 3 x 4H), 6.98 (d, J = 8.2 Hz, 3 x 4H), 6.91 (s, 3 x 4H), 5.19 (q, J = 7.0 Hz, 3 x 2H), 1.78 (d, J = 6.9 Hz, 3 x 6H). ^{13}C NMR (101 MHz, CD_3CN, 300K, δ): 169.42, 168.46, 149.74, 149.03, 143.40, 141.67, 138.49, 138.02, 135.53, 128.68, 127.62, 127.22, 126.87, 125.80, 125.25, 121.32, 52.25, 20.81. HRMS (ESI) calcd. for $C_{144}H_{118}N_{18}O_{12}Sm_2$ [Sm_2(**L3^{RR}**)$_3$ – 3H$^+$]$^{3+}$: 863.9162, found 863.9186. Elemental analysis calcd. for $C_{150}H_{120}F_{18}N_{18}O_{30}S_6Sm_2 \cdot 17H_2O$: C 47.46, H 4.09, N 6.64, found: C 47.34, H 3.96, N 6.48.

[Eu_2**L3**$^{SS}{}_3$]: (36.4 mg, 0.0104 mmol, 79.8 % yield), ^{1}H NMR (400 MHz, CD_3CN, 299 K, δ): 8.21 – 7.96 (m, 3 x 4H), 7.42 (s, 3 x 2H), 7.24 (s, 3 x 4H), 7.15 (d, J = 5.8 Hz, 3 x 6H), 7.08 (s, 3 x 4H), 6.96 (s, 3 x 4H), 6.76 (s, 3 x 2H), 6.58 (s, 3 x 4H), 5.36 (s, 3 x 2H), 4.99 (s, 3 x 2H), 1.76 (s, 3 x 6H). ^{13}C NMR (125 MHz, CD_3CN, 300 K, δ): 155.25, 142.22, 138.62, 137.71, 135.36, 128.25, 127.19, 126.81, 126.69, 125.21, 122.15, 92.13, 91.52, 50.88, 21.13. HRMS (ESI) calcd. for $C_{147}H_{120}Eu_2F_9N_{18}O_{21}S_3$ [Eu_2(**L3^{SS}**)$_3$ + 3OTf$^-$]$^{3+}$: 1015.2111, found 1015.2097. Elemental analysis calcd. for $C_{150}H_{120}F_{18}N_{18}O_{30}S_6Eu_2 \cdot 4H_2O$: C 50.54, H 3.62, N 7.07, found: C 50.55, H 3.90, N 6.50.

[Eu_2**L3**$^{RR}{}_3$]: (37.6 mg, 0.0108 mmol, 82.3% yield), ^{1}H NMR (400 MHz, CD_3CN, 299 K, δ): 8.02 (d, J = 49.6 Hz, 3 x 4H), 7.47 (d, J = 38.6 Hz, 3 x 2H), 7.23 (s, 3 x 4H), 7.16 (d, J = 5.2 Hz, 3 x 6H), 7.07 (s, 3 x 4H), 6.97 (s, 3 x 4H), 6.79 (s, 3 x 2H), 6.59 (s, 3 x 4H), 5.36 (s, 3 x 2H), 5.03 (s, 3 x 2H), 1.73 (d, J = 22.2 Hz, 3 x 6H). ^{13}C NMR (125 MHz, CD_3CN, 300 K, δ): 155.62, 142.66, 138.99, 138.10, 135.80, 128.64, 127.58, 127.20, 127.07, 125.61, 122.56, 92.56, 91.97, 51.29, 21.55. HRMS (ESI) calcd. for $C_{147}H_{120}Eu_2F_9N_{18}O_{21}S_3$ [Eu_2(**L3^{RR}**)$_3$ + 3OTf$^-$]$^{3+}$: 1015.2111, found 1015.2097. Elemental analysis calcd. for $C_{150}H_{120}F_{18}N_{18}O_{30}S_6Eu_2 \cdot 5H_2O$: C 50.28, H 3.66, N 7.04, found: C 50.26, H 4.03, N 6.99.

[Gd_2**L3**$^{SS}{}_3$]: (39.5 mg, 0.0113 mmol, 86.2% yield), HRMS (ESI) calcd. for $C_{144}H_{117}Gd_2N_{18}O_{12}$ [Gd_2(**L3^{SS}**)$_3$ – 3H$^+$]$^{3+}$: 868.5869, found 868.5845. Elemental analysis calcd. for $C_{150}H_{120}F_{18}N_{18}O_{30}S_6Gd_2 \cdot 18H_2O$: C 47.07, H 4.11, N 6.59, found: C 46.97, H 3.72, N 6.40.

[Gd_2**L3**^{RR}$_3$]: (37.6 mg, 0.0107 mmol, 82.0% yield), HRMS (ESI) calcd. for $C_{147}H_{120}F_9Gd_2N_{18}O_{21}S_3$ [Gd_2(**L3**^{RR})$_3$ + 3OTf$^-$]$^{3+}$: 1018.5452, found 1018.5465. Elemental analysis calcd. for $C_{150}H_{120}F_{18}N_{18}O_{30}S_6Gd_2·23H_2O$: C 45.99, H 4.27, N 6.44, found: C 45.88, H 3.76, N 6.21.

[Tb_2**L3**^{SS}$_3$]: (37.2 mg, 0.0106 mmol, 81.1% yield), HRMS (ESI) calcd. for $C_{147}H_{120}F_9Tb_2N_{18}O_{21}S_3$ [Tb_2(**L3**^{SS})$_3$ + 3OTf$^-$]$^{3+}$: 1019.5472, found 1019.5485. Elemental analysis calcd. for $C_{150}H_{120}F_{18}N_{18}O_{30}S_6Tb_2·16H_2O$: C 47.47, H 4.04, N 6.64, found: C 47.58, H 3.87, N 6.56.

[Tb_2**L3**^{RR}$_3$]: (38.6 g, 0.0110 mmol, 84.1% yield), HRMS (ESI) calcd. for $C_{147}H_{120}F_9Tb_2N_{18}O_{21}S_3$ [Tb_2(**L3**^{RR})$_3$ + 3OTf$^-$]$^{3+}$: 1019.5472, found 1019.5485. Elemental analysis calcd. for $C_{150}H_{120}F_{18}N_{18}O_{30}S_6Tb_2·18H_2O$: C 47.03, H 4.10, N 6.58, found: C 47.02, H 4.06, N 6.19.

[Lu_2**L3**^{SS}$_3$]: (38.6 mg, 0.0109 mmol, 83.4% yield), ^{1}H NMR (400 MHz, CD_3CN, 299 K, δ): 10.13 (s, 3 x 2H), 8.99 (d, *J* = 7.0 Hz, 3 x 2H), 8.54 (dd, *J* = 6.0, 2.8 Hz, 3 x 2H), 8.46 (d, *J* = 6.0 Hz, 3 x 4H), 7.83 – 7.66 (m, 3 x 4H), 7.27 (dt, *J* = 5.6, 2.8 Hz, 3 x 6H), 7.12 – 7.00 (m, 3 x 4H), 6.89 (d, *J* = 8.5 Hz, 3 x 4H), 6.83 (s, 3 x 4H), 4.95 (t, *J* = 7.0 Hz, 3 x 2H), 1.69 (d, *J* = 7.0 Hz, 3 x 6H). ^{13}C NMR (101 MHz, CD_3CN, 300K, δ): 166.79, 166.00, 147.36, 146.91, 142.84, 141.66, 138.40, 137.88, 135.37, 128.72, 127.58, 127.16, 126.75, 126.06, 125.62, 120.98, 52.16, 20.90. HRMS (ESI) calcd. for $C_{145}H_{118}F_3Lu_2N_{18}O_{15}S$ [Lu_2(**L3**^{SS})$_3$ + OTf$^-$ - 2H$^+$]$^{3+}$: 930.2509, found 930.2584. Elemental analysis calcd. for $C_{150}H_{120}F_{18}N_{18}O_{30}S_6Lu_2·3H_2O$: C 50.14, H 3.53, N 7.02, found: C 50.10, H 3.74, N 6.90.

[Lu_2**L3**^{RR}$_3$]: (39.3 mg, 0.0111 mmol, 85.0% yield), ^{1}H NMR (400 MHz, CD_3CN, 299 K, δ): 10.18 (s, 3 x 2H), 9.03 (d, *J* = 7.1 Hz, 3 x 2H), 8.54 (d, *J* = 6.2 Hz, 3 x 2H), 8.52 – 8.38 (m, 3 x 4H), 7.76 (d, *J* = 8.4 Hz, 2H), 7.36 – 7.21 (m, 3 x 4H), 7.11 – 6.99 (m, 3 x 4H), 6.89 (d, *J* = 8.5 Hz, 3 x 4H), 6.83 (s, 3 x 4H), 4.94 (t, *J* = 7.0 Hz, 3 x 2H), 1.69 (d, *J* = 7.0 Hz, 3 x 6H). ^{13}C NMR (101 MHz, CD_3CN, 300 K, δ): 166.79, 165.98, 147.36, 146.92, 142.82, 141.67, 138.38, 137.83, 135.37, 128.72, 127.58, 127.14, 126.74, 126.07, 125.63, 120.99, 52.18, 20.92. HRMS (ESI) calcd. for $C_{147}H_{120}F_9Lu_2N_{18}O_{21}S_3$ [Lu_2(**L3**^{SS})$_3$ + 3OTf$^-$]$^{3+}$: 1030.2241, found 1030.2292. Elemental analysis calcd. for $C_{150}H_{120}F_{18}N_{18}O_{30}S_6Lu_2·3H_2O$: C 50.14, H 3.53, N 7.02, found: C 50.09, H 3.95, N 7.00.

Data for result and discussion

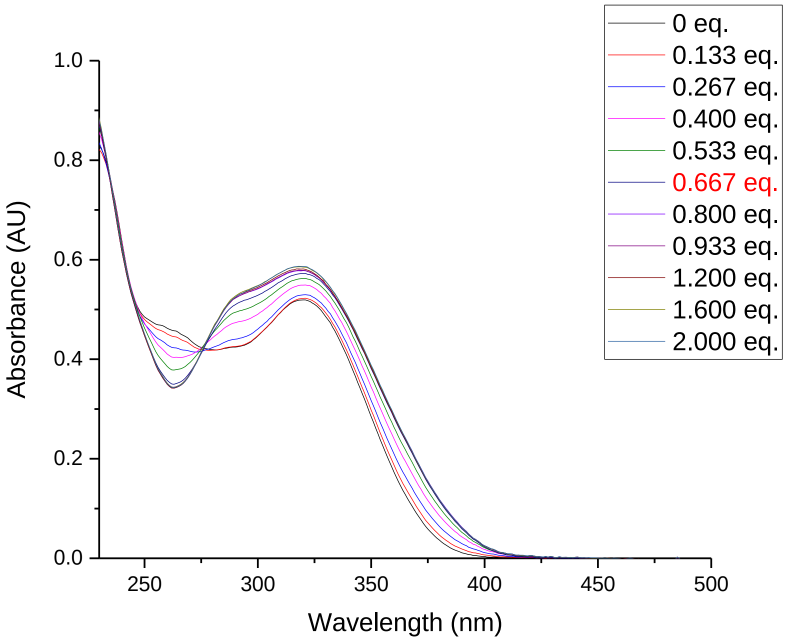

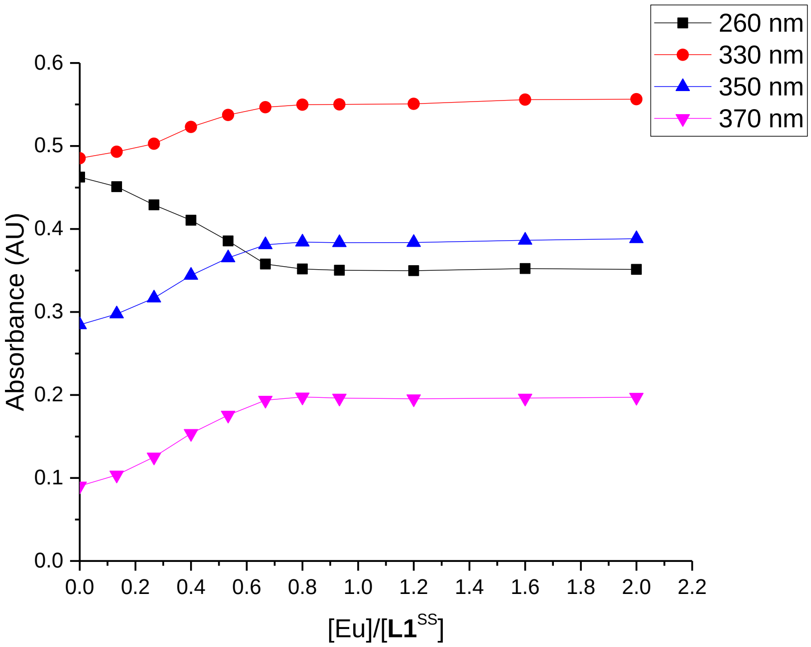

Supplementary Figure 1. UV-Vis titration of **L1** with $Eu(OTf)_3$.(Upper) Variation in UV-Vis absorption spectra of titrating $\mathbf{L1}^{SS}$ (2.79 x 10^{-4}M, in 48:4:48, v/v/v, of $CHCl_3$/MeOH/CH_3CN) with $Eu(OTf)_3$ (0.036M in MeOH) at 298K (Eu:$\mathbf{L1}^{SS}$ = 0.0–2.0). (Bottom)Variation of absorbance at four different wavelengths upon titrating $\mathbf{L1}^{SS}$ with $Eu(OTf)_3$.

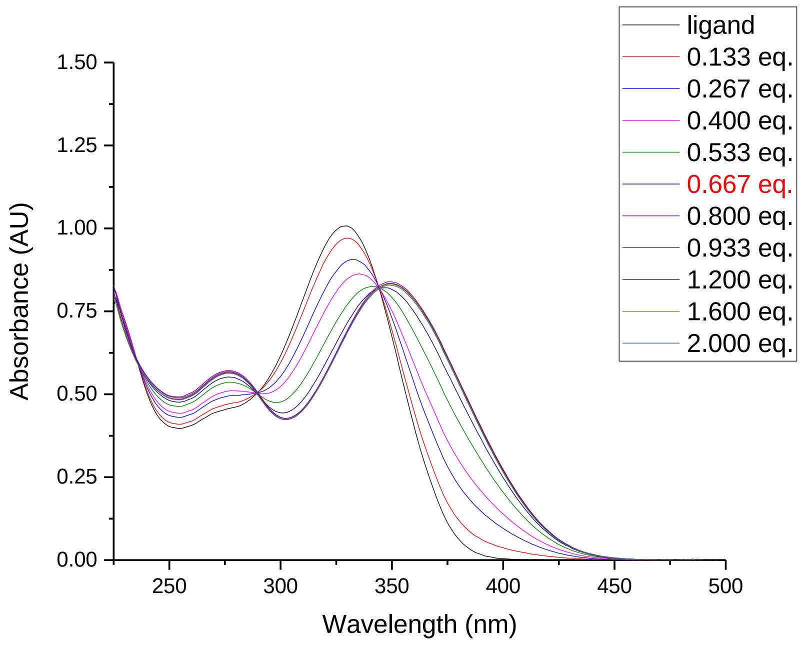

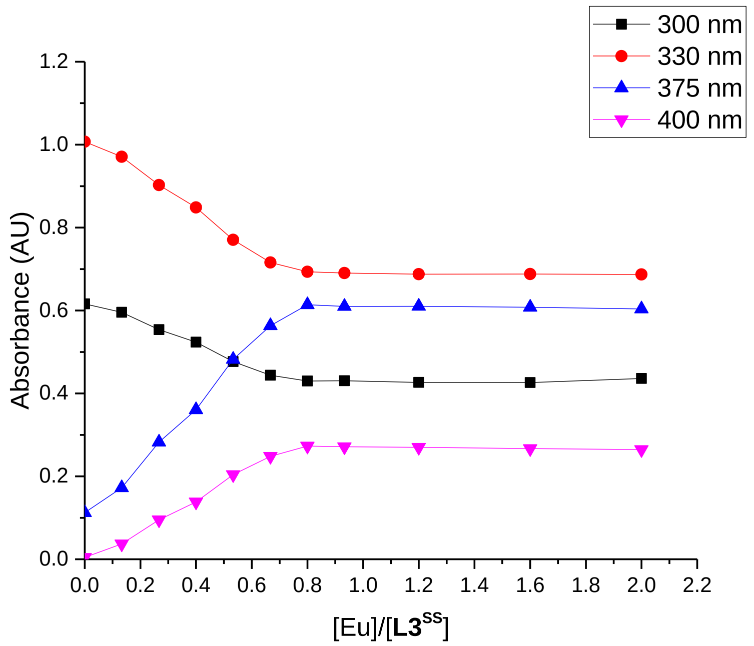

Supplementary Figure 2. UV-Vis titration of **L3** with $Eu(OTf)_3$.(Upper) Variation in UV-Vis absorption spectra of titrating **L3**^{SS} (1.96 x 10^{-4}M, in 48:4:48, v/v/v, of $CHCl_3$/MeOH/CH_3CN) with $Eu(OTf)_3$ (0.014M in MeOH) at 298K (Eu:**L3**^{SS} = 0.0–2.0). (Bottom)Variation of absorbance at four different wavelengths upon titrating **L3**^{SS} with $Eu(OTf)_3$.

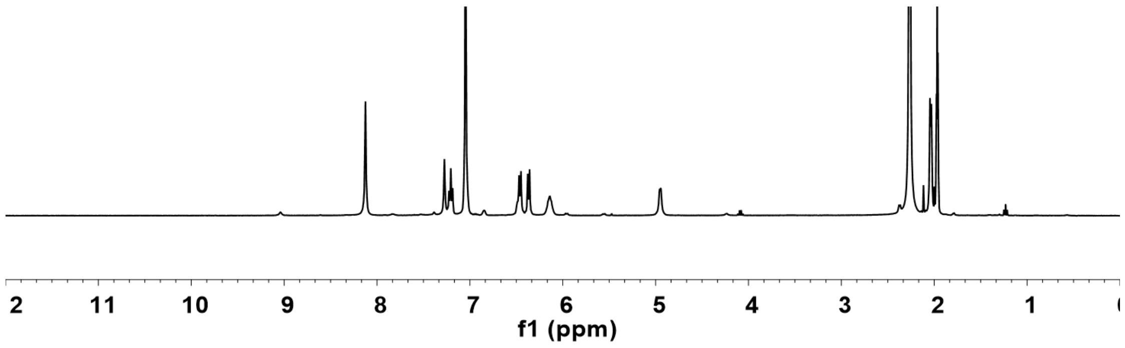

Supplementary Figure 3. Proton NMR of [Eu_2**L1**$_3$] (complexation using DCM/MeCN as solvent).

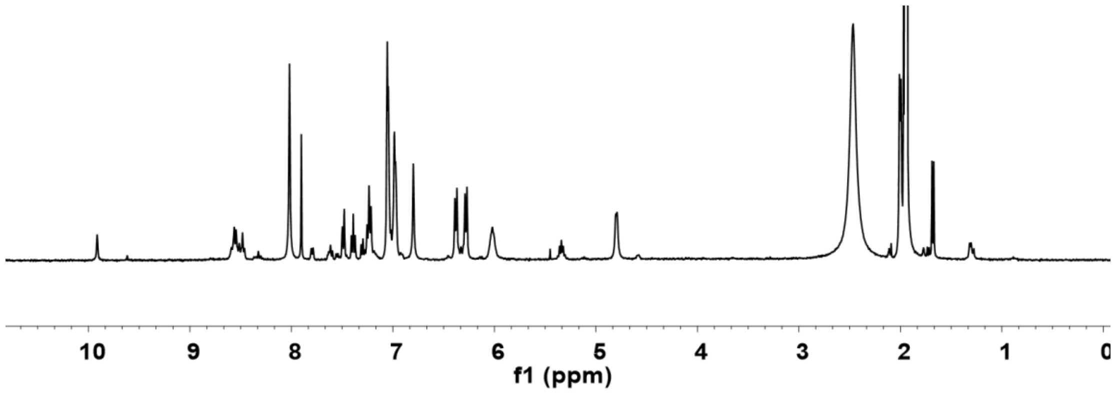

Supplementary Figure 4. Proton NMR of [Eu_2**L1**$_3$](ClO_4)$_6$. Free ligand peaks were observed due to the high water content (50%) in perchlorate solution.

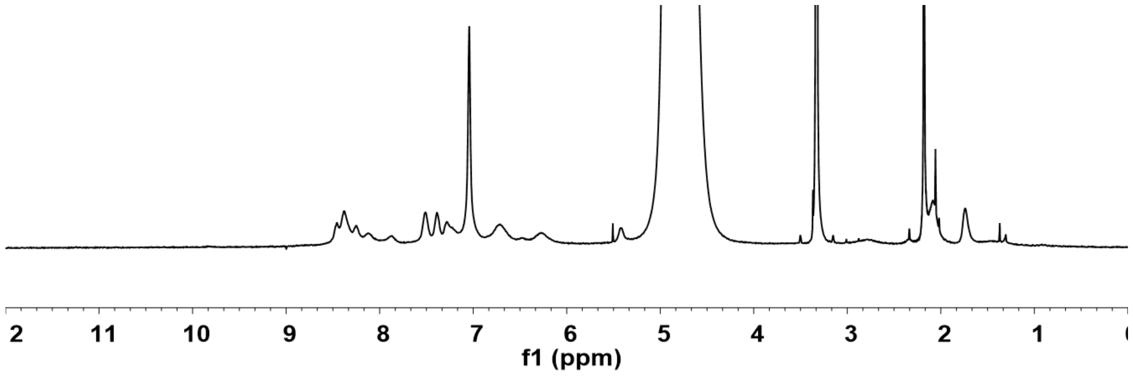

Supplementary Figure 5. Proton NMR of [Eu_2**L1**$_3$]Cl_6.

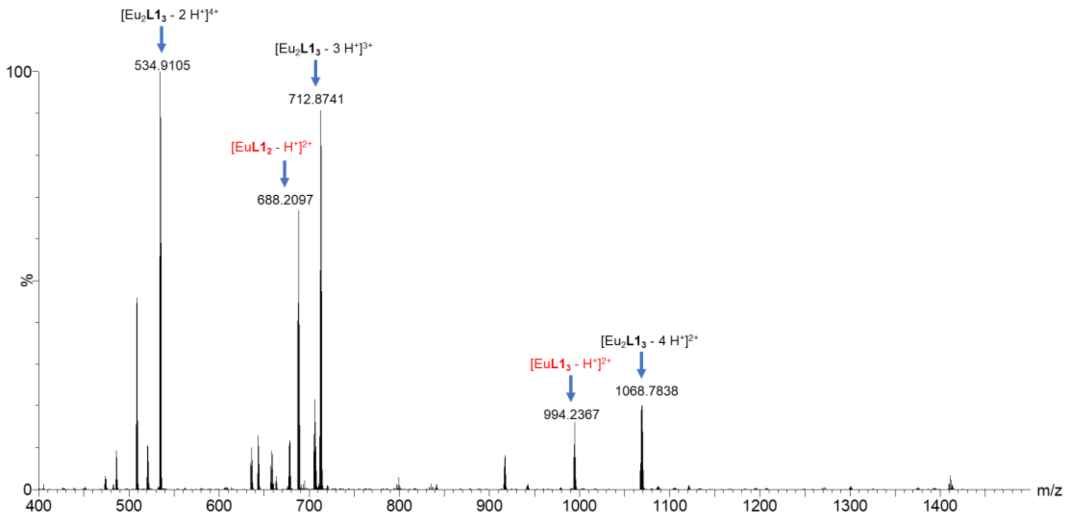

Supplementary Figure 6. ESI-HRMS of $[Eu_2\mathbf{L1}_3]Cl_6$. Complex is only soluble in MeOH and dissociation of complex was found in ESI-HRMS.

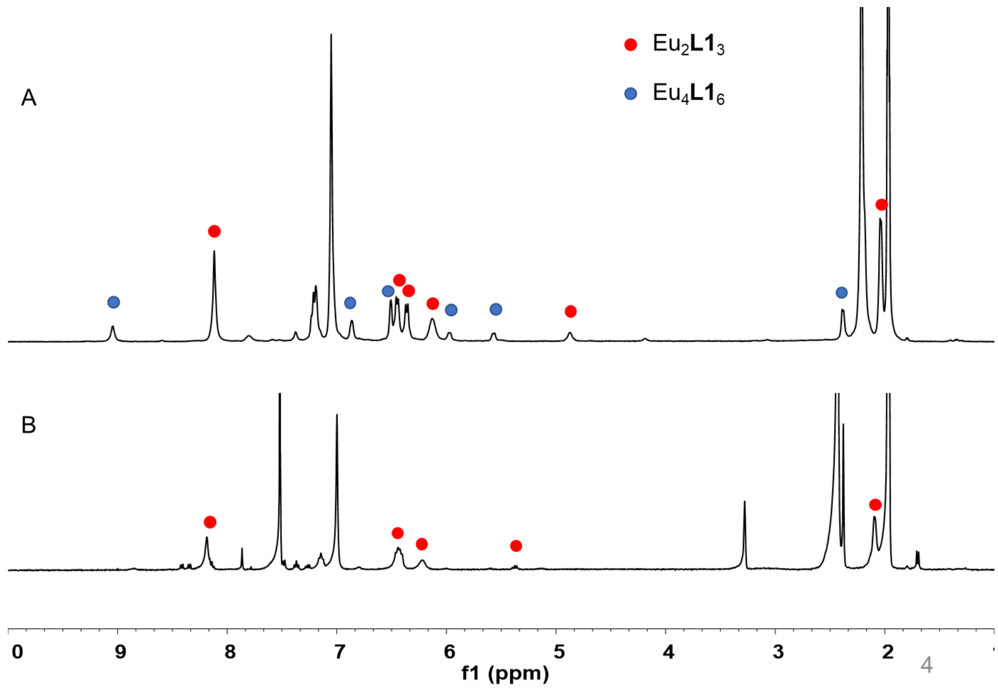

Supplementary Figure 7. ^{1}HNMR of (A) crude Eu complexes prepared by 0.008M **L1** (B) 0.0001M **L1** in $CDCl_3$/MeOD/CD_3CN (12/1/12, v/v/v)

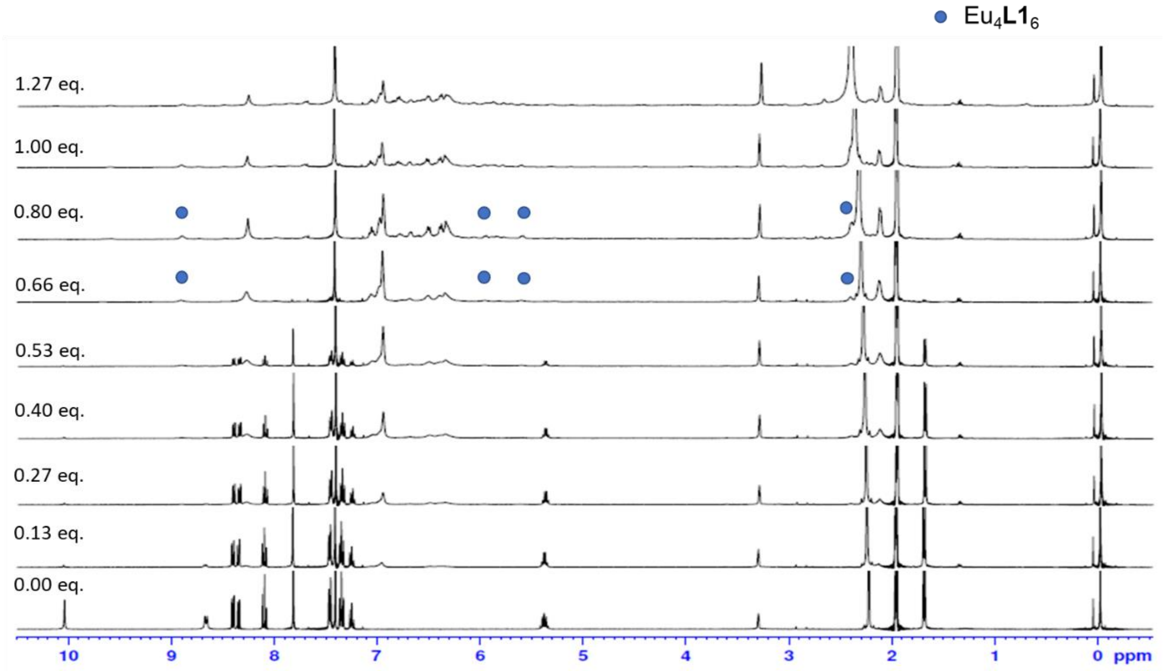

Supplementary Figure 8. Variation in ^{1}HNMR spectra of **L1^{RR}** (2.15 x 10^{-3}M in 70:5:25, v/v/v of $CDCl_3$/CD_3OD/CD_3CN) with Eu(OTf)$_3$ (0.09M in CD_3OD) at 298 K.

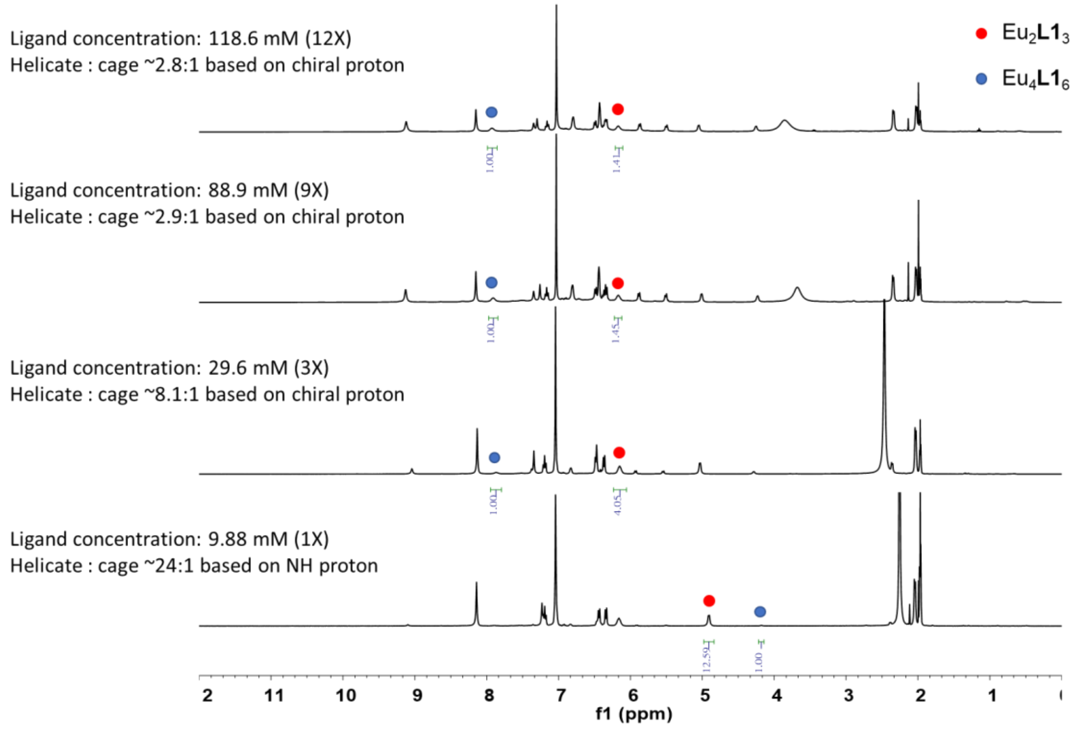

Supplementary Figure 9. ^{1}HNMR spectra of [Eu$_2$(**L1**)$_3$]/[Eu$_4$(**L1**)$_6$] at different ligand concentration in CD_3CN at 298 K.

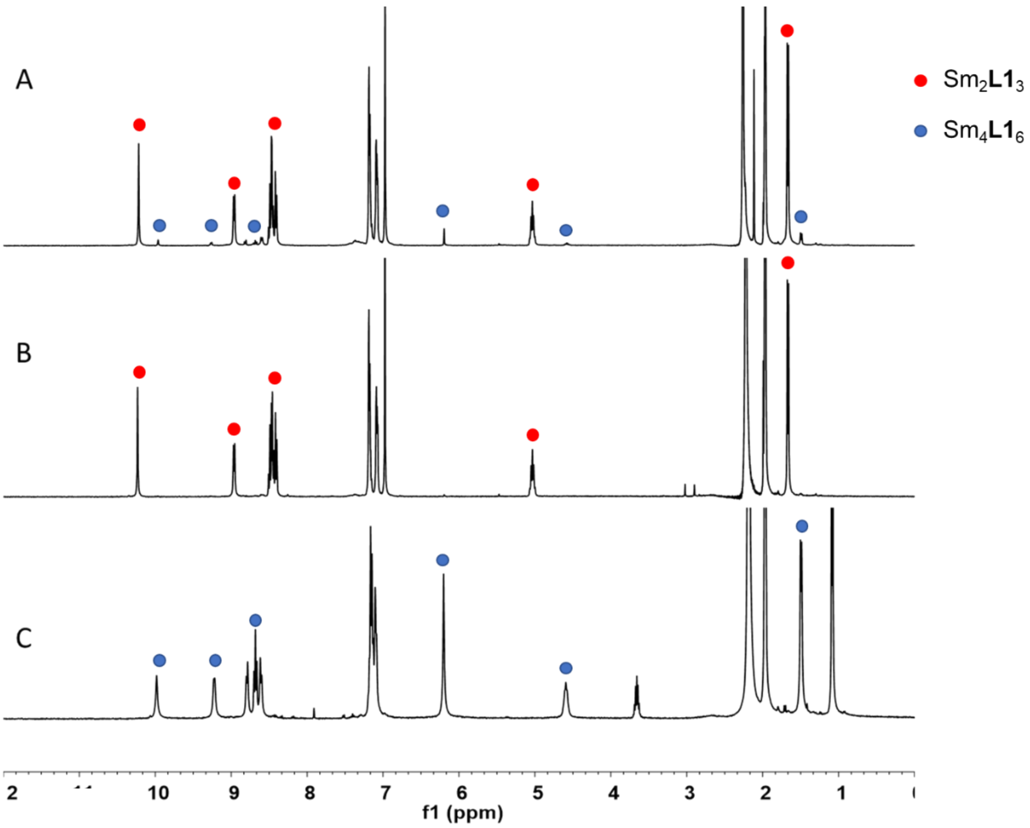

Supplementary Figure 10. ^{1}HNMR of (A) crude mixture of [Sm_2(**L1**)$_3$] and [Sm_4(**L1**)$_6$], (B) pure [Sm_2(**L1**)$_3$] and (C) pure [Sm_4(**L1**)$_6$] in CD_3CN.

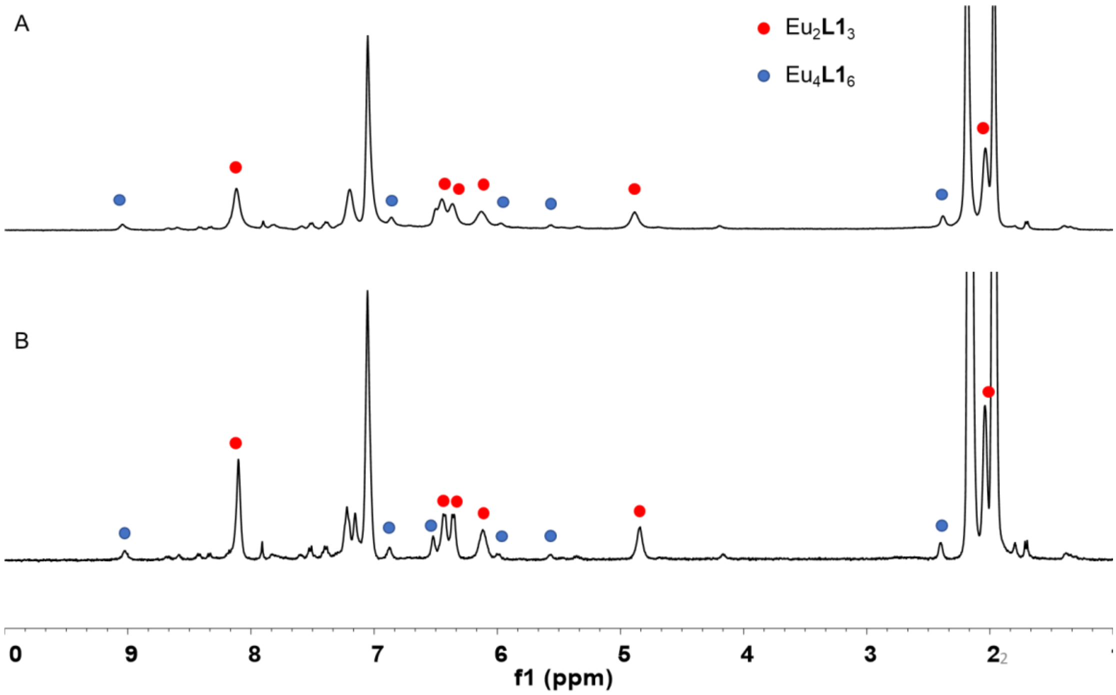

Supplementary Figure 11. ^{1}HNMR of (A) crude Eu complexes prepared by 0.006M **L1** in CD_3CN and (B) subjected to 5-fold dilution and performed ^{1}HNMR instantaneously.

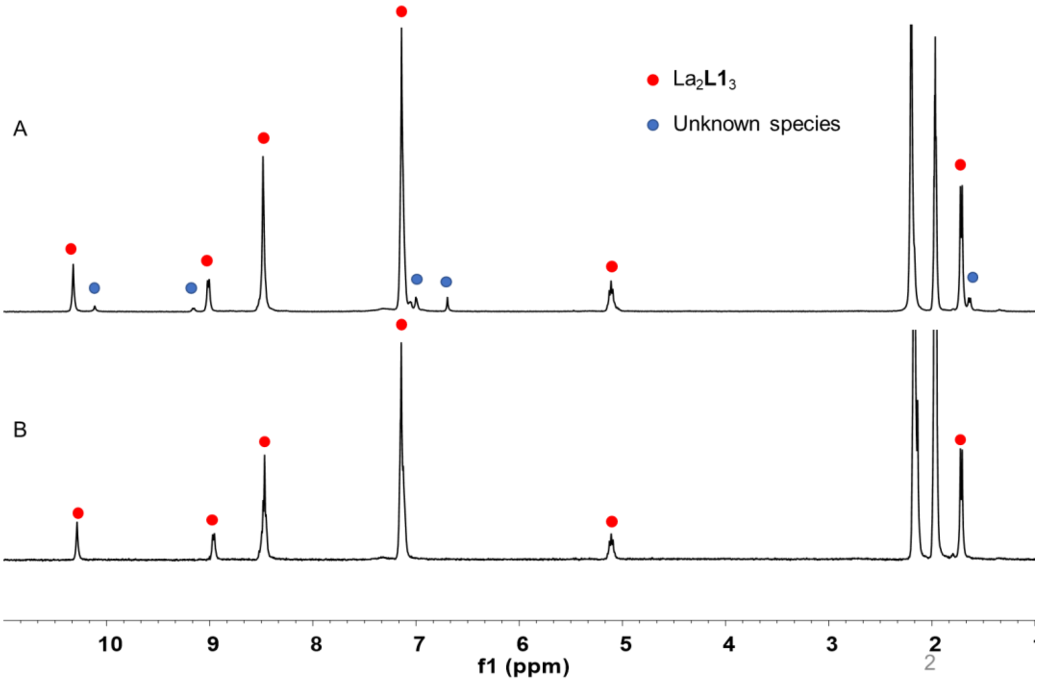

Supplementary Figure 12. ^{1}HNMR of (A) crude La complexes prepared by 0.006M **L1** in CD_3CN and (B) subjected to 5-fold dilution and performed ^{1}HNMR instantaneously. We hypothesized that the unknown species was La_4**L1**$_6$. However, the exact structure cannot be confirmed due to the poor stability of the unknown species upon dissolution.

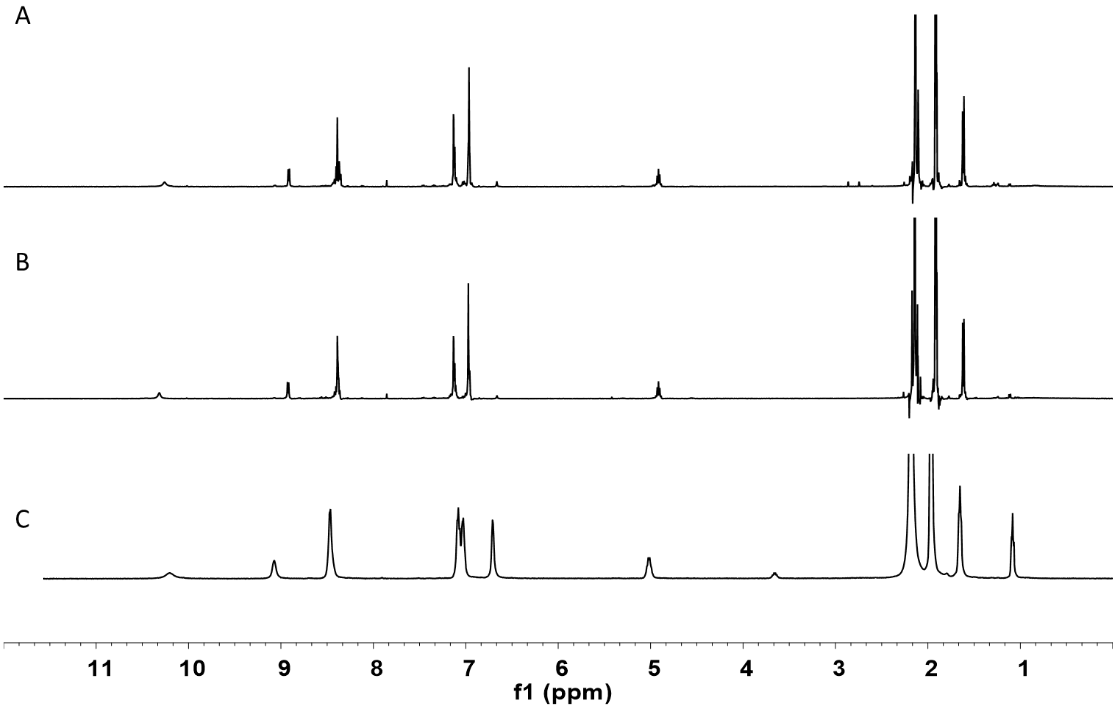

Supplementary Figure 13. ^{1}HNMR of (A) crude [Lu_2(**L1**)$_3$] prepared by 0.008M **L1**, (B) crude [Lu_2(**L1**)$_3$] prepared by 0.001M **L1** and (C) pure [Lu_4(**L1**)$_6$] in CD_3CN.

A

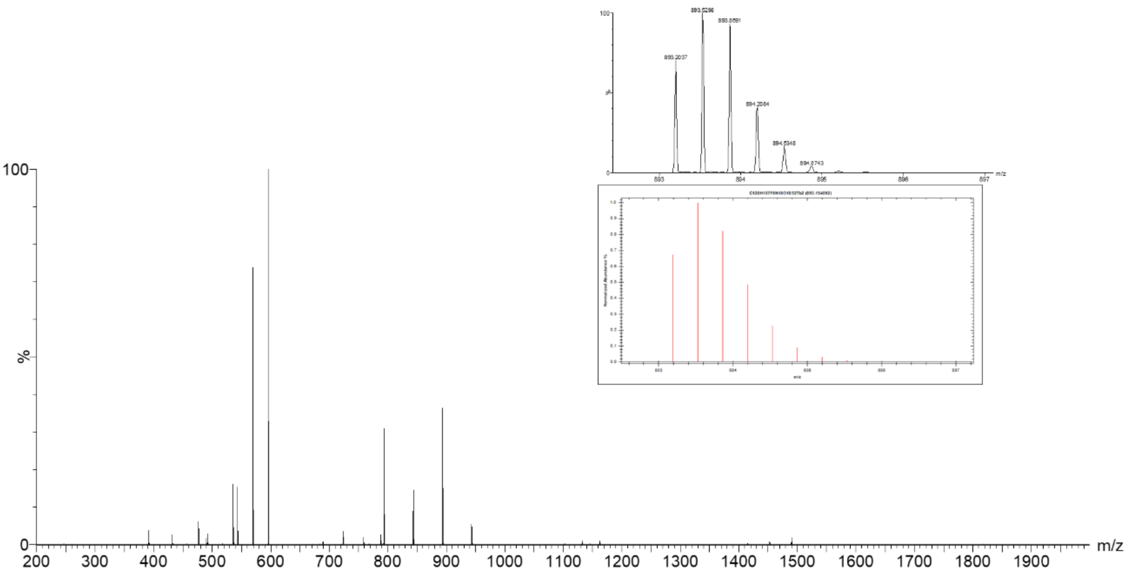

B

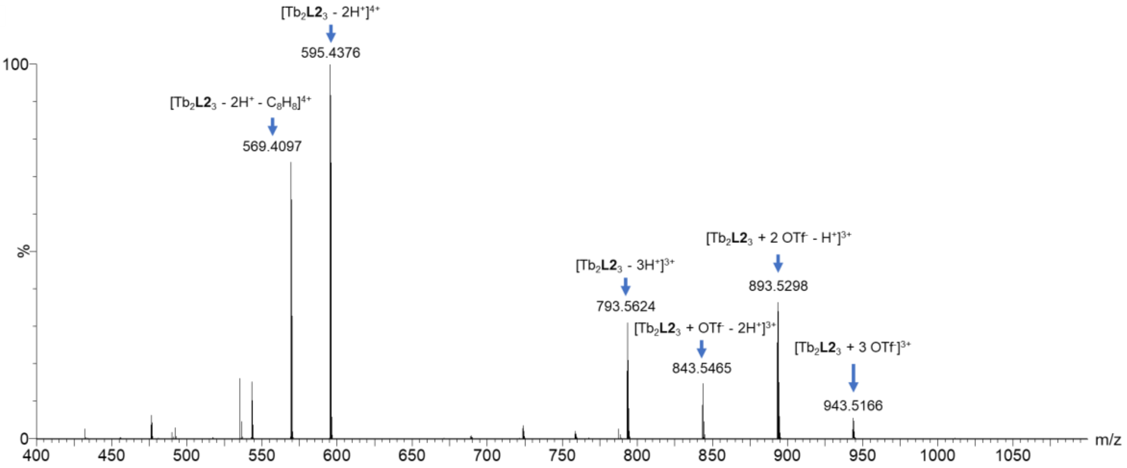

Supplementary Figure 14. ESI-HRMS of tetrahedron [Tb_2**L2**$_3$]. (A) The full spectrum. Simulated m/z for [Tb_2**L3**$_3$ + 2OTf$^-$ - H^+]$^{3+}$ is 893.5292(100%), Experimental found m/z is 893.5298 (100%). Inset showing the experimental (upper) and calculated(lower) isotopic patterns. (B) Expanded region of the mass spectrum to show the possible assignments of the corresponding prominent peaks.

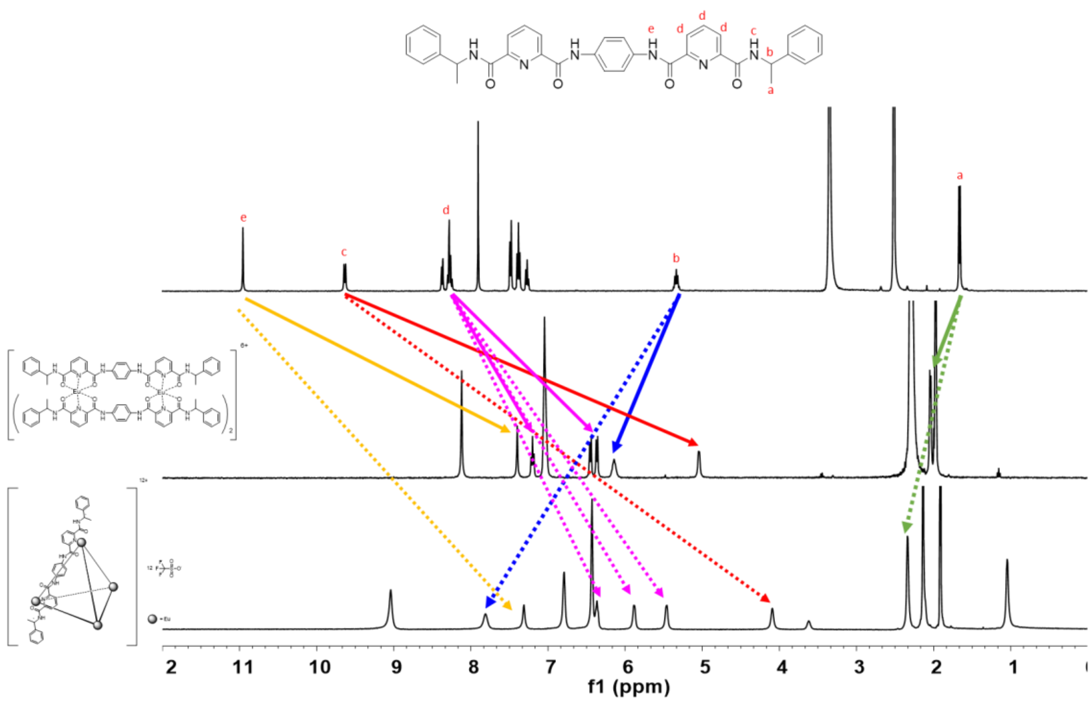

Supplementary Figure 15. Paramagnetic shift of [Eu_2(**L1**)$_3$] and [Eu_4(**L1**)$_6$] in CD_3CN.

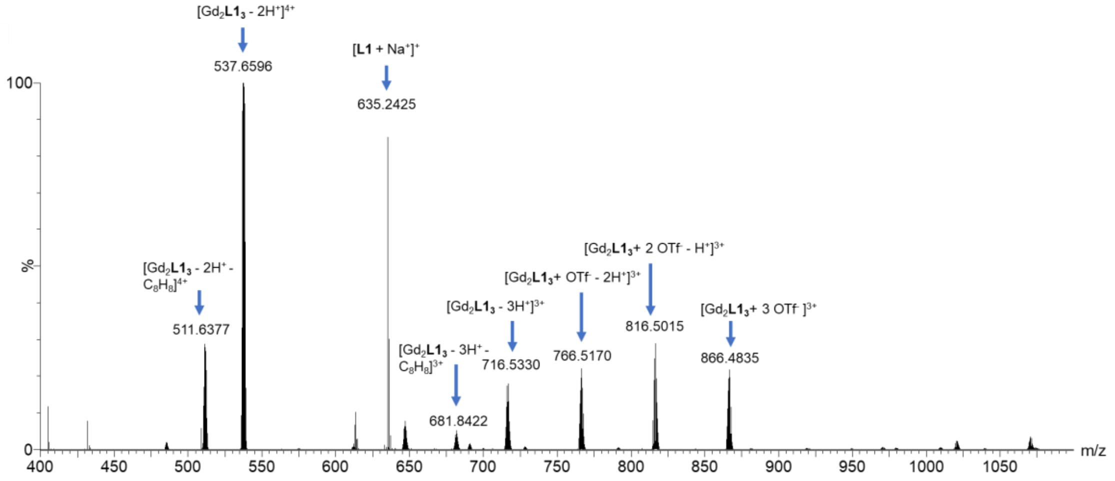

Supplementary Figure 16. ESI-HRMS of tetrahedron [Gd_4**L1**$_6$] after dissolution in CH_3CN for 14 days. The ESI-HRMS result revealed the supramolecular transformation from [Gd_4**L1**$_6$] to [Gd_2**L1**$_3$]. Simulated m/z for [Gd_2**L1**$_3$ + 3 OTf$^-$]$^{3+}$ is 866.4836 (100%), Experimental found m/z is 866.4835(100%).

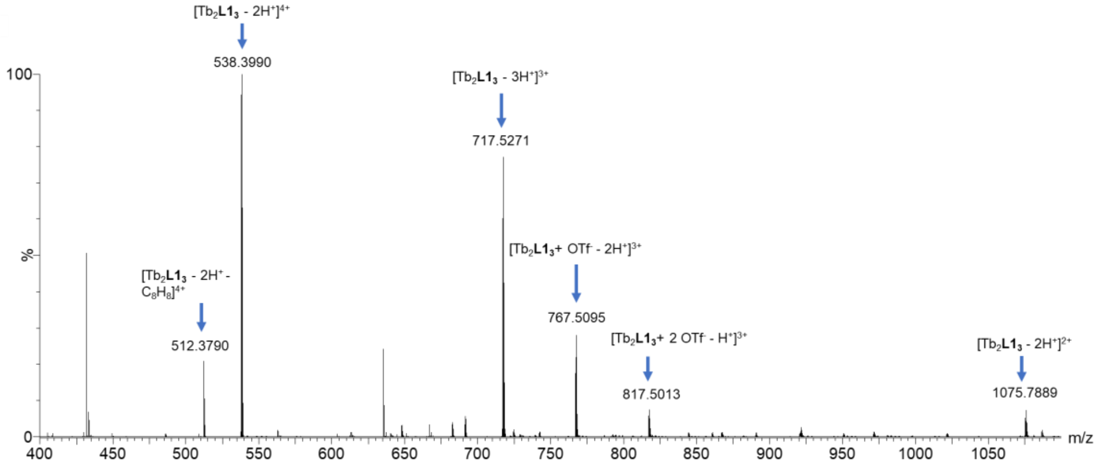

Supplementary Figure 17. ESI-HRMS of tetrahedron [Tb_4**L1**$_6$] after dissolution in CH_3CN for 14 days. The ESI-HRMS result revealed the supramolecular transformation from [Tb_4**L1**$_6$] to [Tb_2**L1**$_3$]. Simulated m/z for [Tb_2**L1**$_3$ – $3H^+$]$^{3+}$ is 717.5271 (100%), Experimental found m/z is 717.5247(100%).

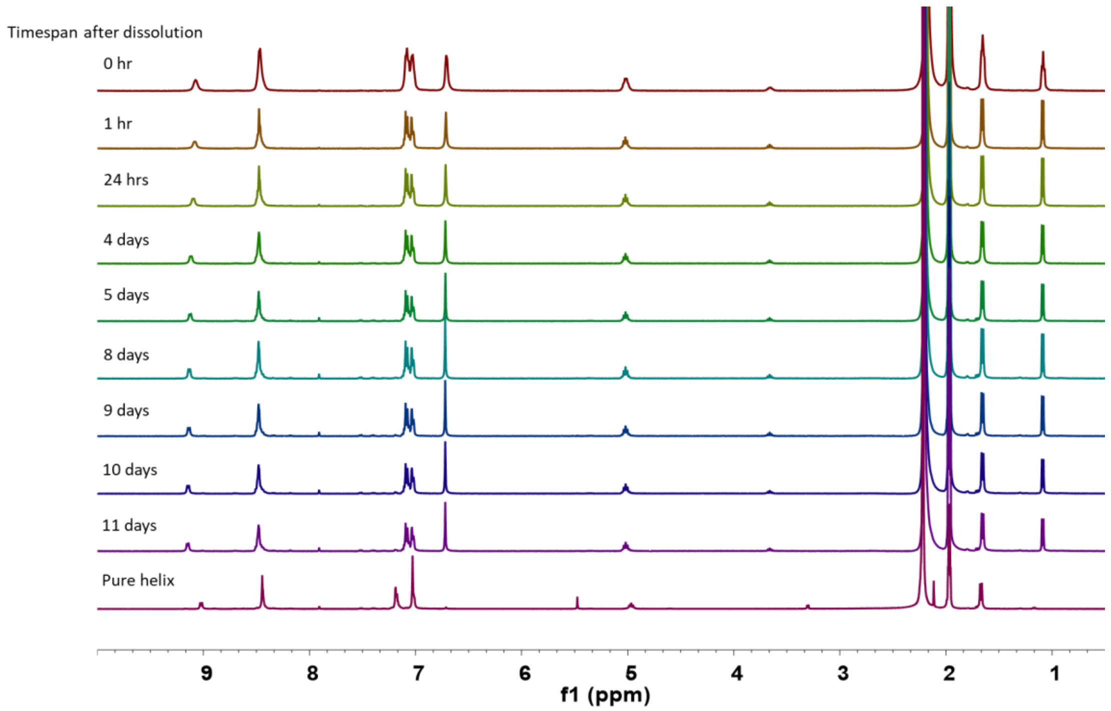

Supplementary Figure 18. ^{1}H NMR of $[Lu_4(\mathbf{L})_6]$ upon dissolution in CD_3CN at different timespan.

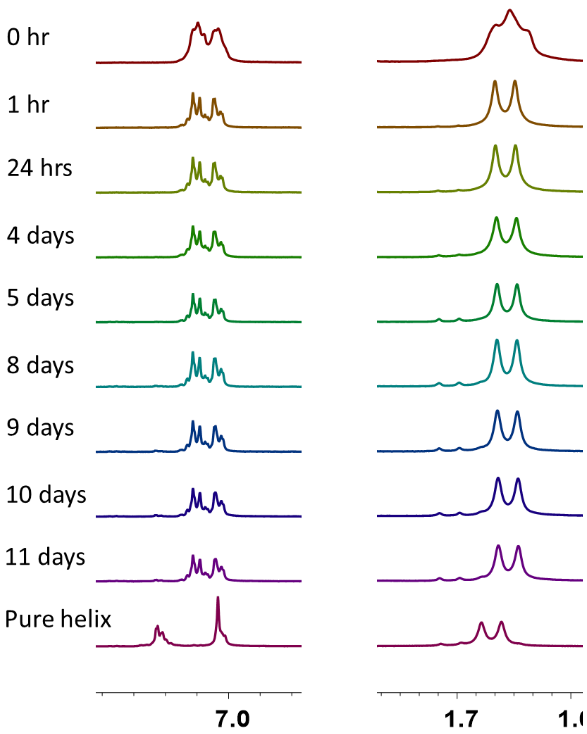

Supplementary Figure 19. Expanded ^{1}H NMR of $[Lu_4(\mathbf{L})_6]$ upon dissolution in CD_3CN at different timespan.

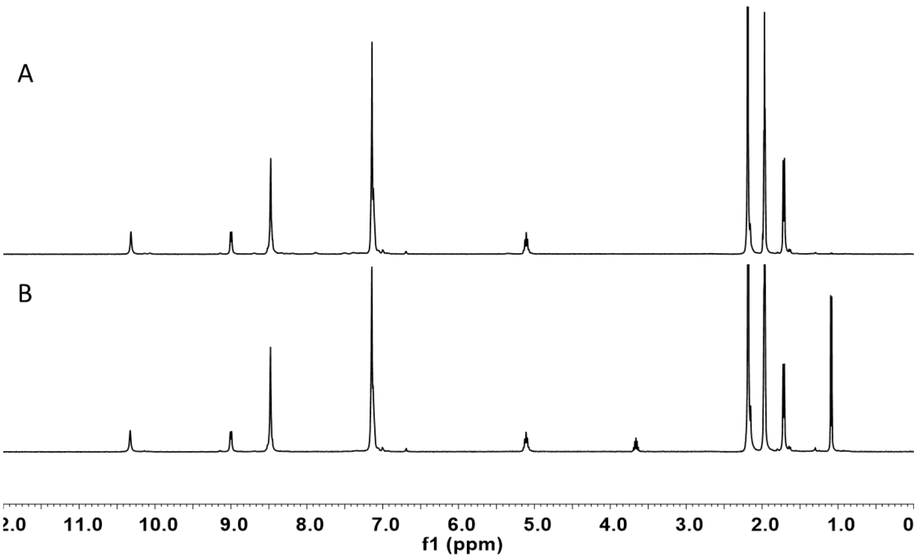

Supplementary Figure 20. ^{1}H NMR of (A) [La_2(**L1**)$_3$] in CD_3CN and (B) the powder left after diffusing ether into a solution containing [La_2(**L1**)$_3$].

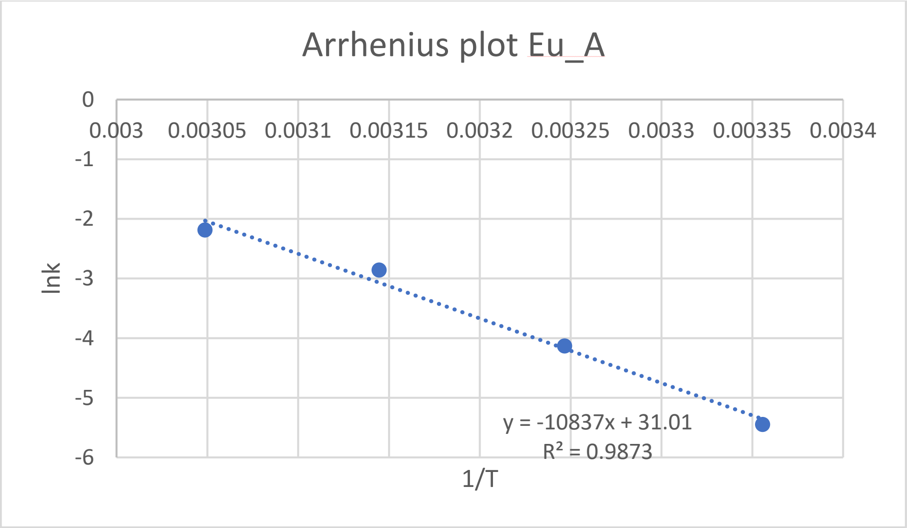

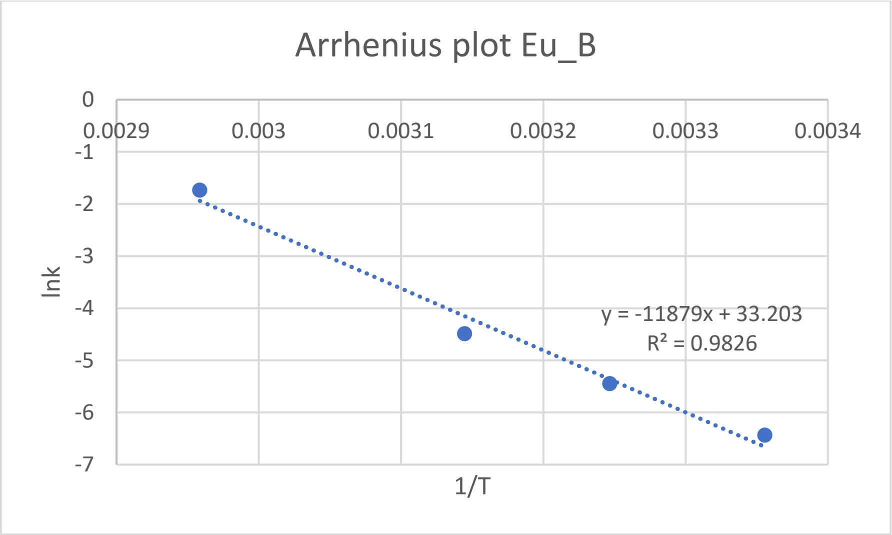

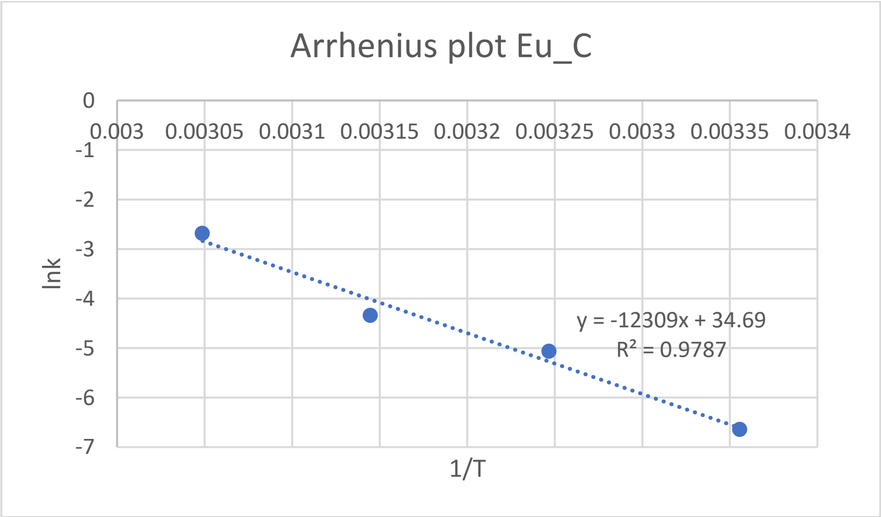

Supplementary Figure 21. Three sets of Arrhenius plot of Eu tetrahedron-to-helicate transformation.

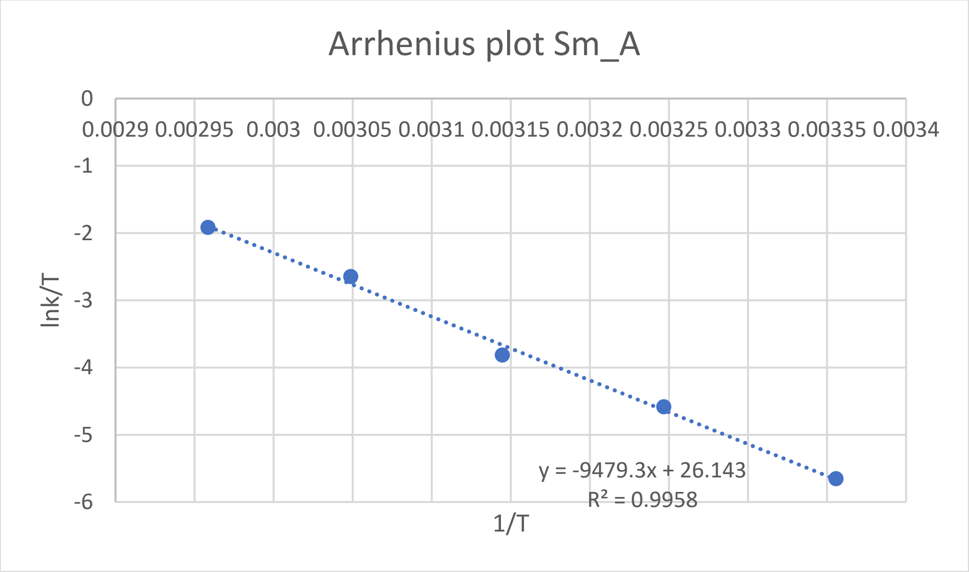
Arrhenius plot Sm_A
0
0.0029 0.00295 0.003 0.00305 0.0031 0.00315 0.0032 0.00325 0.0033 0.00335 0.0034
-1
-2
-3
-4
-5
-6
lnk/T
y = -9479.3x + 26.143
R² = 0.9958
1/T

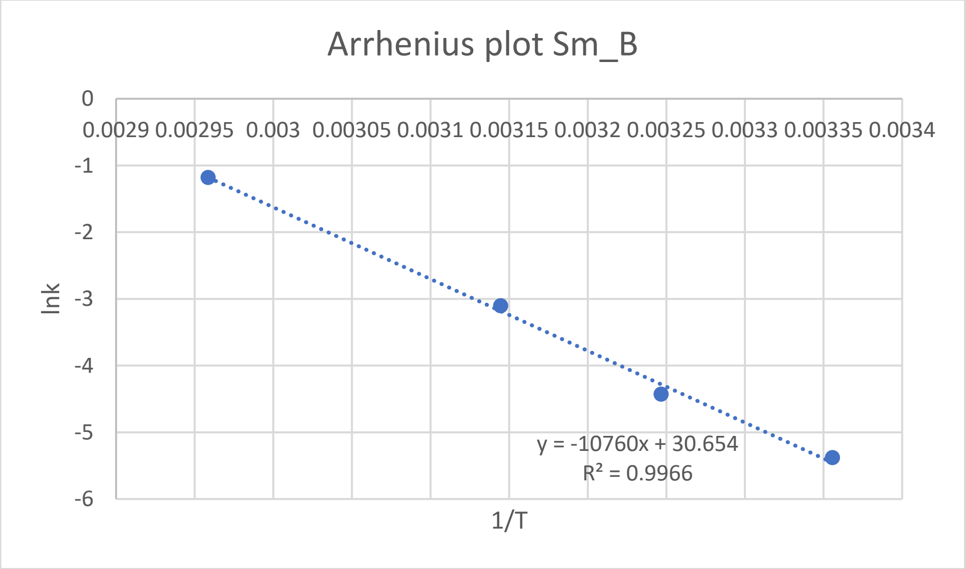
Arrhenius plot Sm_B
0
0.0029 0.00295 0.003 0.00305 0.0031 0.00315 0.0032 0.00325 0.0033 0.00335 0.0034
-1
-2
-3
-4
-5
-6
lnk
y = -10760x + 30.654
R² = 0.9966
1/T

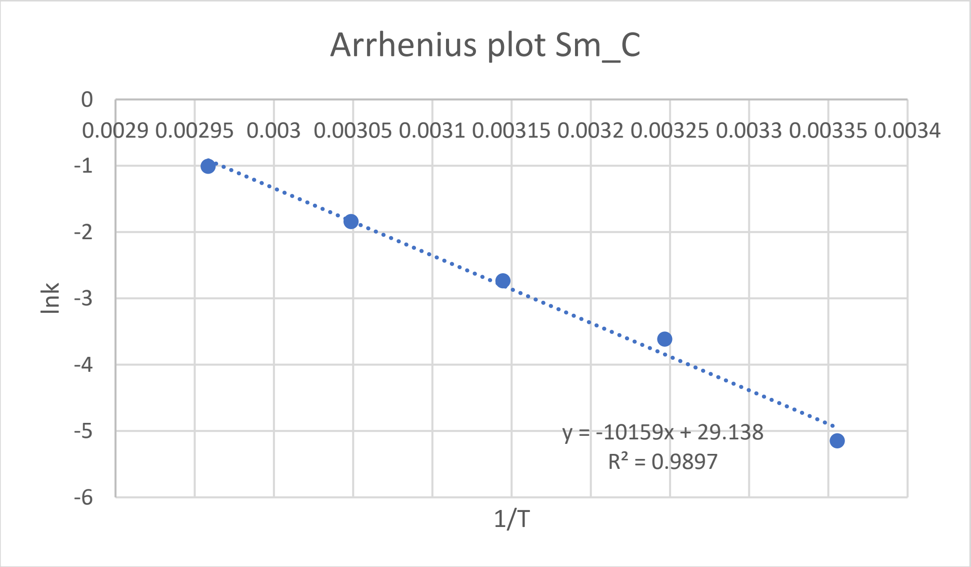
Arrhenius plot Sm_C
0
0.0029 0.00295 0.003 0.00305 0.0031 0.00315 0.0032 0.00325 0.0033 0.00335 0.0034
-1
-2
-3
-4
-5
-6
lnk
y = -10159x + 29.138
R² = 0.9897
1/T

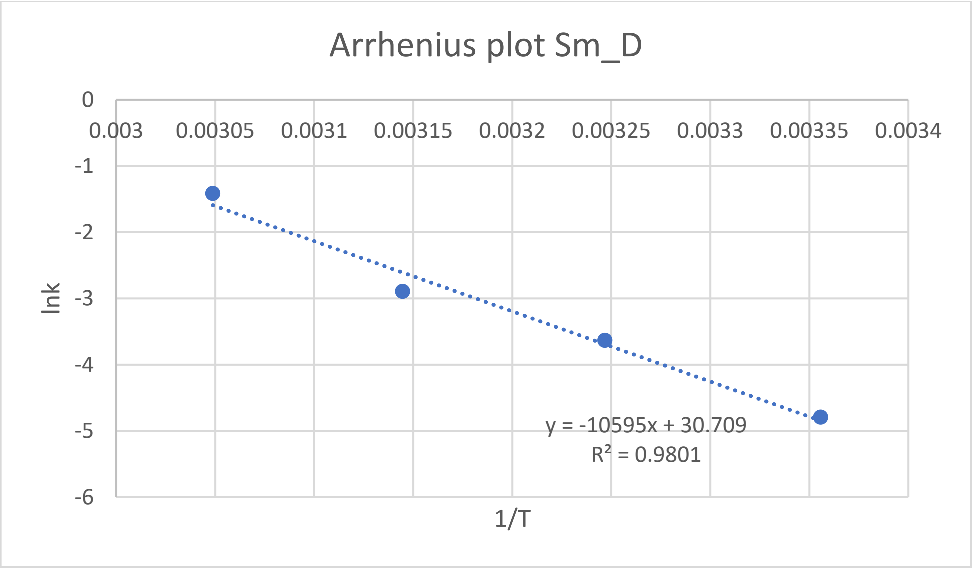

Supplementary Figure 22. Four sets of Arrhenius plot of Sm tetrahedron-to-helicate transformation.

	E_a (kJmol-1)	A	ΔH_{298} (kJmol^{-1})
Eu_A	90.1	2.93 x 10^{13}	+87.6
Eu_B	98.8	2.63 x 10^{13}	+96.3
Eu_C	102.3	1.16 x 10^{15}	+99.8
Average	97.1 (5)	4.05 x 10^{14}	+94.6 (5)

Supplementary Table 1. Kinetic data of Eu tetrahedron-to-helicate transformation.

	E_a (kJmol-1)	A	ΔH_{298} (kJmol^{-1})
Sm_A	78.8	2.25 x 10^{11}	+76.3
Sm_B	89.5	2.06 x 10^{13}	+87.0
Sm_C	84.5	4.51 x 10^{12}	+82.0
Sm_D	88.1	2.17 x 10^{13}	+85.6
Average	85.2 (4)	1.18 x 10^{13}	+82.7 (4)

Supplementary Table 2. Kinetic data of Sm tetrahedron-to-helicate transformation.

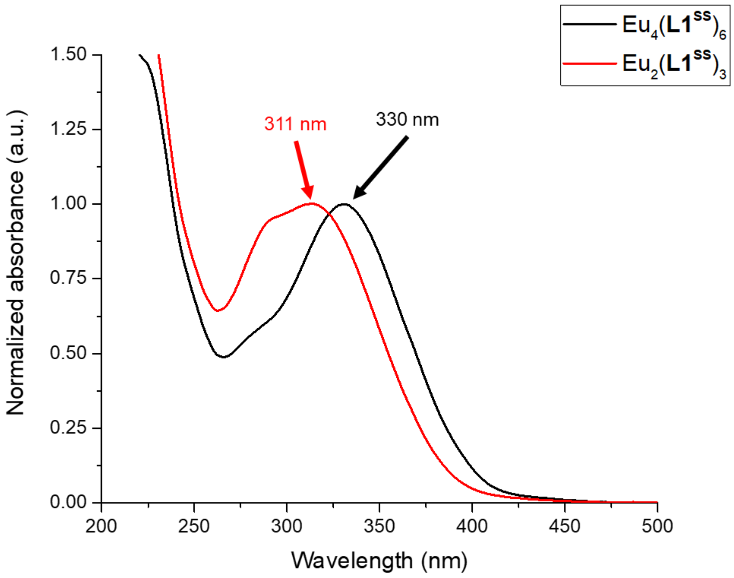

Supplementary Figure 23. Normalized UV-Vis spectrum of [Eu_2(**L1**^{SS})$_3$](OTf)$_6$ and [Eu_4(**L1**^{SS})$_6$](OTf)$_{12}$ in CH_3CN.

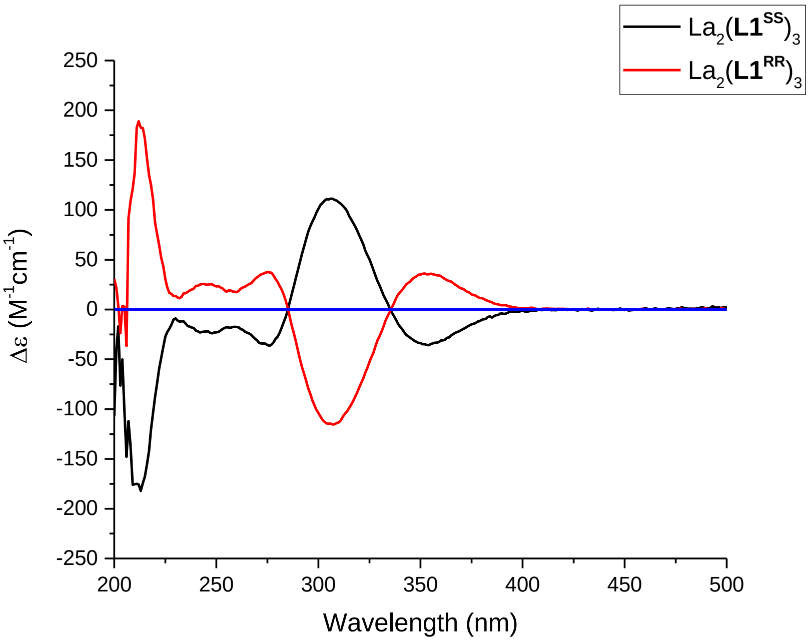

Supplementary Figure 24. CD spectra of [La_2(**L1**^{SS})$_3$](OTf)$_6$ and [La_2(**L1**^{RR})$_3$](OTf)$_6$ in CH_3CN.

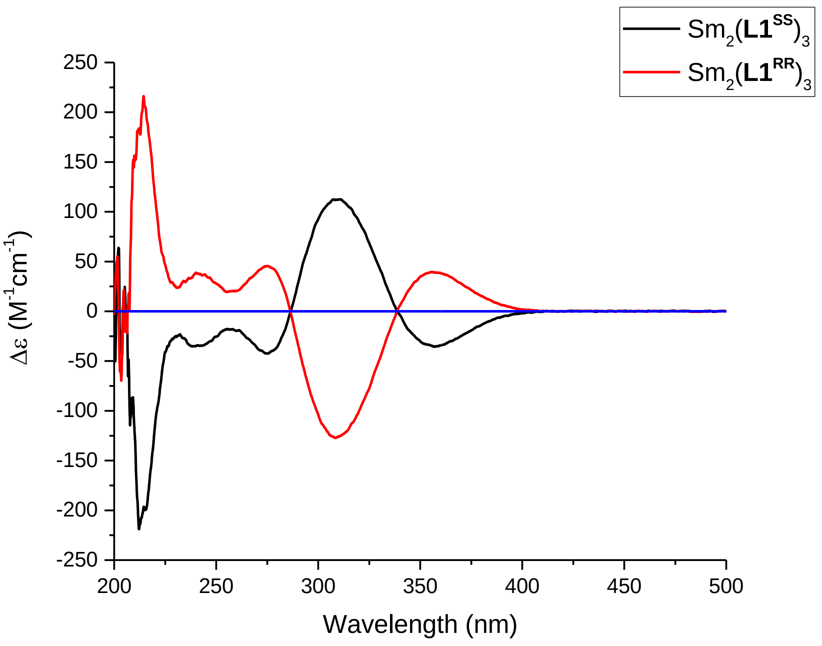

Supplementary Figure 25. CD spectra of [Sm_2(**L1**^{SS})$_3$](OTf)$_6$ and [Sm_2(**L1**^{RR})$_3$](OTf)$_6$ in CH_3CN.

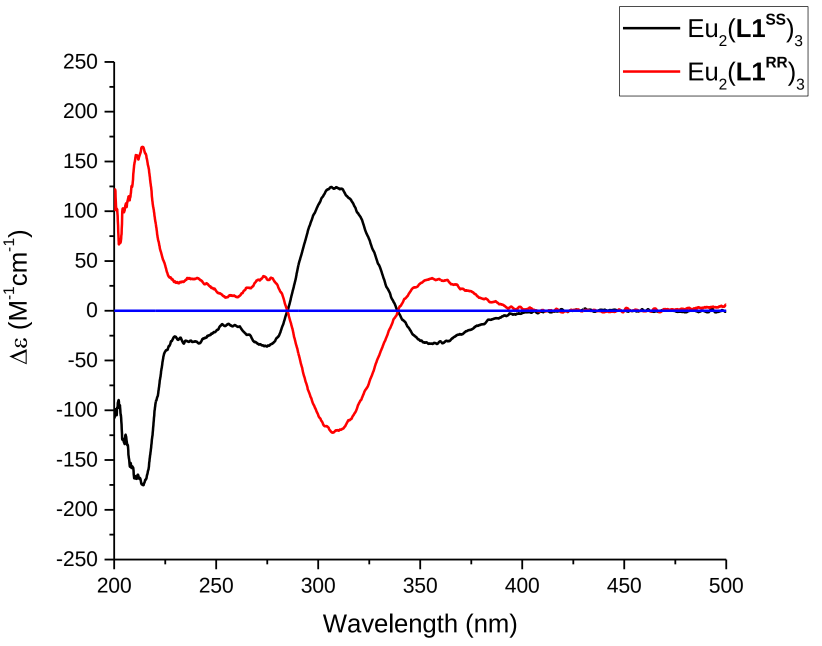

Supplementary Figure 26. CD spectra of [Eu_2(**L1**^{SS})$_3$](OTf)$_6$ and [Eu_2(**L1**^{RR})$_3$](OTf)$_6$ in CH_3CN.

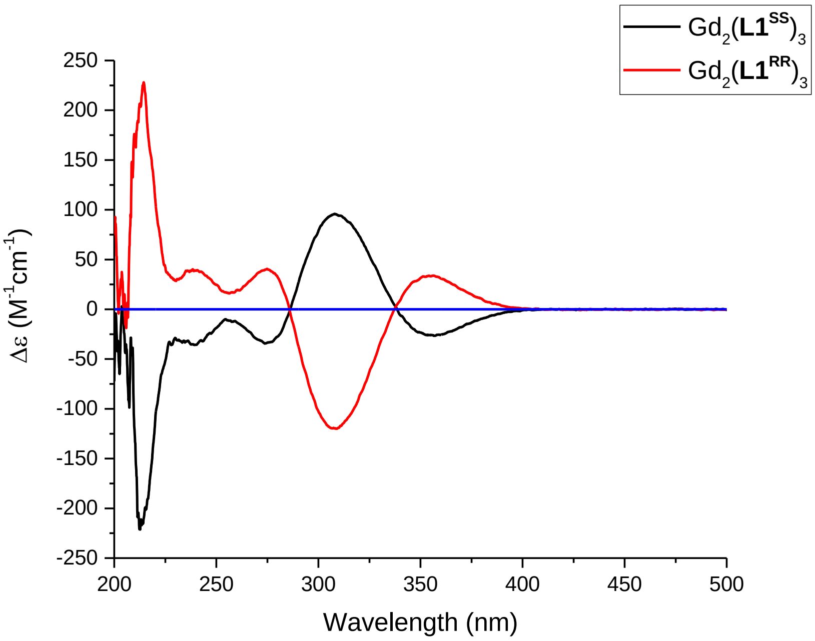

Supplementary Figure 27. CD spectra of [Gd$_2$(**L1**^{SS})$_3$](OTf)$_6$ and [Gd$_2$(**L1**^{RR})$_3$](OTf)$_6$ in CH_3CN.

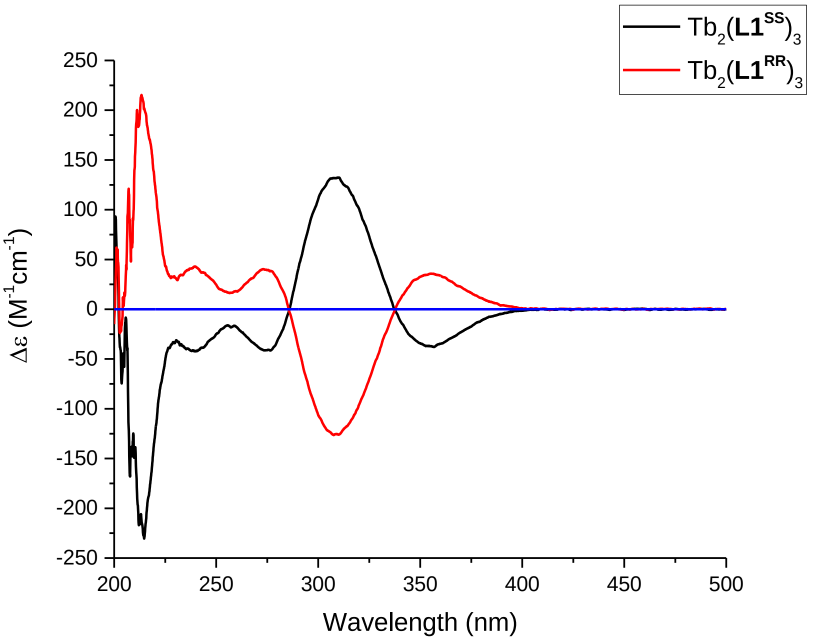

Supplementary Figure 28. CD spectra of [Tb$_2$(**L1**^{SS})$_3$](OTf)$_6$ and [Tb$_2$(**L1**^{RR})$_3$](OTf)$_6$ in CH_3CN.

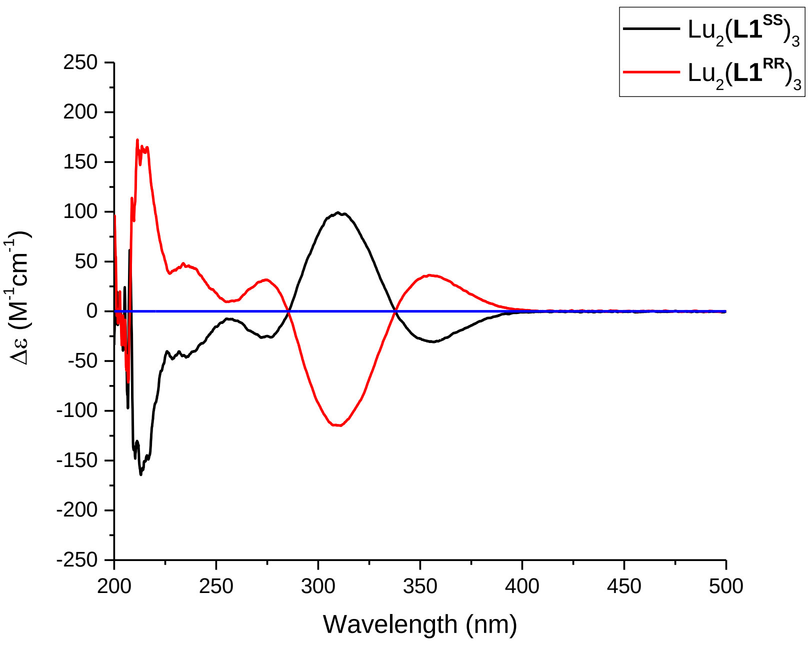

Supplementary Figure 29. CD spectra of [Lu_2(**L1**^{SS})$_3$](OTf)$_6$ and [Eu_2(**L1**^{RR})$_3$](OTf)$_6$ in CH_3CN.

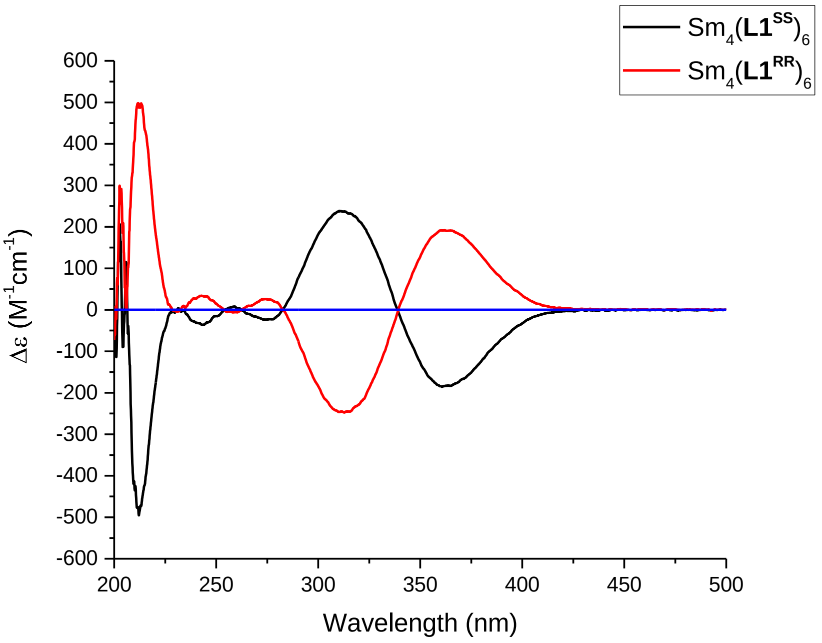

Supplementary Figure 30. CD spectra of [Sm_4(**L1**^{SS})$_6$](OTf)$_{12}$ and [Sm_4(**L1**^{RR})$_6$](OTf)$_{12}$ in CH_3CN.

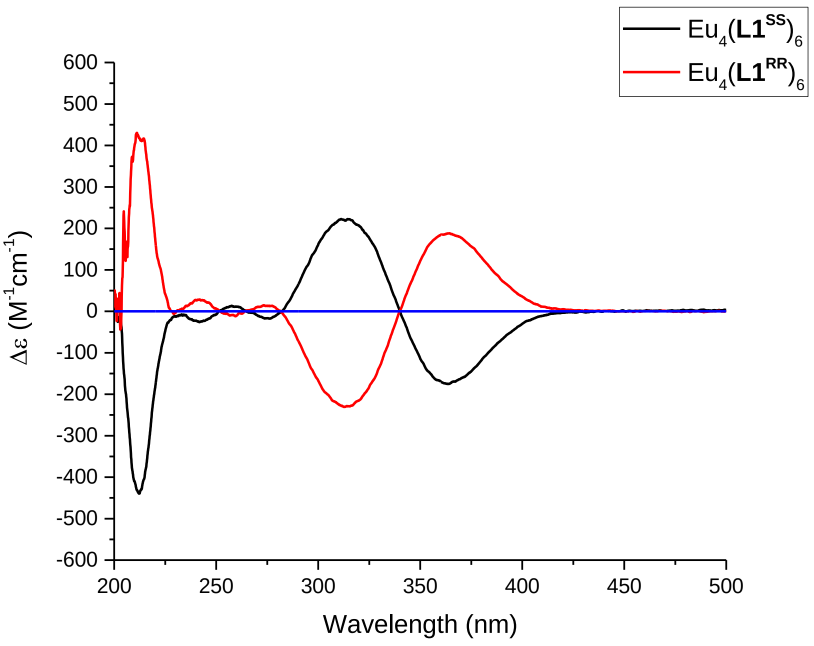

Supplementary Figure 31. CD spectra of $[Eu_4(\mathbf{L1}^{SS})_6](OTf)_{12}$ and $[Eu_4(\mathbf{L1}^{RR})_6](OTf)_{12}$ in CH_3CN.

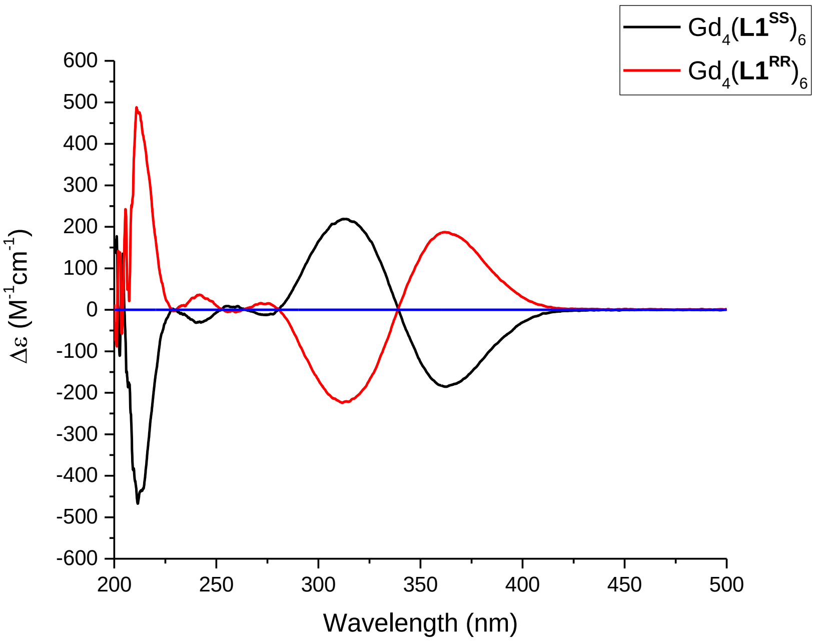

Supplementary Figure 32. CD spectra of $[Gd_4(\mathbf{L1}^{SS})_6](OTf)_{12}$ and $[Gd_4(\mathbf{L1}^{RR})_6](OTf)_{12}$ in CH_3CN.

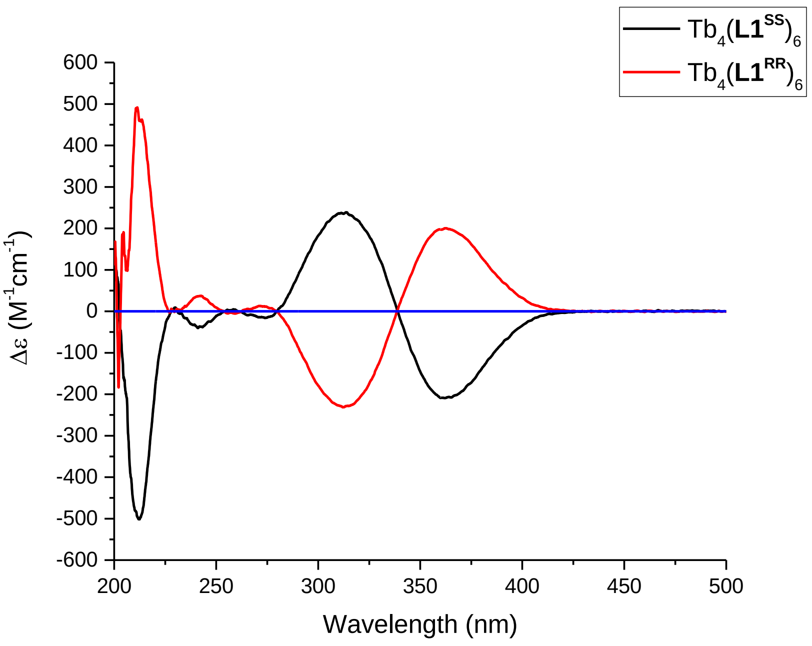

Supplementary Figure 33. CD spectra of $[Tb_4(\mathbf{L1}^{SS})_6](OTf)_{12}$ and $[Tb_4(\mathbf{L1}^{RR})_6](OTf)_{12}$ in CH_3CN.

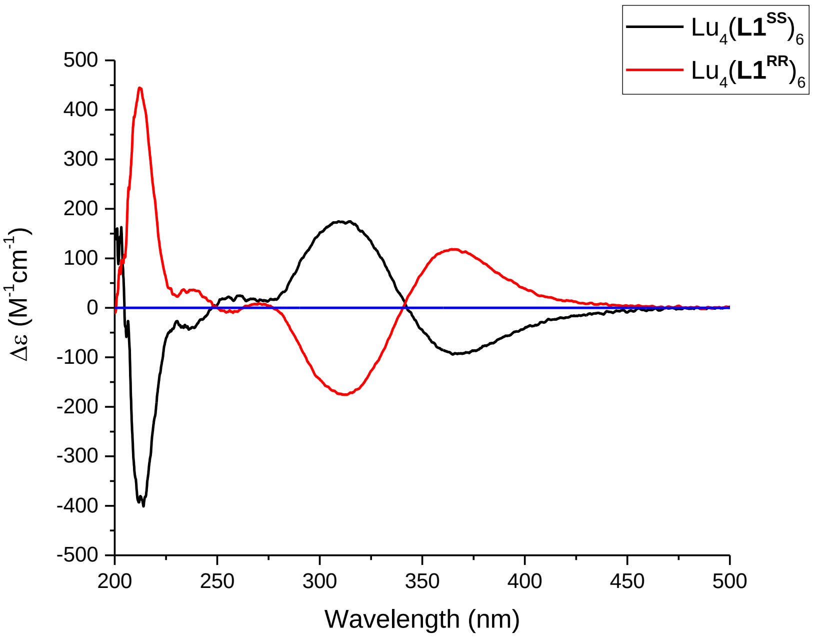

Supplementary Figure 34. CD spectra of $[Lu_4(\mathbf{L1}^{SS})_6](OTf)_{12}$ and $[Lu_4(\mathbf{L1}^{RR})_6](OTf)_{12}$ in CH_3CN.

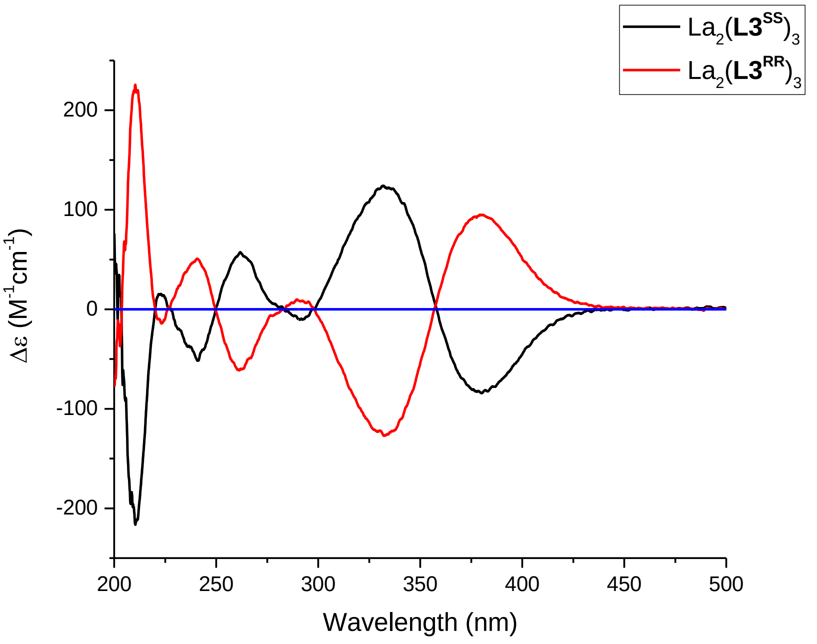

Supplementary Figure 35. CD spectra of $[La_2(\mathbf{L3}^{SS})_3](OTf)_6$ and $[La_2(\mathbf{L3}^{RR})_3](OTf)_6$ in CH_3CN.

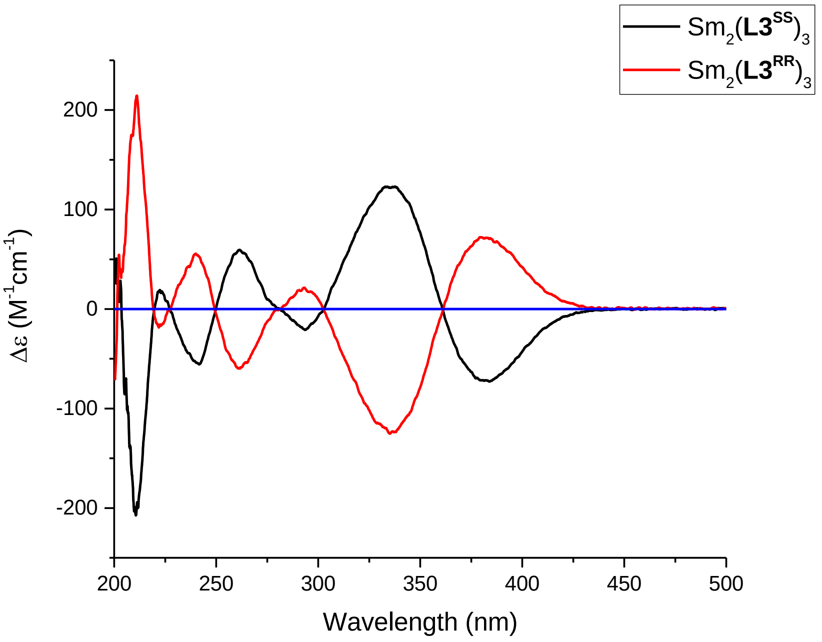

Supplementary Figure 36. CD spectra of $[Sm_2(\mathbf{L3}^{SS})_3](OTf)_6$ and $[Sm_2(\mathbf{L3}^{RR})_3](OTf)_6$ in CH_3CN.

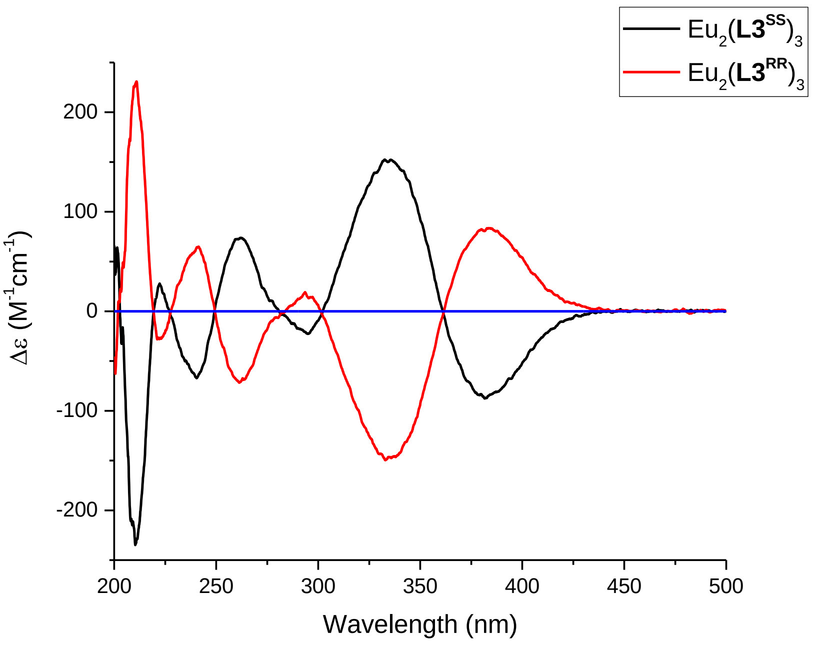

Supplementary Figure 37. CD spectra of [Eu_2(**L3**^{SS})$_3$](OTf)$_6$ and [Eu_2(**L3**^{RR})$_3$](OTf)$_6$ in CH_3CN.

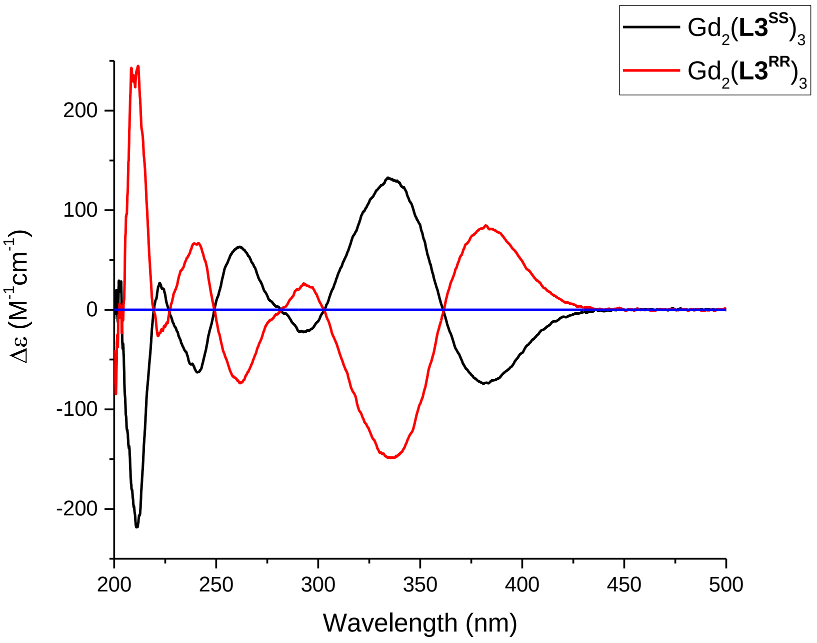

Supplementary Figure 38. CD spectra of [Gd_2(**L3**^{SS})$_3$](OTf)$_6$ and [Gd_2(**L3**^{RR})$_3$](OTf)$_6$ in CH_3CN.

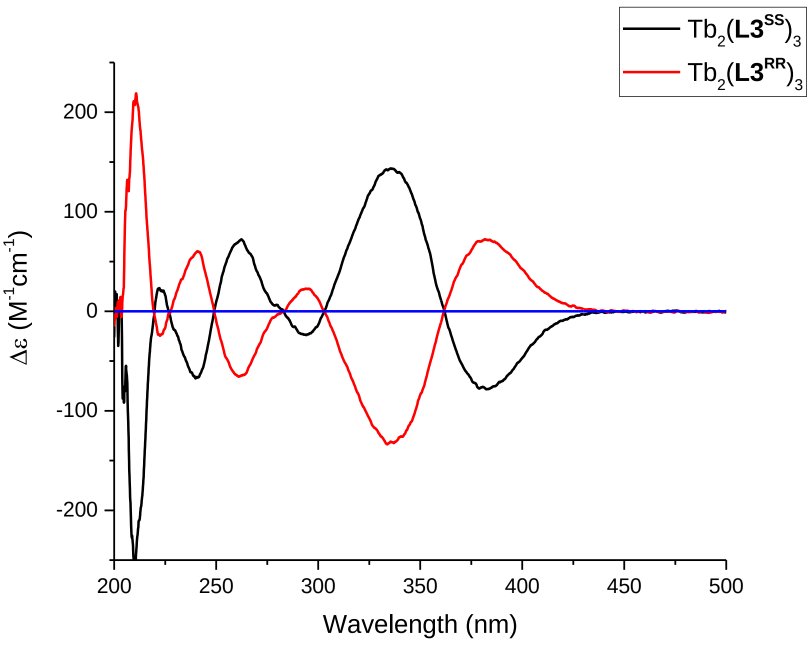

Supplementary Figure 39. CD spectra of $[Tb_2(\mathbf{L3}^{SS})_3](OTf)_6$ and $[Tb_2(\mathbf{L3}^{RR})_3](OTf)_6$ in CH_3CN.

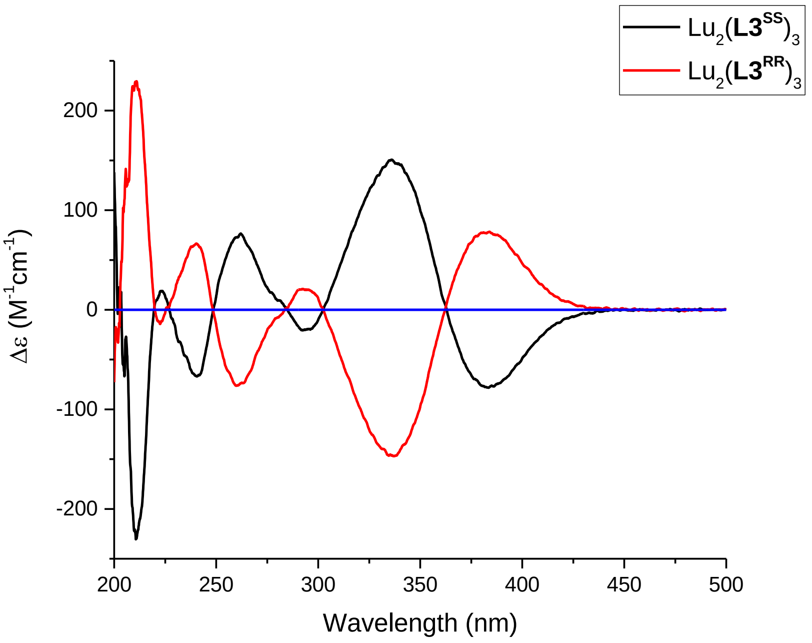

Supplementary Figure 40. CD spectra of $[Lu_2(\mathbf{L3}^{SS})_3](OTf)_6$ and $[Lu_2(\mathbf{L3}^{RR})_3](OTf)_6$ in CH_3CN.

NMR characterization

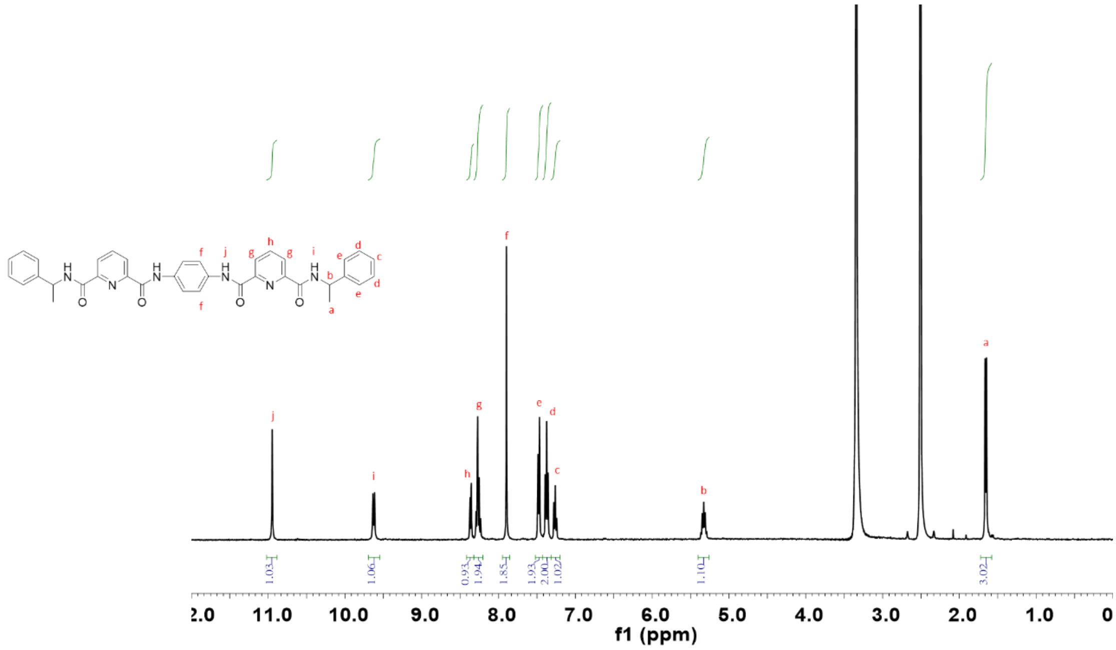

Supplementary Figure 41. ^{1}H NMR of **L1^{SS}** in $(CD_3)_2SO$.

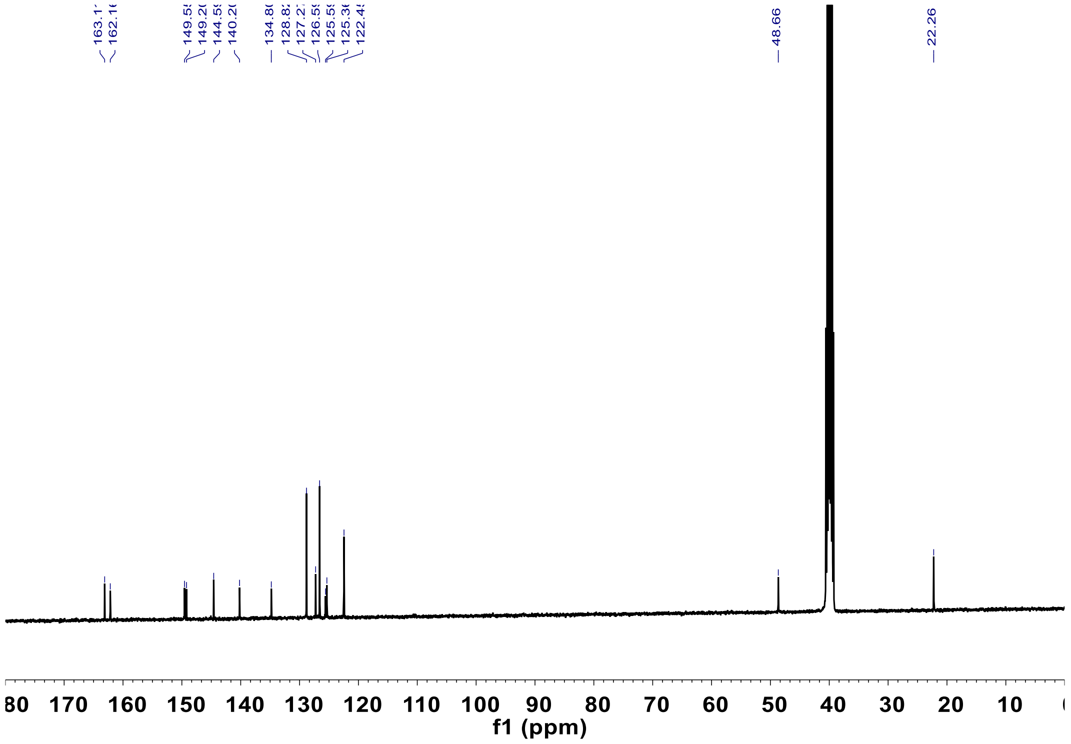

Supplementary Figure 42. ^{13}C NMR of **L1^{SS}** in $(CD_3)_2SO$.

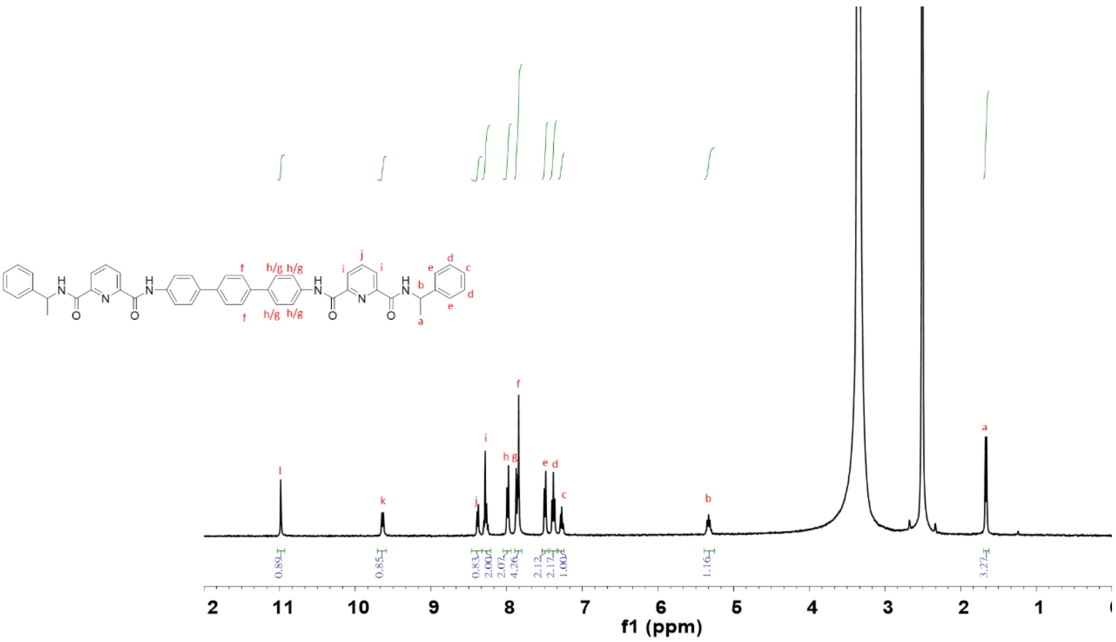

Supplementary Figure 43. ^{1}H NMR of **L3^{RR}** in $(CD_3)_2SO$.

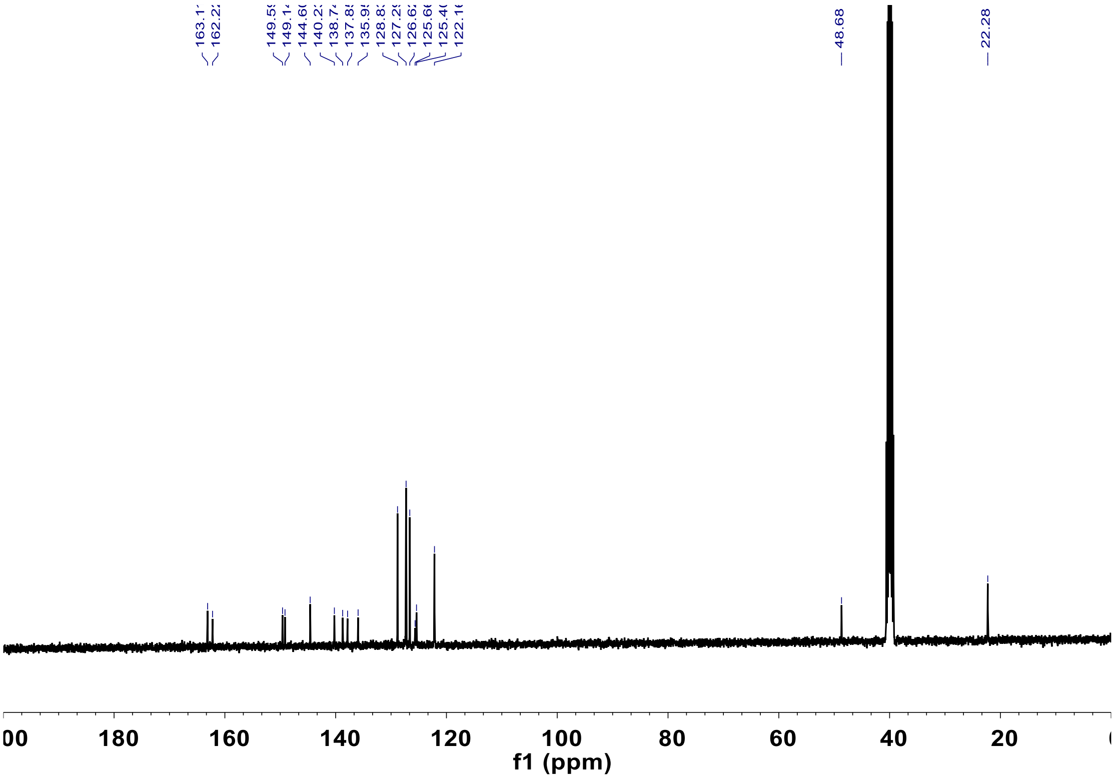

Supplementary Figure 44. ^{13}C NMR of **L3^{RR}** in $(CD_3)_2SO$.

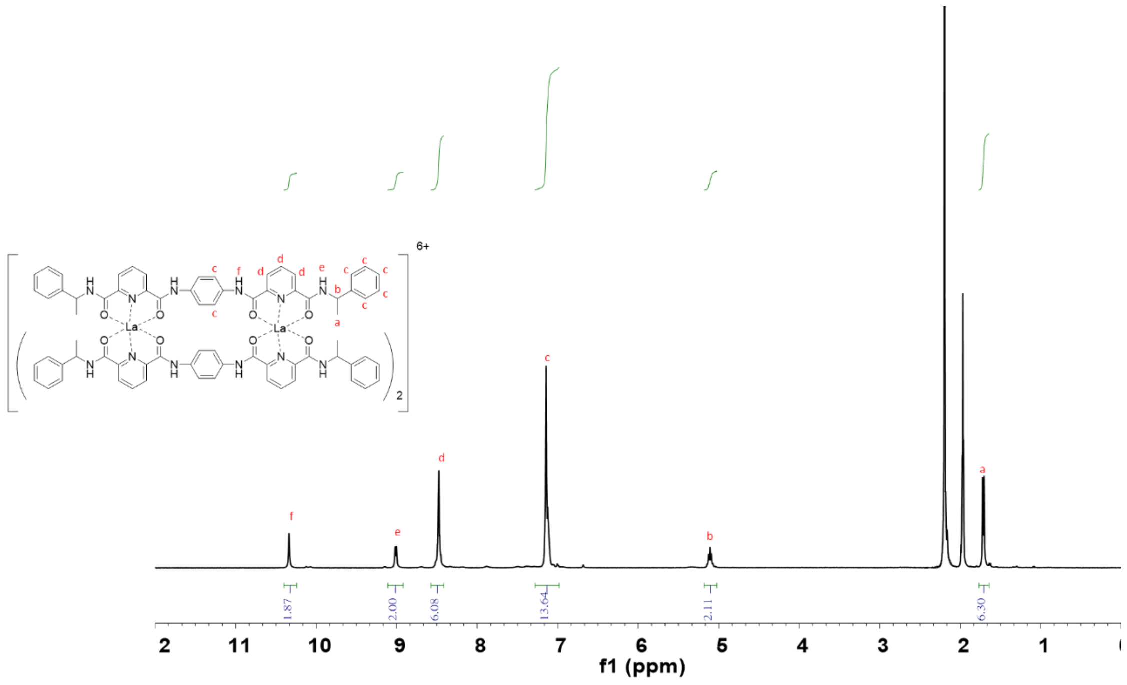

Supplementary Figure 45. ^{1}H NMR of [La_2(**L1**^{SS})$_3$] in CD_3CN.

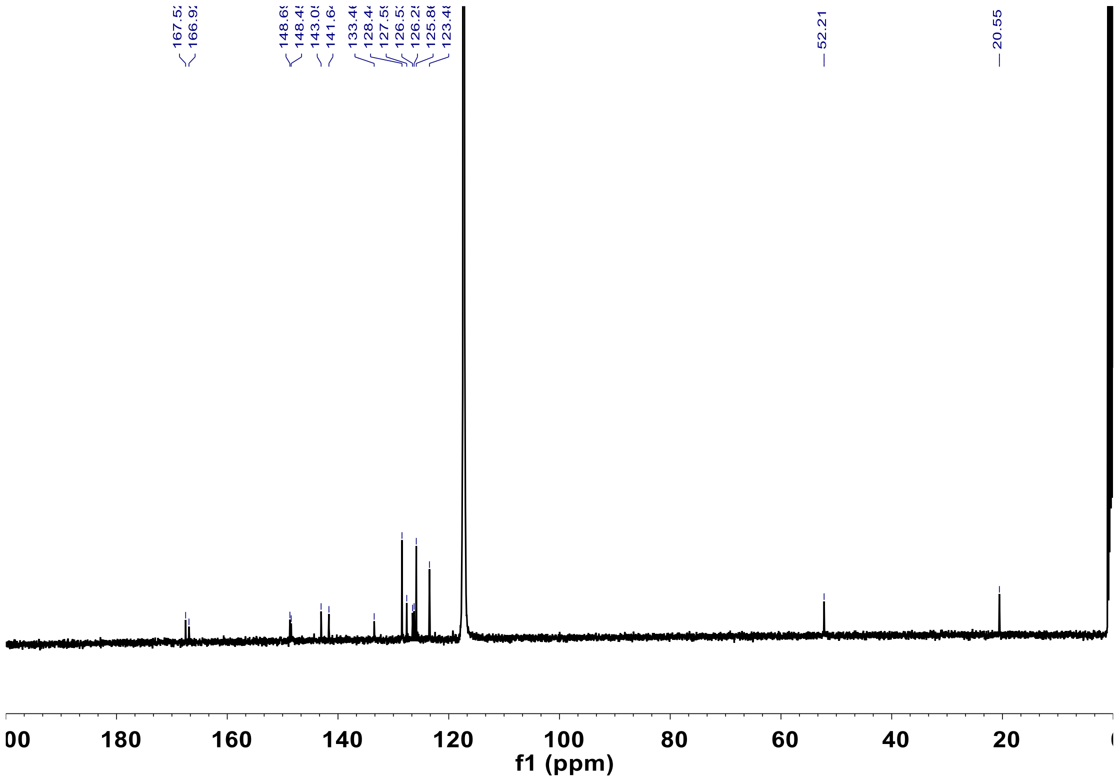

Supplementary Figure 46. ^{13}C NMR of [La_2(**L1**^{SS})$_3$] in CD_3CN.

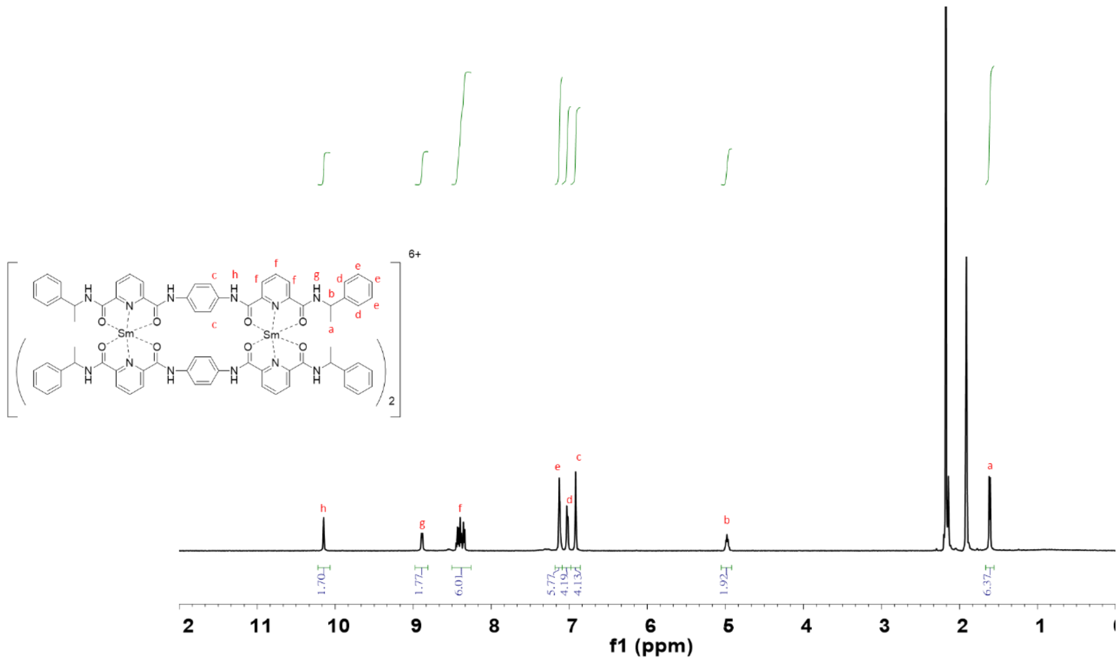

Supplementary Figure 47. ^{1}H NMR of $[Sm_2(\mathbf{L1^{RR}})_3]$ in CD_3CN.

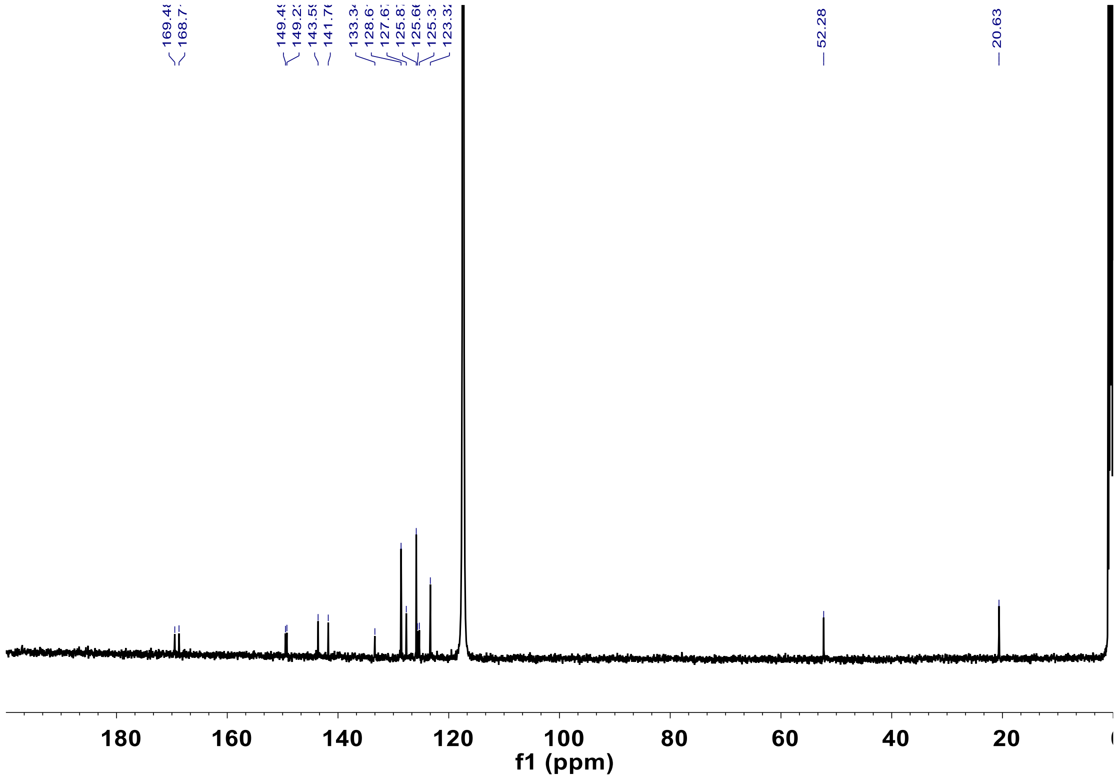

Supplementary Figure 48. ^{13}C NMR of $[Sm_2(\mathbf{L1^{RR}})_3]$ in CD_3CN.

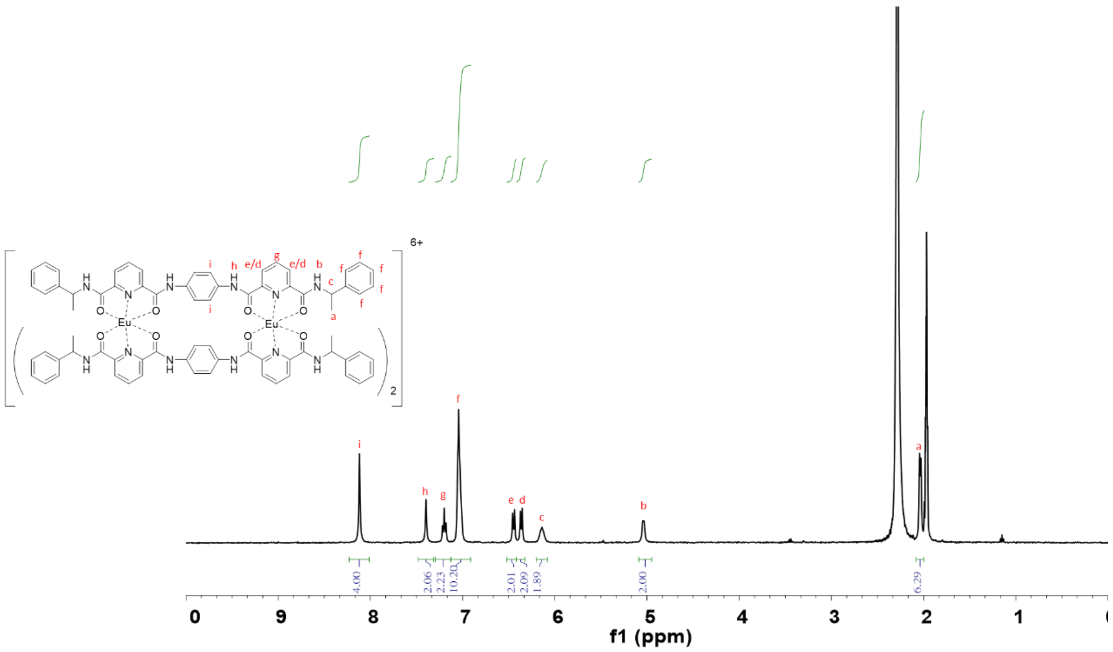

Supplementary Figure 49. ^{1}H NMR of $[Eu_2(\mathbf{L1^{SS}})_3]$ in CD_3CN.

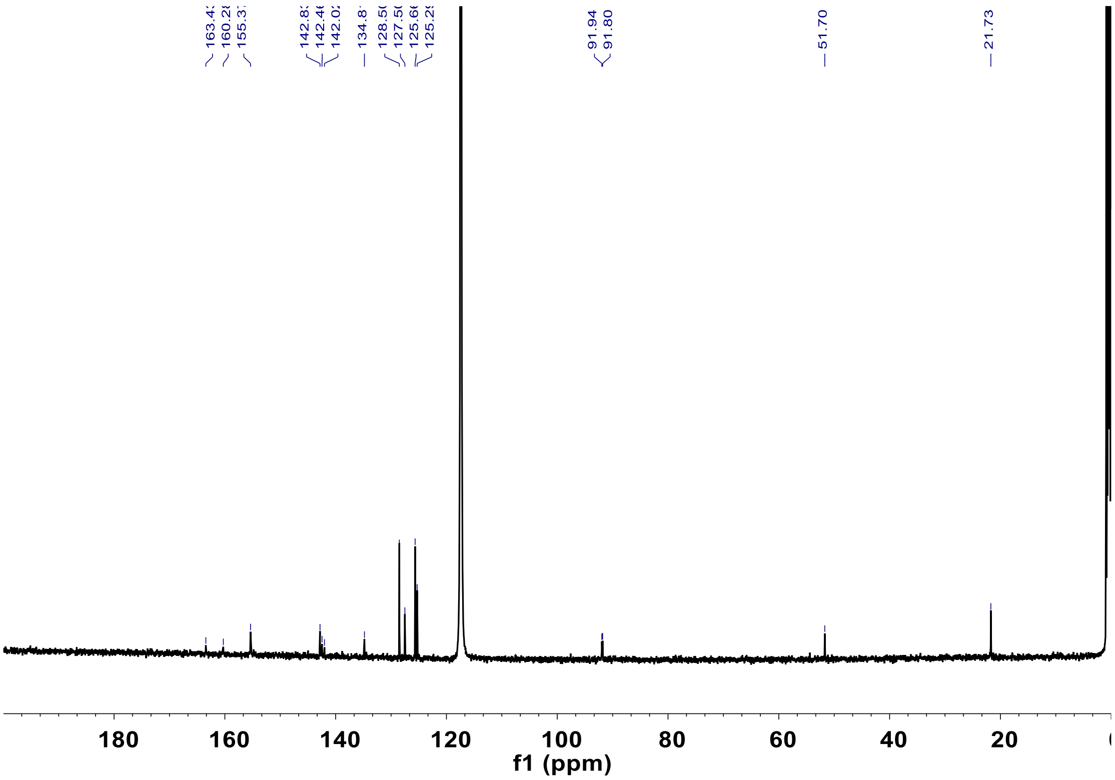

Supplementary Figure 50. ^{13}C NMR of $[Eu_2(\mathbf{L1^{RR}})_3]$ in CD_3CN.

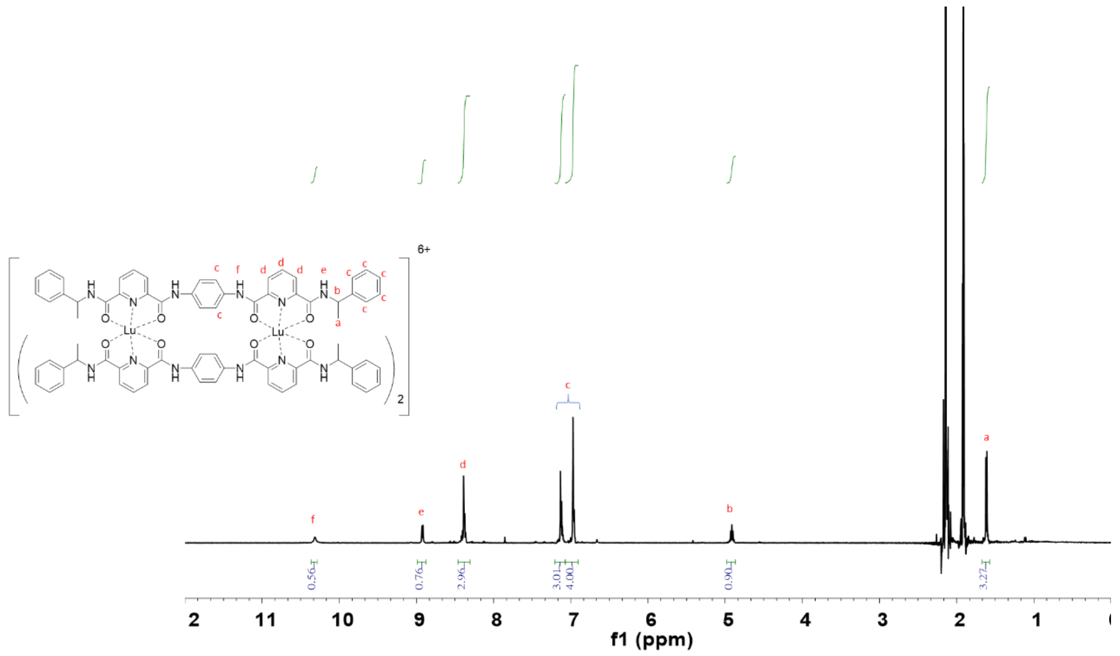

Supplementary Figure 51. ^{1}H NMR of [Lu_2(**L1^{RR}**)$_3$] in CD_3CN.

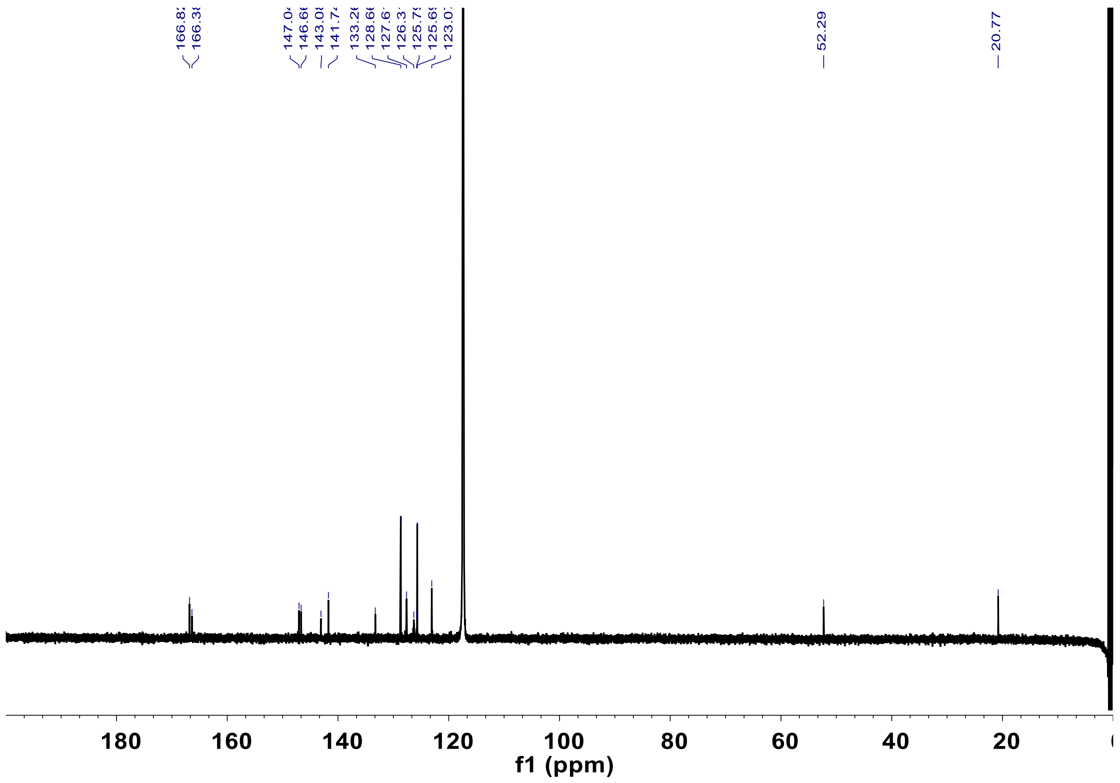

Supplementary Figure 52. ^{13}C NMR of [Lu_2(**L1^{SS}**)$_3$] in CD_3CN.

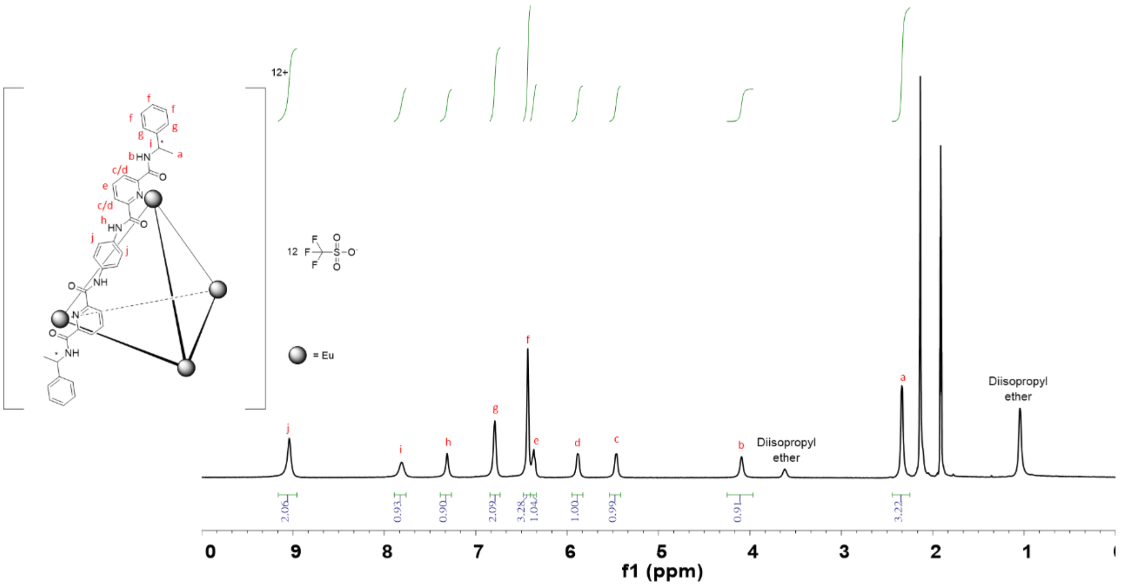

Supplementary Figure 53. ^{1}H NMR of [Eu_4(**L1**^{SS})$_6$] in CD_3CN.

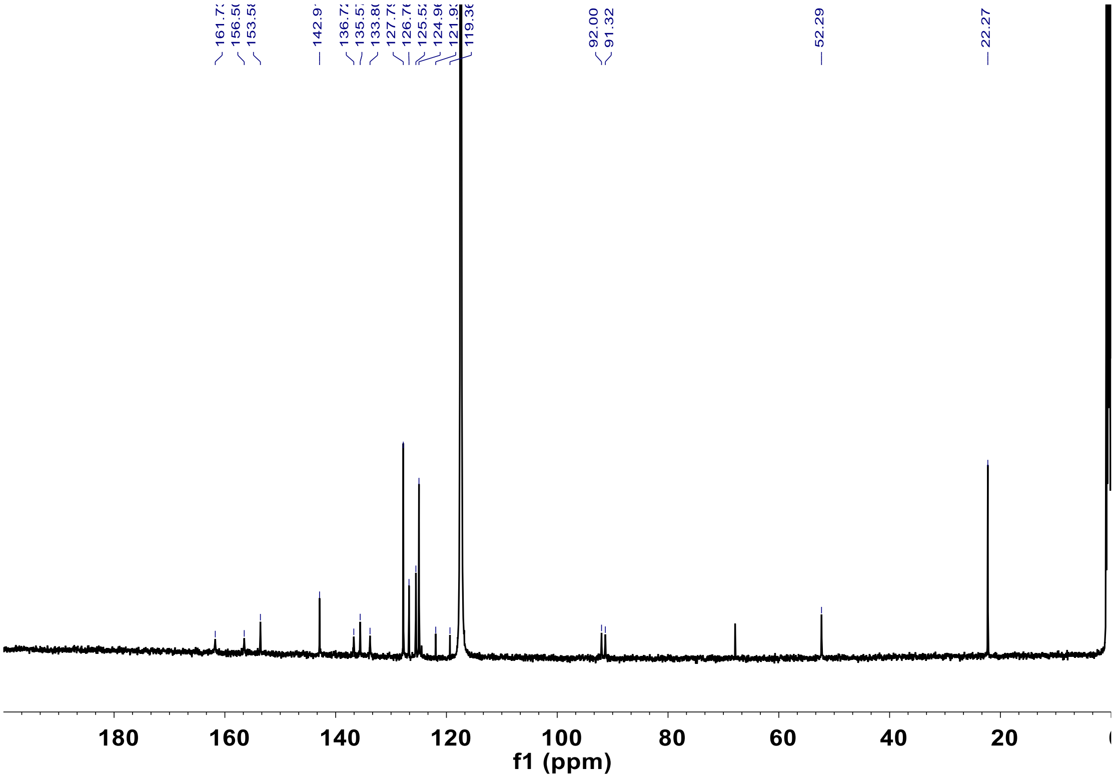

Supplementary Figure 54. ^{13}C NMR of [Eu_4(**L1**^{SS})$_6$] in CD_3CN.

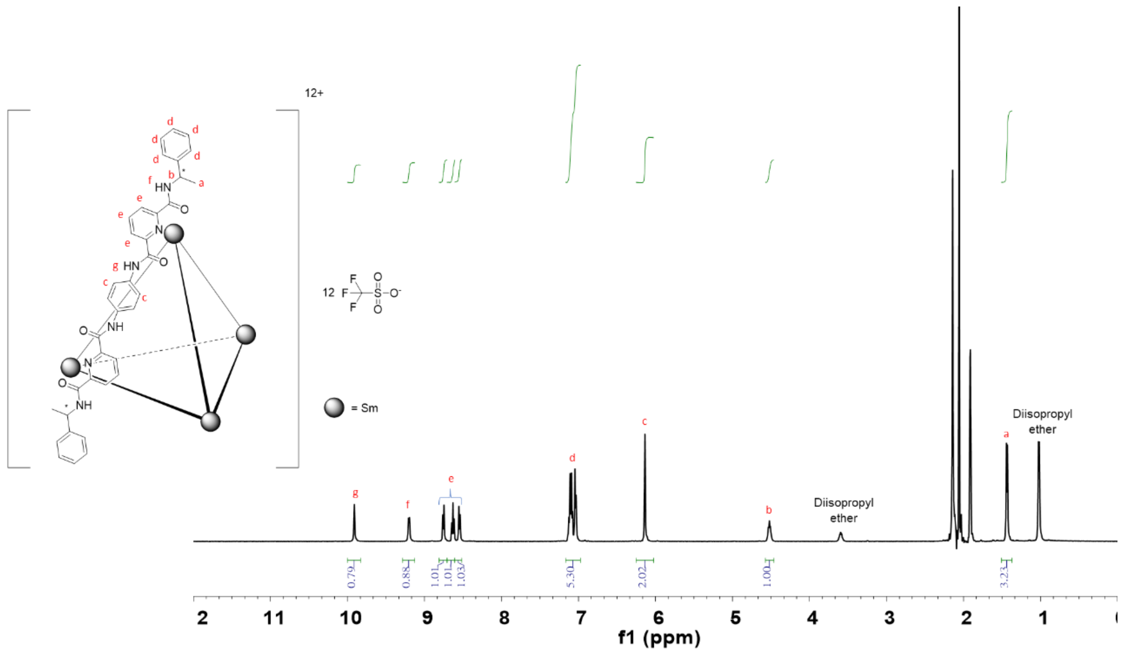

Supplementary Figure 55. ^{1}H NMR of [Sm_4(**L1^{SS}**)$_6$] in CD_3CN.

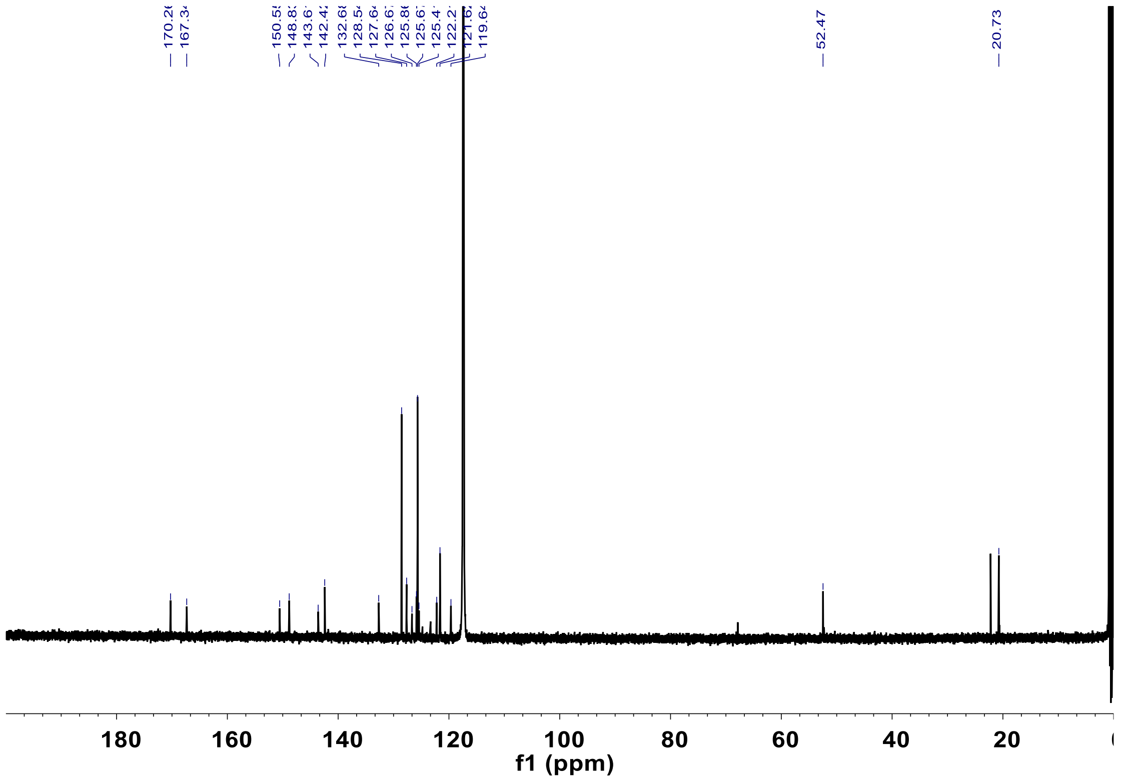

Supplementary Figure 56. ^{13}C NMR of [Sm_4(**L1^{RR}**)$_6$] in CD_3CN.

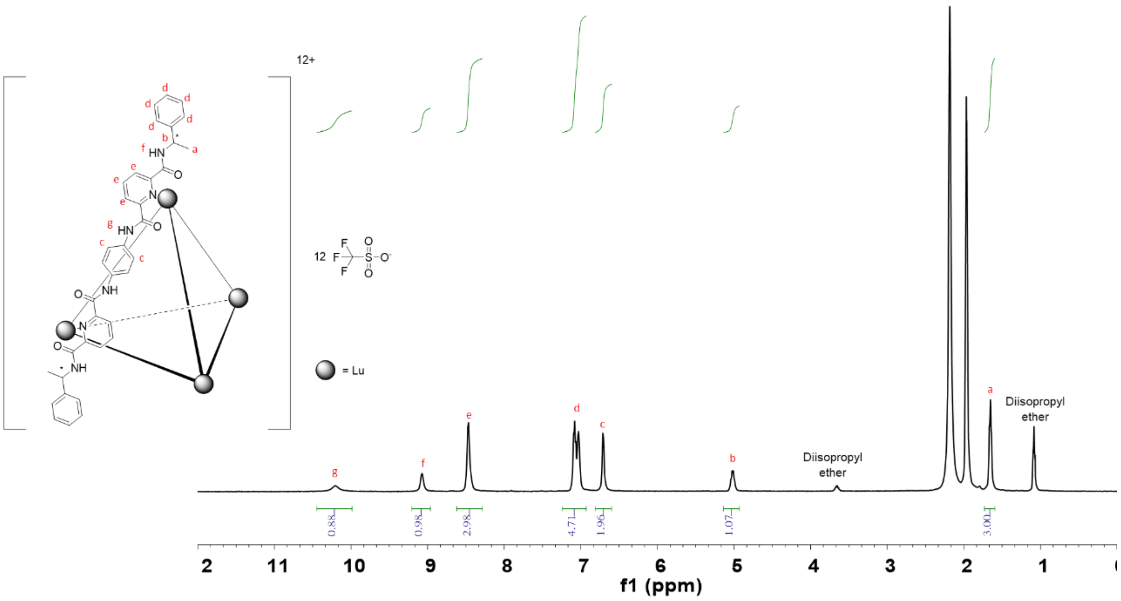

Supplementary Figure 57. ^{1}H NMR of [Lu_4(**L1**^{SS})$_6$] in CD_3CN.

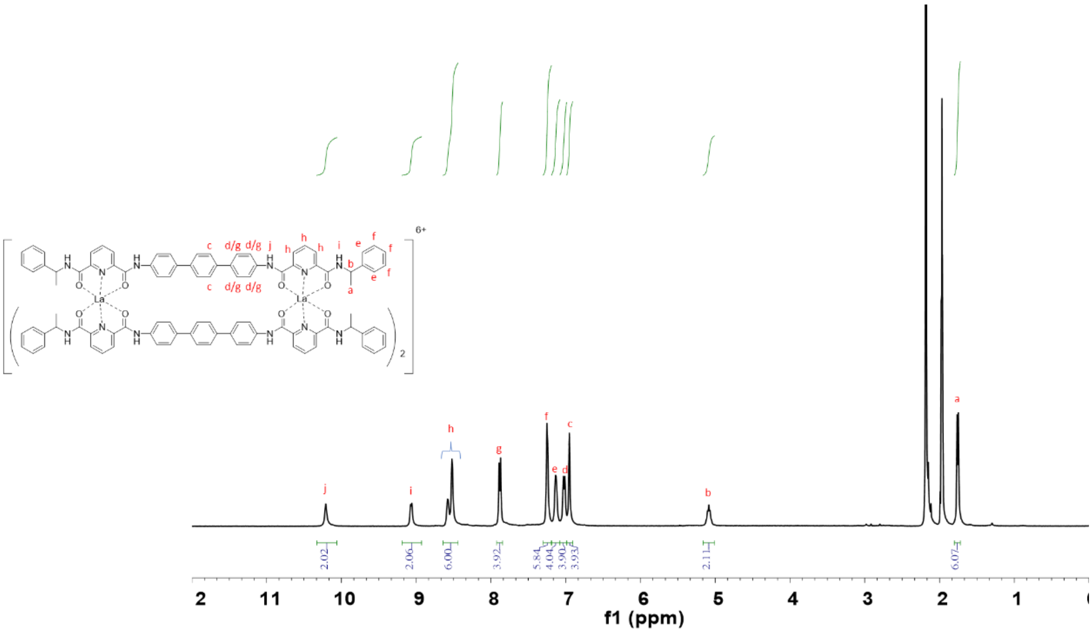

Supplementary Figure 58. ^{1}H NMR of [La_2(**L3**^{SS})$_3$] in CD_3CN.

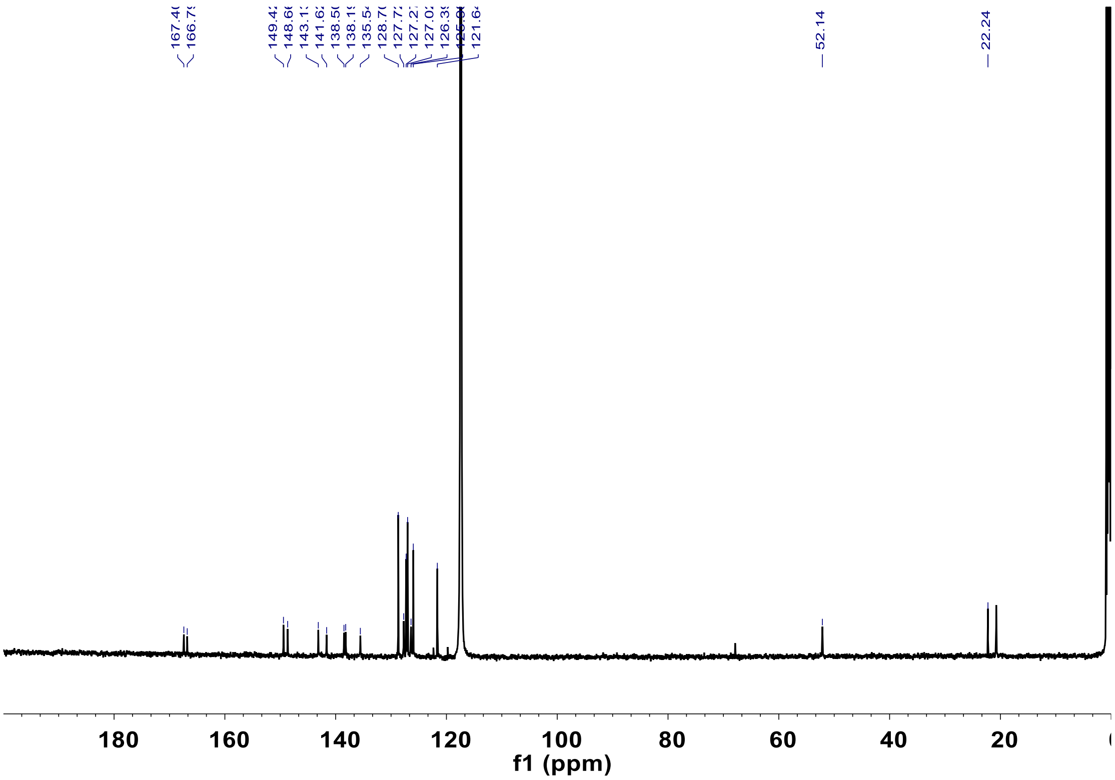

Supplementary Figure 59. ^{13}C NMR of [La_2(**L3^{RR}**)$_3$] in CD_3CN.

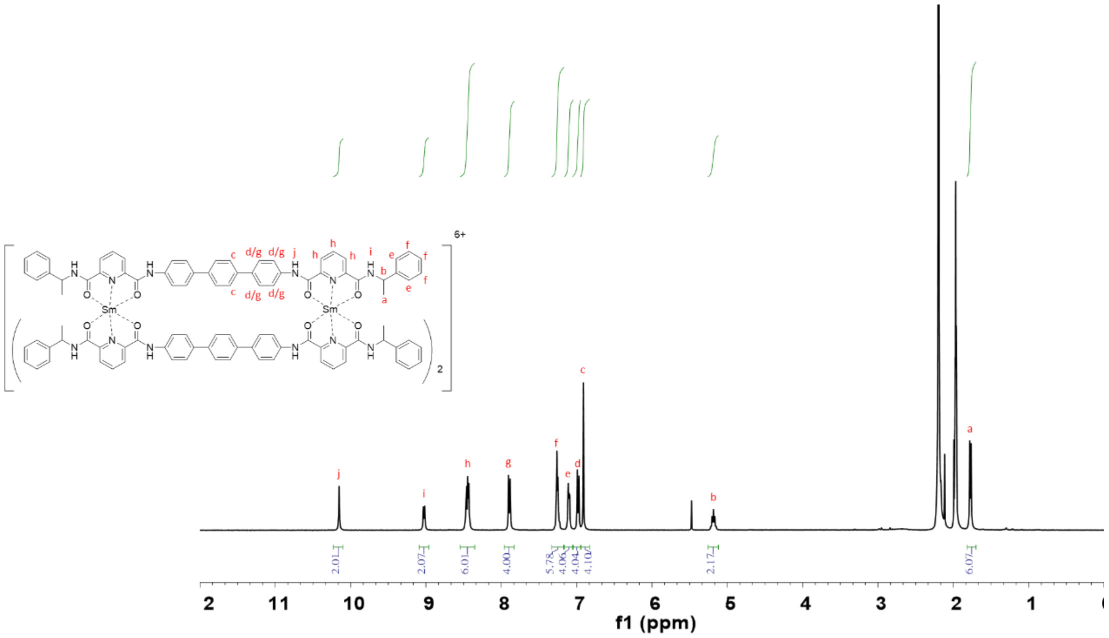

Supplementary Figure 60. ^{1}H NMR of [Sm_2(**L3^{SS}**)$_3$] in CD_3CN.

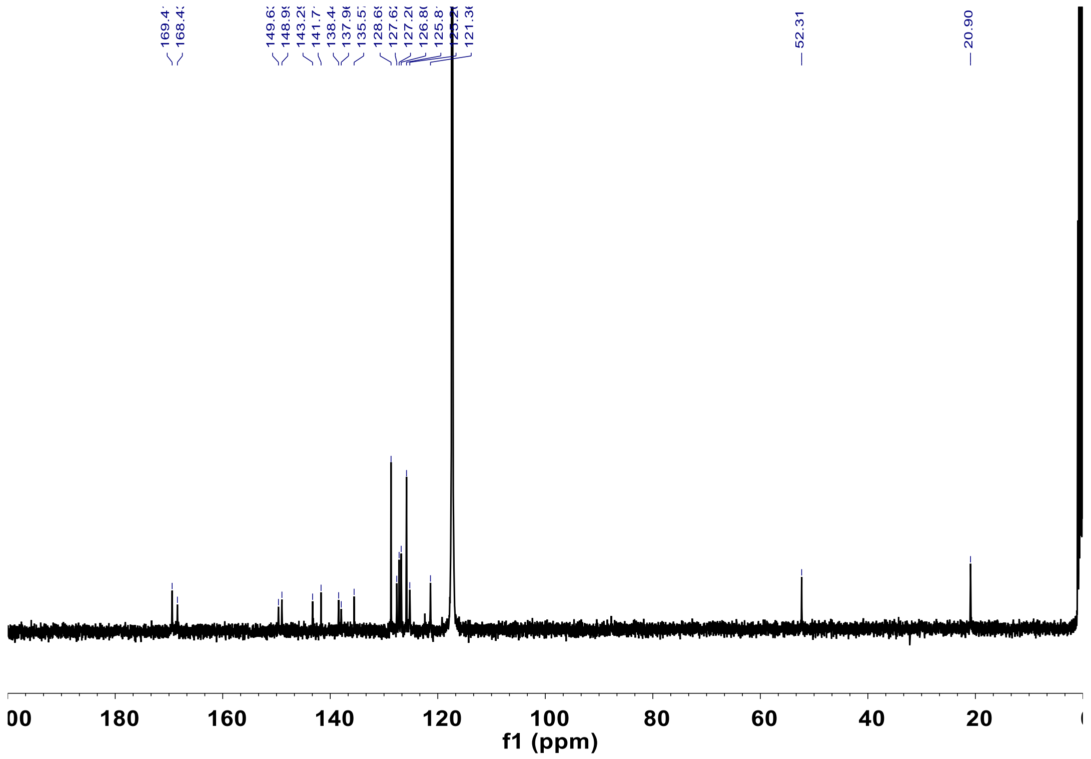

Supplementary Figure 61. ^{13}C NMR of [Sm_2(**L3^{SS}**)$_3$] in CD_3CN.

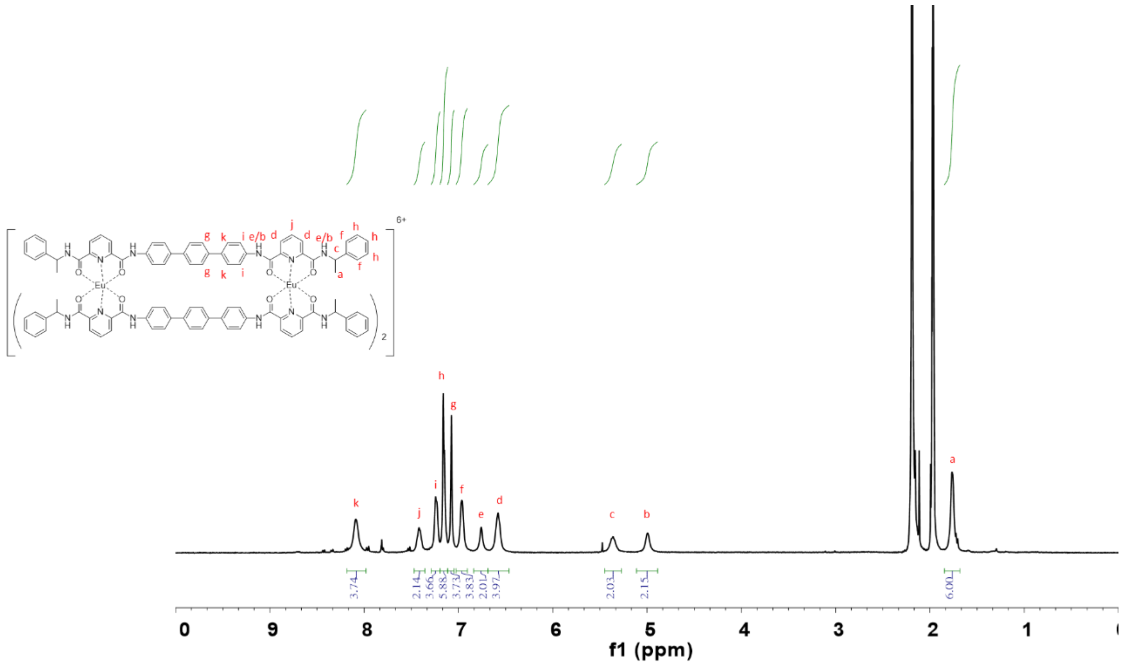

Supplementary Figure 62. ^{1}H NMR of [Eu_2(**L3^{SS}**)$_3$] in CD_3CN.

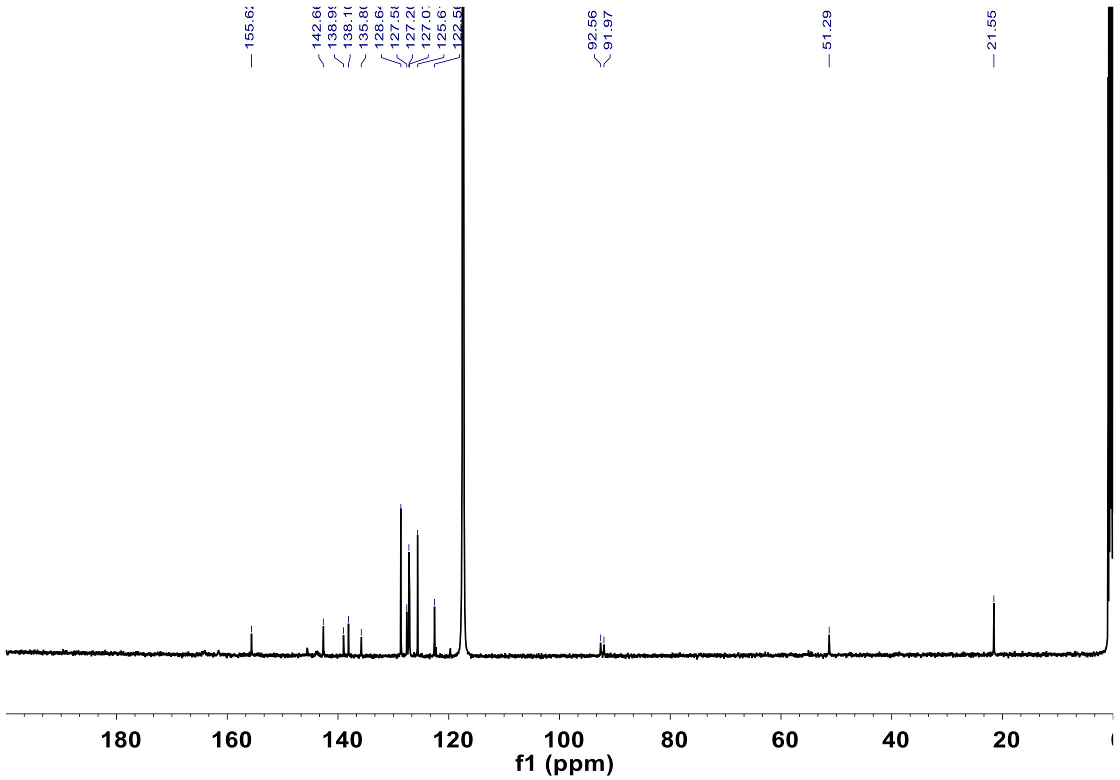

Supplementary Figure 63. ^{13}C NMR of [Eu_2(**L3^{RR}**)$_3$] in CD_3CN.

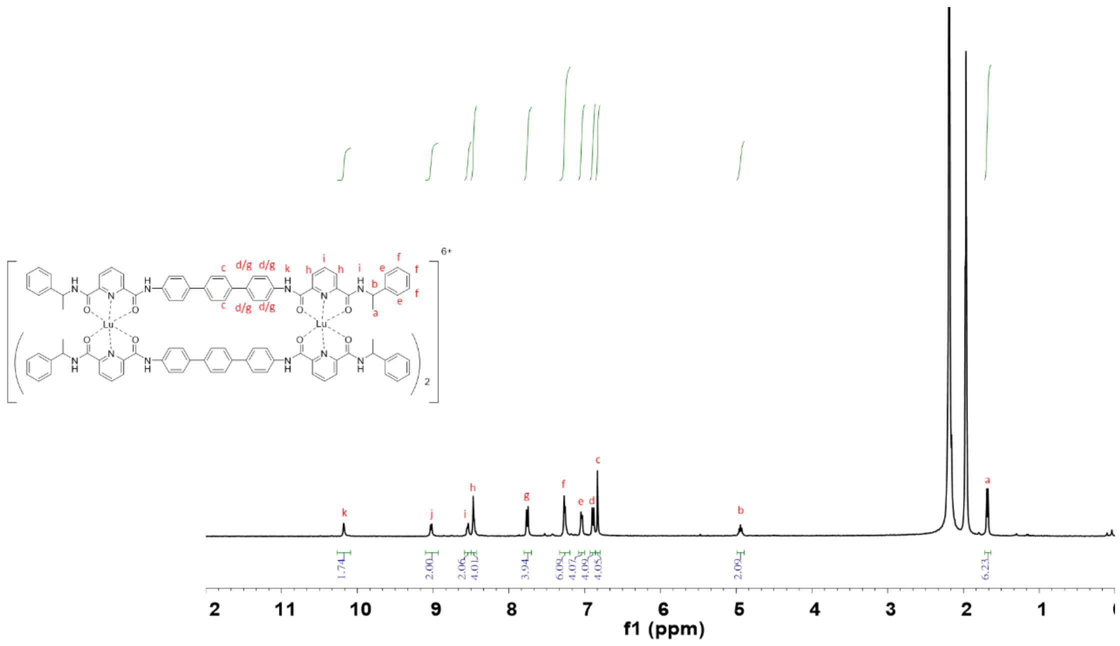

Supplementary Figure 64. ^{1}H NMR of [Lu_2(**L3^{RR}**)$_3$] in CD_3CN.

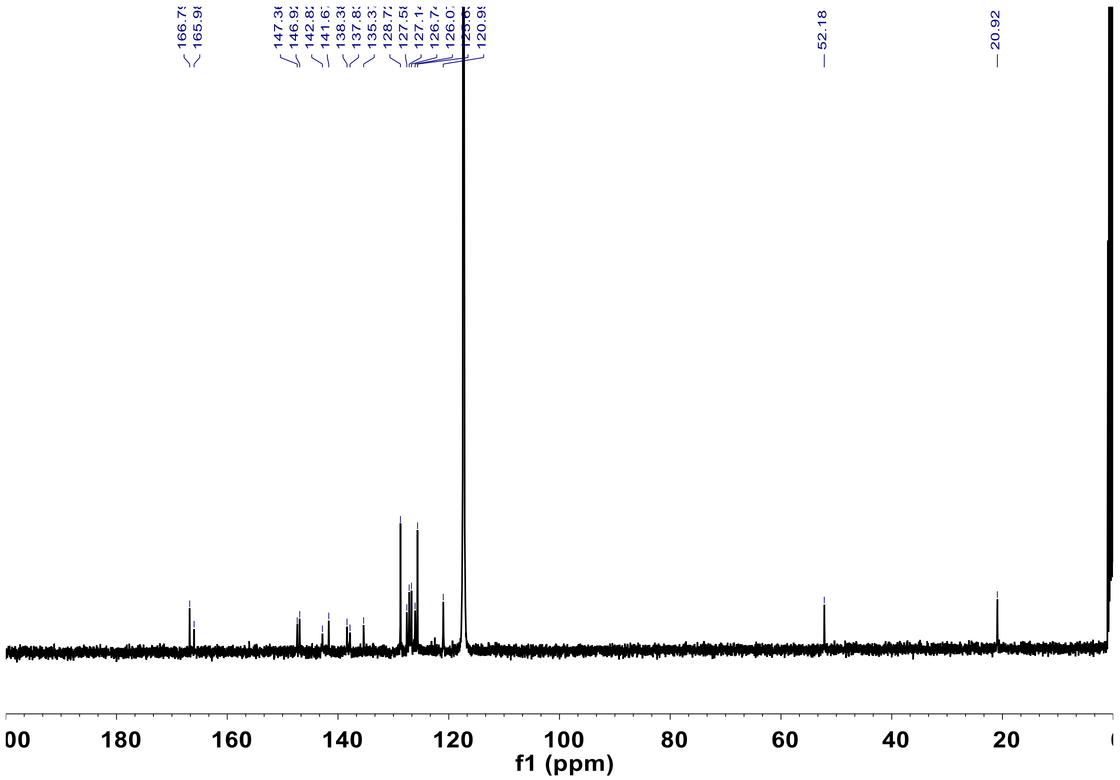

Supplementary Figure 65. ^{13}C NMR of [Lu_2(**L3**^{RR})$_3$] in CD_3CN.

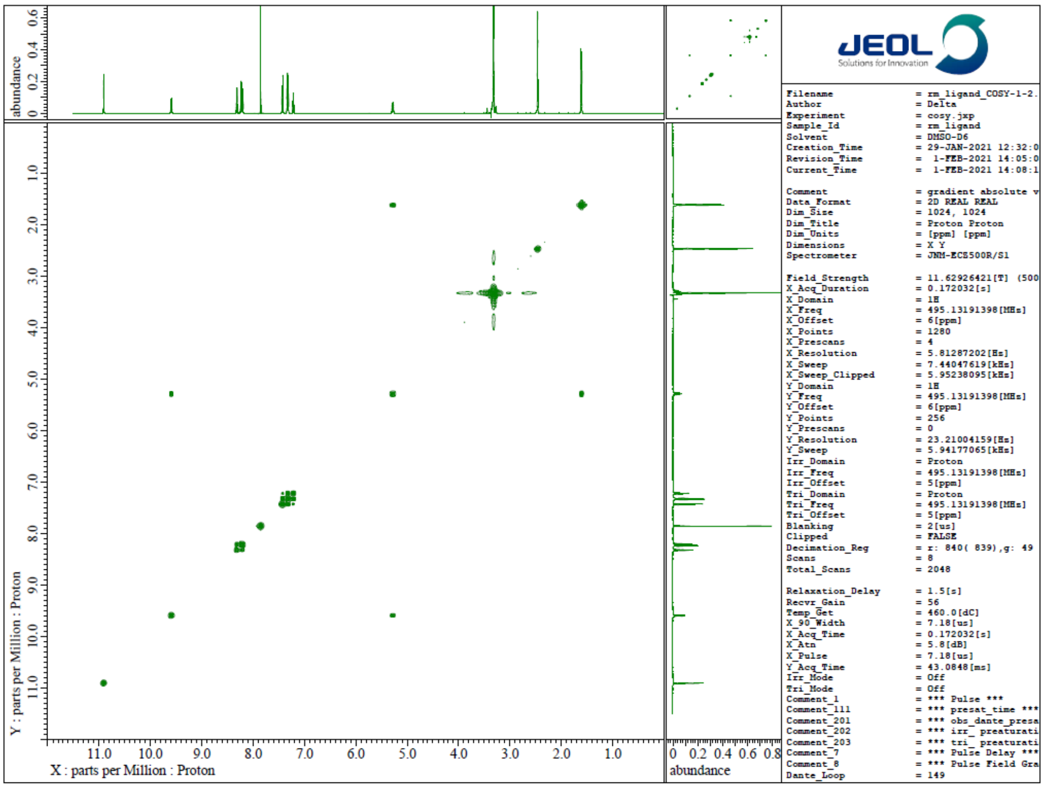

Supplementary Figure 66. COSY NMR of **L1** in $(CD_3)_2SO$.

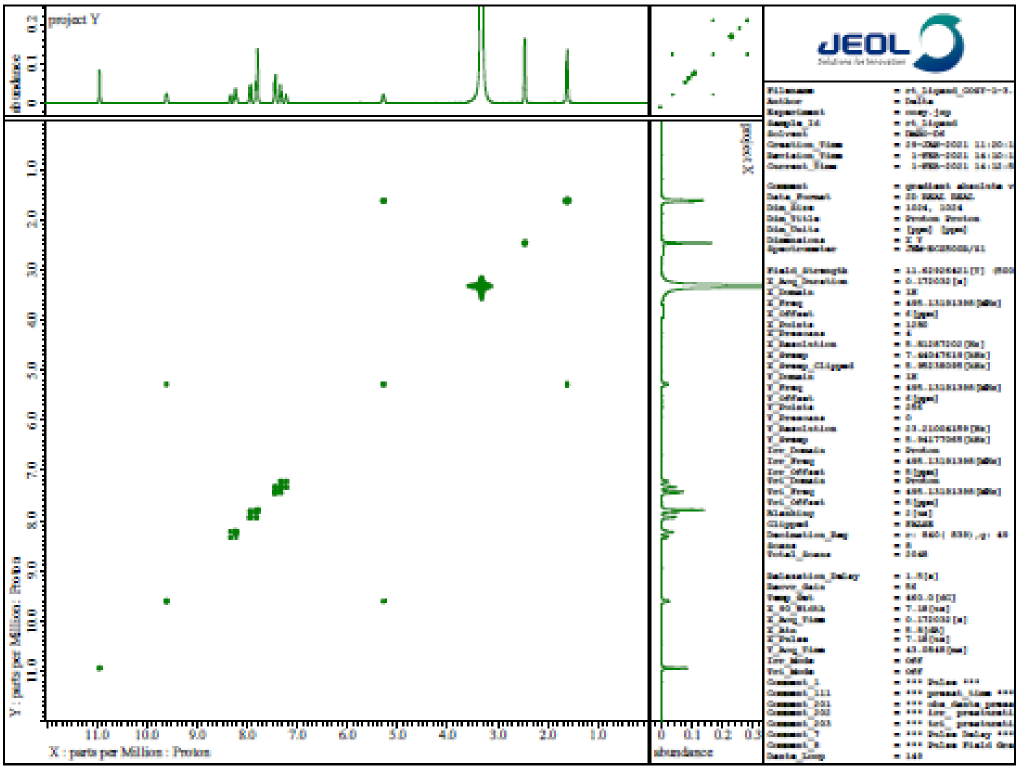

Supplementary Figure 67. COSY NMR of **L3** in $(CD_3)_2SO$.

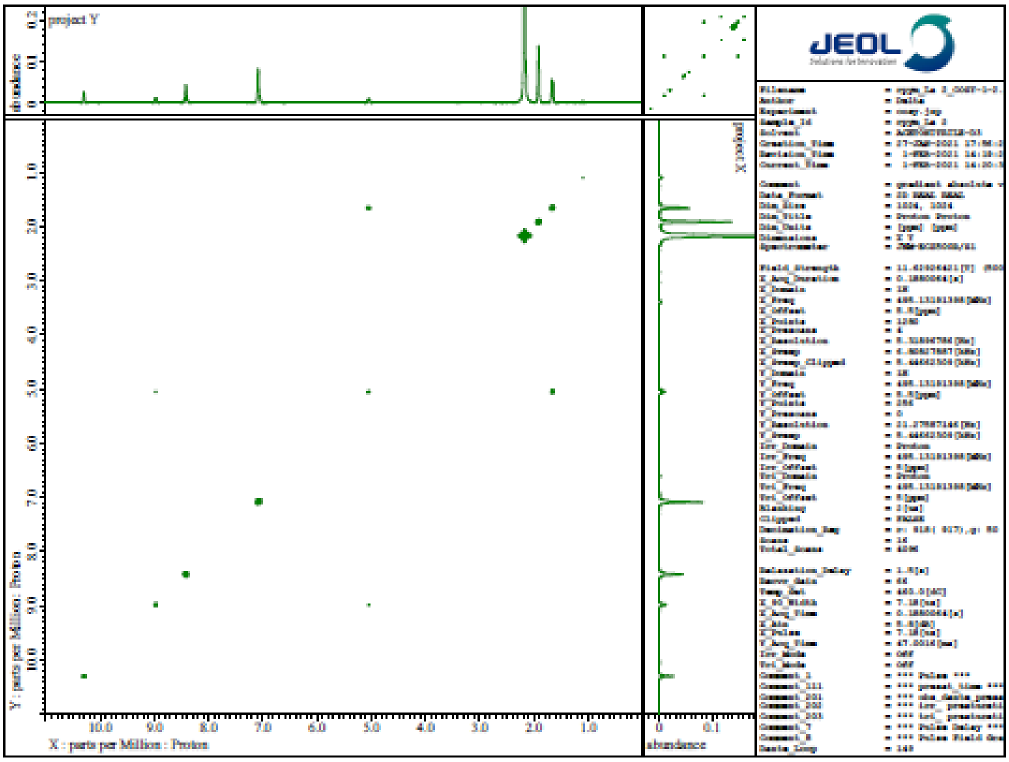

Supplementary Figure 68. COSY NMR of [La_2(**L1**^{RR})$_3$] in CD_3CN.

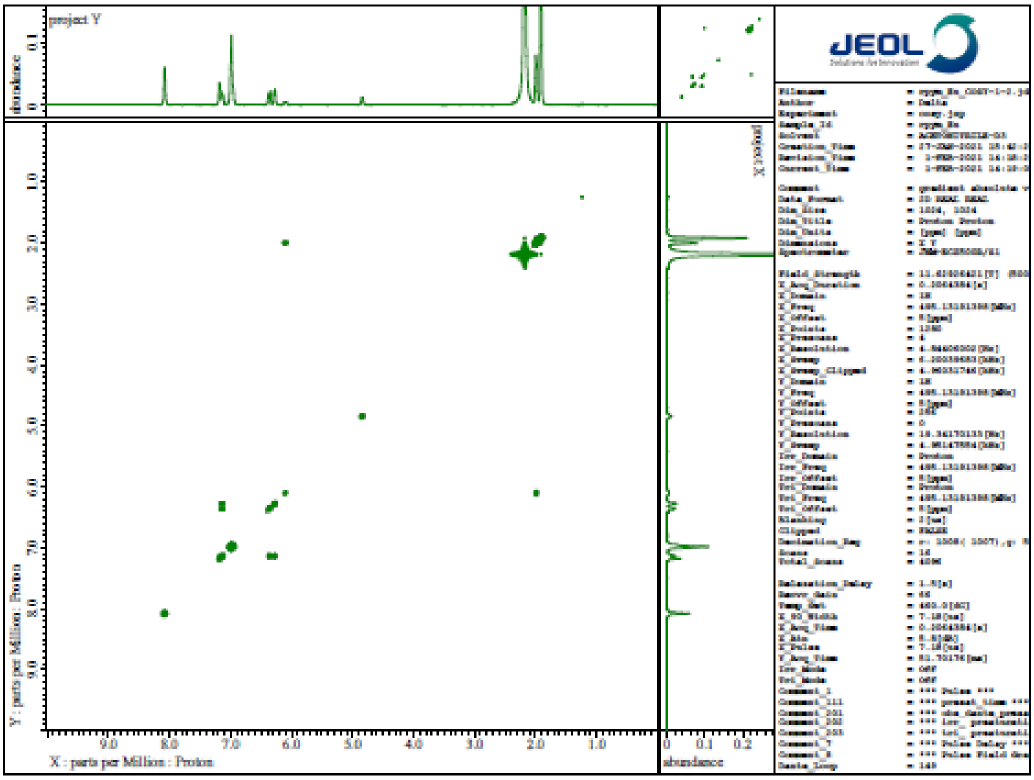

Supplementary Figure 69. COSY NMR of [Eu_2(**L1**^{RR})$_3$] in CD_3CN.

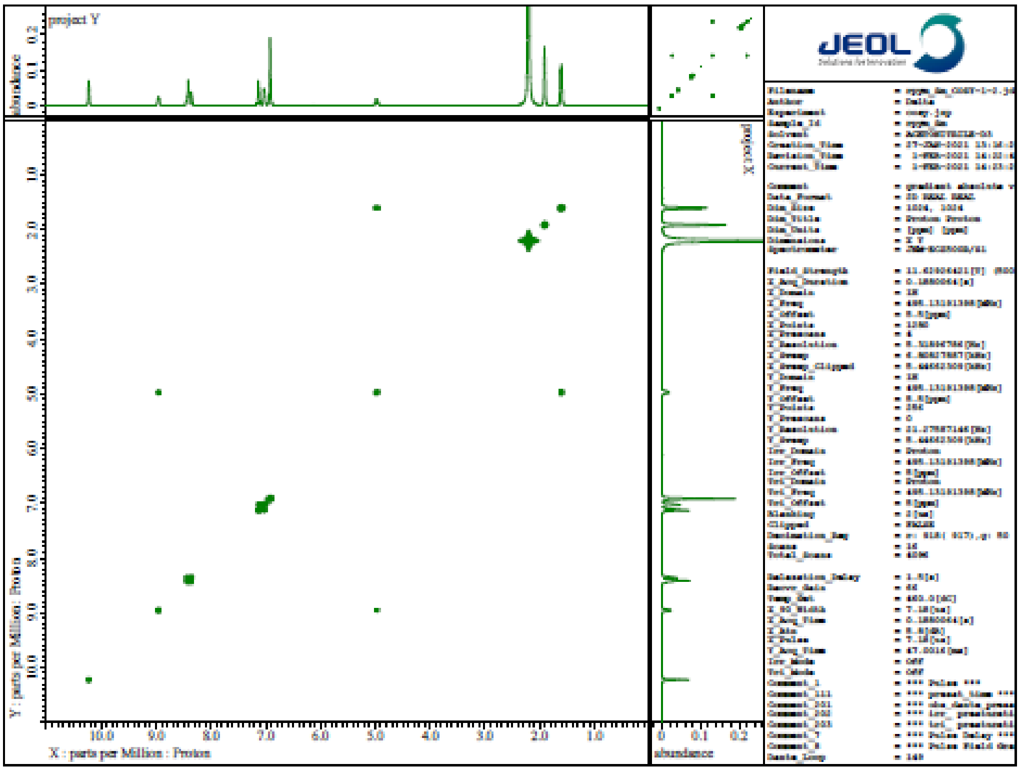

Supplementary Figure 70. COSY NMR of [Sm_2(**L1**^{RR})$_3$] in CD_3CN.

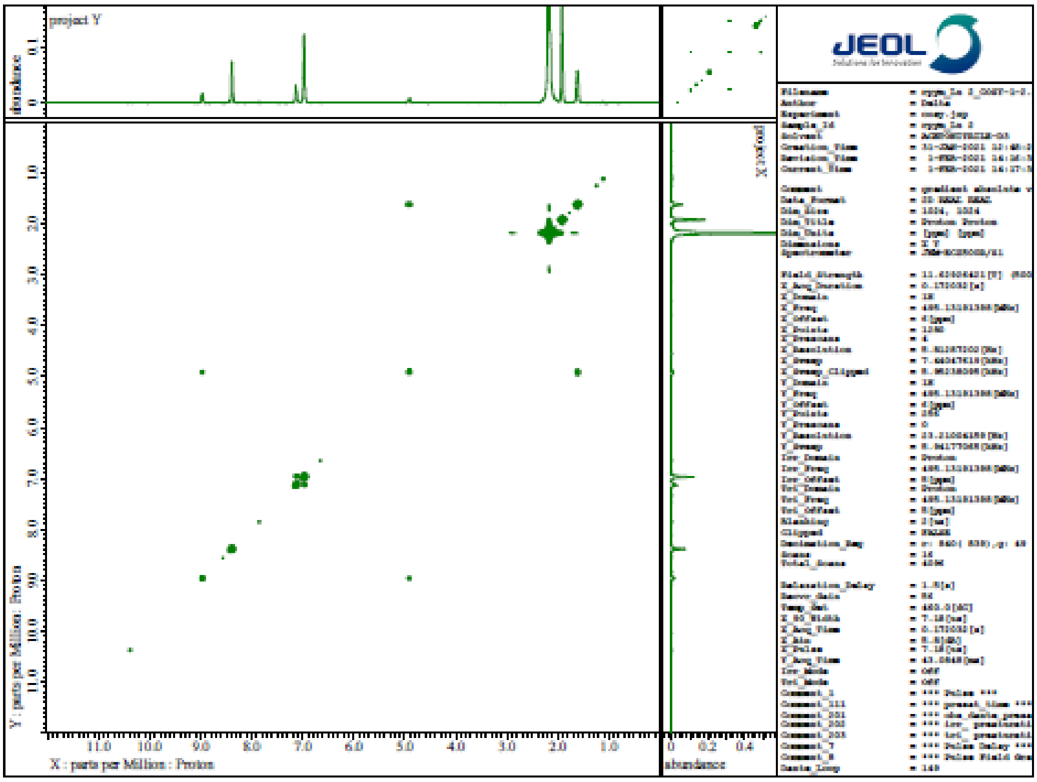

Supplementary Figure 71. COSY NMR of [Lu_2(**L1**^{RR})$_3$] in CD_3CN.

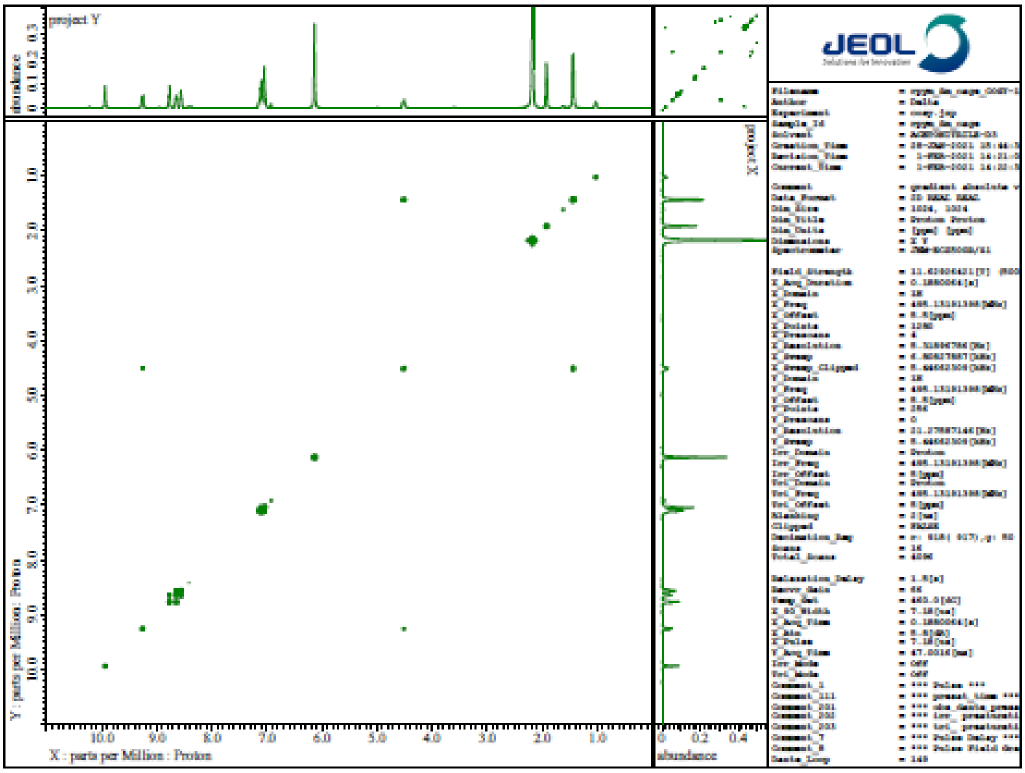

Supplementary Figure 72. COSY NMR of [Sm_4(**L1**^{RR})$_6$] in CD_3CN.

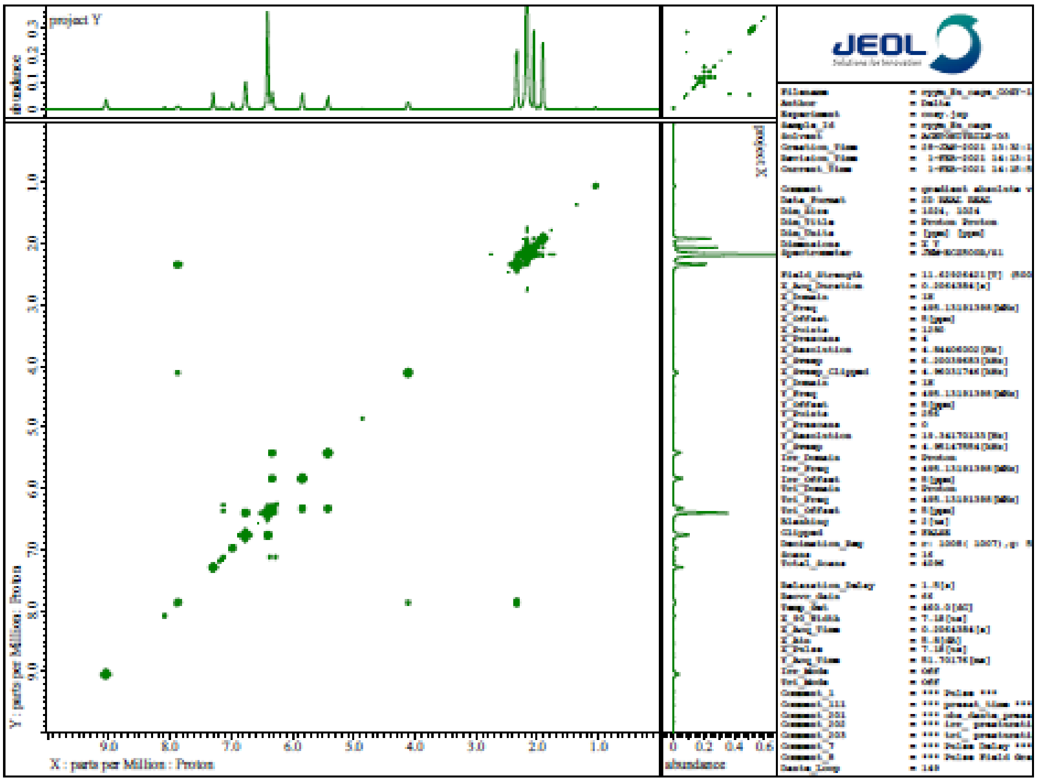

Supplementary Figure 73. COSY NMR of $[Eu_4(\mathbf{L1}^{RR})_6]$ in CD_3CN.

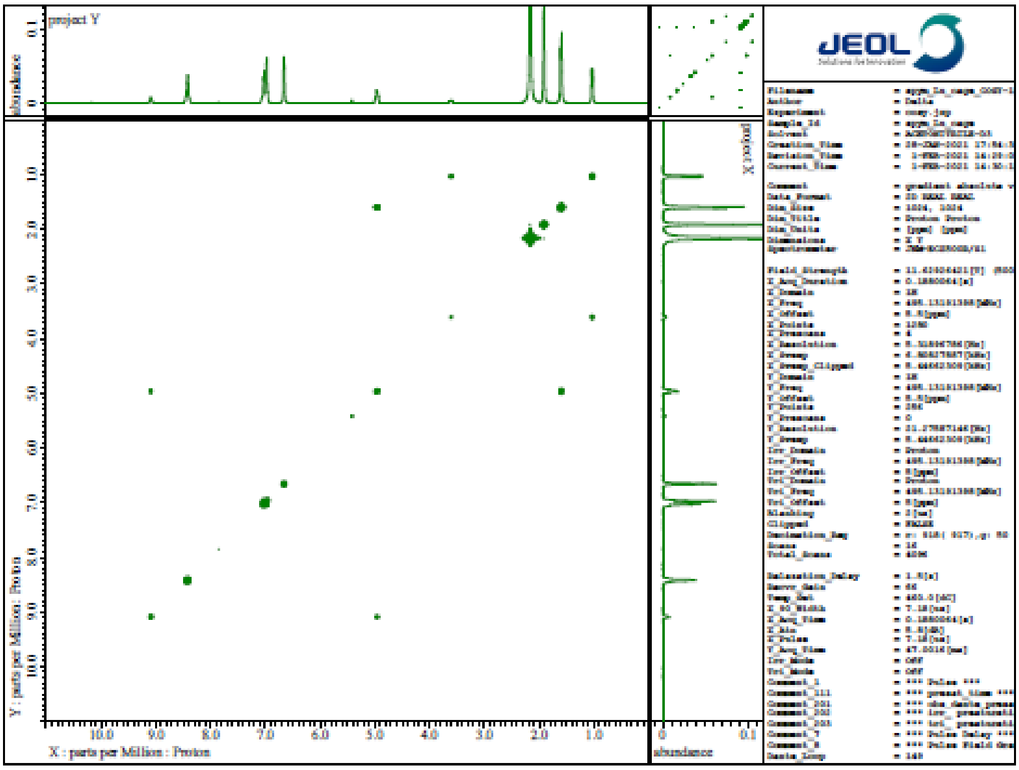

Supplementary Figure 74. COSY NMR of $[Lu_4(\mathbf{L1}^{RR})_6]$ in CD_3CN.

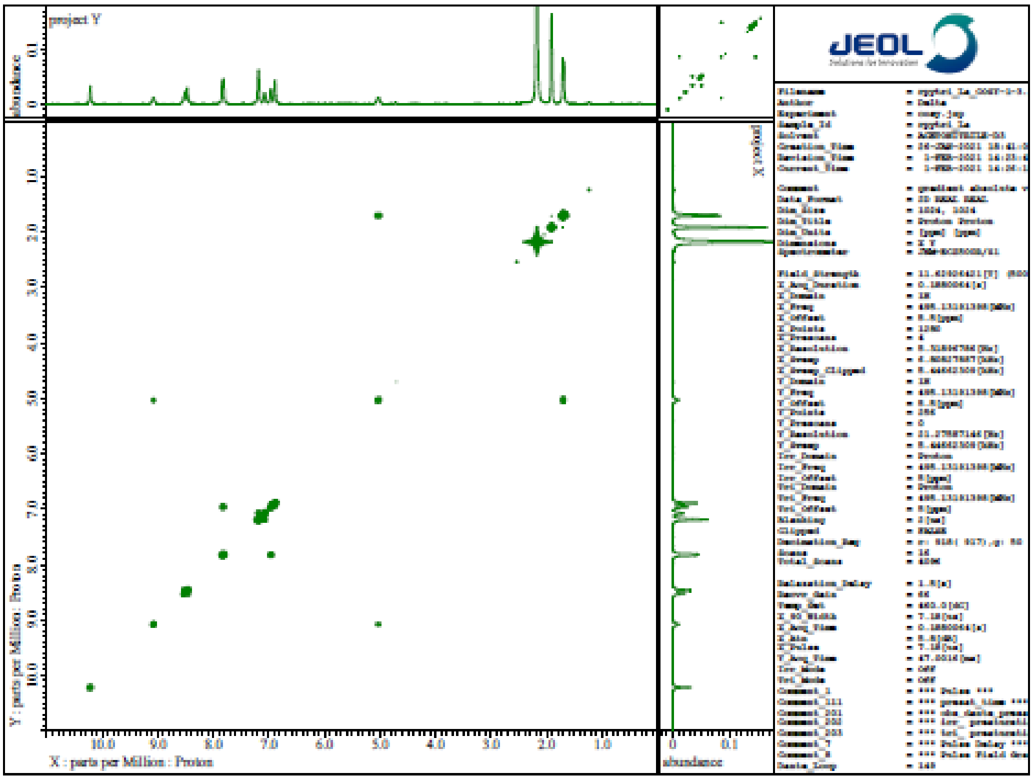

Supplementary Figure 75. COSY NMR of [La_2(**$L3^{RR}$**)$_3$] in CD_3CN.

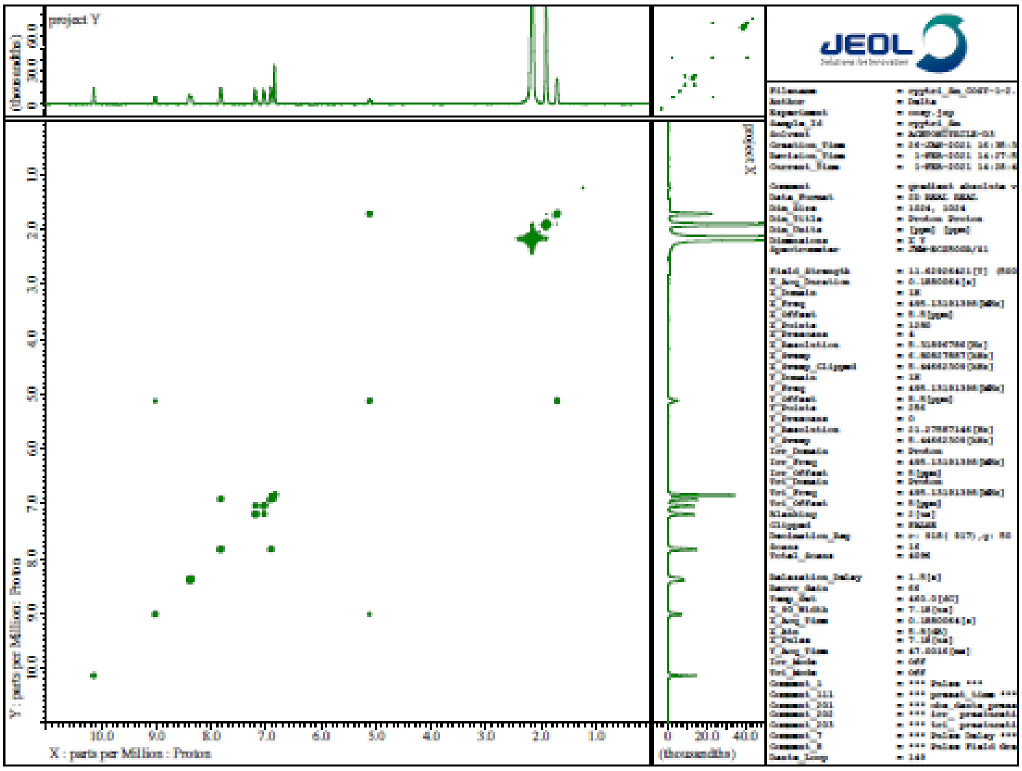

Supplementary Figure 76. COSY NMR of [Sm_2(**$L3^{RR}$**)$_3$] in CD_3CN.

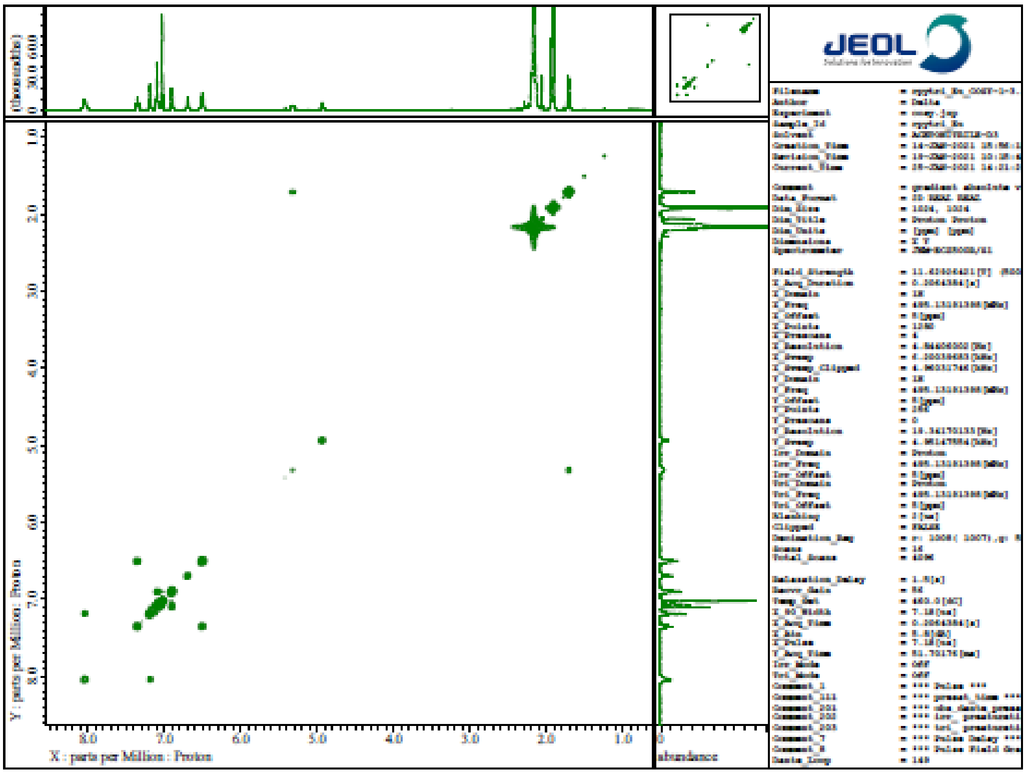

Supplementary Figure 77. COSY NMR of [Eu_2(**L3^{RR}**)$_3$] in CD_3CN.

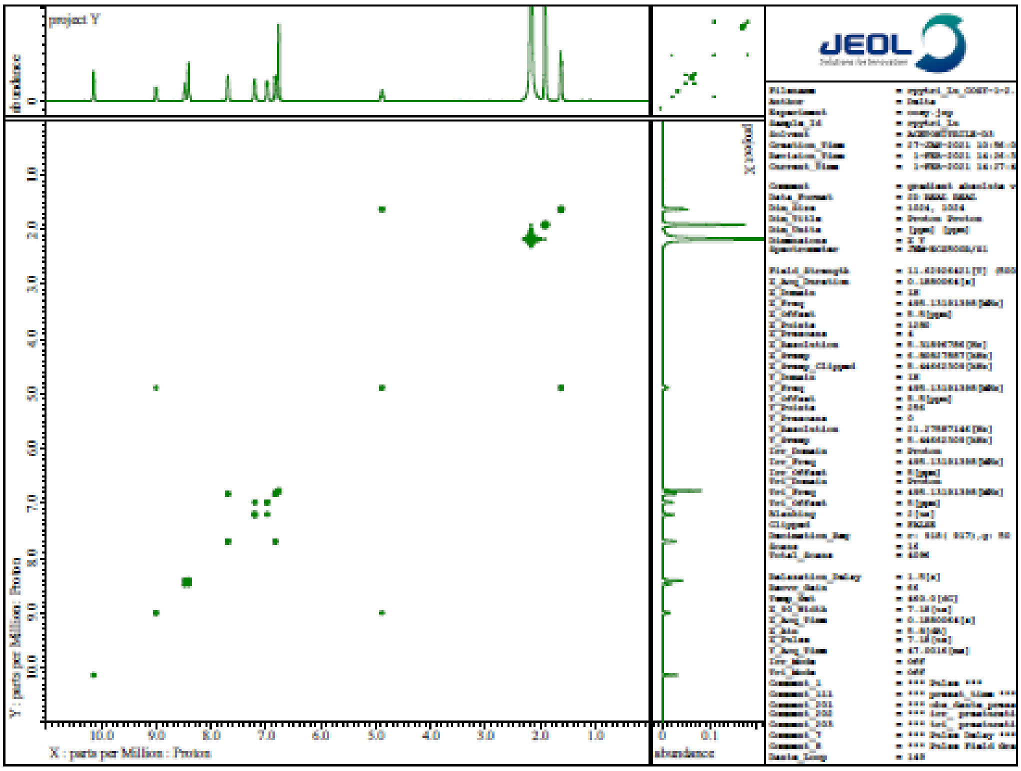

Supplementary Figure 78. COSY NMR of [Lu_2(**L3^{RR}**)$_3$] in CD_3CN.

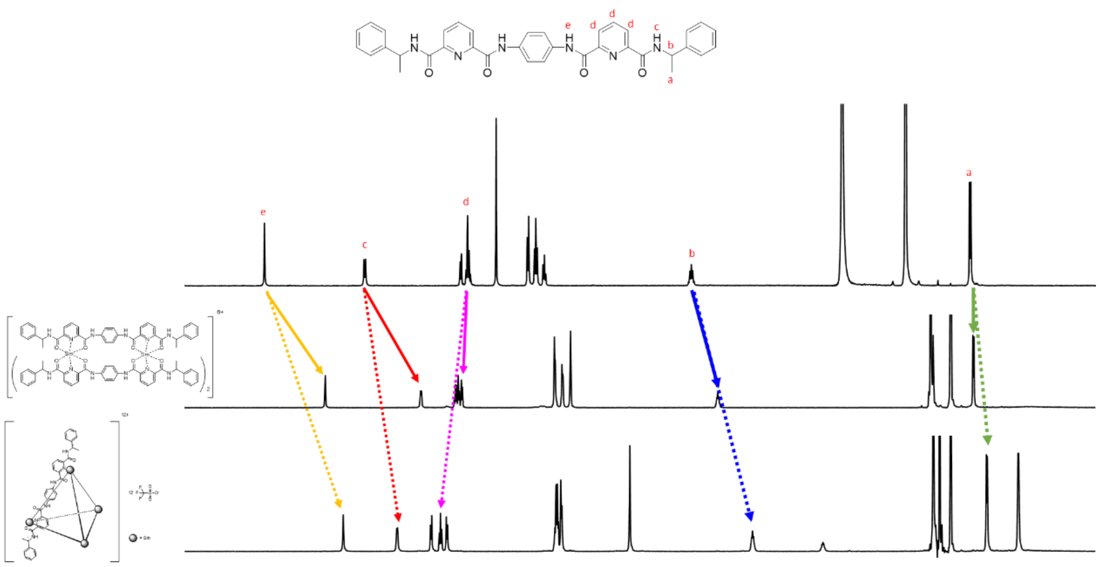

Supplementary Figure 79. Chemical shift of [Sm_2(**L1**)$_3$] and [Sm_4(**L1**)$_6$] in CD_3CN.

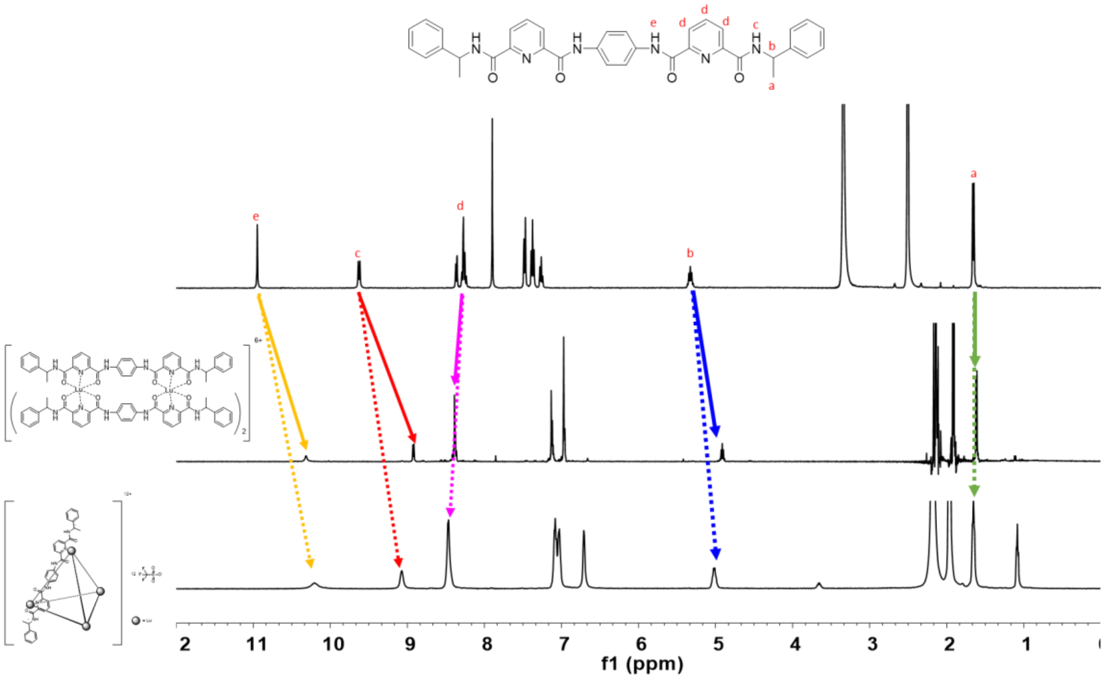

Supplementary Figure 80. Chemical shift of [Lu_2(**L1**)$_3$] and [Lu_4(**L1**)$_6$] in CD_3CN.

ESI-HRMS characterization

A

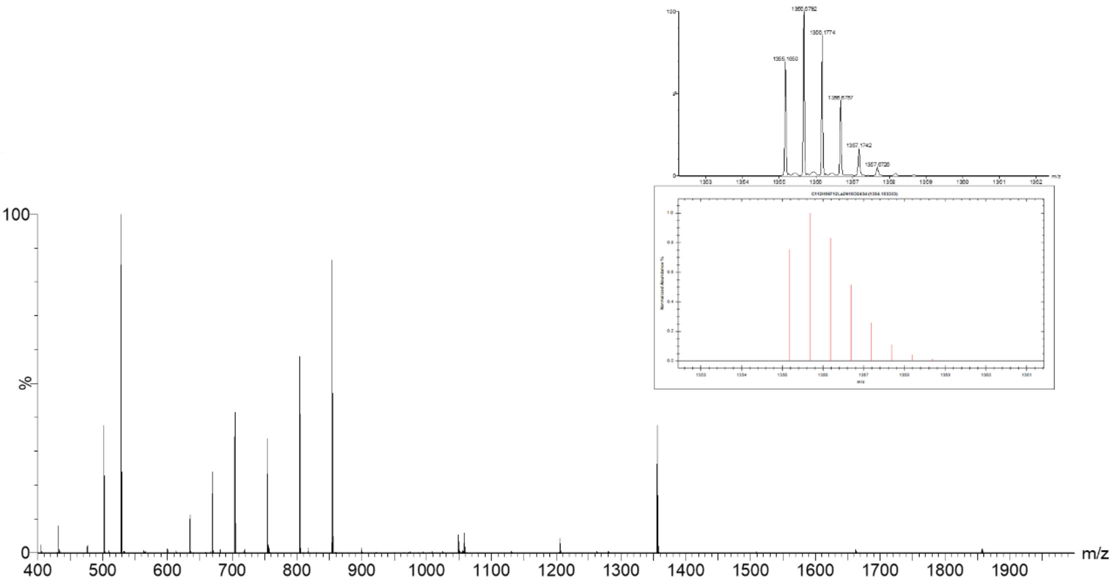

B

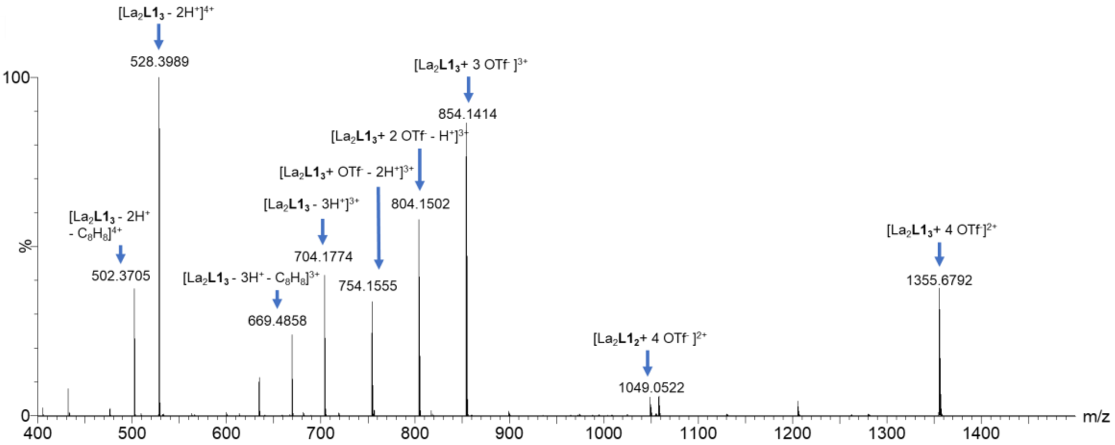

Supplementary Figure 81. ESI-HRMS of bimetallic helicate [La$_2$**L1**$_3$]. (A) The full spectrum. Simulated m/z for [La$_3$**L1**$_3$ + 4 OTf$^-$]$^{2+}$ is 1355.6841(100%), Experimental found m/z is 1355.6792(100%). Inset showing the experimental (upper) and calculated(lower) isotopic patterns. (B) Expanded region of the mass spectrum to show the possible assignments of the corresponding prominent peaks.

A

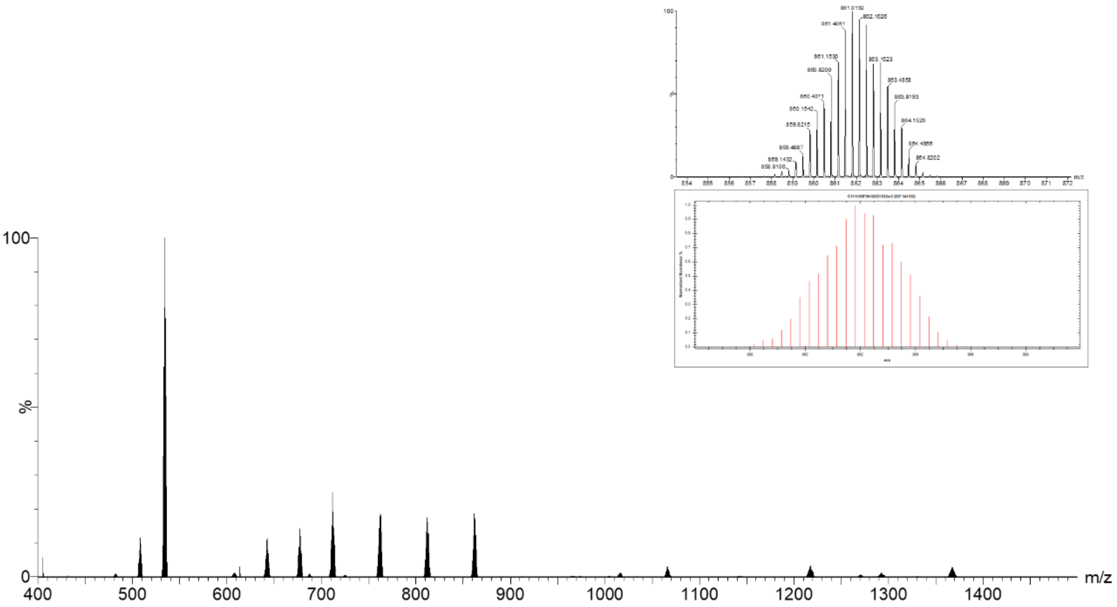

B

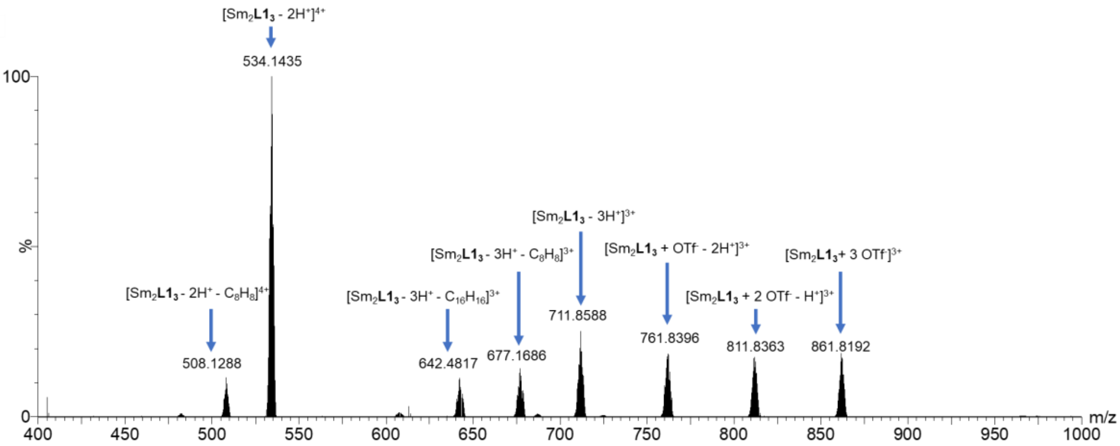

Supplementary Figure 82. ESI-HRMS of bimetallic helicate [Sm_2**L1**$_3$]. (A) The full spectrum. Simulated m/z for [Sm_2**L1**$_3$ + 3 OTf^-]$^{3+}$ is 861.8131(100%), Experimental found m/z is 861.8192(100%). Inset showing the experimental (upper) and calculated(lower) isotopic patterns. (B) Expanded region of the mass spectrum to show the possible assignments of the corresponding prominent peaks

A

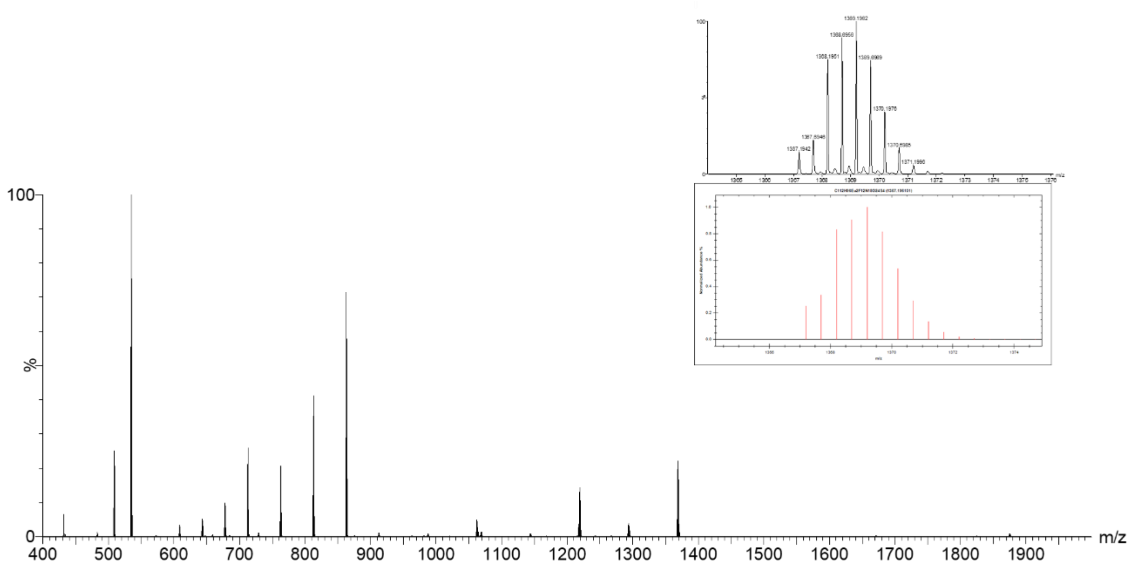

B

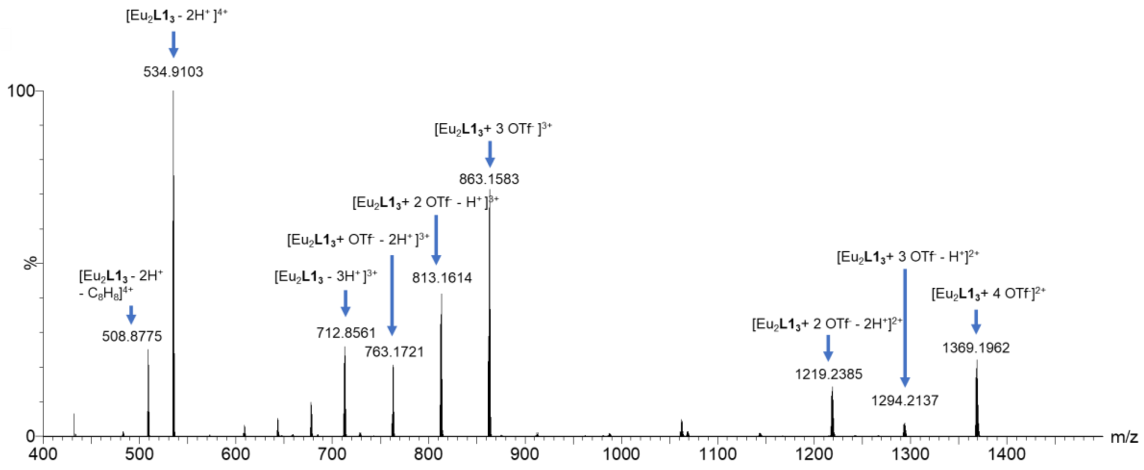

Supplementary Figure 83. ESI-HRMS of bimetallic helicate [Eu_2**L1**$_3$]. (A) The full spectrum. Simulated m/z for [Eu_3**L1**$_3$ + 4 OTf^-]$^{2+}$ is 1369.1985(100%), Experimental found m/z is 1369.1962(100%). Inset showing the experimental (upper) and calculated(lower) isotopic patterns. (B) Expanded region of the mass spectrum to show the possible assignments of the corresponding prominent peaks.

A

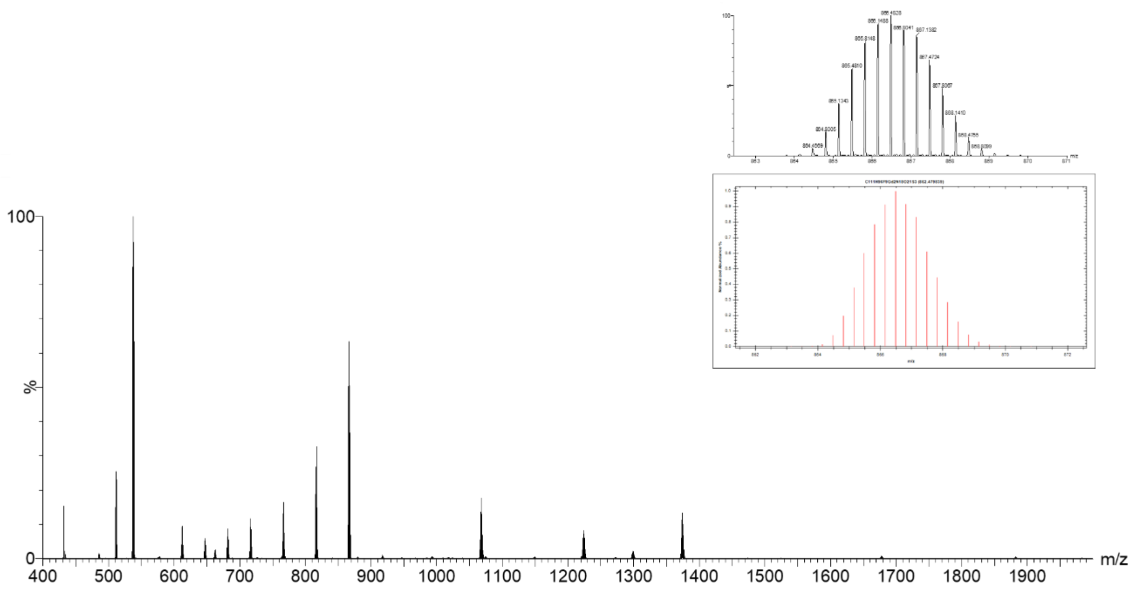

B

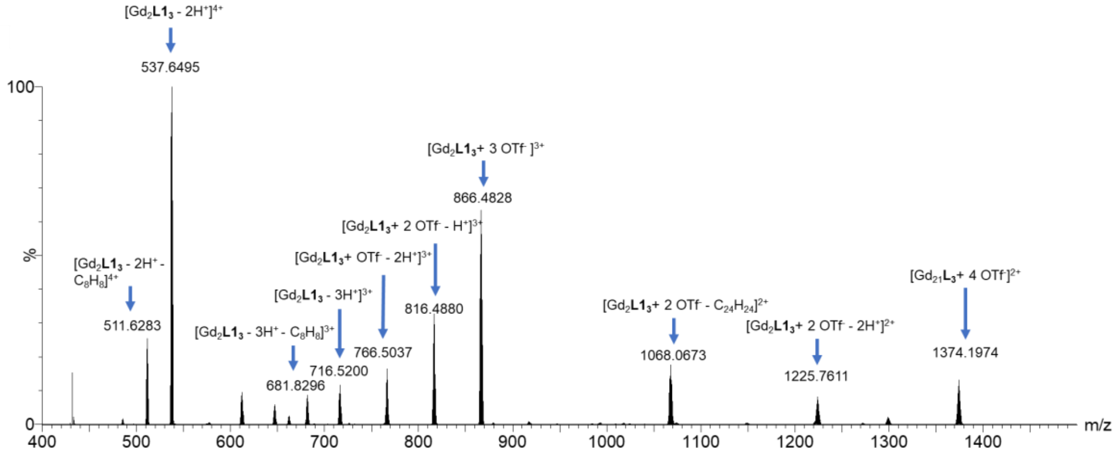

Supplementary Figure 84. ESI-HRMS of bimetallic helicate [Gd_2**L1**$_3$]. (A) The full spectrum. Simulated m/z for [Gd_2**L1**$_3$ + 3 OTf^-]$^{3+}$ is 866.4836 (100%), Experimental found m/z is 866.4828(100%). Inset showing the experimental (upper) and calculated(lower) isotopic patterns. (B) Expanded region of the mass spectrum to show the possible assignments of the corresponding prominent peaks.

A

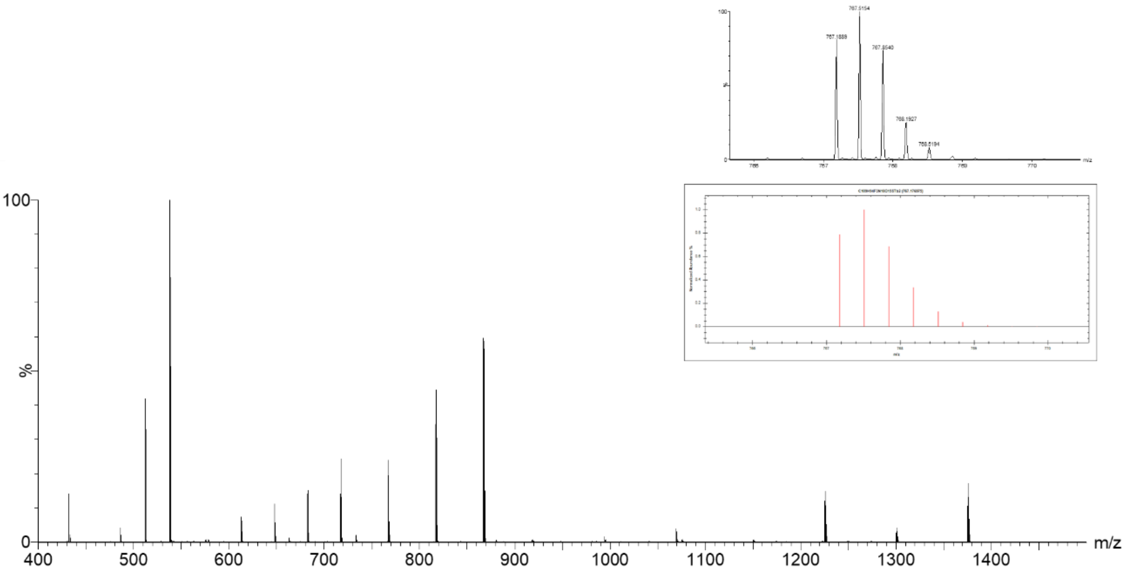

B

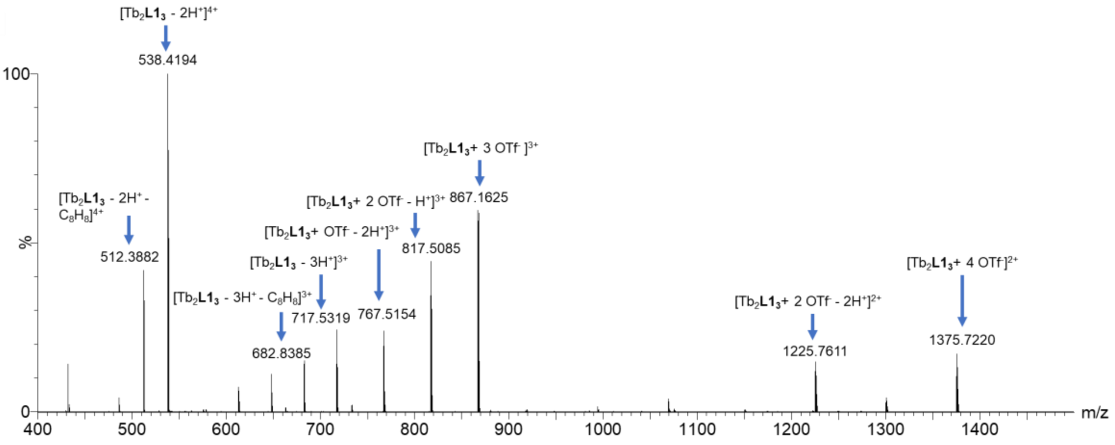

Supplementary Figure 85. ESI-HRMS of bimetallic helicate $[Tb_2\mathbf{L1}_3]$. (A) The full spectrum. Simulated m/z for $[Tb_2\mathbf{L1}_3 + OTf^- -2H^+]^{3+}$ is 767.5113(100%), Experimental found m/z is 767.5154(100%). Inset showing the experimental (upper) and calculated(lower) isotopic patterns. (B) Expanded region of the mass spectrum to show the possible assignments of the corresponding prominent peaks.

A

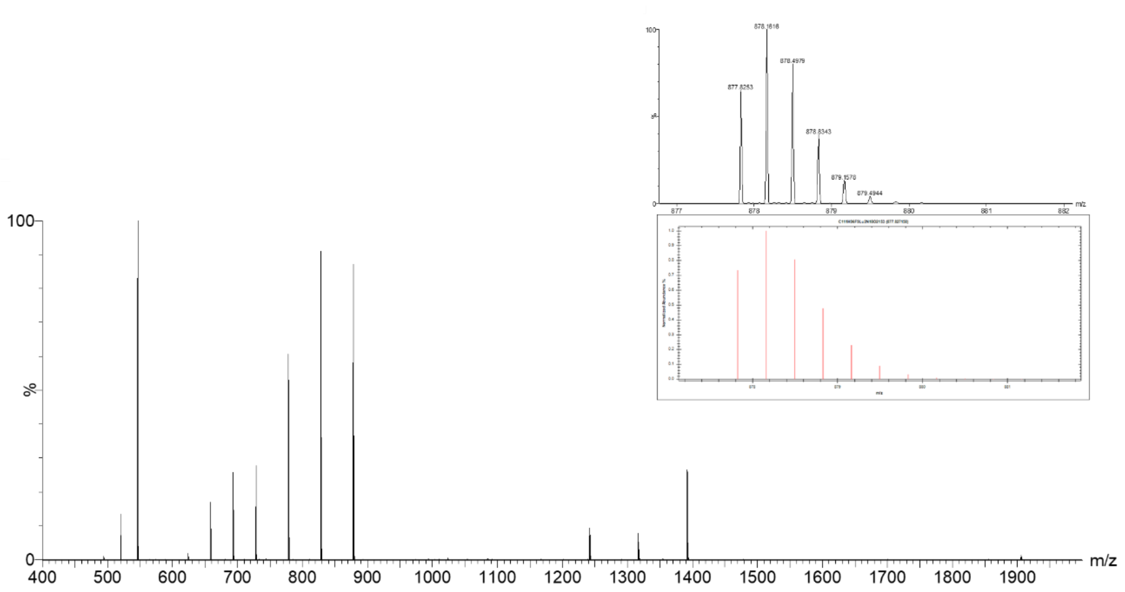

B

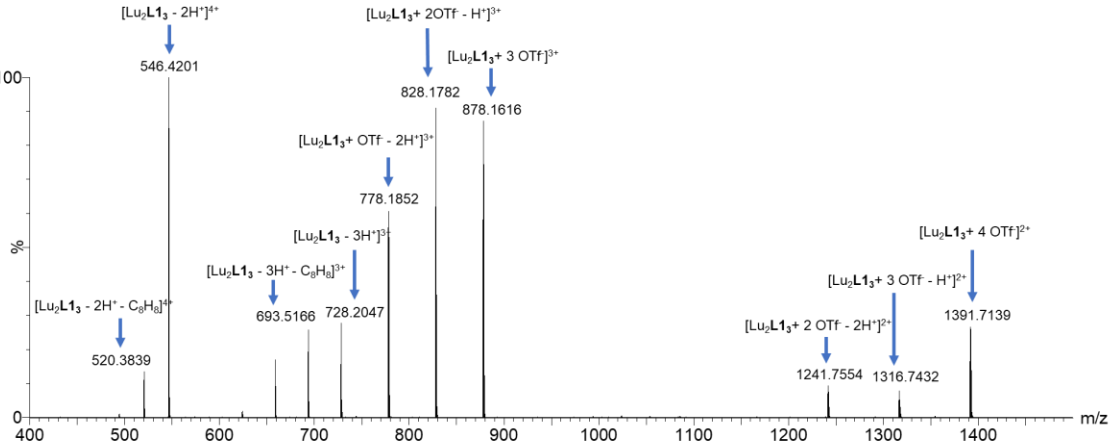

Supplementary Figure 86. ESI-HRMS of bimetallic helicate $[Lu_2\mathbf{L1}_3]$. (A) The full spectrum. Simulated m/z for $[Lu_2\mathbf{L1}_3 + 3OTf^-]^{3+}$ is 878.1615(100%), Experimental found m/z is 878.1616(100%). Inset showing the experimental (upper) and calculated(lower) isotopic patterns. (B) Expanded region of the mass spectrum to show the possible assignments of the corresponding prominent peaks.

A

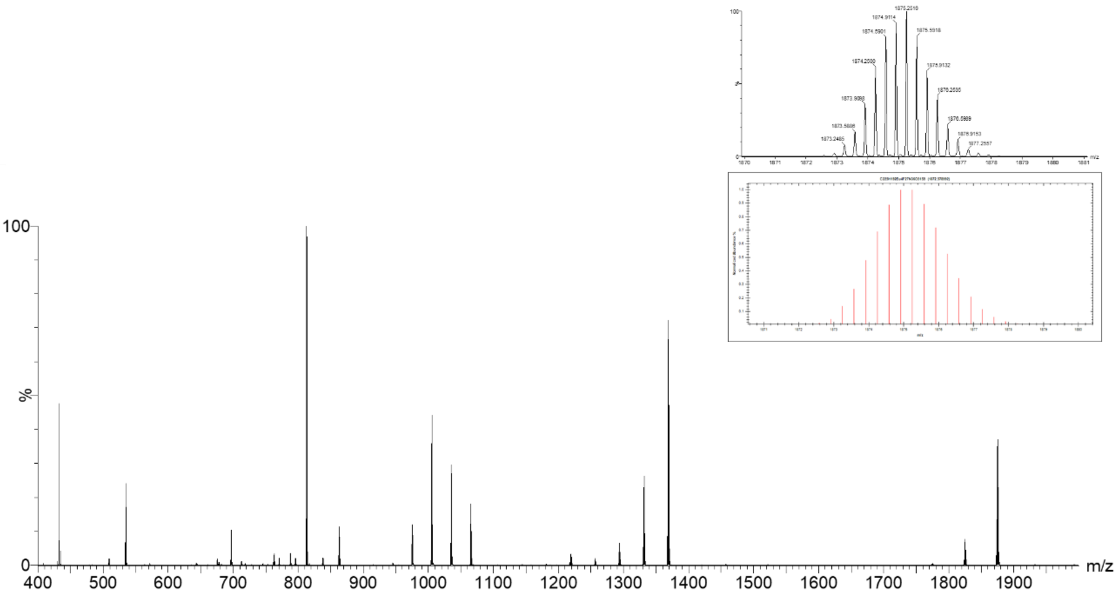

B

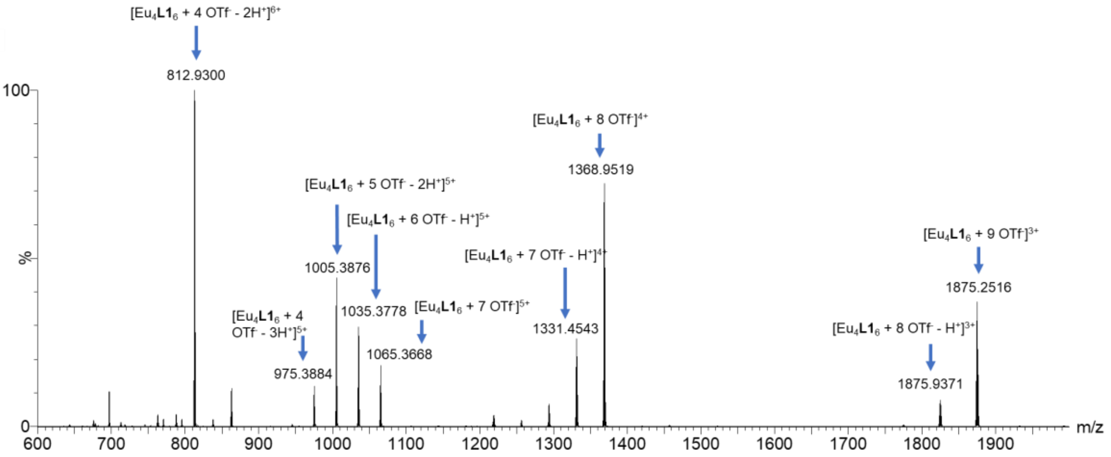

Supplementary Figure 87. ESI-HRMS of tetrahedron [Eu_4**L1**$_6$]. (A) The full spectrum. Simulated m/z for [Eu_4**L1**$_6$ + 9 OTf^-]$^{3+}$ is 1875.2490(100%), Experimental found m/z is 1875.2516(100%). Inset showing the experimental (upper) and calculated(lower) isotopic patterns. (B) Expanded region of the mass spectrum to show the possible assignments of the corresponding prominent peaks.

A

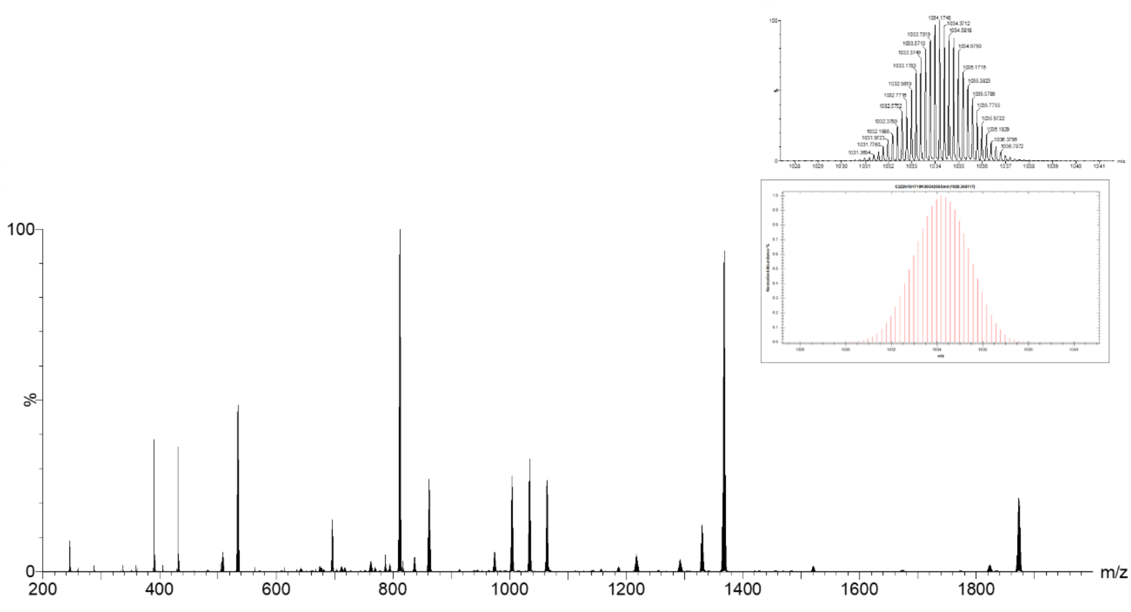

B

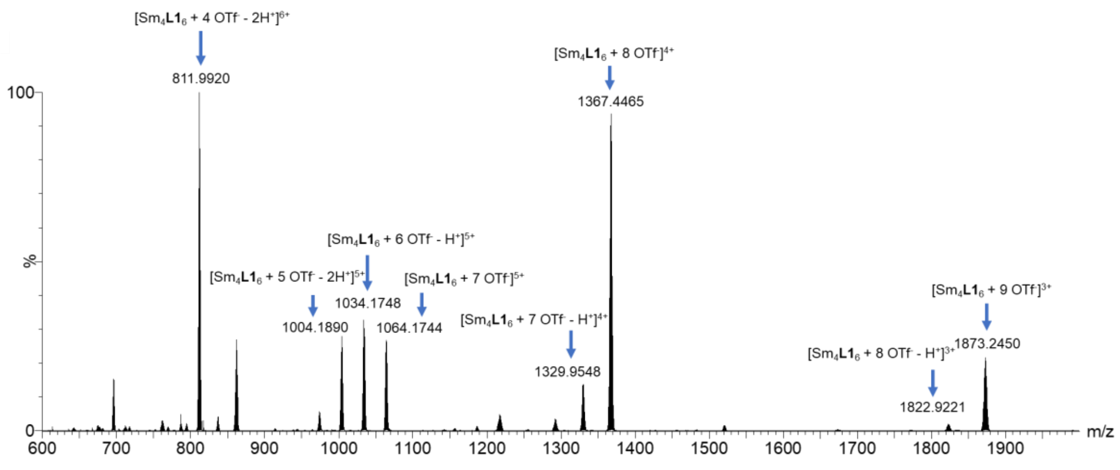

Supplementary Figure 88. ESI-HRMS of tetrahedron [Sm_4**L1**$_6$]. (A) The full spectrum. Simulated m/z for [Sm_4**L**$_6$ + 6 OTf^- - H^+]$^{5+}$ is 1034.1745(100%), Experimental found m/z is 1034.1748(100%). Inset showing the experimental (upper) and calculated(lower) isotopic patterns. (B) Expanded region of the mass spectrum to show the possible assignments of the corresponding prominent peaks.

A

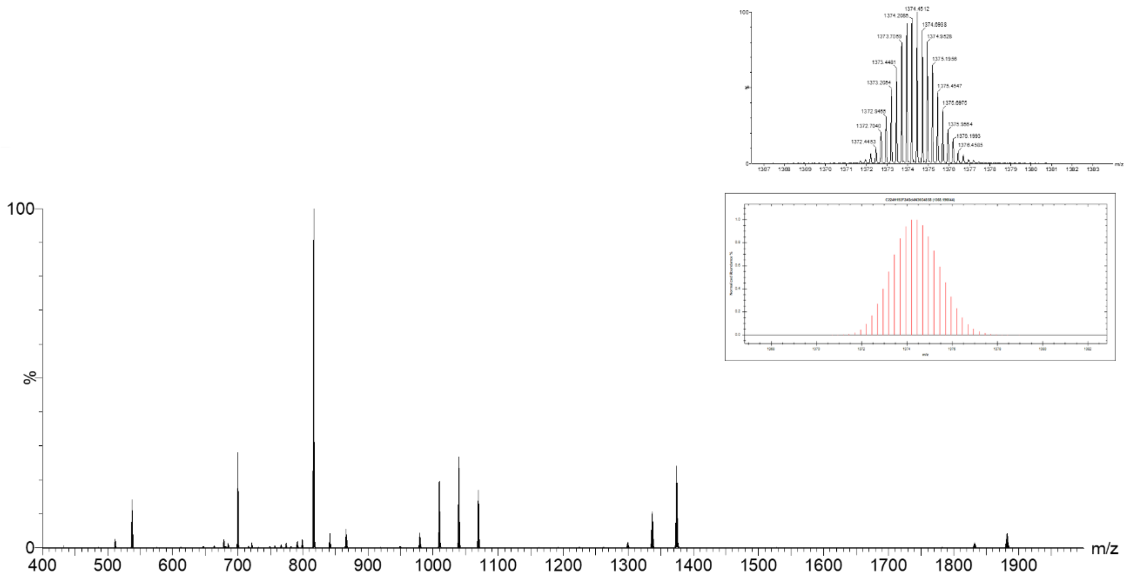

B

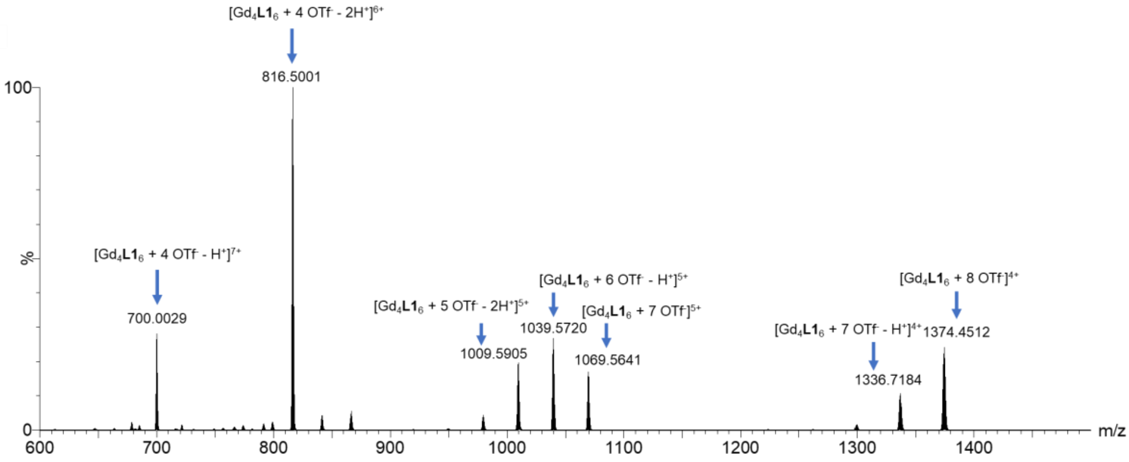

Supplementary Figure 89. ESI-HRMS of tetrahedron [Gd_4**L1**$_6$]. (A) The full spectrum. Simulated m/z for [Gd_4**L1**$_6$ + 8 OTf^-]$^{4+}$ is 1374.4520 (100%), Experimental found m/z is 1374.4512(100%). Inset showing the experimental (upper) and calculated(lower) isotopic patterns. (B) Expanded region of the mass spectrum to show the possible assignments of the corresponding prominent peaks.

A

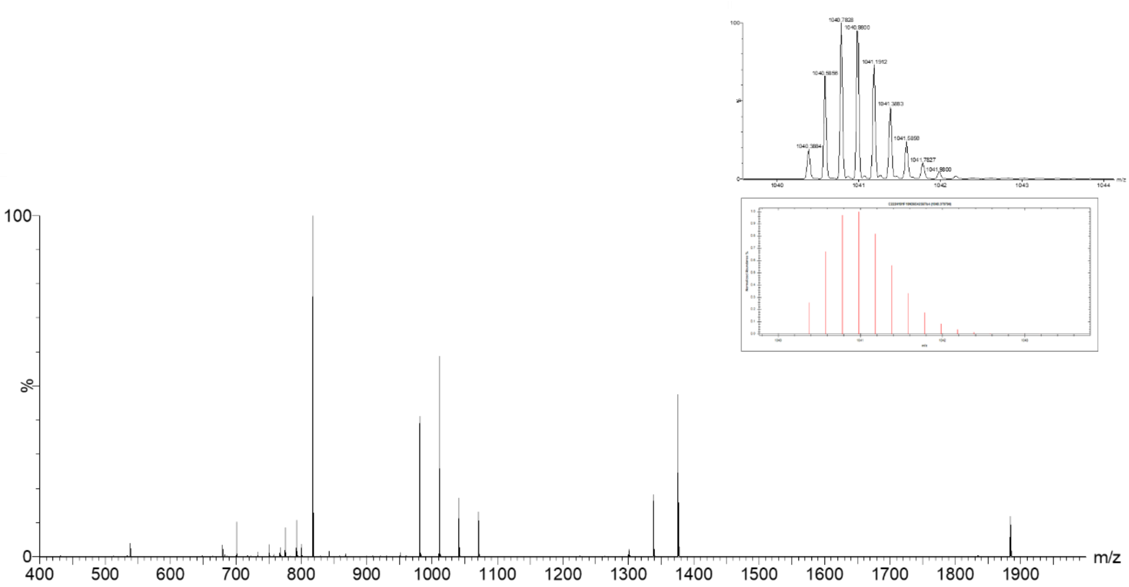

B

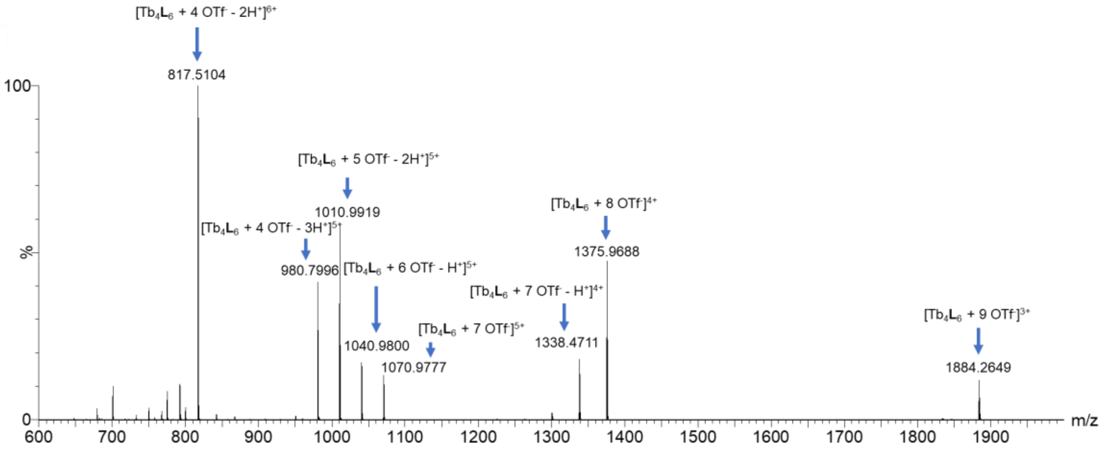

Supplementary Figure 90. ESI-HRMS of tetrahedron [Tb_4**L1**$_6$]. (A) The full spectrum. Simulated m/z for [Tb_4**L1**$_6$ + 6 OTf^- - H^+]$^{5+}$ is 1040.9802 (100%), Experimental found m/z is 1040.9800(100%). Inset showing the experimental (upper) and calculated(lower) isotopic patterns. (B) Expanded region of the mass spectrum to show the possible assignments of the corresponding prominent peaks.

A

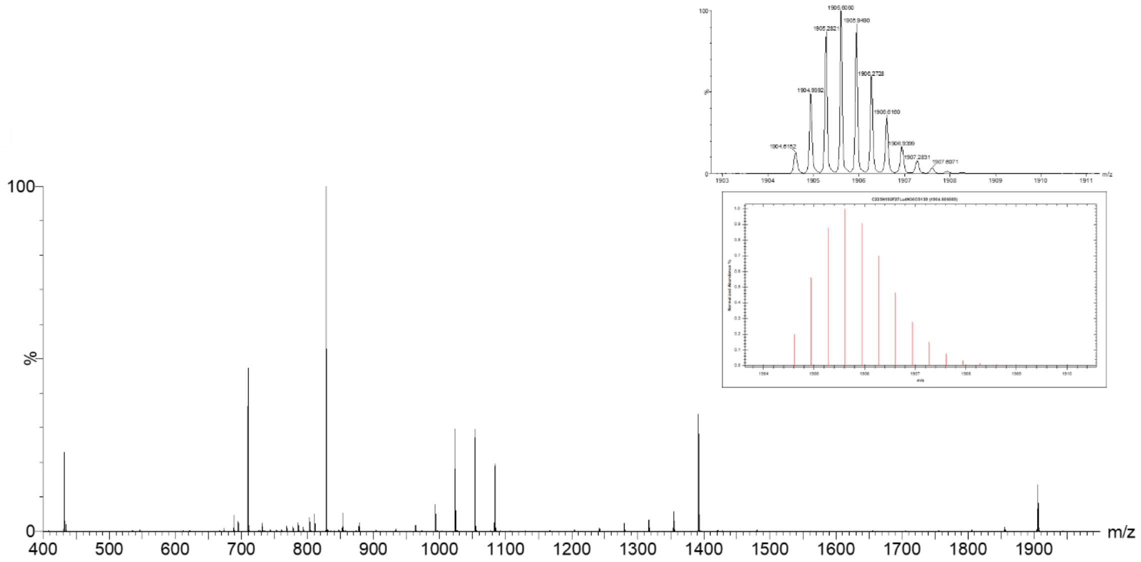

B

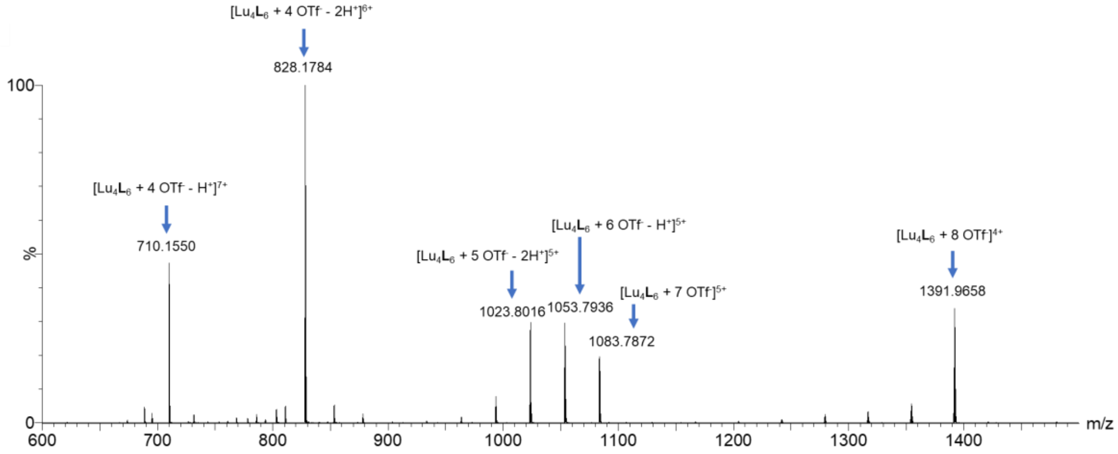

Supplementary Figure 91. ESI-HRMS of tetrahedron $[Lu_4\mathbf{L1}_6]$. (A) The full spectrum. Simulated m/z for $[Lu_4\mathbf{L1}_6 + 9OTf^-]^{3+}$ is 1905.6090(100%), Experimental found m/z is 1905.6060(100%). Inset showing the experimental (upper) and calculated(lower) isotopic patterns. (B) Expanded region of the mass spectrum to show the possible assignments of the corresponding prominent peaks.

A

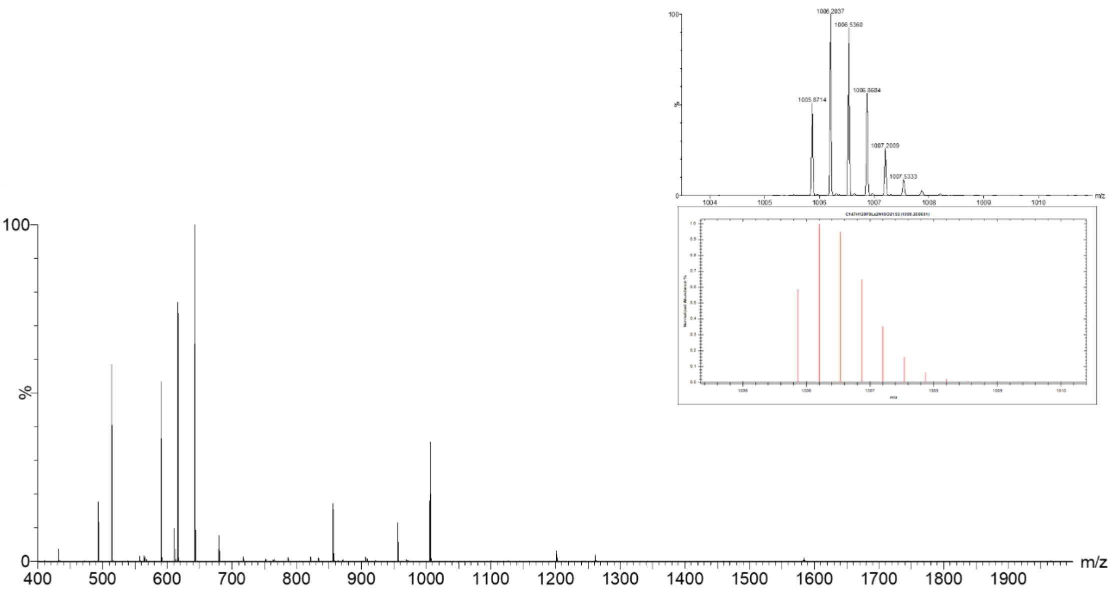

B

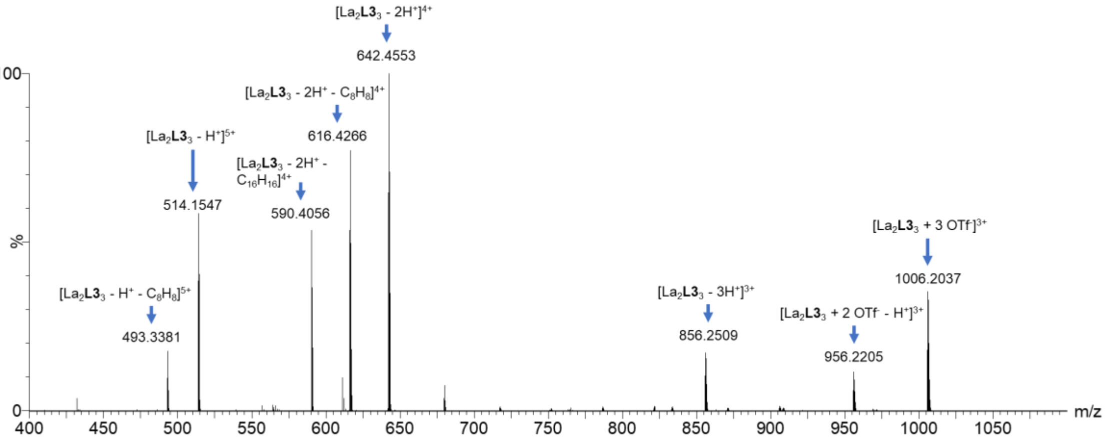

Supplementary Figure 92. ESI-HRMS of bimetallic helicate [La_2**L3**$_3$]. (A) The full spectrum. Simulated m/z for [La_2**L3**$_3$ + $3OTf^-$]$^{3+}$ is 1006.2012(100%), Experimental found m/z is 1006.2037(100%). Inset showing the experimental (upper) and calculated(lower) isotopic patterns. (B) Expanded region of the mass spectrum to show the possible assignments of the corresponding prominent peaks.

A

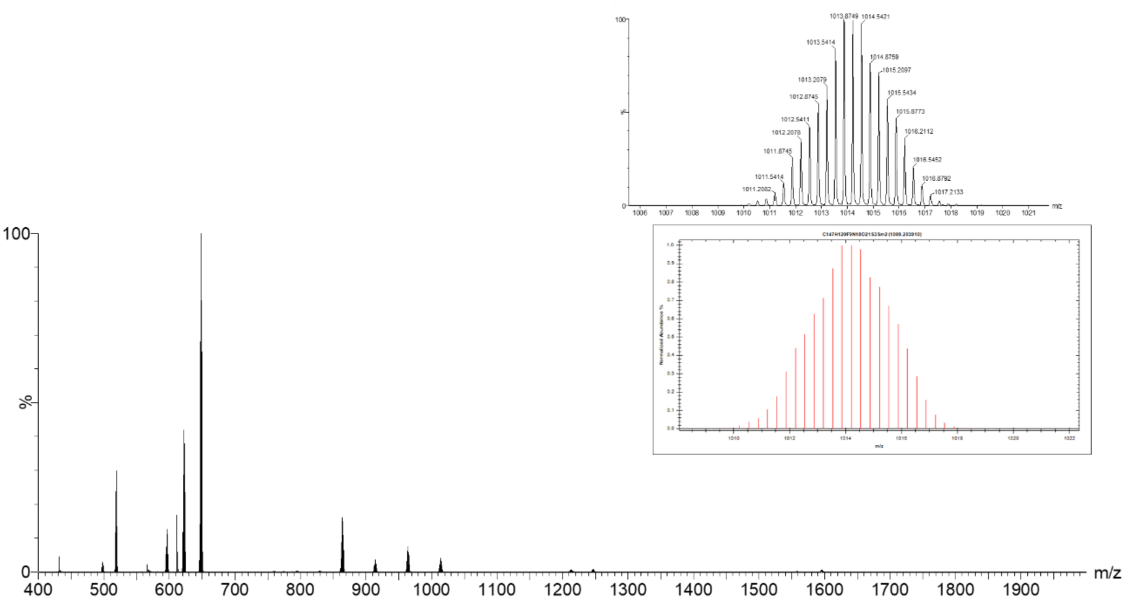

B

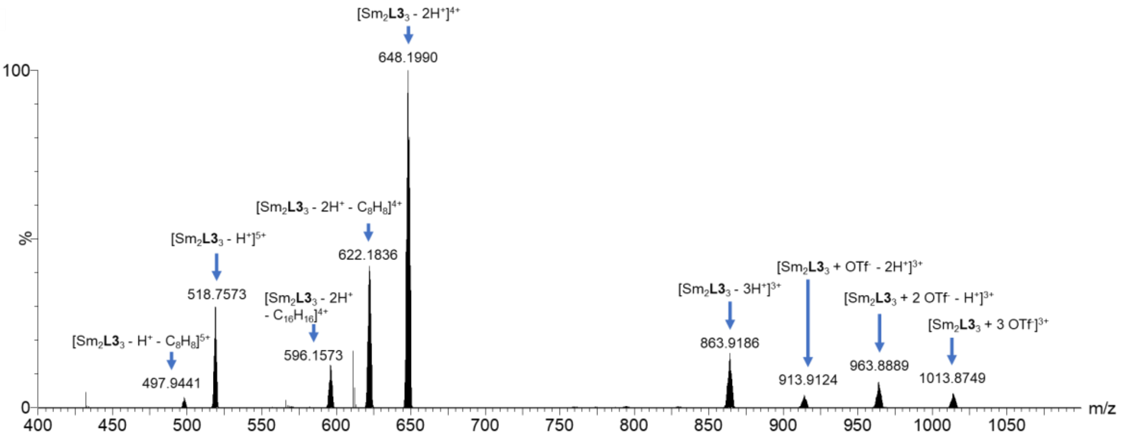

Supplementary Figure 93. ESI-HRMS of bimetallic helicate [Sm_2**L3**$_3$]. (A) The full spectrum. Simulated m/z for [Sm_2**L3**$_3$ + $3OTf^-$]$^{3+}$ is 1013.8759(100%), Experimental found m/z is 1013.8749(100%). Inset showing the experimental (upper) and calculated(lower) isotopic patterns. (B) Expanded region of the mass spectrum to show the possible assignments of the corresponding prominent peaks.

A

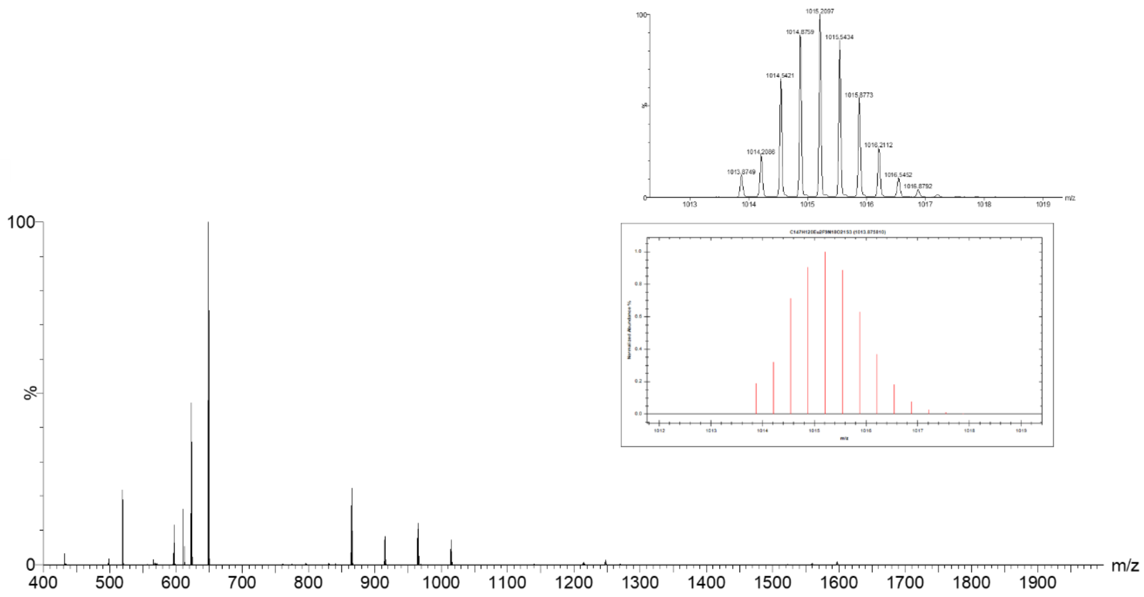

B

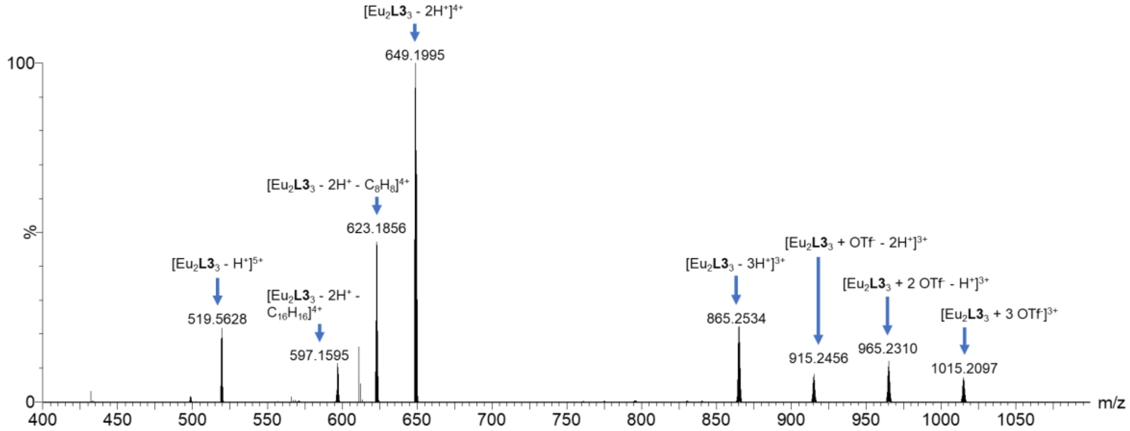

Supplementary Figure 94. ESI-HRMS of bimetallic helicate [Eu_2**L3**$_3$]. (A) The full spectrum. Simulated m/z for [Eu_2**L3**$_3$ + $3OTf^-$]$^{3+}$ is 1015.2111(100%), Experimental found m/z is 1015.2097(100%). Inset showing the experimental (upper) and calculated(lower) isotopic patterns. (B) Expanded region of the mass spectrum to show the possible assignments of the corresponding prominent peaks.

A

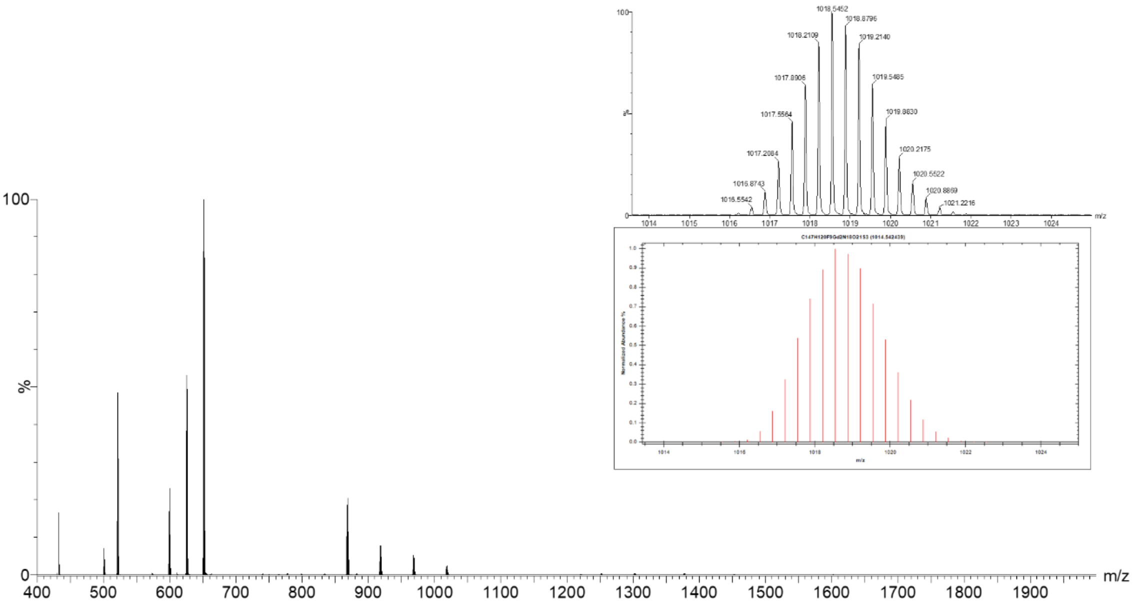

B

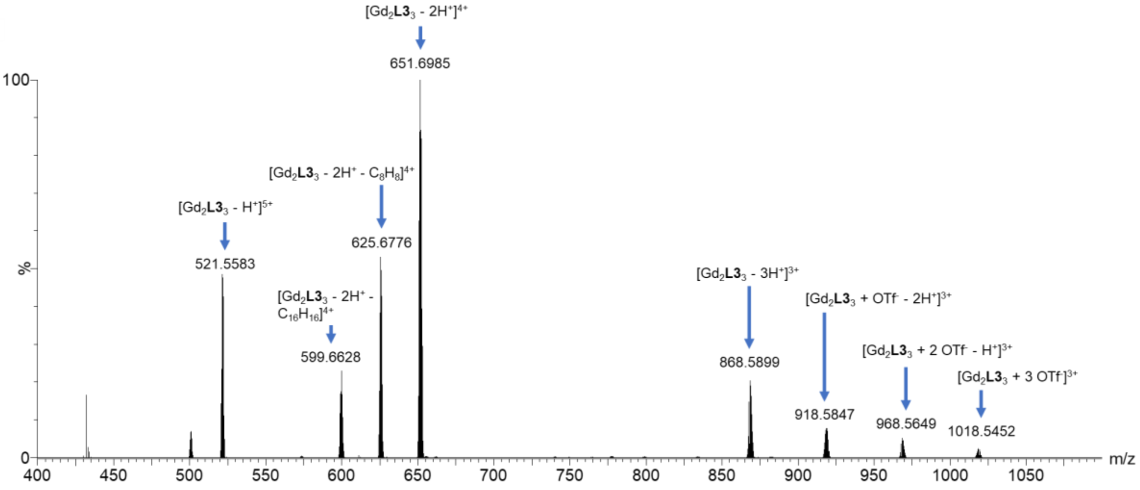

Supplementary Figure 95. ESI-HRMS of bimetallic helicate [Gd_2**L3**$_3$]. (A) The full spectrum. Simulated m/z for [Gd_2**L3**$_3$ + 3OTf$^-$]$^{3+}$ is 1018.5452(100%), Experimental found m/z is 1018.5452(100%). Inset showing the experimental (upper) and calculated(lower) isotopic patterns. (B) Expanded region of the mass spectrum to show the possible assignments of the corresponding prominent peaks.

A

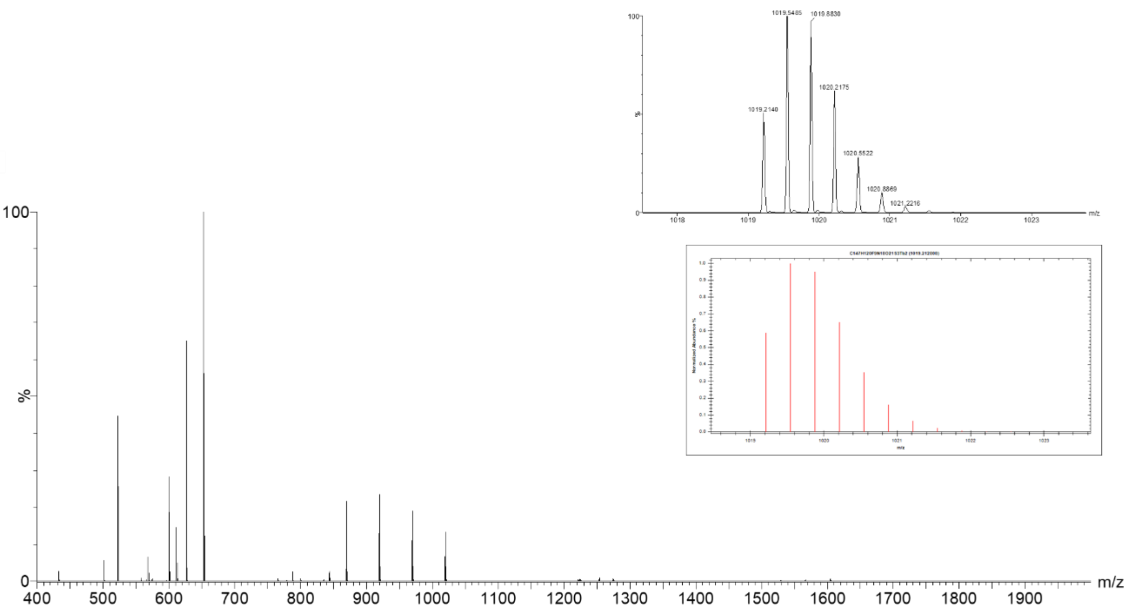

B

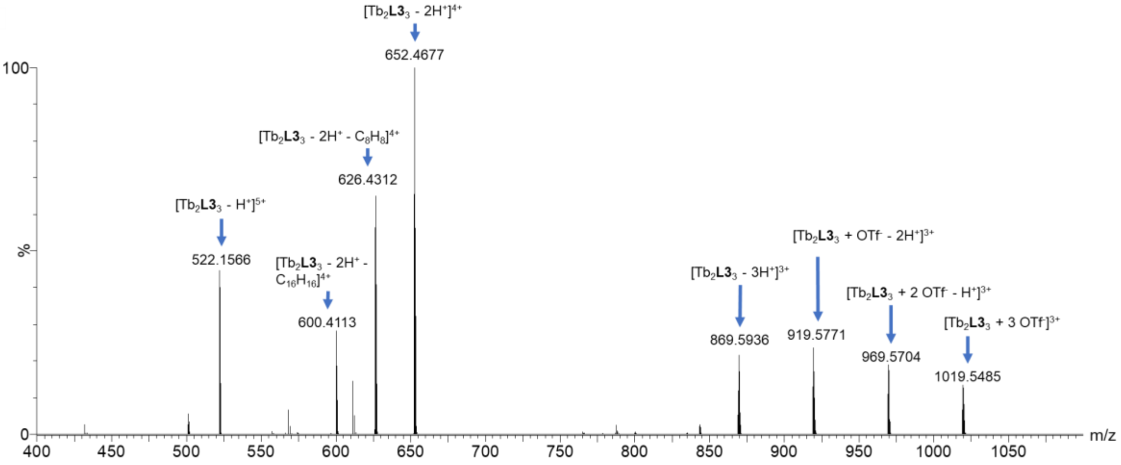

Supplementary Figure 96. ESI-HRMS of bimetallic helicate $[Tb_2\mathbf{L3}_3]$. (A) The full spectrum. Simulated m/z for $[Tb_2\mathbf{L3}_3 + 3OTf^-]^{3+}$ is 1019.5472(100%), Experimental found m/z is 1019.5485(100%). Inset showing the experimental (upper) and calculated(lower) isotopic patterns. (B) Expanded region of the mass spectrum to show the possible assignments of the corresponding prominent peaks.

A

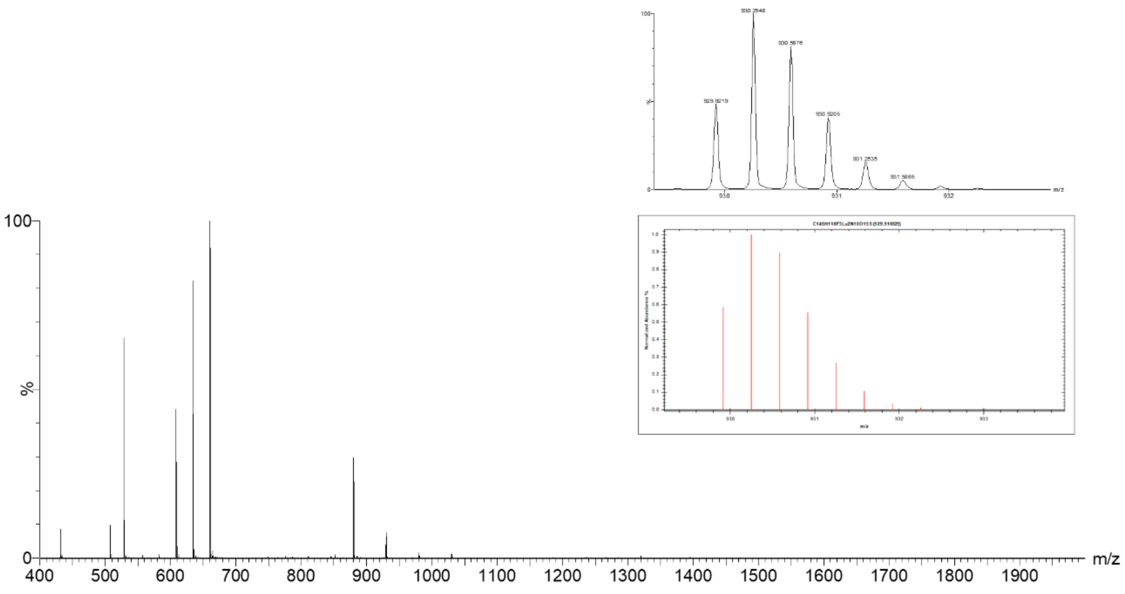

B

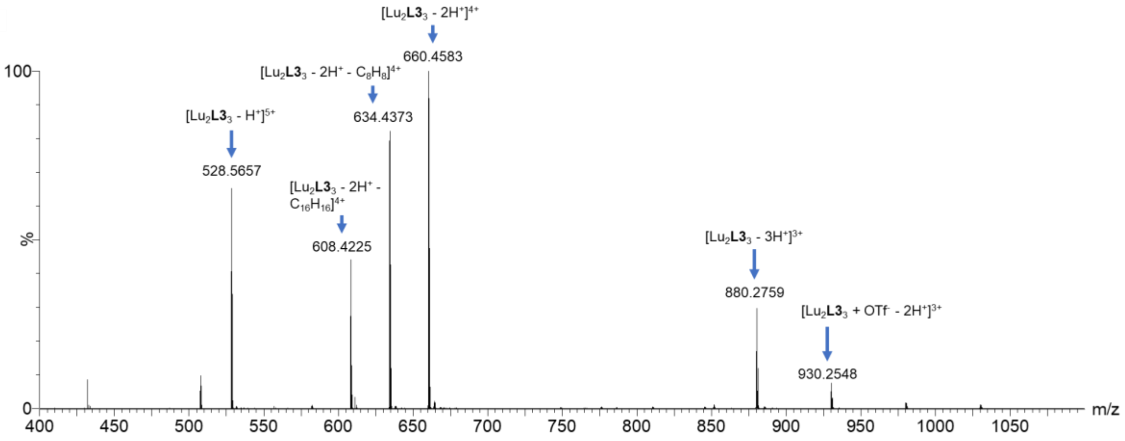

Supplementary Figure 97. ESI-HRMS of bimetallic helicate [Lu_2**L3**$_3$]. (A) The full spectrum. Simulated m/z for [Lu_2**L3**$_3$ + OTf^- - $2H^+$]$^{3+}$ is 930.2509(100%), Experimental found m/z is 930.2548(100%). Inset showing the experimental (upper) and calculated(lower) isotopic patterns. (B) Expanded region of the mass spectrum to show the possible assignments of the corresponding prominent peaks.

Photophysical studies

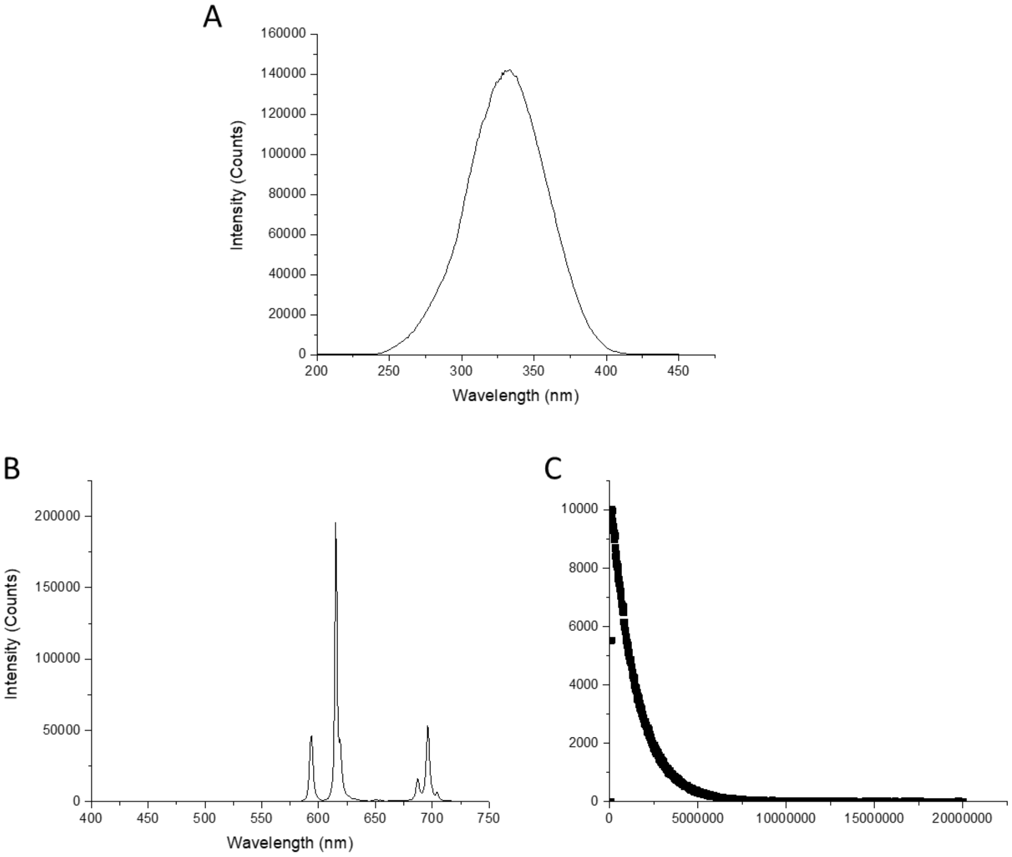

Supplementary Figure 98. Luminescent data of [Eu_2(**L1**^{SS})$_3$](OTf)$_6$ (1.58 x 10^{-6} M in CH_3CN).(A) Excitation spectrum, λ_{em} = 616 nm, slits = 1.5-1.0, filter 380 nm. (b) Emission spectrum, λ_{ex} = 311 nm, slits = 1.5-1.0, filter 380 nm. (c) Excited state decay curve, λ_{em} = 616 nm, slits = 6-5, filter 380 nm.

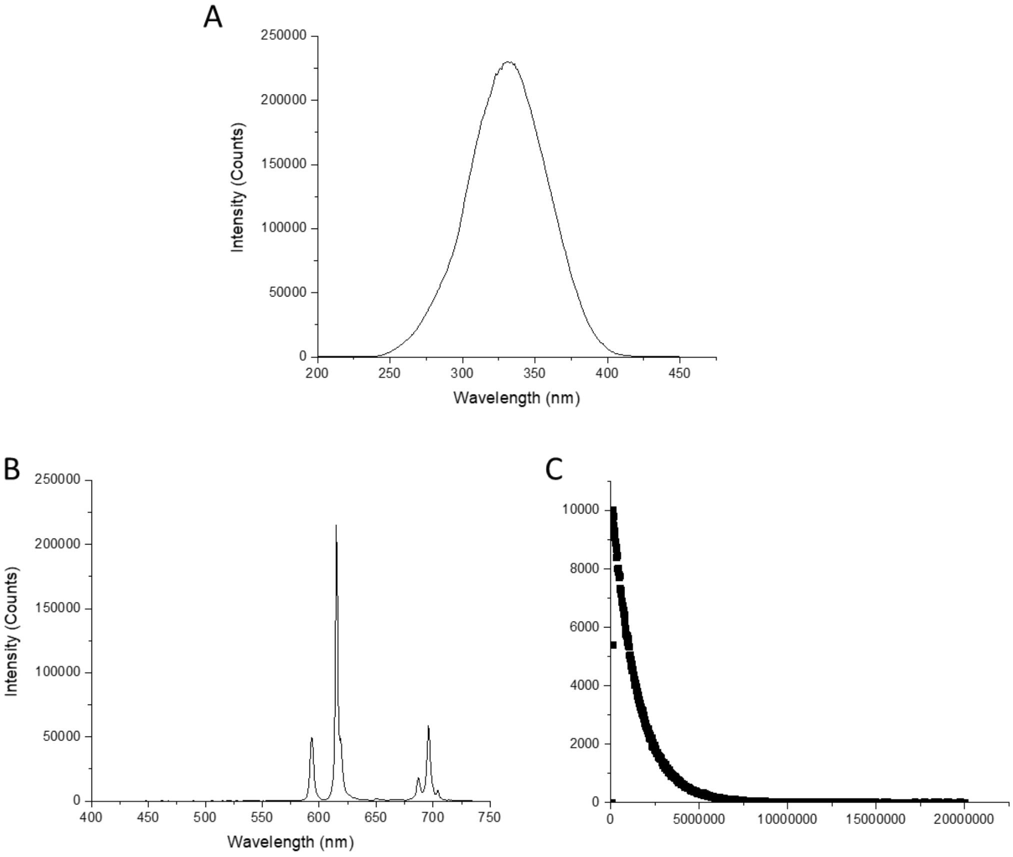

Supplementary Figure 99. Luminescent data of $[Eu_2(\mathbf{L1^{RR}})_3](OTf)_6$ (1.60 x 10^{-5} M in CH_3CN).(A) Excitation spectrum, λ_{em} = 616 nm, slits = 1.5-1.0, filter 380 nm. (b) Emission spectrum, λ_{ex} = 311 nm, slits = 1.5-1.0, filter 380 nm. (c) Excited state decay curve, λ_{em} = 616 nm, slits = 6-5, filter 380 nm.

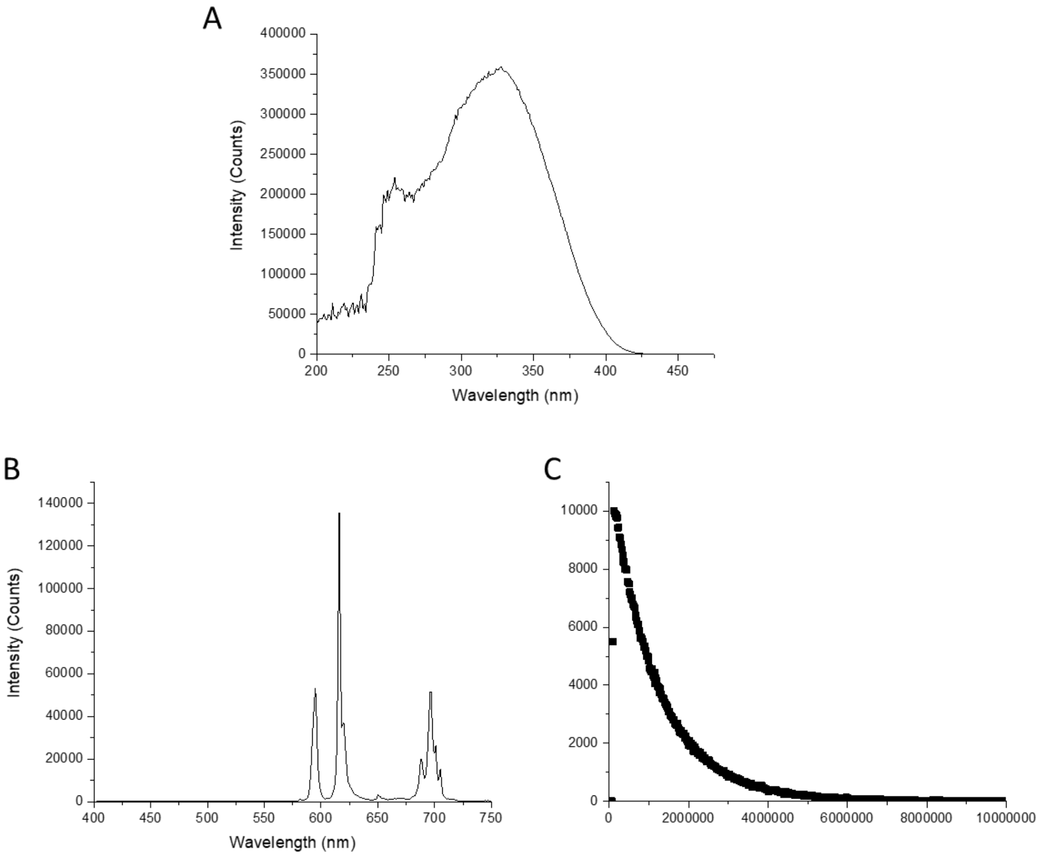

Supplementary Figure 100. Luminescent data of $[Eu_4(\mathbf{L1}^{SS})_6](OTf)_{12}$ (2.50 x 10^{-6} M in CH_3CN).(A) Excitation spectrum, λ_{em} = 616 nm, slits = 1.5-1.0, filter 380 nm. (b) Emission spectrum, λ_{ex} = 330 nm, slits = 1.5-1.0, filter 380 nm. (c) Excited state decay curve, λ_{em} = 616 nm, slits = 5-3, filter 380 nm.

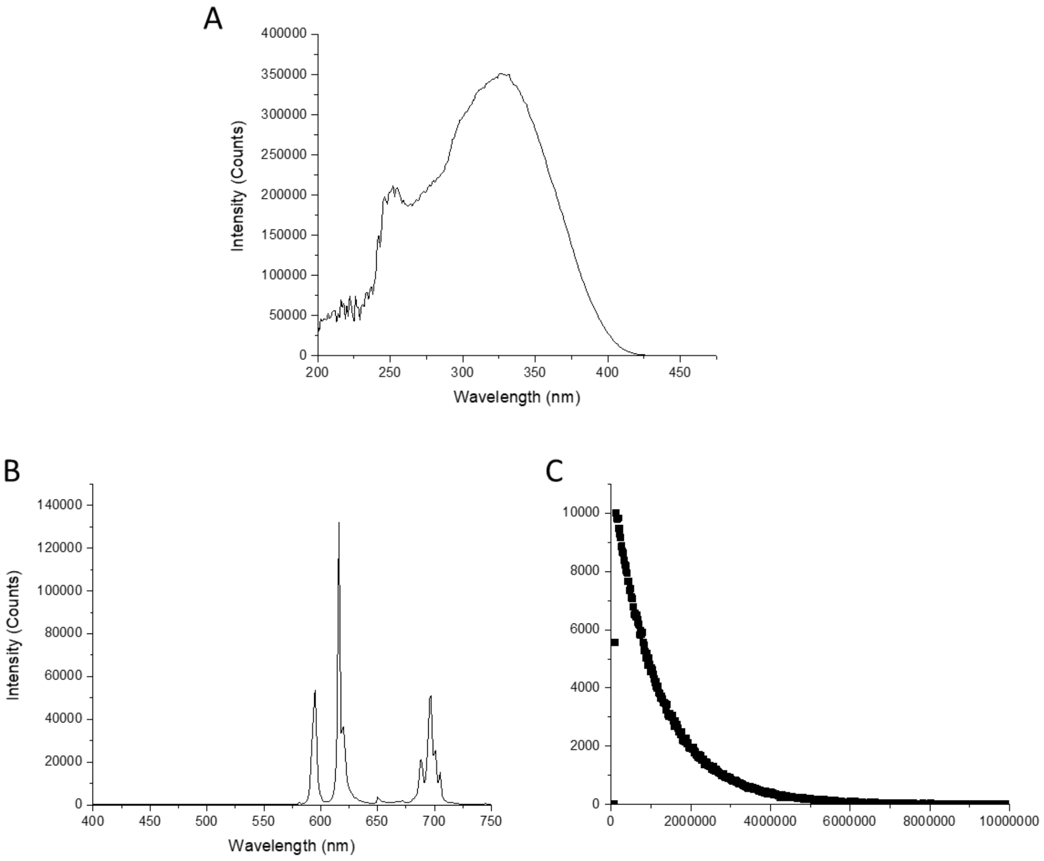

Supplementary Figure 101. Luminescent data of $[Eu_4(\mathbf{L1^{RR}})_6](OTf)_{12}$ (2.41 x 10^{-6} M in CH_3CN).(A) Excitation spectrum, λ_{em} = 616 nm, slits = 1.5-1.0, filter 380 nm. (b) Emission spectrum, λ_{ex} = 330 nm, slits = 1.5-1.0, filter 380 nm. (c) Excited state decay curve, λ_{em} = 616 nm, slits = 5-3, filter 380 nm.

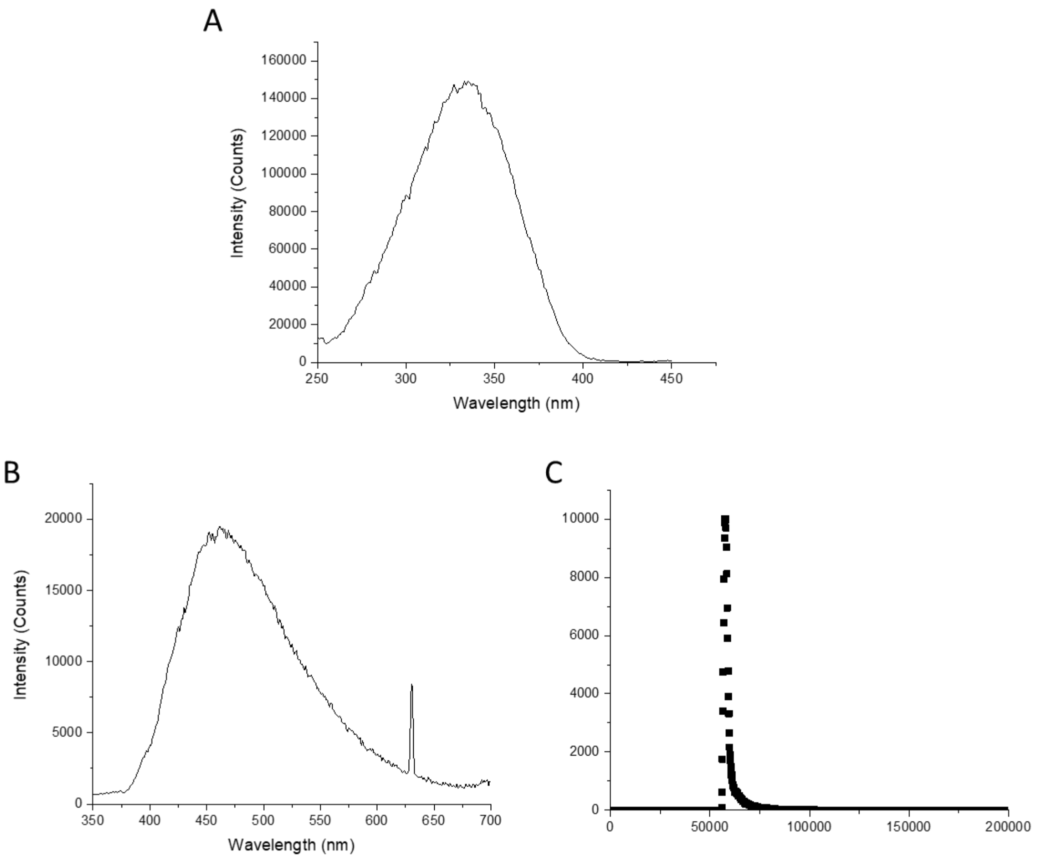

Supplementary Figure 102. Luminescent data of $[Gd_2(\mathbf{L1}^{RR})_3](OTf)_6$ (6.13 x 10^{-6} M in 1:4 of MeOH/EtOH at 77K).(A) Excitation spectrum, λ_{em} = 462 nm, slits = 1.0-0.5, filter 380 nm. (b) Emission spectrum, λ_{ex} = 315 nm, slits = 1.0-0.5, filter 380 nm. (c) Excited state decay curve, λ_{em} = 462 nm, filter 380 nm.

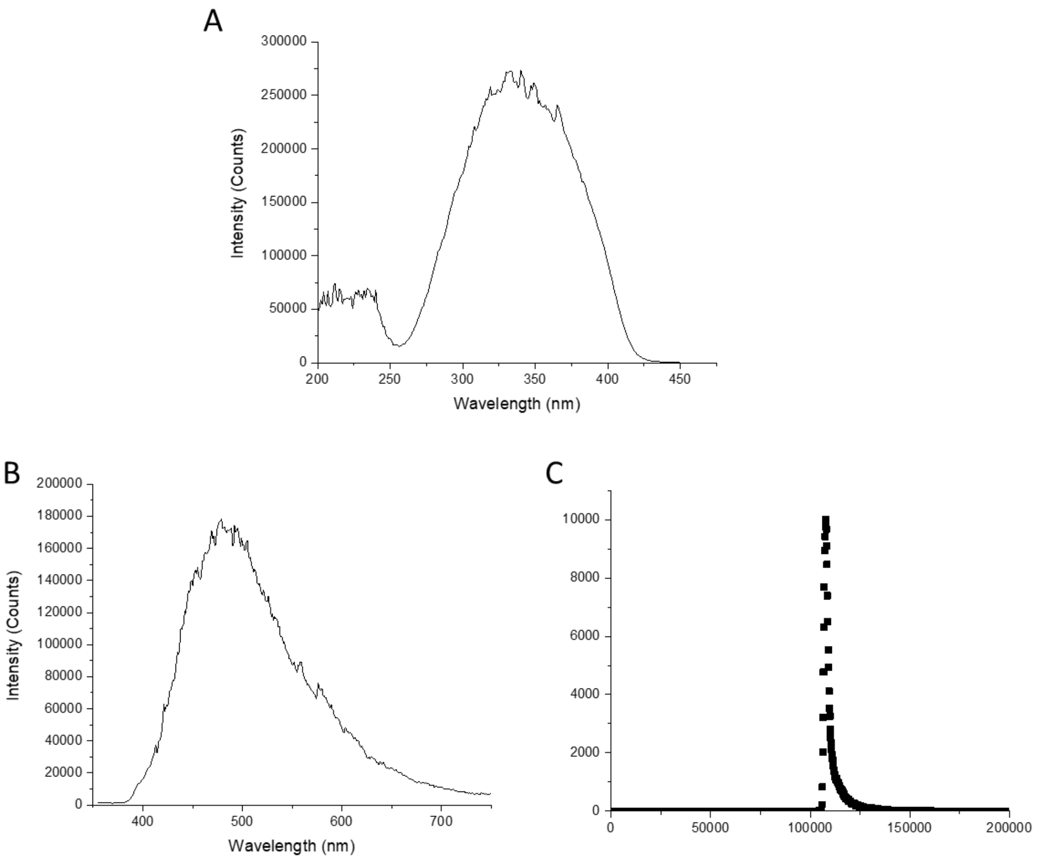

Supplementary Figure 103. Luminescent data of $[Gd_2(\mathbf{L3}^{SS})_6](OTf)_{12}$ (2.55 x 10^{-5} M in 1:4 of MeOH/EtOH at 77K).(A) Excitation spectrum, λ_{em} = 480 nm, slits = 2.0-0.5, filter 380 nm. (b) Emission spectrum, λ_{ex} = 345 nm, slits = 2.0-0.5, filter 380 nm. (c) Excited state decay curve, λ_{em} = 480 nm, filter 380 nm.

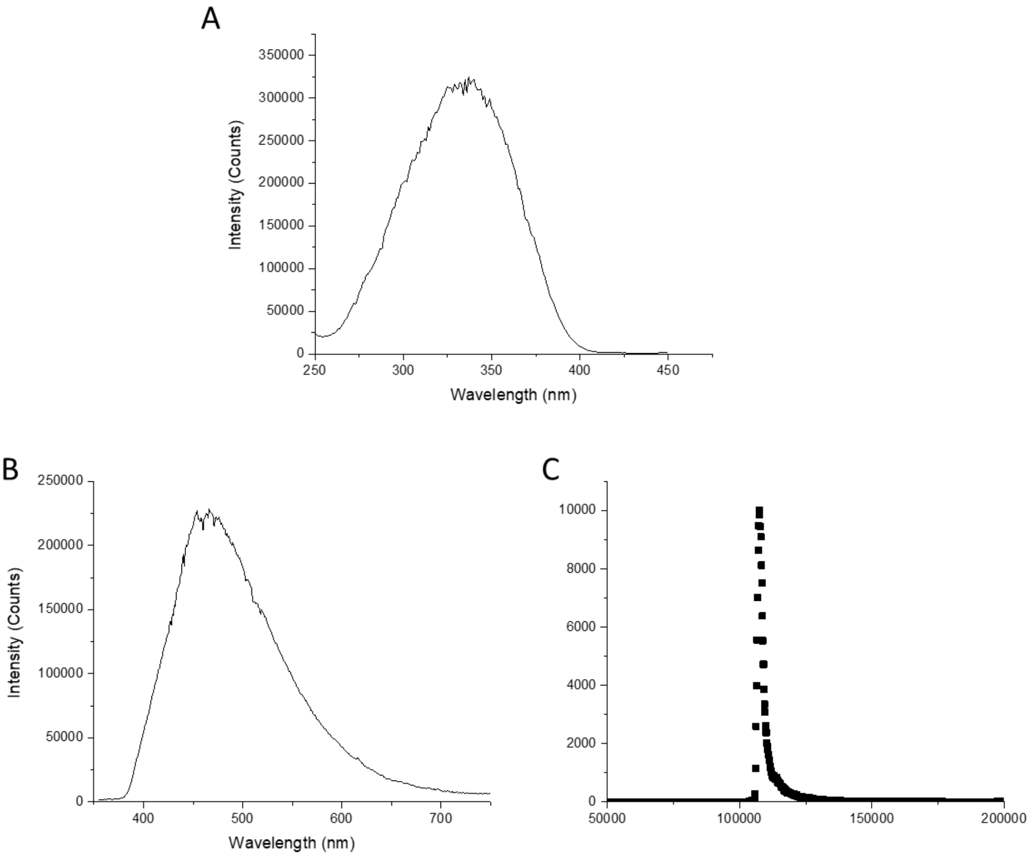

Supplementary Figure 104. Luminescent data of $[Gd_4(\mathbf{L1}^{\mathbf{RR}})_6](OTf)_{12}$ (9.05 x 10^{-6} M in 1:4 of MeOH/EtOH at 77K).(A) Excitation spectrum, λ_{em} = 466 nm, slits = 2.0-0.5, filter 380 nm. (b) Emission spectrum, λ_{ex} = 330 nm, slits = 2.0-0.5, filter 380 nm. (c) Excited state decay curve, λ_{em} = 466 nm, filter 380 nm.

	λ_{abs}^{max} (nm)	ε^{max} ($L \cdot mol^{-1} \cdot cm^{-1}$)	λ_{em}^{max} (nm)	φ_x^{b} (%)	τ (ms)
$[La_2($**L1**$^{SS})_3](CF_3SO_3)_6$	311	59052.4	/	/	/
$[La_2($**L1**$^{RR})_3](CF_3SO_3)_6$	311	58787.3	/	/	/
$[Sm_2($**L1**$^{SS})_3](CF_3SO_3)_6$	311	60056.4	/	/	/
$[Sm_2($**L1**$^{RR})_3](CF_3SO_3)_6$	311	62036.6	/	/	/
$[Eu_2($**L1**$^{SS})_3](CF_3SO_3)_6$	311	62925.5	616	4.07 (0.015)	1.42
$[Eu_2($**L1**$^{RR})_3](CF_3SO_3)_6$	311	61958.6	616	4.41 (0.005)	1.41
$[Gd_2($**L1**$^{SS})_3](CF_3SO_3)_6$	311	58396.8	462	/	0.0073
$[Gd_2($**L1**$^{RR})_3](CF_3SO_3)_6$	311	59227.0	/	/	/
$[Tb_2($**L1**$^{SS})_3](CF_3SO_3)_6$	311	61469.6	/	/	/
$[Tb_2($**L1**$^{RR})_3](CF_3SO_3)_6$	311	60113.3	/	/	/
$[Lu_2($**L1**$^{SS})_3](CF_3SO_3)_6$	311	60668.1	/	/	/
$[Lu_2($**L1**$^{RR})_3](CF_3SO_3)_6$	311	59773.5	/	/	/
$[Sm_4($**L1**$^{SS})_6](CF_3SO_3)_{12}$	330	130019.8	/	/	/
$[Sm_4($**L1**$^{RR})_6](CF_3SO_3)_{12}$	330	139354.6	/	/	/
$[Eu_4($**L1**$^{SS})_6](CF_3SO_3)_{12}$	330	123947.0	616	0.81 (0.035)	1.28
$[Eu_4($**L1**$^{RR})_6](CF_3SO_3)_{12}$	330	121335.7	616	0.83 (0.035)	1.27
$[Gd_4($**L1**$^{SS})_6](CF_3SO_3)_{12}$	330	123657.1	/	/	/
$[Gd_4($**L1**$^{RR})_6](CF_3SO_3)_{12}$	330	121829.6	466	/	0.0087[c]
$[Tb_4($**L1**$^{SS})_6](CF_3SO_3)_{12}$	330	131075.9	/	/	/
$[Tb_4($**L1**$^{RR})_6](CF_3SO_3)_{12}$	330	127713.5	/	/	/
$[Lu_4($**L1**$^{SS})_6](CF_3SO_3)_{12}$	330	110557.9	/	/	/
$[Lu_4($**L1**$^{RR})_6](CF_3SO_3)_{12}$	330	112691.3	/	/	/

Supplementary Table 3. A summary of selected photophysical properties, UV-Vis absorption and luminescence data of $[Ln_2$**L1**$_3]$ and $[Ln_4$**L1**$_6]$ complexes in acetonitrile solution[a]. [a]using a 10 mm cuvette and filter 380 nm. [b]The relative quantum yields were referenced with quinine sulfate in 0.1 M sulfuric acid (φ= 0.577, λ_{ex} = 350nm) with 10 mm cuvette. [c]Measurement performed at 77K in 1:4 MeOH/EtOH.

	λ_{abs}^{max} (nm)	ε^{max} ($L \cdot mol^{-1} \cdot cm^{-1}$)
$[La_2(\mathbf{L3^{SS}})_3](CF_3SO_3)_6$	345	125117.0
$[La_2(\mathbf{L3^{RR}})_3](CF_3SO_3)_6$	345	128419.6
$[Sm_2(\mathbf{L3^{SS}})_3](CF_3SO_3)_6$	345	104030.4
$[Sm_2(\mathbf{L3^{RR}})_3](CF_3SO_3)_6$	345	104038.3
$[Eu_2(\mathbf{L3^{SS}})_3](CF_3SO_3)_6$	345	124128.9
$[Eu_2(\mathbf{L3^{RR}})_3](CF_3SO_3)_6$	345	125331.4
$[Gd_2(\mathbf{L3^{SS}})_3](CF_3SO_3)_6$	345	108324.9
$[Gd_2(\mathbf{L3^{RR}})_3](CF_3SO_3)_6$	345	115018.7
$[Tb_2(\mathbf{L3^{SS}})_3](CF_3SO_3)_6$	345	114213.3
$[Tb_2(\mathbf{L3^{RR}})_3](CF_3SO_3)_6$	345	119793.5
$[Lu_2(\mathbf{L3^{SS}})_3](CF_3SO_3)_6$	345	117521.9
$[Lu_2(\mathbf{L3^{RR}})_3](CF_3SO_3)_6$	345	115357.7

Supplementary Table 4. A summary of selected photophysical properties, UV-Vis absorption and luminescence data of $[Ln_2\mathbf{L3}_3]$ in acetonitrile solution using a 10 mm cuvette and filter 380 nm.

CPL measurements

	g_{lum}			
	$^5D_0$ -> $^7F_1$	$^5D_0$ -> $^7F_2$	$^5D_0$ -> $^7F_4$	$^5D_0$ -> $^7F_4$
	ΔJ = 1	ΔJ = 2	ΔJ = 4	ΔJ = 4
Complex	(592.5 nm)	(615 nm)	(695.5 nm)	(704 nm)
$[Eu_2(\mathbf{L1^{SS}})_3]$	+0.10	-0.02	—	—
$[Eu_2(\mathbf{L1^{RR}})_3]$	- 0.10	+0.02	—	—
$[Eu_4(\mathbf{L1^{SS}})_6]$	+0.10	-0.02	-0.05	+0.23
$[Eu_4(\mathbf{L1^{RR}})_6]$	- 0.08	+0.02	+0.04	-0.23

*All g_{lum} values have associated nominal assumed instrumental uncertainty of ± 0.01.

Supplementary Table 5. g_{lum} values for $[Eu_2(\mathbf{L1^{SS/RR}})_3]$ and $[Eu_4(\mathbf{L1^{SS/RR}})_6]$ calculated from total intensity and CPL spectra.

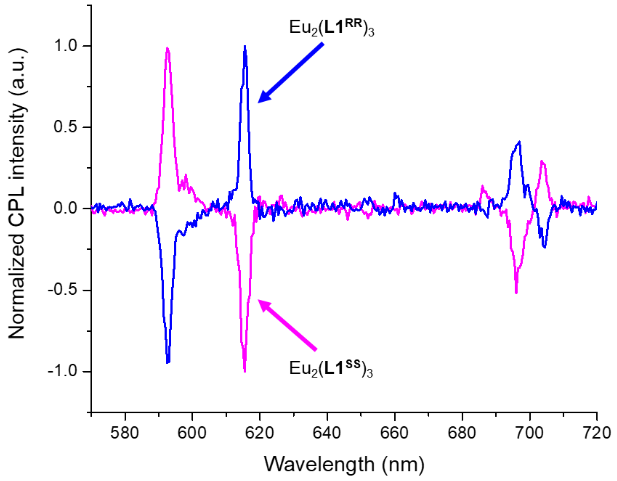

Supplementary Figure 105. Normalized CPL spectra and Eu_2(**L1**^{SS})$_3$ and Eu_2(**L1**^{RR})$_3$.

X-ray crystallography data

Crystal structure of Tb_2(L2^{SS})$_3$:

Crystal data for Tb_2(**L2**^{SS})$_3$: *formula*, M_r = 2372.04, *crystal system*, space group P 2_1 2_1 2, Z = 4, a = 26.075(3) Å, b = 28.363(4)Å, c = 23.396(3)Å, α =90°, β = 90°, γ = 90°, V = 17304(4) Å^3, μ(Mo$_{K\alpha}$) = 0.857 mm^{-1}, ρ_{calc} = 0.911 mgm^{-3}, T = 296(2) K. The crystallographic data for the structural analyses have been deposited with the Cambridge Crystallographic Data Centre, CCDC No. 1009616, and the data can be obtained free of charge via www.ccdc.cam.ac.uk/data_request/cif.

Information	Identifier from cif file
Empirical formula	$C_{126}H_{96}N_{18}O_{12}Tb_2$
Formula weight	2372.04
Temperature/K	296(2)
Crystal system	orthorhombic
Space group	$P2_12_12$
a/Å	26.075(3)
b/Å	28.363(4)
c/Å	23.396(3)
α/°	90
β/°	90
γ/°	90
Volume/Å^3	17304(4)
Z	4
ρ_{calc}g/cm^3	0.911
μ/mm^{-1}	0.857
F(000)	4816.0
Crystal size/mm^3	0.480 × 0.320 × 0.200
Radiation	MoKα (λ = 0.71073)
2Θ range for data collection/°	5.202 to 50.7
Index ranges	$-29 \le h \le 31$, $-34 \le k \le 29$, $-28 \le l \le 28$
Reflections collected	210003

Independent reflections	31661 [R_{int} = 0.0893, R_{sigma} = 0.0808]
Data/restraints/parameters	31661/17/1363
Goodness-of-fit on F^2	0.941
Final R indexes [I>=2σ (I)]	R_1 = 0.0524, wR_2 = 0.1159
Final R indexes [all data]	R_1 = 0.0774, wR_2 = 0.1240
Largest diff. peak/hole / e Å^{-3}	0.59/-0.33
Flack parameter	0.002(5)

Tb_2(**L2**^{SS})$_3$	Shape measure (º)	
	Tricapped trigonal prism (D_{3h})	Capped square antiprism (C_{4v})
Tb1	7.04	10.89
Tb2	6.77	10.43

Supplementary Table 6. Results of the Shape Analysis for Tb_2(**L2**^{SS})$_3$ helix.

The coordination geometry of the Tb_2(**L2**^{SS})$_3$: helix can be best described as tricapped trigonal prism.

Crystal structure of Eu_4(L1^{SS})$_6$:

The crystallographic data for the structural analyses have been deposited with the Cambridge Crystallographic Data Centre, CCDC No. 2014348.

Information	Identifier from cif file
Empirical formula	$C_{225}H_{192}Eu_4F_{27}N_{36}O_{51}S_9$
Formula weight	5625.51
Temperature/K	220.0
Crystal system	trigonal
Space group	R3
a/Å	29.9510(4)
b/Å	29.9510(4)
c/Å	71.8428(16)
α/°	90
β/°	90
γ/°	120
Volume/Å^3	55813.1(19)
Z	6
ρ_{calc}g/cm^3	1.004
μ/mm^{-1}	5.808
F(000)	17046.0
Crystal size/mm^3	0.24 × 0.17 × 0.14
Radiation	CuKα (λ = 1.54178)
2Θ range for data collection/°	5.902 to 100.82
Index ranges	-28 ≤ h ≤ 27, -27 ≤ k ≤ 29, -69 ≤ l ≤ 71
Reflections collected	41125
Independent reflections	20620 [R_{int} = 0.0639, R_{sigma} = 0.0648]
Data/restraints/parameters	20620/1946/2017
Goodness-of-fit on F^2	1.055

Final R indexes [I>=2σ (I)]	R_1 = 0.0553, wR_2 = 0.1497
Final R indexes [all data]	R_1 = 0.0685, wR_2 = 0.1594
Largest diff. peak/hole / e Å^{-3}	0.66/-0.18
Flack parameter	0.030(4)

Eu_4(**L1**^{SS})$_6$	Shape measure (°)	
	Tricapped trigonal prism (D_{3h})	Capped square antiprism (C_{4v})
Eu1	6.45	11.13
Eu2	5.82	10.33
Eu3	5.94	10.23
Eu4	6.11	10.93

Supplementary Table 7. Results of the Shape Analysis for Eu_4(**L1**^{SS})$_6$ tetrahedron.

The coordination geometry of the Eu_4(**L1**^{SS})$_6$ tetrahedron can be best described as tricapped trigonal prism.

Supplementary References

1. Yeung, C.-T. et al. Lanthanide supramolecular helical diastereoselective breaking induced by point chirality: mixture or P-helix, M-helix. *Chem. Commun.* **51**, 592-595 (2014).
2. Krause, L. et al. Comparison of silver and molybdenum microfocus X-ray sources for single-crystal structure determination. *J. Appl. Cryst.* **48**, 3-10 (2015).
3. Bruker-AXS, APEX3 Software Suite. Madison, Wisconsin, USA, (2014)
4. Hübschle, C. B., Sheldrick, G. M. & Dittrich, B. *ShelXle*: a Qt graphical user interface for *SHELXL*. *J. Appl. Cryst.* **44**, 1281-1284 (2011).
