# Supplementary material for: Helicate-to-tetrahedron transformation of chiral lanthanide supramolecular complexes induced by ionic radii effect and linker length
Source: Commun Chem. 2021 Aug 5;4:116. doi: 10.1038/s42004-021-00553-8 (PMC9814731; doi:10.1038/s42004-021-00553-8)
Supplement: Supplementary file 2 — Description of Additional Supplementary Files [file 42004_2021_553_MOESM2_ESM.pdf]

Description of Additional Supplementary Files

File Name: Supplementary Data 1

Description: X-ray crystallography data of $Tb_2(\mathbf{L2}^{SS})_3$

File Name: Supplementary Data 2

Description: X-ray crystallography data of $Eu_4(\mathbf{L1}^{SS})_6$
