# Supplementary material for: Helicate-to-tetrahedron transformation of chiral lanthanide supramolecular complexes induced by ionic radii effect and linker length
Source: Commun Chem. 2021 Aug 5;4:116. doi: 10.1038/s42004-021-00553-8 (PMC9814731; doi:10.1038/s42004-021-00553-8)

checkCIF/PLATON report

Structure factors have been supplied for datablock(s) missing_3_triflates_sq

THIS REPORT IS FOR GUIDANCE ONLY. IF USED AS PART OF A REVIEW PROCEDURE FOR PUBLICATION, IT SHOULD NOT REPLACE THE EXPERTISE OF AN EXPERIENCED CRYSTALLOGRAPHIC REFEREE.

No syntax errors found. CIF dictionary Interpreting this report

Datablock: missing_3_triflates_sq

Bond precision: C-C = 0.0372 A Wavelength=1.54178

Cell:	a=29.9510(4)	b=29.9510(4)	c=71.8428(16)
	alpha=90	beta=90	gamma=120
Temperature:	220 K		

	Calculated	Reported
Volume	55813.2(19)	55813.1(19)
Space group	R 3	R 3
Hall group	R 3	R 3
Moiety formula	C216 H192 Eu4 N36 O24, 9(C F3 O3 S) [+ solvent]	C216 H192 Eu4 N36 O24, 9(C F3 O3 S)
Sum formula	C225 H192 Eu4 F27 N36 O51 S9 [+ solvent]	C225 H192 Eu4 F27 N36 O51 S9
Mr	5625.57	5625.51
Dx,g cm-3	1.004	1.004
Z	6	6
Mu (mm-1)	5.808	5.808
F000	17046.0	17046.0
F000’	16907.71	
h,k,lmax	29,29,71	28,29,71
Nref	25972[12986]	20620
Tmin,Tmax	0.352,0.443	0.546,0.753
Tmin’	0.216	

Correction method= # Reported T Limits: Tmin=0.546 Tmax=0.753
AbsCorr = MULTI-SCAN

Data completeness= 1.59/0.79 Theta(max)= 50.410

R(reflections)= 0.0553(17843) wR2(reflections)= 0.1594(20620)

S = 1.055 Npar= 2017

The following ALERTS were generated. Each ALERT has the format
test-name_ALERT_alert-type_alert-level.
Click on the hyperlinks for more details of the test.

Alert level A

```
SHFSU01_ALERT_2_A  The absolute value of parameter shift to su ratio > 0.20
            Absolute value of the parameter shift to su ratio given   0.871
            Additional refinement cycles may be required.
```

Author Response: There is considerable displacement of many atoms due to the quality of the data being affected by gradual solvent loss and associated atomic displacements during the data collection. This has resulted in some regions of poorly defined electron density that give rise to oscillations in the least squares minimisations. These fluctuations have little impact on the final model and could be removed by restricting the model further, however the inclusion of this final shift level indicates the true nature of the model and some of its limitations.

```
THETM01_ALERT_3_A  The value of sine(theta_max)/wavelength is less than 0.550
            Calculated sin(theta_max)/wavelength =    0.4998
```

Author Response: Gradual solvent loss throughout the data collection limited the useable diffraction resolution

```
PLAT080_ALERT_2_A Maximum Shift/Error ............................       0.87 Why ?
```

Author Response: see _vrf_SHFSU01_missing_3_triflates_sq

```
PLAT241_ALERT_2_A High   'MainMol' Ueq as Compared to Neighbors of       C20A Check
```

Author Response: The high level of certain anisotropic displacement parameters is due to the gradual solvent loss through the data collection that enables localised atomic movement throughout the structure.

```
PLAT241_ALERT_2_A High   'MainMol' Ueq as Compared to Neighbors of       C17D Check
```

Author Response: The high level of certain anisotropic displacement parameters is due to the gradual solvent loss through the data collection that enables localised atomic movement throughout the structure.

Alert level B

```
PLAT220_ALERT_2_B NonSolvent   Resd 1  C   Ueq(max)/Ueq(min) Range        7.0 Ratio
```

Author Response: The high level of certain anisotropic displacement parameters is due to the gradual solvent loss through the data collection that enables localised atomic movement throughout the structure.

PLAT220_ALERT_2_B NonSolvent   Resd 2 C   Ueq(max)/Ueq(min) Range   6.4 Ratio

Author Response: The high level of certain anisotropic displacement parameters is due to the gradual solvent loss through the data collection that enables localised atomic movement throughout the structure.

PLAT241_ALERT_2_B High   'MainMol' Ueq as Compared to Neighbors of   C5A Check

Author Response: The high level of certain anisotropic displacement parameters is due to the gradual solvent loss through the data collection that enables localised atomic movement throughout the structure.

PLAT241_ALERT_2_B High   'MainMol' Ueq as Compared to Neighbors of   C7B Check

Author Response: The high level of certain anisotropic displacement parameters is due to the gradual solvent loss through the data collection that enables localised atomic movement throughout the structure.

PLAT241_ALERT_2_B High   'MainMol' Ueq as Compared to Neighbors of   C34A Check

Author Response: The high level of certain anisotropic displacement parameters is due to the gradual solvent loss through the data collection that enables localised atomic movement throughout the structure.

PLAT241_ALERT_2_B High   'MainMol' Ueq as Compared to Neighbors of   C34B Check

Author Response: The high level of certain anisotropic displacement parameters is due to the gradual solvent loss through the data collection that enables localised atomic movement throughout the structure.

PLAT241_ALERT_2_B High   'MainMol' Ueq as Compared to Neighbors of   C6C Check

Author Response: The high level of certain anisotropic displacement parameters is due to the gradual solvent loss through the data collection that enables localised atomic movement throughout the structure.

PLAT241_ALERT_2_B High   'MainMol' Ueq as Compared to Neighbors of   C34D Check

Author Response: The high level of certain anisotropic displacement parameters is due to the gradual solvent loss through the data collection that enables localised atomic movement throughout the structure.

PLAT242_ALERT_2_B Low   'MainMol' Ueq as Compared to Neighbors of   C8A Check

Author Response: The high level of certain anisotropic displacement parameters is due to the gradual solvent loss through the data collection that enables localised atomic movement throughout the structure. This has in come cases resulted in localised deviations af neighbouring atom displacement parameter ratios

```
PLAT242_ALERT_2_B Low    'MainMol' Ueq as Compared to Neighbors of       C19A Check
```

Author Response: The high level of certain anisotropic displacement parameters is due to the gradual solvent loss through the data collection that enables localised atomic movement throughout the structure. This has in come cases resulted in localised deviations af neighbouring atom displacement parameter ratios

```
PLAT242_ALERT_2_B Low    'MainMol' Ueq as Compared to Neighbors of       C21A Check
```

Author Response: The high level of certain anisotropic displacement parameters is due to the gradual solvent loss through the data collection that enables localised atomic movement throughout the structure. This has in come cases resulted in localised deviations af neighbouring atom displacement parameter ratios

```
PLAT242_ALERT_2_B Low    'MainMol' Ueq as Compared to Neighbors of       C16D Check
```

Author Response: The high level of certain anisotropic displacement parameters is due to the gradual solvent loss through the data collection that enables localised atomic movement throughout the structure. This has in come cases resulted in localised deviations af neighbouring atom displacement parameter ratios

```
PLAT242_ALERT_2_B Low    'MainMol' Ueq as Compared to Neighbors of       C31D Check
```

Author Response: The high level of certain anisotropic displacement parameters is due to the gradual solvent loss through the data collection that enables localised atomic movement throughout the structure. This has in come cases resulted in localised deviations af neighbouring atom displacement parameter ratios

```
PLAT242_ALERT_2_B Low    'MainMol' Ueq as Compared to Neighbors of       C36D Check
```

Author Response: The high level of certain anisotropic displacement parameters is due to the gradual solvent loss through the data collection that enables localised atomic movement throughout the structure. This has in come cases resulted in localised deviations af neighbouring atom displacement parameter ratios

```
PLAT260_ALERT_2_B Large Average Ueq of Residue Including       S1U      0.346 Check
```

Author Response: The high level of certain anisotropic displacement parameters is due to the gradual solvent loss through the data collection that enables localised atomic movement throughout the structure.

```
PLAT260_ALERT_2_B Large Average Ueq of Residue Including       S1V      0.351 Check
```

Author Response: The high level of certain anisotropic displacement parameters is due to the gradual solvent loss through the data collection that enables localised atomic movement throughout the structure.

```
PLAT260_ALERT_2_B Large Average Ueq of Residue Including       S1X      0.349 Check
```

Author Response: The high level of certain anisotropic displacement parameters is due to the gradual solvent loss through the data collection that enables localised atomic movement throughout the structure.

```
PLAT260_ALERT_2_B Large Average Ueq of Residue Including       S1Y      0.392 Check
```

Author Response: The high level of certain anisotropic displacement parameters is due to the gradual solvent loss through the data collection that enables localised atomic movement throughout the structure.

```
PLAT342_ALERT_3_B Low Bond Precision on  C-C Bonds ..............    0.03718 Ang.
```

Author Response: The high level of certain anisotropic displacement parameters is due to the gradual solvent loss through the data collection that enables localised atomic movement throughout the structure. This has resulted in a general reduction in the confidence level of bond lengths throughout.

```
PLAT987_ALERT_1_B The Flack x is >> 0 -  Do a BASF/TWIN Refinement     Please Check
```

Author Response: Due to the data treatment methodology, any interpretation of absolute structure from the crystallographic data alone should be treated with significant levels of caution

---

Alert level C

```
PLAT029_ALERT_3_C _diffrn_measured_fraction_theta_full value Low .      0.976 Why?
PLAT090_ALERT_3_C Poor Data / Parameter Ratio (Zmax > 18) ........       6.28 Note
PLAT094_ALERT_2_C Ratio of Maximum / Minimum Residual Density ....       3.62 Report
PLAT213_ALERT_2_C Atom C5A             has ADP max/min Ratio .....        3.2 prolat
PLAT213_ALERT_2_C Atom C6A             has ADP max/min Ratio .....        3.4 prolat
PLAT213_ALERT_2_C Atom C20A            has ADP max/min Ratio .....        3.2 prolat
PLAT213_ALERT_2_C Atom C29B            has ADP max/min Ratio .....        3.1 prolat
PLAT213_ALERT_2_C Atom C30B            has ADP max/min Ratio .....        3.8 prolat
PLAT213_ALERT_2_C Atom C1C             has ADP max/min Ratio .....        3.1 prolat
```

```
PLAT213_ALERT_2_C Atom C17D            has ADP max/min Ratio .....        3.5 prolat
PLAT222_ALERT_3_C NonSolvent Resd 1  H   Uiso(max)/Uiso(min) Range        5.8 Ratio
PLAT222_ALERT_3_C NonSolvent Resd 2  H   Uiso(max)/Uiso(min) Range        5.3 Ratio
PLAT241_ALERT_2_C High   'MainMol' Ueq as Compared to Neighbors of        N1B Check
```

Author Response: The high level of certain anisotropic displacement parameters is due to the gradual solvent loss through the data collection that enables localised atomic movement throughout the structure.

```
PLAT241_ALERT_2_C High   'MainMol' Ueq as Compared to Neighbors of        N3A Check
```

Author Response: The high level of certain anisotropic displacement parameters is due to the gradual solvent loss through the data collection that enables localised atomic movement throughout the structure.

```
PLAT241_ALERT_2_C High   'MainMol' Ueq as Compared to Neighbors of        N4A Check
```

Author Response: The high level of certain anisotropic displacement parameters is due to the gradual solvent loss through the data collection that enables localised atomic movement throughout the structure.

```
PLAT241_ALERT_2_C High   'MainMol' Ueq as Compared to Neighbors of        C6A Check
```

Author Response: The high level of certain anisotropic displacement parameters is due to the gradual solvent loss through the data collection that enables localised atomic movement throughout the structure.

```
PLAT241_ALERT_2_C High   'MainMol' Ueq as Compared to Neighbors of        C7A Check
```

Author Response: The high level of certain anisotropic displacement parameters is due to the gradual solvent loss through the data collection that enables localised atomic movement throughout the structure.

```
PLAT241_ALERT_2_C High   'MainMol' Ueq as Compared to Neighbors of       C12A Check
```

Author Response: The high level of certain anisotropic displacement parameters is due to the gradual solvent loss through the data collection that enables localised atomic movement throughout the structure.

```
PLAT241_ALERT_2_C High   'MainMol' Ueq as Compared to Neighbors of       C12B Check
```

Author Response: The high level of certain anisotropic displacement parameters is due to the gradual solvent loss through the data collection that enables localised atomic movement throughout the structure.

```
PLAT241_ALERT_2_C High   'MainMol' Ueq as Compared to Neighbors of       C13A Check
```

Author Response: The high level of certain anisotropic displacement parameters is due to the gradual solvent loss through the data collection that enables localised atomic movement throughout the structure.

```
PLAT241_ALERT_2_C High   ’MainMol’ Ueq as Compared to Neighbors of       C18A Check
```

Author Response: The high level of certain anisotropic displacement parameters is due to the gradual solvent loss through the data collection that enables localised atomic movement throughout the structure.

```
PLAT241_ALERT_2_C High   ’MainMol’ Ueq as Compared to Neighbors of       C20B Check
```

Author Response: The high level of certain anisotropic displacement parameters is due to the gradual solvent loss through the data collection that enables localised atomic movement throughout the structure.

```
PLAT241_ALERT_2_C High   ’MainMol’ Ueq as Compared to Neighbors of       C21B Check
```

Author Response: The high level of certain anisotropic displacement parameters is due to the gradual solvent loss through the data collection that enables localised atomic movement throughout the structure.

```
PLAT241_ALERT_2_C High   ’MainMol’ Ueq as Compared to Neighbors of       C25B Check
```

Author Response: The high level of certain anisotropic displacement parameters is due to the gradual solvent loss through the data collection that enables localised atomic movement throughout the structure.

```
PLAT241_ALERT_2_C High   ’MainMol’ Ueq as Compared to Neighbors of       C32B Check
```

Author Response: The high level of certain anisotropic displacement parameters is due to the gradual solvent loss through the data collection that enables localised atomic movement throughout the structure.

```
PLAT241_ALERT_2_C High   ’MainMol’ Ueq as Compared to Neighbors of        N1D Check
```

Author Response: The high level of certain anisotropic displacement parameters is due to the gradual solvent loss through the data collection that enables localised atomic movement throughout the structure.

```
PLAT241_ALERT_2_C High   ’MainMol’ Ueq as Compared to Neighbors of        N3D Check
```

Author Response: The high level of certain anisotropic displacement parameters is due to the gradual solvent loss through the data collection that enables localised atomic movement throughout the structure.

PLAT241_ALERT_2_C High 'MainMol' Ueq as Compared to Neighbors of N4D Check

Author Response: The high level of certain anisotropic displacement parameters is due to the gradual solvent loss through the data collection that enables localised atomic movement throughout the structure.

PLAT241_ALERT_2_C High 'MainMol' Ueq as Compared to Neighbors of C6D Check

Author Response: The high level of certain anisotropic displacement parameters is due to the gradual solvent loss through the data collection that enables localised atomic movement throughout the structure.

PLAT241_ALERT_2_C High 'MainMol' Ueq as Compared to Neighbors of C8C Check

Author Response: The high level of certain anisotropic displacement parameters is due to the gradual solvent loss through the data collection that enables localised atomic movement throughout the structure.

PLAT241_ALERT_2_C High 'MainMol' Ueq as Compared to Neighbors of C11D Check

Author Response: The high level of certain anisotropic displacement parameters is due to the gradual solvent loss through the data collection that enables localised atomic movement throughout the structure.

PLAT241_ALERT_2_C High 'MainMol' Ueq as Compared to Neighbors of C12C Check

Author Response: The high level of certain anisotropic displacement parameters is due to the gradual solvent loss through the data collection that enables localised atomic movement throughout the structure.

PLAT241_ALERT_2_C High 'MainMol' Ueq as Compared to Neighbors of C21C Check

Author Response: The high level of certain anisotropic displacement parameters is due to the gradual solvent loss through the data collection that enables localised atomic movement throughout the structure.

PLAT241_ALERT_2_C High 'MainMol' Ueq as Compared to Neighbors of C21D Check

Author Response: The high level of certain anisotropic displacement parameters is due to the gradual solvent loss through the data collection that enables localised atomic movement throughout the structure.

PLAT241_ALERT_2_C High 'MainMol' Ueq as Compared to Neighbors of C25C Check

Author Response: The high level of certain anisotropic displacement parameters is due to the gradual solvent loss through the data collection that enables localised atomic movement throughout the structure.

PLAT241_ALERT_2_C High 'MainMol' Ueq as Compared to Neighbors of C25D Check

Author Response: The high level of certain anisotropic displacement parameters is due to the gradual solvent loss through the data collection that enables localised atomic movement throughout the structure.

PLAT241_ALERT_2_C High 'MainMol' Ueq as Compared to Neighbors of C33D Check

Author Response: The high level of certain anisotropic displacement parameters is due to the gradual solvent loss through the data collection that enables localised atomic movement throughout the structure.

PLAT241_ALERT_2_C High 'MainMol' Ueq as Compared to Neighbors of C35C Check

Author Response: The high level of certain anisotropic displacement parameters is due to the gradual solvent loss through the data collection that enables localised atomic movement throughout the structure.

PLAT241_ALERT_2_C High 'MainMol' Ueq as Compared to Neighbors of C35D Check

Author Response: The high level of certain anisotropic displacement parameters is due to the gradual solvent loss through the data collection that enables localised atomic movement throughout the structure.

PLAT241_ALERT_2_C High 'MainMol' Ueq as Compared to Neighbors of C36C Check

Author Response: The high level of certain anisotropic displacement parameters is due to the gradual solvent loss through the data collection that enables localised atomic movement throughout the structure.

PLAT242_ALERT_2_C Low 'MainMol' Ueq as Compared to Neighbors of Eu2 Check

Author Response: The high level of certain anisotropic displacement parameters is due to the gradual solvent loss through the data collection that enables localised atomic movement throughout the structure. This has in come cases resulted in localised deviations af neighbouring atom displacement parameter ratios

PLAT242_ALERT_2_C Low 'MainMol' Ueq as Compared to Neighbors of C2A Check

Author Response: The high level of certain anisotropic displacement parameters is due to the gradual solvent loss through the data collection that enables localised atomic movement throughout the structure. This has in come cases resulted in localised deviations af neighbouring atom displacement parameter ratios

PLAT242_ALERT_2_C Low 'MainMol' Ueq as Compared to Neighbors of C2B Check

Author Response: The high level of certain anisotropic displacement parameters is due to the gradual solvent loss through the data collection that enables localised atomic movement throughout the structure. This has in come cases resulted in localised deviations af neighbouring atom displacement parameter ratios

PLAT242_ALERT_2_C Low 'MainMol' Ueq as Compared to Neighbors of C3A Check

Author Response: The high level of certain anisotropic displacement parameters is due to the gradual solvent loss through the data collection that enables localised atomic movement throughout the structure. This has in come cases resulted in localised deviations af neighbouring atom displacement parameter ratios

PLAT242_ALERT_2_C Low 'MainMol' Ueq as Compared to Neighbors of C3B Check

Author Response: The high level of certain anisotropic displacement parameters is due to the gradual solvent loss through the data collection that enables localised atomic movement throughout the structure. This has in come cases resulted in localised deviations af neighbouring atom displacement parameter ratios

PLAT242_ALERT_2_C Low 'MainMol' Ueq as Compared to Neighbors of C8B Check

Author Response: The high level of certain anisotropic displacement parameters is due to the gradual solvent loss through the data collection that enables localised atomic movement throughout the structure. This has in come cases resulted in localised deviations af neighbouring atom displacement parameter ratios

PLAT242_ALERT_2_C Low 'MainMol' Ueq as Compared to Neighbors of C11A Check

Author Response: The high level of certain anisotropic displacement parameters is due to the gradual solvent loss through the data collection that enables localised atomic movement throughout the structure. This has in come cases resulted in localised deviations af neighbouring atom displacement parameter ratios

PLAT242_ALERT_2_C Low 'MainMol' Ueq as Compared to Neighbors of C16A Check

Author Response: The high level of certain anisotropic displacement parameters is due to the gradual solvent loss through the data collection that enables localised atomic movement throughout the structure. This has in come cases resulted in localised deviations af neighbouring atom displacement parameter ratios

PLAT242_ALERT_2_C Low 'MainMol' Ueq as Compared to Neighbors of C19B Check

Author Response: The high level of certain anisotropic displacement parameters is due to the gradual solvent loss through the data collection that enables localised atomic movement throughout the structure. This has in come cases resulted in localised deviations af neighbouring atom displacement parameter ratios

PLAT242_ALERT_2_C Low 'MainMol' Ueq as Compared to Neighbors of C29A Check

Author Response: The high level of certain anisotropic displacement parameters is due to the gradual solvent loss through the data collection that enables localised atomic movement throughout the structure. This has in come cases resulted in localised deviations af neighbouring atom displacement parameter ratios

PLAT242_ALERT_2_C Low 'MainMol' Ueq as Compared to Neighbors of C29B Check

Author Response: The high level of certain anisotropic displacement parameters is due to the gradual solvent loss through the data collection that enables localised atomic movement throughout the structure. This has in come cases resulted in localised deviations af neighbouring atom displacement parameter ratios

PLAT242_ALERT_2_C Low 'MainMol' Ueq as Compared to Neighbors of C31B Check

Author Response: The high level of certain anisotropic displacement parameters is due to the gradual solvent loss through the data collection that enables localised atomic movement throughout the structure. This has in come cases resulted in localised deviations af neighbouring atom displacement parameter ratios

PLAT242_ALERT_2_C Low 'MainMol' Ueq as Compared to Neighbors of C35A Check

Author Response: The high level of certain anisotropic displacement parameters is due to the gradual solvent loss through the data collection that enables localised atomic movement throughout the structure. This has in come cases resulted in localised deviations af neighbouring atom displacement parameter ratios

PLAT242_ALERT_2_C Low 'MainMol' Ueq as Compared to Neighbors of C36B Check

Author Response: The high level of certain anisotropic displacement parameters is due to the gradual solvent loss through the data collection that enables localised atomic movement throughout the structure. This has in come cases resulted in localised deviations af neighbouring atom displacement parameter ratios

```
PLAT242_ALERT_2_C Low    'MainMol' Ueq as Compared to Neighbors of        Eu3 Check
```

Author Response: The high level of certain anisotropic displacement parameters is due to the gradual solvent loss through the data collection that enables localised atomic movement throughout the structure. This has in come cases resulted in localised deviations af neighbouring atom displacement parameter ratios

```
PLAT242_ALERT_2_C Low    'MainMol' Ueq as Compared to Neighbors of        C2D Check
```

Author Response: The high level of certain anisotropic displacement parameters is due to the gradual solvent loss through the data collection that enables localised atomic movement throughout the structure. This has in come cases resulted in localised deviations af neighbouring atom displacement parameter ratios

```
PLAT242_ALERT_2_C Low    'MainMol' Ueq as Compared to Neighbors of        C3C Check
```

Author Response: The high level of certain anisotropic displacement parameters is due to the gradual solvent loss through the data collection that enables localised atomic movement throughout the structure. This has in come cases resulted in localised deviations af neighbouring atom displacement parameter ratios

```
PLAT242_ALERT_2_C Low    'MainMol' Ueq as Compared to Neighbors of        C7C Check
```

Author Response: The high level of certain anisotropic displacement parameters is due to the gradual solvent loss through the data collection that enables localised atomic movement throughout the structure. This has in come cases resulted in localised deviations af neighbouring atom displacement parameter ratios

```
PLAT242_ALERT_2_C Low    'MainMol' Ueq as Compared to Neighbors of       C15D Check
```

Author Response: The high level of certain anisotropic displacement parameters is due to the gradual solvent loss through the data collection that enables localised atomic movement throughout the structure. This has in come cases resulted in localised deviations af neighbouring atom displacement parameter ratios

```
PLAT242_ALERT_2_C Low    'MainMol' Ueq as Compared to Neighbors of       C16C Check
```

Author Response: The high level of certain anisotropic displacement parameters is due to the gradual solvent loss through the data collection that enables localised atomic movement throughout the structure. This has in come cases resulted in localised deviations af neighbouring atom displacement parameter ratios

```
PLAT242_ALERT_2_C Low    'MainMol' Ueq as Compared to Neighbors of       C18D Check
```

Author Response: The high level of certain anisotropic displacement parameters is due to the gradual solvent loss through the data collection that enables localised atomic movement throughout the structure. This has in come cases resulted in localised deviations af neighbouring atom displacement parameter ratios

```
PLAT242_ALERT_2_C Low    'MainMol' Ueq as Compared to Neighbors of       C19D Check
```

Author Response: The high level of certain anisotropic displacement parameters is due to the gradual solvent loss through the data collection that enables localised atomic movement throughout the structure. This has in come cases resulted in localised deviations af neighbouring atom displacement parameter ratios

```
PLAT242_ALERT_2_C Low    'MainMol' Ueq as Compared to Neighbors of       C29D Check
```

Author Response: The high level of certain anisotropic displacement parameters is due to the gradual solvent loss through the data collection that enables localised atomic movement throughout the structure. This has in come cases resulted in localised deviations af neighbouring atom displacement parameter ratios

```
PLAT242_ALERT_2_C Low    'MainMol' Ueq as Compared to Neighbors of       C31C Check
```

Author Response: The high level of certain anisotropic displacement parameters is due to the gradual solvent loss through the data collection that enables localised atomic movement throughout the structure. This has in come cases resulted in localised deviations af neighbouring atom displacement parameter ratios

```
PLAT244_ALERT_4_C Low    'Solvent' Ueq as Compared to Neighbors of        S1U Check
PLAT244_ALERT_4_C Low    'Solvent' Ueq as Compared to Neighbors of        S1V Check
PLAT244_ALERT_4_C Low    'Solvent' Ueq as Compared to Neighbors of        S1W Check
PLAT244_ALERT_4_C Low    'Solvent' Ueq as Compared to Neighbors of        S1X Check
PLAT244_ALERT_4_C Low    'Solvent' Ueq as Compared to Neighbors of        S1Y Check
PLAT244_ALERT_4_C Low    'Solvent' Ueq as Compared to Neighbors of        S1Z Check
PLAT250_ALERT_2_C Large U3/U1 Ratio for Average U(i,j) Tensor ....        2.5 Note
PLAT260_ALERT_2_C Large Average Ueq of Residue Including       Eu1      0.192 Check
```

Author Response: The high level of certain anisotropic displacement parameters is due to the gradual solvent loss through the data collection that enables localised atomic movement throughout the structure.

```
PLAT260_ALERT_2_C Large Average Ueq of Residue Including       Eu3      0.184 Check
```

Author Response: The high level of certain anisotropic displacement parameters is due to the gradual solvent loss through the data collection that enables localised atomic movement throughout the structure.

```
PLAT260_ALERT_2_C Large Average Ueq of Residue Including       S1W      0.262 Check
```

Author Response: The high level of certain anisotropic displacement parameters is due to the gradual solvent loss through the data collection that enables localised atomic movement throughout the structure.

```
PLAT260_ALERT_2_C Large Average Ueq of Residue Including       S1Z      0.271 Check
```

Author Response: The high level of certain anisotropic displacement parameters is due to the gradual solvent loss through the data collection that enables localised atomic movement throughout the structure.

```
PLAT362_ALERT_2_C Short  C(sp3)-C(sp2) Bond  C2A      - C3A      .       1.34 Ang.
PLAT362_ALERT_2_C Short  C(sp3)-C(sp2) Bond  C29D     - C31D     .       1.38 Ang.
PLAT369_ALERT_2_C Long   C(sp2)-C(sp2) Bond  C14A     - C15A     .       1.54 Ang.
PLAT369_ALERT_2_C Long   C(sp2)-C(sp2) Bond  C22D     - C23D     .       1.53 Ang.
PLAT420_ALERT_2_C D-H Without Acceptor       N1A      --H1A      .     Please Check
PLAT420_ALERT_2_C D-H Without Acceptor       N3A      --H3A      .     Please Check
PLAT420_ALERT_2_C D-H Without Acceptor       N4D      --H4D      .     Please Check
PLAT420_ALERT_2_C D-H Without Acceptor       N6D      --H6D      .     Please Check
PLAT911_ALERT_3_C Missing FCF Refl Between Thmin & STh/L=    0.500        307 Report
PLAT915_ALERT_3_C No Flack x Check Done: Low Friedel Pair Coverage         61 %
PLAT923_ALERT_1_C S    Values in the CIF and FCF Differ by .......      0.022 Check
PLAT934_ALERT_3_C Number of (Iobs-Icalc)/Sigma(W) > 10 Outliers ..          1 Check
```

Alert level G

```
PLAT003_ALERT_2_G Number of Uiso or Uij Restrained non-H Atoms ...        236 Report
PLAT007_ALERT_5_G Number of Unrefined Donor-H Atoms .............          16 Report
PLAT033_ALERT_4_G Flack x Value Deviates > 3.0 * sigma from Zero .      0.030 Note
PLAT072_ALERT_2_G SHELXL First  Parameter in WGHT  Unusually Large       0.11 Report
PLAT083_ALERT_2_G SHELXL Second Parameter in WGHT  Unusually Large      24.76 Why ?
PLAT187_ALERT_4_G The CIF-Embedded .res File Contains RIGU Records          1 Report
PLAT244_ALERT_4_G Low    'Solvent' Ueq as Compared to Neighbors of        C1U Check
PLAT244_ALERT_4_G Low    'Solvent' Ueq as Compared to Neighbors of        C1V Check
PLAT244_ALERT_4_G Low    'Solvent' Ueq as Compared to Neighbors of        C1W Check
PLAT244_ALERT_4_G Low    'Solvent' Ueq as Compared to Neighbors of        C1X Check
PLAT244_ALERT_4_G Low    'Solvent' Ueq as Compared to Neighbors of        C1Y Check
PLAT244_ALERT_4_G Low    'Solvent' Ueq as Compared to Neighbors of        C1Z Check
PLAT300_ALERT_4_G Atom Site Occupancy of H1BA       Constrained at        0.5 Check
PLAT300_ALERT_4_G Atom Site Occupancy of H1BB       Constrained at        0.5 Check
PLAT300_ALERT_4_G Atom Site Occupancy of H1BC       Constrained at        0.5 Check
PLAT300_ALERT_4_G Atom Site Occupancy of H1BD       Constrained at        0.5 Check
PLAT300_ALERT_4_G Atom Site Occupancy of H1BE       Constrained at        0.5 Check
PLAT300_ALERT_4_G Atom Site Occupancy of H1BF       Constrained at        0.5 Check
PLAT300_ALERT_4_G Atom Site Occupancy of H1AA       Constrained at        0.5 Check
PLAT300_ALERT_4_G Atom Site Occupancy of H1AB       Constrained at        0.5 Check
PLAT300_ALERT_4_G Atom Site Occupancy of H1AC       Constrained at        0.5 Check
PLAT300_ALERT_4_G Atom Site Occupancy of H1AD       Constrained at        0.5 Check
PLAT300_ALERT_4_G Atom Site Occupancy of H1AE       Constrained at        0.5 Check
PLAT300_ALERT_4_G Atom Site Occupancy of H1AF       Constrained at        0.5 Check
PLAT300_ALERT_4_G Atom Site Occupancy of H30A       Constrained at        0.5 Check
```

```
PLAT300_ALERT_4_G Atom Site Occupancy of H30B       Constrained at        0.5 Check
PLAT300_ALERT_4_G Atom Site Occupancy of H30C       Constrained at        0.5 Check
PLAT300_ALERT_4_G Atom Site Occupancy of H30D       Constrained at        0.5 Check
PLAT300_ALERT_4_G Atom Site Occupancy of H30E       Constrained at        0.5 Check
PLAT300_ALERT_4_G Atom Site Occupancy of H30F       Constrained at        0.5 Check
PLAT300_ALERT_4_G Atom Site Occupancy of H30G       Constrained at        0.5 Check
PLAT300_ALERT_4_G Atom Site Occupancy of H30H       Constrained at        0.5 Check
PLAT300_ALERT_4_G Atom Site Occupancy of H30I       Constrained at        0.5 Check
PLAT300_ALERT_4_G Atom Site Occupancy of H30J       Constrained at        0.5 Check
PLAT300_ALERT_4_G Atom Site Occupancy of H30K       Constrained at        0.5 Check
PLAT300_ALERT_4_G Atom Site Occupancy of H30L       Constrained at        0.5 Check
PLAT300_ALERT_4_G Atom Site Occupancy of H1DA       Constrained at        0.5 Check
PLAT300_ALERT_4_G Atom Site Occupancy of H1DB       Constrained at        0.5 Check
PLAT300_ALERT_4_G Atom Site Occupancy of H1DC       Constrained at        0.5 Check
PLAT300_ALERT_4_G Atom Site Occupancy of H1DD       Constrained at        0.5 Check
PLAT300_ALERT_4_G Atom Site Occupancy of H1DE       Constrained at        0.5 Check
PLAT300_ALERT_4_G Atom Site Occupancy of H1DF       Constrained at        0.5 Check
PLAT300_ALERT_4_G Atom Site Occupancy of H1CA       Constrained at        0.5 Check
PLAT300_ALERT_4_G Atom Site Occupancy of H1CB       Constrained at        0.5 Check
PLAT300_ALERT_4_G Atom Site Occupancy of H1CC       Constrained at        0.5 Check
PLAT300_ALERT_4_G Atom Site Occupancy of H1CD       Constrained at        0.5 Check
PLAT300_ALERT_4_G Atom Site Occupancy of H1CE       Constrained at        0.5 Check
PLAT300_ALERT_4_G Atom Site Occupancy of H1CF       Constrained at        0.5 Check
PLAT300_ALERT_4_G Atom Site Occupancy of H30M       Constrained at        0.5 Check
PLAT300_ALERT_4_G Atom Site Occupancy of H30N       Constrained at        0.5 Check
PLAT300_ALERT_4_G Atom Site Occupancy of H30O       Constrained at        0.5 Check
PLAT300_ALERT_4_G Atom Site Occupancy of H30P       Constrained at        0.5 Check
PLAT300_ALERT_4_G Atom Site Occupancy of H30Q       Constrained at        0.5 Check
PLAT300_ALERT_4_G Atom Site Occupancy of H30R       Constrained at        0.5 Check
PLAT300_ALERT_4_G Atom Site Occupancy of H30S       Constrained at        0.5 Check
PLAT300_ALERT_4_G Atom Site Occupancy of H30T       Constrained at        0.5 Check
PLAT300_ALERT_4_G Atom Site Occupancy of H30U       Constrained at        0.5 Check
PLAT300_ALERT_4_G Atom Site Occupancy of H30V       Constrained at        0.5 Check
PLAT300_ALERT_4_G Atom Site Occupancy of H30W       Constrained at        0.5 Check
PLAT300_ALERT_4_G Atom Site Occupancy of H30X       Constrained at        0.5 Check
PLAT335_ALERT_2_G Check Large C6 Ring C-C Range C16A     -C21A           0.19 Ang.
PLAT335_ALERT_2_G Check Large C6 Ring C-C Range C16C     -C21C           0.17 Ang.
PLAT344_ALERT_2_G Unusual sp3       Angle Range in Solvent/Ion for        C1U Check
PLAT344_ALERT_2_G Unusual sp3       Angle Range in Solvent/Ion for        C1V Check
PLAT344_ALERT_2_G Unusual           Angle Range in Solvent/Ion for        C1X Check
PLAT367_ALERT_2_G Long?  C(sp?)-C(sp?) Bond  C1A      - C2A      .       1.64 Ang.
PLAT367_ALERT_2_G Long?  C(sp?)-C(sp?) Bond  C29A     - C30A     .       1.51 Ang.
PLAT367_ALERT_2_G Long?  C(sp?)-C(sp?) Bond  C29B     - C30B     .       1.58 Ang.
PLAT367_ALERT_2_G Long?  C(sp?)-C(sp?) Bond  C1C      - C2C      .       1.54 Ang.
PLAT367_ALERT_2_G Long?  C(sp?)-C(sp?) Bond  C1D      - C2D      .       1.53 Ang.
PLAT412_ALERT_2_G Short Intra XH3 .. XHn     H2B      ..H1BF     .       2.09 Ang.
                                                       x,y,z  =      1_555 Check
PLAT412_ALERT_2_G Short Intra XH3 .. XHn     H29A     ..H30L     .       2.14 Ang.
                                                       x,y,z  =      1_555 Check
PLAT412_ALERT_2_G Short Intra XH3 .. XHn     H30I     ..H32A     .       2.10 Ang.
                                                       x,y,z  =      1_555 Check
PLAT412_ALERT_2_G Short Intra XH3 .. XHn     H30K     ..H32A     .       2.14 Ang.
                                                       x,y,z  =      1_555 Check
PLAT412_ALERT_2_G Short Intra XH3 .. XHn     H29C     ..H30X     .       2.11 Ang.
                                                       x,y,z  =      1_555 Check
PLAT412_ALERT_2_G Short Intra XH3 .. XHn     H29D     ..H30Q     .       2.11 Ang.
                                                       x,y,z  =      1_555 Check
PLAT414_ALERT_2_G Short Intra D-H..H-X       H1A      ..H1AF             2.09 Ang.
                                                       x,y,z  =      1_555 Check
PLAT414_ALERT_2_G Short Intra D-H..H-X       H6B      ..H30F             1.96 Ang.
                                                       x,y,z  =      1_555 Check
PLAT414_ALERT_2_G Short Intra D-H..H-X       H1C      ..H1CF             1.90 Ang.
```

```
                                                      x,y,z  =      1_555 Check
PLAT606_ALERT_4_G VERY LARGE Solvent Accessible VOID(S) in Structure       ! Info
PLAT720_ALERT_4_G Number of Unusual/Non-Standard Labels ..........        32 Note
PLAT721_ALERT_1_G Bond    Calc     0.96000, Rep     0.97000 Dev...       0.01 Ang.
              C30B     -H30B           1.555   1.555 ........     #  252 Check
PLAT721_ALERT_1_G Bond    Calc     0.96000, Rep     0.97000 Dev...       0.01 Ang.
              C30B     -H30D           1.555   1.555 ........     #  254 Check
PLAT779_ALERT_4_G Suspect or Irrelevant (Bond) Angle(s) in CIF . #        18 Check
              O4C  -C28C -EU4     1.555   1.555   1.555             40.90 Deg.
PLAT779_ALERT_4_G Suspect or Irrelevant (Bond) Angle(s) in CIF . #       141 Check
              O1D  -C9D  -EU3     1.555   1.555   1.555             35.60 Deg.
PLAT779_ALERT_4_G Suspect or Irrelevant (Bond) Angle(s) in CIF . #       314 Check
              O2A  -C15A -EU2     1.555   1.555   2.665             36.70 Deg.
PLAT779_ALERT_4_G Suspect or Irrelevant (Bond) Angle(s) in CIF . #       704 Check
              O4A  -C28A -EU2     1.555   1.555   1.555             37.40 Deg.
PLAT860_ALERT_3_G Number of Least-Squares Restraints .............      1946 Note
PLAT869_ALERT_4_G ALERTS Related to the Use of SQUEEZE Suppressed          ! Info
PLAT883_ALERT_1_G No Info/Value for _atom_sites_solution_primary .     Please Do !
PLAT909_ALERT_3_G Percentage of I>2sig(I) Data at Theta(Max) Still       79% Note
PLAT910_ALERT_3_G Missing # of FCF Reflection(s) Below Theta(Min).         3 Note
PLAT913_ALERT_3_G Missing # of Very Strong Reflections in FCF ....         2 Note
PLAT941_ALERT_3_G Average HKL Measurement Multiplicity ...........       3.2 Low
PLAT978_ALERT_2_G Number C-C Bonds with Positive Residual Density.         0 Info
```

```
   5 ALERT level A = Most likely a serious problem - resolve or explain
  20 ALERT level B = A potentially serious problem, consider carefully
  87 ALERT level C = Check. Ensure it is not caused by an omission or oversight
  95 ALERT level G = General information/check it is not something unexpected

   5 ALERT type 1 CIF construction/syntax error, inconsistent or missing data
 118 ALERT type 2 Indicator that the structure model may be wrong or deficient
  14 ALERT type 3 Indicator that the structure quality may be low
  69 ALERT type 4 Improvement, methodology, query or suggestion
   1 ALERT type 5 Informative message, check
```

It is advisable to attempt to resolve as many as possible of the alerts in all categories. Often the minor alerts point to easily fixed oversights, errors and omissions in your CIF or refinement strategy, so attention to these fine details can be worthwhile. In order to resolve some of the more serious problems it may be necessary to carry out additional measurements or structure refinements. However, the purpose of your study may justify the reported deviations and the more serious of these should normally be commented upon in the discussion or experimental section of a paper or in the "special_details" fields of the CIF. checkCIF was carefully designed to identify outliers and unusual parameters, but every test has its limitations and alerts that are not important in a particular case may appear. Conversely, the absence of alerts does not guarantee there are no aspects of the results needing attention. It is up to the individual to critically assess their own results and, if necessary, seek expert advice.

Publication of your CIF in IUCr journals

A basic structural check has been run on your CIF. These basic checks will be run on all CIFs submitted for publication in IUCr journals (*Acta Crystallographica*, *Journal of Applied Crystallography*, *Journal of Synchrotron Radiation*); however, if you intend to submit to *Acta Crystallographica Section C* or *E* or *IUCrData*, you should make sure that full publication checks are run on the final version of your CIF prior to submission.

Publication of your CIF in other journals

Please refer to the *Notes for Authors* of the relevant journal for any special instructions relating to CIF submission.

PLATON version of 26/06/2020; check.def file version of 17/06/2020

Datablock missing_3_triflates_sq - ellipsoid plot

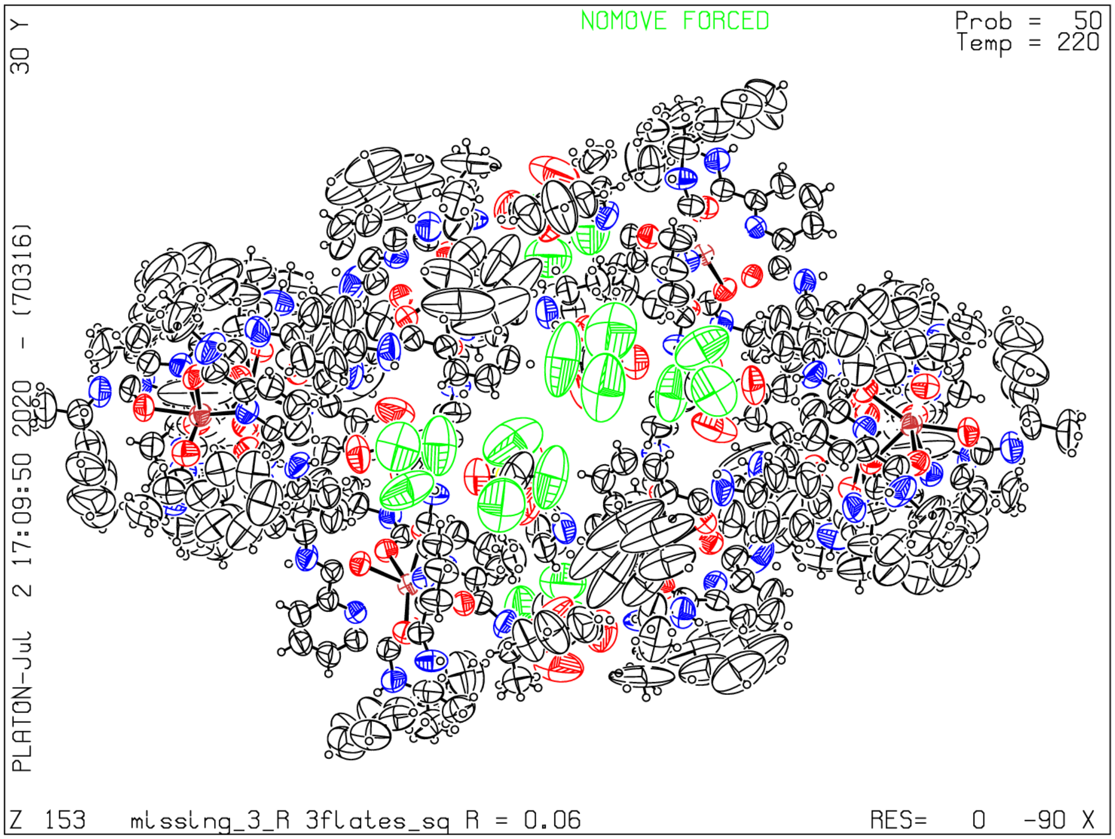

Supplement: Supplementary file 4 — Supplementary Data 2 [file 42004_2021_553_MOESM4_ESM.zip › Supplementary Data 2/most_stable_refinement_missing_3_triflates_per_unit/missing_3_triflates_sq_checkcif.pdf]
