# Supplementary material for: Helicate-to-tetrahedron transformation of chiral lanthanide supramolecular complexes induced by ionic radii effect and linker length
Source: Commun Chem. 2021 Aug 5;4:116. doi: 10.1038/s42004-021-00553-8 (PMC9814731; doi:10.1038/s42004-021-00553-8)

checkCIF/PLATON report

Structure factors have been supplied for datablock(s) BCYCT30

THIS REPORT IS FOR GUIDANCE ONLY. IF USED AS PART OF A REVIEW PROCEDURE FOR PUBLICATION, IT SHOULD NOT REPLACE THE EXPERTISE OF AN EXPERIENCED CRYSTALLOGRAPHIC REFEREE.

No syntax errors found.   CIF dictionary   Interpreting this report

Datablock: BCYCT30

```
Bond precision:  C-C = 0.0163 A          Wavelength=0.71073

Cell:              a=26.075(3)      b=28.363(4)      c=23.396(3)
                   alpha=90         beta=90          gamma=90
Temperature:       296 K

                Calculated                 Reported
Volume          17303(4)                   17304(4)
Space group     P 21 21 2                  P 21 21 2
Hall group      P 2 2ab                    P 2 2ab
Moiety formula  C126 H96 N18 O12 Tb2       C126 H96 N18 O12 Tb2
Sum formula     C126 H96 N18 O12 Tb2       C126 H96 N18 O12 Tb2
Mr              2372.07                    2372.04
Dx,g cm-3       0.911                      0.911
Z               4                          4
Mu (mm-1)       0.857                      0.857
F000            4816.0                     4816.0
F000'           4816.27
h,k,lmax        31,34,28                   31,34,28
Nref            31697[ 16983]              31661
Tmin,Tmax       0.727,0.842                0.684,0.847
Tmin'           0.656

Correction method= # Reported T Limits: Tmin=0.684 Tmax=0.847
AbsCorr = MULTI-SCAN

Data completeness= 1.86/1.00      Theta(max)= 25.350

R(reflections)= 0.0524( 21847)    wR2(reflections)= 0.1240( 31661)

S = 0.941                  Npar= 1363
```

The following ALERTS were generated. Each ALERT has the format
test-name_ALERT_alert-type_alert-level.
Click on the hyperlinks for more details of the test.

Alert level A

PLAT234_ALERT_4_A Large Hirshfeld Difference C114 --C115 . 0.39 Ang.

Author Response: This is a result of the poor data, where a significant drop-off in intensity can be observed beyond a resolution of 1.20 A, which leads to poor accuracy in refinements and unusually high displacement parameters.

PLAT234_ALERT_4_A Large Hirshfeld Difference C122 --C123 . 0.35 Ang.

Author Response: This is a result of the poor data, where a significant drop-off in intensity can be observed beyond a resolution of 1.20 A, which leads to poor accuracy in refinements and unusually high displacement parameters.

PLAT241_ALERT_2_A High 'MainMol' Ueq as Compared to Neighbors of C108 Check

Author Response: This is a result of the poor data, where a significant drop-off in intensity can be observed beyond a resolution of 1.20 A, which leads to poor accuracy in refinements and unusually high displacement parameters.

PLAT602_ALERT_2_A VERY LARGE Solvent Accessible VOID(S) in Structure ! Info

Author Response: Due to significant disorder, not all anion and/or solvent moiety could be located. As a result PLATON/SQEEZE was applied to remove these moieties from refinements.

Alert level B

PLAT049_ALERT_1_B Calculated Density Less Than 1.0 gcm-3 .... 0.9106 Check

Author Response: The low density is due to application of PLATON/SQUEEZE.

PLAT220_ALERT_2_B NonSolvent Resd 1 C Ueq(max) / Ueq(min) Range 10.0 Ratio

Author Response: This is a result of the poor data, where a significant drop-off in intensity can be observed beyond a resolution of 1.20 A, which leads to poor accuracy in refinements and unusually high displacement parameters.

PLAT234_ALERT_4_B Large Hirshfeld Difference C101 --C102 . 0.30 Ang.

Author Response: This is a result of the poor data, where a significant drop-off in intensity can be observed beyond a resolution of 1.20 A, which leads to poor accuracy in refinements and unusually high displacement parameters.

PLAT234_ALERT_4_B Large Hirshfeld Difference C113 --C114 . 0.28 Ang.

Author Response: This is a result of the poor data, where a significant drop-off in intensity can be observed beyond a resolution of 1.20 A, which leads to poor accuracy in refinements and unusually high displacement parameters.

PLAT234_ALERT_4_B Large Hirshfeld Difference C117 --C118 . 0.28 Ang.

Author Response: This is a result of the poor data, where a significant drop-off in intensity can be observed beyond a resolution of 1.20 A, which leads to poor accuracy in refinements and unusually high displacement parameters.

PLAT241_ALERT_2_B High 'MainMol' Ueq as Compared to Neighbors of C85 Check

Author Response: This is a result of the poor data, where a significant drop-off in intensity can be observed beyond a resolution of 1.20 A, which leads to poor accuracy in refinements and unusually high displacement parameters.

PLAT241_ALERT_2_B High 'MainMol' Ueq as Compared to Neighbors of C90 Check

Author Response: This is a result of the poor data, where a significant drop-off in intensity can be observed beyond a resolution of 1.20 A, which leads to poor accuracy in refinements and unusually high displacement parameters.

PLAT241_ALERT_2_B High 'MainMol' Ueq as Compared to Neighbors of C110 Check

Author Response: This is a result of the poor data, where a significant drop-off in intensity can be observed beyond a resolution of 1.20 A, which leads to poor accuracy in refinements and unusually high displacement parameters.

PLAT241_ALERT_2_B High 'MainMol' Ueq as Compared to Neighbors of C116 Check

Author Response: This is a result of the poor data, where a significant drop-off in intensity can be observed beyond a resolution of 1.20 A, which leads to poor accuracy in refinements and unusually high displacement parameters.

```
PLAT241_ALERT_2_B High   'MainMol' Ueq as Compared to Neighbors of       C123 Check
```

Author Response: This is a result of the poor data, where a significant drop-off in intensity can be observed beyond a resolution of 1.20 A, which leads to poor accuracy in refinements and unusually high displacement parameters.

```
PLAT241_ALERT_2_B High   'MainMol' Ueq as Compared to Neighbors of       C125 Check
```

Author Response: This is a result of the poor data, where a significant drop-off in intensity can be observed beyond a resolution of 1.20 A, which leads to poor accuracy in refinements and unusually high displacement parameters.

```
PLAT242_ALERT_2_B Low    'MainMol' Ueq as Compared to Neighbors of        C86 Check
```

Author Response: This is a result of the poor data, where a significant drop-off in intensity can be observed beyond a resolution of 1.20 A, which leads to poor accuracy in refinements and unusually high displacement parameters.

```
PLAT242_ALERT_2_B Low    'MainMol' Ueq as Compared to Neighbors of        C89 Check
```

Author Response: This is a result of the poor data, where a significant drop-off in intensity can be observed beyond a resolution of 1.20 A, which leads to poor accuracy in refinements and unusually high displacement parameters.

```
PLAT242_ALERT_2_B Low    'MainMol' Ueq as Compared to Neighbors of        C91 Check
```

Author Response: This is a result of the poor data, where a significant drop-off in intensity can be observed beyond a resolution of 1.20 A, which leads to poor accuracy in refinements and unusually high displacement parameters.

```
PLAT242_ALERT_2_B Low    'MainMol' Ueq as Compared to Neighbors of       C109 Check
```

Author Response: This is a result of the poor data, where a significant drop-off in intensity can be observed beyond a resolution of 1.20 A, which leads to poor accuracy in refinements and unusually high displacement parameters.

```
PLAT242_ALERT_2_B Low    'MainMol' Ueq as Compared to Neighbors of       C122 Check
```

Author Response: This is a result of the poor data, where a significant drop-off in intensity can be observed beyond a resolution of 1.20 A, which leads to poor accuracy in refinements and unusually high displacement parameters.

```
PLAT242_ALERT_2_B Low    'MainMol' Ueq as Compared to Neighbors of       C124 Check
```

Author Response: This is a result of the poor data, where a significant drop-off in intensity can be observed beyond a resolution of 1.20 A, which leads to poor accuracy in refinements and unusually high displacement parameters.

```
PLAT369_ALERT_2_B Long   C(sp2)-C(sp2) Bond  C58      - C59      .       1.61 Ang.
```

Author Response: This is a result of the poor data, where a significant drop-off in intensity can be observed beyond a resolution of 1.20 A, which leads to poor accuracy in refinements and unusually high displacement parameters.

```
PLAT910_ALERT_3_B Missing # of FCF Reflection(s) Below Theta(Min).         28 Note
```

Alert level C

```
PLAT222_ALERT_3_C NonSolvent Resd 1  H   Uiso(max)/Uiso(min) Range        9.4 Ratio
```

Author Response: This is a result of the poor data, where a significant drop-off in intensity can be observed beyond a resolution of 1.20 A, which leads to poor accuracy in refinements and unusually high displacement parameters.

```
PLAT230_ALERT_2_C Hirshfeld Test Diff for    N16      --C72      .        5.4 s.u.
```

Author Response: This is a result of the poor data, where a significant drop-off in intensity can be observed beyond a resolution of 1.20 A, which leads to poor accuracy in refinements and unusually high displacement parameters.

```
PLAT230_ALERT_2_C Hirshfeld Test Diff for    C37      --C39      .        6.3 s.u.
```

Author Response: This is a result of the poor data, where a significant drop-off in intensity can be observed beyond a resolution of 1.20 A, which leads to poor accuracy in refinements and unusually high displacement parameters.

```
PLAT230_ALERT_2_C Hirshfeld Test Diff for    C44      --C45      .        5.6 s.u.
```

Author Response: This is a result of the poor data, where a significant drop-off in intensity can be observed beyond a resolution of 1.20 A, which leads to poor accuracy in refinements and unusually high displacement parameters.

```
PLAT230_ALERT_2_C Hirshfeld Test Diff for    C73      --C74      .        5.4 s.u.
```

Author Response: This is a result of the poor data, where a significant drop-off in intensity can be observed beyond a resolution of 1.20 A, which leads to poor accuracy in refinements and unusually high displacement parameters.

```
PLAT234_ALERT_4_C Large Hirshfeld Difference O2       --C7       .       0.16 Ang.
```

Author Response: This is a result of the poor data, where a significant drop-off in intensity can be observed beyond a resolution of 1.20 A, which leads to poor accuracy in refinements and unusually high displacement parameters.

```
PLAT234_ALERT_4_C Large Hirshfeld Difference O11      --C72      .       0.16 Ang.
```

Author Response: This is a result of the poor data, where a significant drop-off in intensity can be observed beyond a resolution of 1.20 A, which leads to poor accuracy in refinements and unusually high displacement parameters.

```
PLAT234_ALERT_4_C Large Hirshfeld Difference N6       --C87      .       0.19 Ang.
```

Author Response: This is a result of the poor data, where a significant drop-off in intensity can be observed beyond a resolution of 1.20 A, which leads to poor accuracy in refinements and unusually high displacement parameters.

```
PLAT234_ALERT_4_C Large Hirshfeld Difference N12      --C103     .       0.20 Ang.
```

Author Response: This is a result of the poor data, where a significant drop-off in intensity can be observed beyond a resolution of 1.20 A, which leads to poor accuracy in refinements and unusually high displacement parameters.

```
PLAT234_ALERT_4_C Large Hirshfeld Difference N13      --C111     .       0.21 Ang.
```

Author Response: This is a result of the poor data, where a significant drop-off in intensity can be observed beyond a resolution of 1.20 A, which leads to poor accuracy in refinements and unusually high displacement parameters.

```
PLAT234_ALERT_4_C Large Hirshfeld Difference C9       --C11      .       0.16 Ang.
```

Author Response: This is a result of the poor data, where a significant drop-off in intensity can be observed beyond a resolution of 1.20 A, which leads to poor accuracy in refinements and unusually high displacement parameters.

```
PLAT234_ALERT_4_C Large Hirshfeld Difference C15      --C17      .       0.19 Ang.
```

Author Response: This is a result of the poor data, where a significant drop-off in intensity can be observed beyond a resolution of 1.20 A, which leads to poor accuracy in refinements and unusually high displacement parameters.

```
PLAT234_ALERT_4_C Large Hirshfeld Difference C17      --C19      .       0.19 Ang.
```

Author Response: This is a result of the poor data, where a significant drop-off in intensity can be observed beyond a resolution of 1.20 A, which leads to poor accuracy in refinements and unusually high displacement parameters.

```
PLAT234_ALERT_4_C Large Hirshfeld Difference C23      --C24      .       0.16 Ang.
```

Author Response: This is a result of the poor data, where a significant drop-off in intensity can be observed beyond a resolution of 1.20 A, which leads to poor accuracy in refinements and unusually high displacement parameters.

```
PLAT234_ALERT_4_C Large Hirshfeld Difference C40      --C41      .       0.18 Ang.
```

Author Response: This is a result of the poor data, where a significant drop-off in intensity can be observed beyond a resolution of 1.20 A, which leads to poor accuracy in refinements and unusually high displacement parameters.

```
PLAT234_ALERT_4_C Large Hirshfeld Difference C56      --C57      .       0.16 Ang.
```

Author Response: This is a result of the poor data, where a significant drop-off in intensity can be observed beyond a resolution of 1.20 A, which leads to poor accuracy in refinements and unusually high displacement parameters.

PLAT234_ALERT_4_C Large Hirshfeld Difference C66 --C67 . 0.17 Ang.

Author Response: This is a result of the poor data, where a significant drop-off in intensity can be observed beyond a resolution of 1.20 A, which leads to poor accuracy in refinements and unusually high displacement parameters.

PLAT234_ALERT_4_C Large Hirshfeld Difference C69 --C71 . 0.16 Ang.

Author Response: This is a result of the poor data, where a significant drop-off in intensity can be observed beyond a resolution of 1.20 A, which leads to poor accuracy in refinements and unusually high displacement parameters.

PLAT234_ALERT_4_C Large Hirshfeld Difference C70 --C71 . 0.17 Ang.

Author Response: This is a result of the poor data, where a significant drop-off in intensity can be observed beyond a resolution of 1.20 A, which leads to poor accuracy in refinements and unusually high displacement parameters.

PLAT234_ALERT_4_C Large Hirshfeld Difference C74 --C75 . 0.16 Ang.

Author Response: This is a result of the poor data, where a significant drop-off in intensity can be observed beyond a resolution of 1.20 A, which leads to poor accuracy in refinements and unusually high displacement parameters.

PLAT234_ALERT_4_C Large Hirshfeld Difference C79 --C80 . 0.17 Ang.

Author Response: This is a result of the poor data, where a significant drop-off in intensity can be observed beyond a resolution of 1.20 A, which leads to poor accuracy in refinements and unusually high displacement parameters.

PLAT234_ALERT_4_C Large Hirshfeld Difference C83 --C84 . 0.21 Ang.

Author Response: This is a result of the poor data, where a significant drop-off in intensity can be observed beyond a resolution of 1.20 A, which leads to poor accuracy in refinements and unusually high displacement parameters.

PLAT234_ALERT_4_C Large Hirshfeld Difference C87 --C89 . 0.23 Ang.

Author Response: This is a result of the poor data, where a significant drop-off in intensity can be observed beyond a resolution of 1.20 A, which leads to poor accuracy in refinements and unusually high displacement parameters.

```
PLAT234_ALERT_4_C Large Hirshfeld Difference C97      --C102     .       0.20 Ang.
```

Author Response: This is a result of the poor data, where a significant drop-off in intensity can be observed beyond a resolution of 1.20 A, which leads to poor accuracy in refinements and unusually high displacement parameters.

```
PLAT234_ALERT_4_C Large Hirshfeld Difference C98      --C99      .       0.25 Ang.
```

Author Response: This is a result of the poor data, where a significant drop-off in intensity can be observed beyond a resolution of 1.20 A, which leads to poor accuracy in refinements and unusually high displacement parameters.

```
PLAT234_ALERT_4_C Large Hirshfeld Difference C99      --C100     .       0.24 Ang.
```

Author Response: This is a result of the poor data, where a significant drop-off in intensity can be observed beyond a resolution of 1.20 A, which leads to poor accuracy in refinements and unusually high displacement parameters.

```
PLAT234_ALERT_4_C Large Hirshfeld Difference C103     --C104     .       0.22 Ang.
```

Author Response: This is a result of the poor data, where a significant drop-off in intensity can be observed beyond a resolution of 1.20 A, which leads to poor accuracy in refinements and unusually high displacement parameters.

```
PLAT234_ALERT_4_C Large Hirshfeld Difference C105     --C106     .       0.22 Ang.
```

Author Response: This is a result of the poor data, where a significant drop-off in intensity can be observed beyond a resolution of 1.20 A, which leads to poor accuracy in refinements and unusually high displacement parameters.

```
PLAT234_ALERT_4_C Large Hirshfeld Difference C121     --C122     .       0.25 Ang.
```

Author Response: This is a result of the poor data, where a significant drop-off in intensity can be observed beyond a resolution of 1.20 A, which leads to poor accuracy in refinements and unusually high displacement parameters.

```
PLAT241_ALERT_2_C High   'MainMol' Ueq as Compared to Neighbors of         C4 Check
```

Author Response: This is a result of the poor data, where a significant drop-off in intensity can be observed beyond a resolution of 1.20 A, which leads to poor accuracy in refinements and unusually high displacement parameters.

```
PLAT241_ALERT_2_C High   'MainMol' Ueq as Compared to Neighbors of        C10 Check
```

Author Response: This is a result of the poor data, where a significant drop-off in intensity can be observed beyond a resolution of 1.20 A, which leads to poor accuracy in refinements and unusually high displacement parameters.

```
PLAT241_ALERT_2_C High   'MainMol' Ueq as Compared to Neighbors of        C22 Check
```

Author Response: This is a result of the poor data, where a significant drop-off in intensity can be observed beyond a resolution of 1.20 A, which leads to poor accuracy in refinements and unusually high displacement parameters.

```
PLAT241_ALERT_2_C High   'MainMol' Ueq as Compared to Neighbors of        C30 Check
```

Author Response: This is a result of the poor data, where a significant drop-off in intensity can be observed beyond a resolution of 1.20 A, which leads to poor accuracy in refinements and unusually high displacement parameters.

```
PLAT241_ALERT_2_C High   'MainMol' Ueq as Compared to Neighbors of        C37 Check
```

Author Response: This is a result of the poor data, where a significant drop-off in intensity can be observed beyond a resolution of 1.20 A, which leads to poor accuracy in refinements and unusually high displacement parameters.

```
PLAT241_ALERT_2_C High   'MainMol' Ueq as Compared to Neighbors of        C43 Check
```

Author Response: This is a result of the poor data, where a significant drop-off in intensity can be observed beyond a resolution of 1.20 A, which leads to poor accuracy in refinements and unusually high displacement parameters.

```
PLAT241_ALERT_2_C High   'MainMol' Ueq as Compared to Neighbors of        C44 Check
```

Author Response: This is a result of the poor data, where a significant drop-off in intensity can be observed beyond a resolution of 1.20 A, which leads to poor accuracy in refinements and unusually high displacement parameters.

```
PLAT241_ALERT_2_C High   'MainMol' Ueq as Compared to Neighbors of       C75 Check
```

Author Response: This is a result of the poor data, where a significant drop-off in intensity can be observed beyond a resolution of 1.20 A, which leads to poor accuracy in refinements and unusually high displacement parameters.

```
PLAT241_ALERT_2_C High   'MainMol' Ueq as Compared to Neighbors of       C92 Check
```

Author Response: This is a result of the poor data, where a significant drop-off in intensity can be observed beyond a resolution of 1.20 A, which leads to poor accuracy in refinements and unusually high displacement parameters.

```
PLAT241_ALERT_2_C High   'MainMol' Ueq as Compared to Neighbors of       C93 Check
```

Author Response: This is a result of the poor data, where a significant drop-off in intensity can be observed beyond a resolution of 1.20 A, which leads to poor accuracy in refinements and unusually high displacement parameters.

```
PLAT241_ALERT_2_C High   'MainMol' Ueq as Compared to Neighbors of       C94 Check
```

Author Response: This is a result of the poor data, where a significant drop-off in intensity can be observed beyond a resolution of 1.20 A, which leads to poor accuracy in refinements and unusually high displacement parameters.

```
PLAT241_ALERT_2_C High   'MainMol' Ueq as Compared to Neighbors of       C98 Check
```

Author Response: This is a result of the poor data, where a significant drop-off in intensity can be observed beyond a resolution of 1.20 A, which leads to poor accuracy in refinements and unusually high displacement parameters.

```
PLAT241_ALERT_2_C High   'MainMol' Ueq as Compared to Neighbors of       C99 Check
```

Author Response: This is a result of the poor data, where a significant drop-off in intensity can be observed beyond a resolution of 1.20 A, which leads to poor accuracy in refinements and unusually high displacement parameters.

```
PLAT241_ALERT_2_C High   'MainMol' Ueq as Compared to Neighbors of       C101 Check
```

Author Response: This is a result of the poor data, where a significant drop-off in intensity can be observed beyond a resolution of 1.20 A, which leads to poor accuracy in refinements and unusually high displacement parameters.

```
PLAT241_ALERT_2_C High   'MainMol' Ueq as Compared to Neighbors of       C115 Check
```

Author Response: This is a result of the poor data, where a significant drop-off in intensity can be observed beyond a resolution of 1.20 A, which leads to poor accuracy in refinements and unusually high displacement parameters.

```
PLAT242_ALERT_2_C Low    'MainMol' Ueq as Compared to Neighbors of        C39 Check
```

Author Response: This is a result of the poor data, where a significant drop-off in intensity can be observed beyond a resolution of 1.20 A, which leads to poor accuracy in refinements and unusually high displacement parameters.

```
PLAT242_ALERT_2_C Low    'MainMol' Ueq as Compared to Neighbors of        C45 Check
```

Author Response: This is a result of the poor data, where a significant drop-off in intensity can be observed beyond a resolution of 1.20 A, which leads to poor accuracy in refinements and unusually high displacement parameters.

```
PLAT242_ALERT_2_C Low    'MainMol' Ueq as Compared to Neighbors of        C53 Check
```

Author Response: This is a result of the poor data, where a significant drop-off in intensity can be observed beyond a resolution of 1.20 A, which leads to poor accuracy in refinements and unusually high displacement parameters.

```
PLAT242_ALERT_2_C Low    'MainMol' Ueq as Compared to Neighbors of        C71 Check
```

Author Response: This is a result of the poor data, where a significant drop-off in intensity can be observed beyond a resolution of 1.20 A, which leads to poor accuracy in refinements and unusually high displacement parameters.

```
PLAT242_ALERT_2_C Low    'MainMol' Ueq as Compared to Neighbors of        C79 Check
```

Author Response: This is a result of the poor data, where a significant drop-off in intensity can be observed beyond a resolution of 1.20 A, which leads to poor accuracy in refinements and unusually high displacement parameters.

```
PLAT242_ALERT_2_C Low    'MainMol' Ueq as Compared to Neighbors of        C82 Check
```

Author Response: This is a result of the poor data, where a significant drop-off in intensity can be observed beyond a resolution of 1.20 A, which leads to poor accuracy in refinements and unusually high displacement parameters.

```
PLAT242_ALERT_2_C Low    'MainMol' Ueq as Compared to Neighbors of        C84 Check
```

Author Response: This is a result of the poor data, where a significant drop-off in intensity can be observed beyond a resolution of 1.20 A, which leads to poor accuracy in refinements and unusually high displacement parameters.

```
PLAT242_ALERT_2_C Low    'MainMol' Ueq as Compared to Neighbors of        C87 Check
```

Author Response: This is a result of the poor data, where a significant drop-off in intensity can be observed beyond a resolution of 1.20 A, which leads to poor accuracy in refinements and unusually high displacement parameters.

```
PLAT242_ALERT_2_C Low    'MainMol' Ueq as Compared to Neighbors of        C97 Check
```

Author Response: This is a result of the poor data, where a significant drop-off in intensity can be observed beyond a resolution of 1.20 A, which leads to poor accuracy in refinements and unusually high displacement parameters.

```
PLAT242_ALERT_2_C Low    'MainMol' Ueq as Compared to Neighbors of       C100 Check
```

Author Response: This is a result of the poor data, where a significant drop-off in intensity can be observed beyond a resolution of 1.20 A, which leads to poor accuracy in refinements and unusually high displacement parameters.

```
PLAT242_ALERT_2_C Low    'MainMol' Ueq as Compared to Neighbors of       C103 Check
```

Author Response: This is a result of the poor data, where a significant drop-off in intensity can be observed beyond a resolution of 1.20 A, which leads to poor accuracy in refinements and unusually high displacement parameters.

```
PLAT242_ALERT_2_C Low    'MainMol' Ueq as Compared to Neighbors of       C105 Check
```

Author Response: This is a result of the poor data, where a significant drop-off in intensity can be observed beyond a resolution of 1.20 A, which leads to poor accuracy in refinements and unusually high displacement parameters.

```
PLAT242_ALERT_2_C Low    'MainMol' Ueq as Compared to Neighbors of       C106 Check
```

Author Response: This is a result of the poor data, where a significant drop-off in intensity can be observed beyond a resolution of 1.20 A, which leads to poor accuracy in refinements and unusually high displacement parameters.

```
PLAT242_ALERT_2_C Low    'MainMol' Ueq as Compared to Neighbors of       C107 Check
```

Author Response: This is a result of the poor data, where a significant drop-off in intensity can be observed beyond a resolution of 1.20 A, which leads to poor accuracy in refinements and unusually high displacement parameters.

```
PLAT242_ALERT_2_C Low    'MainMol' Ueq as Compared to Neighbors of       C111 Check
```

Author Response: This is a result of the poor data, where a significant drop-off in intensity can be observed beyond a resolution of 1.20 A, which leads to poor accuracy in refinements and unusually high displacement parameters.

```
PLAT242_ALERT_2_C Low    'MainMol' Ueq as Compared to Neighbors of       C113 Check
```

Author Response: This is a result of the poor data, where a significant drop-off in intensity can be observed beyond a resolution of 1.20 A, which leads to poor accuracy in refinements and unusually high displacement parameters.

```
PLAT242_ALERT_2_C Low    'MainMol' Ueq as Compared to Neighbors of       C117 Check
```

Author Response: This is a result of the poor data, where a significant drop-off in intensity can be observed beyond a resolution of 1.20 A, which leads to poor accuracy in refinements and unusually high displacement parameters.

```
PLAT242_ALERT_2_C Low    'MainMol' Ueq as Compared to Neighbors of       C121 Check
```

Author Response: This is a result of the poor data, where a significant drop-off in intensity can be observed beyond a resolution of 1.20 A, which leads to poor accuracy in refinements and unusually high displacement parameters.

```
PLAT242_ALERT_2_C Low    'MainMol' Ueq as Compared to Neighbors of       C126 Check
```

Author Response: This is a result of the poor data, where a significant drop-off in intensity can be observed beyond a resolution of 1.20 A, which leads to poor accuracy in refinements and unusually high displacement parameters.

```
PLAT334_ALERT_2_C Small Aver. Benzene C-C Dist C8       -C10             1.36 Ang.
PLAT334_ALERT_2_C Small Aver. Benzene C-C Dist C14      -C16             1.37 Ang.
PLAT334_ALERT_2_C Small Aver. Benzene C-C Dist C66      -C68             1.35 Ang.
PLAT342_ALERT_3_C Low Bond Precision on  C-C Bonds ...............    0.01626 Ang.
PLAT360_ALERT_2_C Short  C(sp3)-C(sp3) Bond  C95      - C96      .       1.41 Ang.
PLAT362_ALERT_2_C Short  C(sp3)-C(sp2) Bond  C111     - C113     .       1.36 Ang.
PLAT369_ALERT_2_C Long   C(sp2)-C(sp2) Bond  C25      - C26      .       1.55 Ang.
```

Author Response: This is a result of the poor data, where a significant drop-off in intensity can be observed beyond a resolution of 1.20 A, which leads to poor accuracy in refinements and unusually high displacement parameters.

```
PLAT369_ALERT_2_C Long   C(sp2)-C(sp2) Bond  C72      - C73      .       1.55 Ang.
```

Author Response: This is a result of the poor data, where a significant drop-off in intensity can be observed beyond a resolution of 1.20 A, which leads to poor accuracy in refinements and unusually high displacement parameters.

```
PLAT369_ALERT_2_C Long   C(sp2)-C(sp2) Bond  C77      - C78      .       1.56 Ang.
```

Author Response: This is a result of the poor data, where a significant drop-off in intensity can be observed beyond a resolution of 1.20 A, which leads to poor accuracy in refinements and unusually high displacement parameters.

```
PLAT906_ALERT_3_C Large K Value in the Analysis of Variance ......      3.197 Check
PLAT913_ALERT_3_C Missing # of Very Strong Reflections in FCF ....          6 Note
PLAT934_ALERT_3_C Number of (Iobs-Icalc)/Sigma(W) > 10 Outliers ..          1 Check
```

Alert level G

```
PLAT003_ALERT_2_G Number of Uiso or Uij Restrained non-H Atoms ...         17 Report
PLAT177_ALERT_4_G The CIF-Embedded .res File Contains DELU Records         11 Report
PLAT178_ALERT_4_G The CIF-Embedded .res File Contains SIMU Records          1 Report
PLAT187_ALERT_4_G The CIF-Embedded .res File Contains RIGU Records          1 Report
PLAT791_ALERT_4_G Model has Chirality at C79         (Sohnke SpGr)          S Verify
PLAT791_ALERT_4_G Model has Chirality at C87         (Sohnke SpGr)          S Verify
PLAT791_ALERT_4_G Model has Chirality at C95         (Sohnke SpGr)          S Verify
```

```
PLAT791_ALERT_4_G Model has Chirality at C103        (Sohnke SpGr)          S Verify
PLAT791_ALERT_4_G Model has Chirality at C111        (Sohnke SpGr)          S Verify
PLAT791_ALERT_4_G Model has Chirality at C119        (Sohnke SpGr)          S Verify
PLAT794_ALERT_5_G Tentative Bond Valency for Tb1       (III)     .       3.42 Info
PLAT794_ALERT_5_G Tentative Bond Valency for Tb2       (III)     .       3.42 Info
PLAT860_ALERT_3_G Number of Least-Squares Restraints .............         17 Note
PLAT961_ALERT_5_G Dataset Contains no Negative Intensities .......     Please Check
PLAT978_ALERT_2_G Number C-C Bonds with Positive Residual Density.          2 Info
PLAT992_ALERT_5_G Repd & Actual _reflns_number_gt Values Differ by          1 Check
```

```
   4 ALERT level A = Most likely a serious problem - resolve or explain
  19 ALERT level B = A potentially serious problem, consider carefully
  75 ALERT level C = Check. Ensure it is not caused by an omission or oversight
  16 ALERT level G = General information/check it is not something unexpected

   1 ALERT type 1 CIF construction/syntax error, inconsistent or missing data
  64 ALERT type 2 Indicator that the structure model may be wrong or deficient
   7 ALERT type 3 Indicator that the structure quality may be low
  38 ALERT type 4 Improvement, methodology, query or suggestion
   4 ALERT type 5 Informative message, check
```

It is advisable to attempt to resolve as many as possible of the alerts in all categories. Often the minor alerts point to easily fixed oversights, errors and omissions in your CIF or refinement strategy, so attention to these fine details can be worthwhile. In order to resolve some of the more serious problems it may be necessary to carry out additional measurements or structure refinements. However, the purpose of your study may justify the reported deviations and the more serious of these should normally be commented upon in the discussion or experimental section of a paper or in the "special_details" fields of the CIF. checkCIF was carefully designed to identify outliers and unusual parameters, but every test has its limitations and alerts that are not important in a particular case may appear. Conversely, the absence of alerts does not guarantee there are no aspects of the results needing attention. It is up to the individual to critically assess their own results and, if necessary, seek expert advice.

Publication of your CIF in IUCr journals

A basic structural check has been run on your CIF. These basic checks will be run on all CIFs submitted for publication in IUCr journals (*Acta Crystallographica*, *Journal of Applied Crystallography*, *Journal of Synchrotron Radiation*); however, if you intend to submit to *Acta Crystallographica Section C* or *E* or *IUCrData*, you should make sure that full publication checks are run on the final version of your CIF prior to submission.

Publication of your CIF in other journals

Please refer to the *Notes for Authors* of the relevant journal for any special instructions relating to CIF submission.

PLATON version of 22/04/2020; check.def file version of 09/03/2020

Datablock BCYCT30 - ellipsoid plot

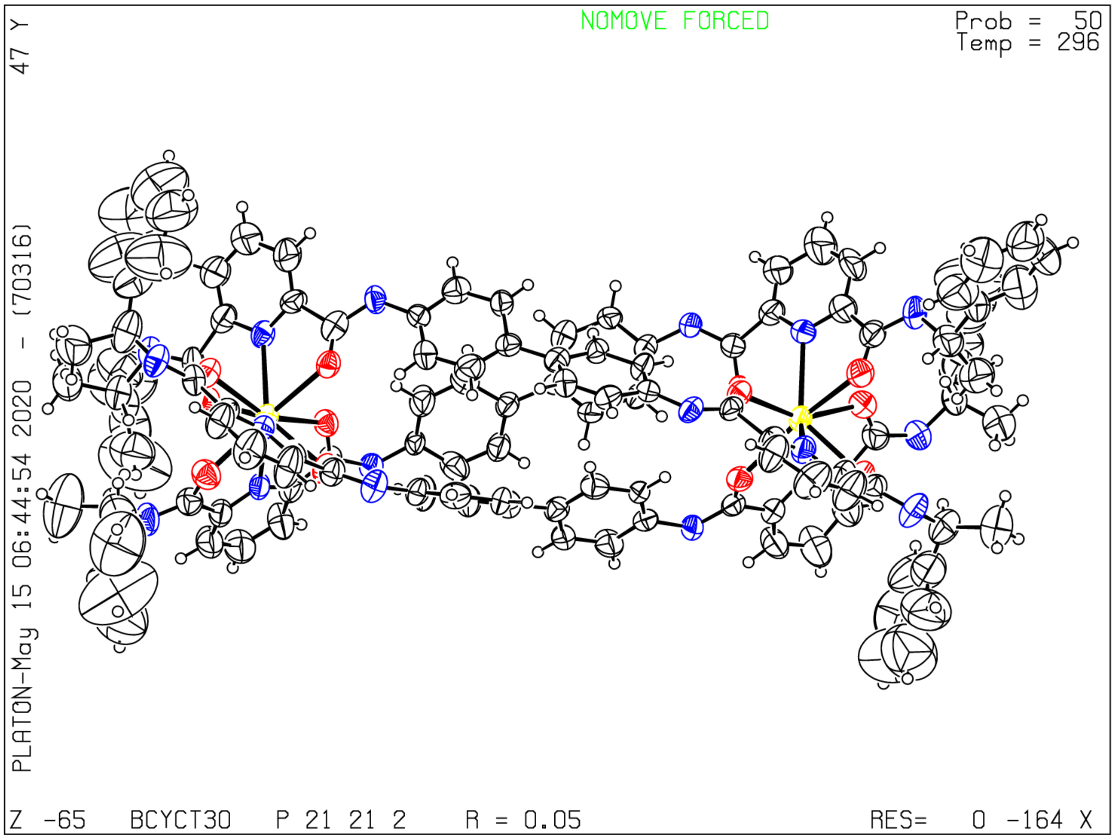

Supplement: Supplementary file 3 — Supplementary Data 1 [file BCYCT30_checkcif_new.pdf › Supplementary Data 1/BCYCT30_checkcif_new.pdf]
